# Real-world data analyses unveiled the immune-related adverse effects of immune checkpoint inhibitors across cancer types

**DOI:** 10.1038/s41698-021-00223-x

**Published:** 2021-09-10

**Authors:** Feicheng Wang, Shihao Yang, Nathan Palmer, Kathe Fox, Isaac S. Kohane, Katherine P. Liao, Kun-Hsing Yu, S. C. Kou

**Affiliations:** 1 Department of Statistics, Harvard University, Cambridge, MA USA; 2 Department of Biomedical Informatics, Harvard Medical School, Boston, MA USA; 3 H. Milton Stewart School of Industrial and Systems Engineering, Georgia Institute of Technology, Atlanta, GA USA; 4 Department of Medicine, Brigham and Women’s Hospital, Boston, MA USA; 5 Department of Pathology, Brigham and Women’s Hospital, Boston, MA USA

**Keywords:** Cancer, Cancer therapy

## Abstract

Immune checkpoint inhibitors have demonstrated significant survival benefits in treating many types of cancers. However, their immune-related adverse events (irAEs) have not been systematically evaluated across cancer types in large-scale real-world populations. To address this gap, we conducted real-world data analyses using nationwide insurance claims data with 85.97 million enrollees across 8 years. We identified a significantly increased risk of developing irAEs among patients receiving immunotherapy agents in all seven cancer types commonly treated with immune checkpoint inhibitors. By six months after treatment initialization, those receiving immunotherapy were 1.50–4.00 times (95% CI, lower bound from 1.15 to 2.16, upper bound from 1.69 to 20.36) more likely to develop irAEs in the first 6 months of treatment, compared to matched chemotherapy or targeted therapy groups, with a total of 92,858 patients. The risk of developing irAEs among patients using nivolumab is higher compared to those using pembrolizumab. These results confirmed the need for clinicians to assess irAEs among cancer patients undergoing immunotherapy as part of management. Our methods are extensible to characterizing the effectiveness and adverse effects of novel treatments in large populations in an efficient and economical fashion.

## Introduction

Immune checkpoint inhibitors have transformed the landscape of cancer treatments^1^. These new treatment agents have demonstrated a substantial survival benefit in various cancer types, and the U.S. Food and Drug Administration (FDA) has approved the use of immune checkpoint inhibitors for over a dozen types of cancers^2^, including lung cancer, renal cancer, head and neck cancers, melanoma, and many other types of skin cancers^3^. The survival gains observed in malignancies with traditionally poor prognosis and high rates of resistance to conventional therapies are unprecedented^4^. Many types of immune checkpoint inhibitors have been developed, including CTLA-4 inhibitors, PD-1 inhibitors, and PD-L1 inhibitors^5^. Drugs inhibiting the immune checkpoints work by enabling specific aspects of the immune system to target cancer cells^1,6,7^. Immunotherapy agents possess different mechanisms of action compared to conventional chemotherapy and targeted therapy, and as a result also have different side effect profiles, particularly with regard to immune-related adverse events (irAEs)^8^.

Despite the noteworthy success of immunotherapy in cancer treatment, irAEs are still not well understood at the large-scale population level (e.g., cohorts with millions of participants). Randomized controlled trials and small-scale observational studies described a number of side effects of immunotherapy, including fatigue, decreased appetite^9,10^, skin reactions, endocrine disorders, arthralgia^11^, pyrexia^12^, and drug-induced hepatitis^13^. Due to the immune-modulating effects of immune checkpoint inhibitors, these novel treatment agents may decrease the level of immunological tolerance^14^, resulting in autoimmune phenotypes. The risk for irAEs is related to the immunotherapy agent used, duration of treatment, dosage, and patients’ clinical characteristics^8^. Previous studies in this domain focused on individual adverse effects, such as endocrine dysfunction^15^ and fatal toxic effects^16^. Although it is reported that the majority of patients undergoing immune checkpoint blockade can develop irAEs (up to 90% of patients treated with an anti-CTLA-4 antibody and 70% of patients treated with a PD-1/PD-L1 antibody)^17^, many types of irAEs have relatively low incidence rates, making them very difficult to confirm with a high confidence level in previous studies based on a limited number of participants.

Real-world data (RWD) has demonstrated substantial promise in characterizing the effectiveness and adverse effects of novel treatment modalities^18–20^. RWD is often defined as the routinely collected information related to patient health status and healthcare delivery^18^, and the use of RWD allows for a better understanding of the benefits and adverse effects of treatments in large patient populations^21^. Previous studies have successfully leveraged RWD to investigate the adverse effects of chemotherapy^22^, the cardiotoxicity of targeted therapies^23^, and drug interactions^24^. One recent real-world population study showed the increased risk for irAEs among lung cancer patients undergoing immunotherapy^25^ and successfully identified hypothyroidism and other irAEs with high significance at the population level. Nevertheless, there remains a gap in knowledge for studies that systematically analyze and quantify the large-scale population-level risk for irAEs among patients receiving immune checkpoint blockade across multiple cancer types. Understanding the irAEs of immunotherapy on the large-scale population level will improve our understanding of this new type of cancer treatment and facilitate clinical decision-making^26^.

One major challenge of using RWD is the difficulty in establishing causality^27^. RWD is collected in an observational manner without any specific research question in mind, making it difficult to ascertain the effects of treatments in the study populations^21^. To address this issue, we employed the matching method in the causal inference literature^28^ to deduce the relationship between immunotherapy and subsequent adverse drug effects. Our analytical approaches do not rely on heavy model assumptions and can effectively identify the effects of treatments in large-scale clinical datasets.

In this study, we leveraged the health insurance claims of 85.97 million participants in the U.S. from 2008 to 2019. This large cohort of participants provides significantly higher statistical power and greater representativeness compared with previous studies^29,30^. We systematically identified cancer patients with no irAE before treatments and compared the irAE of immune checkpoint inhibitors and those of conventional cancer treatments. Through this data-driven analysis, we demonstrated a significant effect of immunotherapy on the subsequent development of irAEs, especially hypothyroidism and thyrotoxicosis. Our large-scale RWD analyses reveal adverse effects in patients with rarer malignancies with higher confidence in an inexpensive and more efficient way. Our described methods can be easily extended to investigate the effectiveness and adverse effects of other treatments using large-scale RWD.

## Results

### Overview of the study cohort and the trend of treatments

**Fig. 1:**
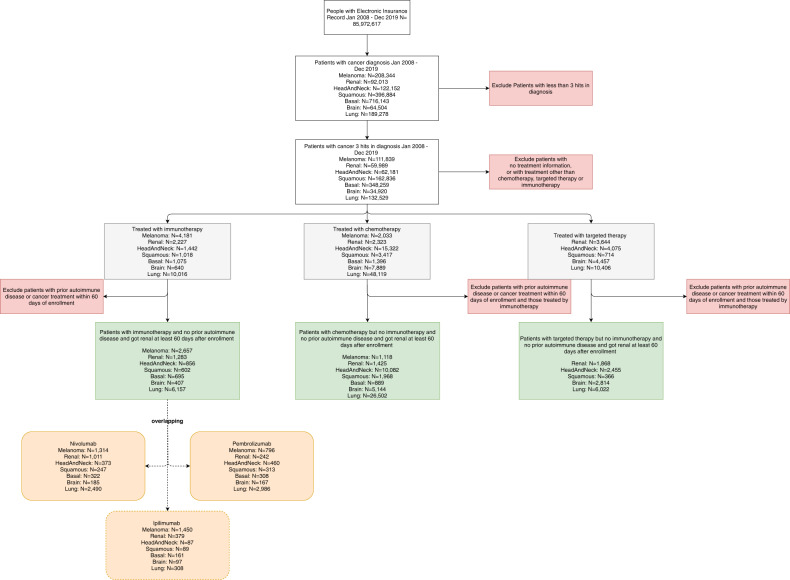
A summary of the patient cohorts in our study. The numbers of patients at each stage of cohort derivation were shown. The detailed inclusion and exclusion criteria can be found in the Methods section.

Following our inclusion and exclusion criteria, which will be introduced in the methods section, we identified patients with melanoma, lung, renal, head and neck, brain cancers, basal cell carcinoma, or squamous cell carcinoma of the skin from all 85,972,617 participants in the dataset (Fig. 1). The average enrollment period of the patients with any of the cancer types under investigation is ~5.2 years. For each of the seven cancer types under study, we obtained an immunotherapy treatment group and control groups consisting of patients receiving conventional chemotherapy or targeted therapy. Patients with irAEs before their cancer treatments were excluded from the study.

We summarized the number of patients receiving chemotherapy, targeted therapy, or immunotherapy of each cancer type of interest in Supplementary Fig. 14. Results showed that targeted therapy is relatively commonly used in lung, brain, head and neck, and renal cancers (6022 (4.5% of all patients with that cancer type), 2814 (8.0%), 2455 (3.9%), and 1868 (3.1%) patients respectively), and chemotherapy is commonly used in patients with lung (26,502 patients; 20.0%), head and neck (10,082 patients; 16.2%), or brain cancers (5144 patients; 14.7%). Across the seven cancer types, immunotherapy has the largest patient count in lung cancer (6157 patients; 4.6%), and melanoma (2657 patients; 2.4%), and the smallest patient number in brain cancer (407 patients; 1.2%). Among the patients receiving immunotherapy, nivolumab (4746 patients across the seven cancer types we evaluated) and pembrolizumab (4138 patients) are the two most commonly used agents for immune checkpoint blockade.

Supplementary Figure 1 shows the distribution of the initiation date of chemotherapy, targeted therapy, and immunotherapy. As an illustration, one can observe a sharp increase in immunotherapy among renal cancer patients after 2015 Q3, while the number of patients receiving chemotherapy and targeted therapy remains roughly at the same level. The treatment trends of other cancer types follow similar distributions (Supplementary Fig. 1).

We compared the demographics of our study cohort before and after covariate adjustment. Table 1 shows the detailed demographics of patients with renal cancer. Results for other cancer types can be found in Supplementary Tables 8–9. Before matching, the immunotherapy group and the control group had different age distributions in all cancer types, but the difference did not share a common pattern across the seven cancer types. For example, among patients with basal cell carcinoma, squamous cell carcinoma of the skin, and renal cancer, the immunotherapy groups were significantly older than the chemotherapy or targeted therapy groups. However, we observed a reversed trend among patients with brain cancer. We did not observe a significant difference in the racial distribution among the treatment groups, but the distribution of sex is considerably imbalanced for melanoma, basal cell carcinoma, renal cancer, and squamous cancer patients before matching. After matching, there is no significant difference in these covariates between the comparison groups (Supplementary Fig. 3). We achieved an almost perfect balance (absolute standardized difference <0.1) on sex, age, average income, the annual frequency of hospital visits prior to treatment therapy initialization, and the annual frequency of ICD code counts prior to treatment therapy initialization.

**Table 1:** Patient characteristics of the immunotherapy and chemotherapy groups in the renal cancer cohort.

Demographic characteristics	Pembrolizumab	%	Nivolumab	%	All immunotherapy drugs	%	Chemotherapy	%
	Number of patients		Number of patients		Number of patients		Number of patients	
*Sex*								
Both sexes	214	100.00	1003	100.00	1284	100.00	1425	100.00
Female	71	33.18	249	24.83	336	26.17	523	36.70
Male	143	66.82	754	75.17	948	73.83	902	63.30
Sex *p*-value	*p* = 0.3205		*p* < 1e-04		*p* < 1e-04		Control group	
*Age*								
(0,40)	<5	<2.34	29	2.89	34	2.65	195	13.68
(40,50)	7	3.27	76	7.58	86	6.7	109	7.65
(50,60)	29	13.55	256	25.52	295	22.98	301	21.12
(60,70)	72	33.64	358	35.69	449	34.97	413	28.98
(70,80)	64	29.91	217	21.64	304	23.68	291	20.42
(80,120)	38	17.76	67	6.68	116	9.03	116	8.14
Age *p*-value	*p* < 1e-04		*p* < 1e-04		*p* < 1e-04		Control group	
Matched	120	56.07	552	55.03	709	55.22	Control group	

Detailed profiles of patients with other cancer types and in other comparisons could be found in Supplementary Tables 8–9.

Suppressed patient counts <5 to protect patient privacy.

### Immunotherapy and the Risk of Developing irAEs

**Fig. 2:**
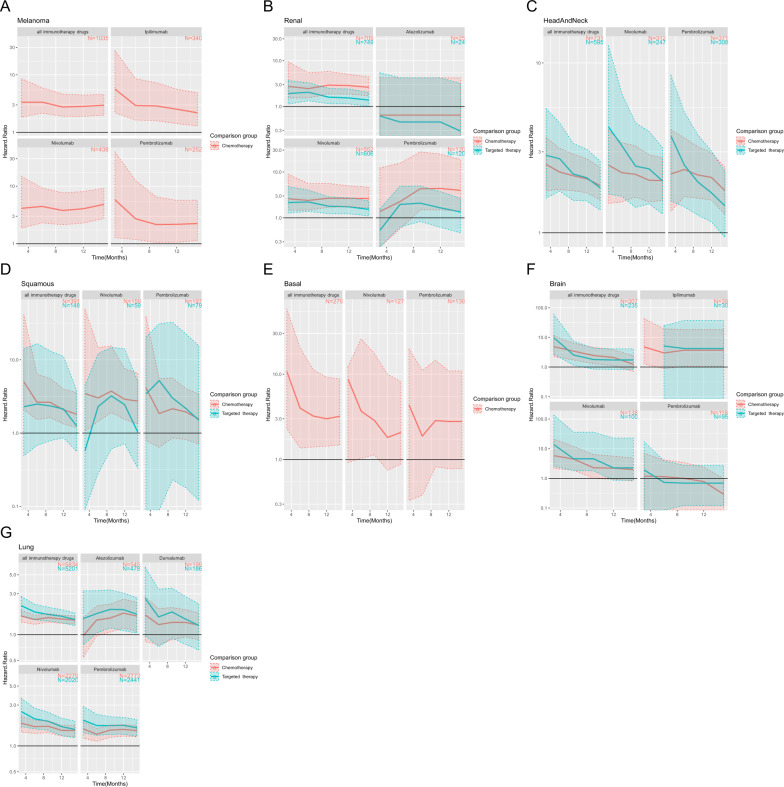
The immunotherapy group has a higher risk of developing subsequent irAEs across all cancer types under study. **A** Melanoma. **B** Renal cancer. **C** Head and neck cancer. **D** Squamous cell carcinoma. **E** Basal cell carcinoma. **F** Brain cancer. **G** Lung cancer. The point estimates of the hazard ratios comparing the immunotherapy group with either chemotherapy (red curves) or targeted therapy (cyan curves) in 3, 6, 9, 12, and 15 months after treatment initiation are shown. Their corresponding 95% confidence intervals are displayed by the shaded areas. Chemotherapy agents include carboplatin, cisplatin, doxorubicin, fluorouracil, gemcitabine, and paclitaxel. Targeted therapy agents include bevacizumab, temsirolimus, axitinib, cabozantinib, erlotinib, everolimus, pazopanib, sorafenib, and sunitinib. The detailed list of CPT/HCPCS procedure codes and national drug codes can be found in Tables S3–S6. The number on the top right indicates the number of matched patients in the treatment group. With the exception of renal cancer, the hazard ratios did not differ significantly when using the chemotherapy group or the targeted therapy group as the comparison group. With the exception of comparing immunotherapy with targeted therapy among patients with squamous cell carcinoma of the skin and brain cancer, the 95% confidence intervals in all other comparisons involving all immunotherapy drugs do not include 1.

We conducted detailed analyses on the risk of developing irAEs among patients receiving immunotherapy, chemotherapy, or targeted therapy using the matching method. For patients with any of the cancer types under evaluation, we found significantly increased irAEs risks in patients receiving immunotherapy compared to chemotherapy: 6-month adjusted hazard ratio = 2.64, 95% confidence interval = (1.53–6.34) in squamous cell carcinoma of the skin; 2.46 (1.54, 5.45) in renal cancer; 4.00 (1.36, 20.36) in basal cell carcinoma; 3.35 (2.16, 5.99) in melanoma; 2.28 (1.80, 3.14) in head and neck cancer; 3.42 (1.73, 6.33) in brain cancer; and 1.50 (1.32, 1.69) in lung cancer. When comparing immunotherapy to targeted therapy, the 6-month adjusted hazard ratios are 2.49 (0.68, 16.66) in squamous cell carcinoma of the skin, 2.73 (1.76, 4.44) in head and neck cancer, 2.54 (1.15, 7.39) in brain cancer, 1.84 (1.49, 2.26) in lung cancer, and 2.05 (1.33, 3.3) in renal cancer. Figure 2 provides an overview of the hazard ratios in 3, 6, 9, 12, and 15 months from treatment initialization across cancer types. Table 2 and Supplementary Figs. 10–11 provide the log-rank test *p*-value and 95% confidence intervals of the event (developing irAEs) probability and hazard ratios in different time intervals. In general, there is a downward trend in the hazard ratios as time elapses.

**Table 2:** The cumulative incidence rate (%) of irAEs of the immunotherapy and the chemotherapy groups in the renal cancer cohort as well as the hazard ratios between them.

Time	Immunotherapy	Chemotherapy	Hazard Ratio	Immunotherapy	Chemotherapy	Hazard Ratio
	Matched			Unmatched		
Pembrolizumab	Log-rank test p value = 1.30E-02; number of patients receiving pembrolizumab = 120			Log-rank test *p* value = 9.80E-12; number of patients receiving pembrolizumab = 214		
Month = 3	4.27 [0.05, 8.31]	3.13 [0, 6.26]	1.37 [0, 12.25]	5.25 [1.8, 8.59]	2.08 [1.32, 2.84]	2.52 [1.1, 5.26]
Month = 6	13.91 [5.25, 21.78]	6.15 [1.19, 10.86]	2.26 [0.57, 15.6]	15.43 [8.38, 21.94]	3.82 [2.72, 4.9]	4.04 [2.29, 7.91]
Month = 9	26.46 [12.84, 37.95]	6.15 [1.19, 10.86]	4.3 [1.49, 27.45]	25.53 [15.1, 34.68]	5.08 [3.76, 6.38]	5.03 [3.03, 7.97]
Month = 12	30.14 [14.89, 42.65]	6.89 [1.32, 12.15]	4.37 [1.49, 24.54]	30.97 [18.32, 41.66]	7.34 [5.64, 9.01]	4.22 [2.41, 6.46]
Month = 15	30.14 [14.89, 42.65]	7.58 [1.37, 13.41]	3.97 [1.4, 18.61]	34.26 [20.22, 45.83]	8.89 [6.93, 10.8]	3.86 [2.34, 5.65]
Nivolumab	Log-rank test *p* value = 3.35E-13; number of patients receiving nivolumab = 552			Log-rank test *p* value < 1.40E-45; number of patients receiving nivolumab = 1003		
Month = 3	9.66 [6.98, 12.26]	3.71 [2.05, 5.34]	2.6 [1.47, 8.89]	10.44 [8.39, 12.45]	2.08 [1.32, 2.84]	5.02 [3.13, 8.69]
Month = 6	15.71 [12.25, 19.05]	6.55 [4.22, 8.83]	2.4 [1.47, 5.57]	16.63 [14, 19.17]	3.82 [2.72, 4.9]	4.36 [3.24, 6.06]
Month = 9	21.18 [16.96, 25.19]	7.91 [5.27, 10.48]	2.68 [1.7, 5.62]	23.13 [19.9, 26.23]	5.08 [3.76, 6.38]	4.56 [3.32, 6.4]
Month = 12	26.77 [21.79, 31.44]	10.05 [6.91, 13.09]	2.66 [1.74, 4.99]	28.2 [24.51, 31.71]	7.34 [5.64, 9.01]	3.84 [2.89, 4.82]
Month = 15	30.89 [25.24, 36.12]	11.73 [8.17, 15.16]	2.63 [1.66, 4.63]	31.3 [27.26, 35.11]	8.89 [6.93, 10.8]	3.52 [2.82, 4.4]
All immunotherapy drugs	Log-rank test *p* value = 1.63E-15; number of patients receiving any immunotherapy drugs = 709			Log-rank test *p* value < 1.40E-45; number of patients receiving any immunotherapy drugs = 1284		
Month = 3	9.25 [6.92, 11.53]	3.37 [1.97, 4.74]	2.75 [1.54, 9.43]	9.42 [7.67, 11.12]	2.08 [1.32, 2.84]	4.52 [3.13, 7.5]
Month = 6	15.2 [12.11, 18.19]	6.18 [4.18, 8.15]	2.46 [1.54, 5.45]	15.77 [13.44, 18.03]	3.82 [2.72, 4.9]	4.13 [3.07, 5.67]
Month = 9	20.95 [17.07, 24.65]	7.14 [4.93, 9.29]	2.93 [1.88, 5.81]	22.4 [19.46, 25.23]	5.08 [3.76, 6.38]	4.41 [3.44, 5.68]
Month = 12	26.04 [21.46, 30.35]	9.08 [6.42, 11.67]	2.87 [1.86, 5.05]	27.35 [23.96, 30.59]	7.34 [5.64, 9.01]	3.73 [2.86, 4.79]
Month = 15	29.76 [24.57, 34.59]	11.29 [8.11, 14.37]	2.63 [1.71, 4.42]	30.34 [26.63, 33.87]	8.89 [6.93, 10.8]	3.41 [2.53, 4.06]

The hazard ratios in the matched columns of this table correspond to the red curve of renal cancer in Fig. 2. The numbers in the square brackets are 95% confidence intervals. The total number of patients who receive each immune checkpoint inhibitor was specified. For example, there are 120 matched (214 unmatched) renal cancer patients who are treated by pembrolizumab. Both matched and unmatched results are shown. Summary tables of other cancer types can be found in Supplementary Tables 10–11.

**Fig. 3:**
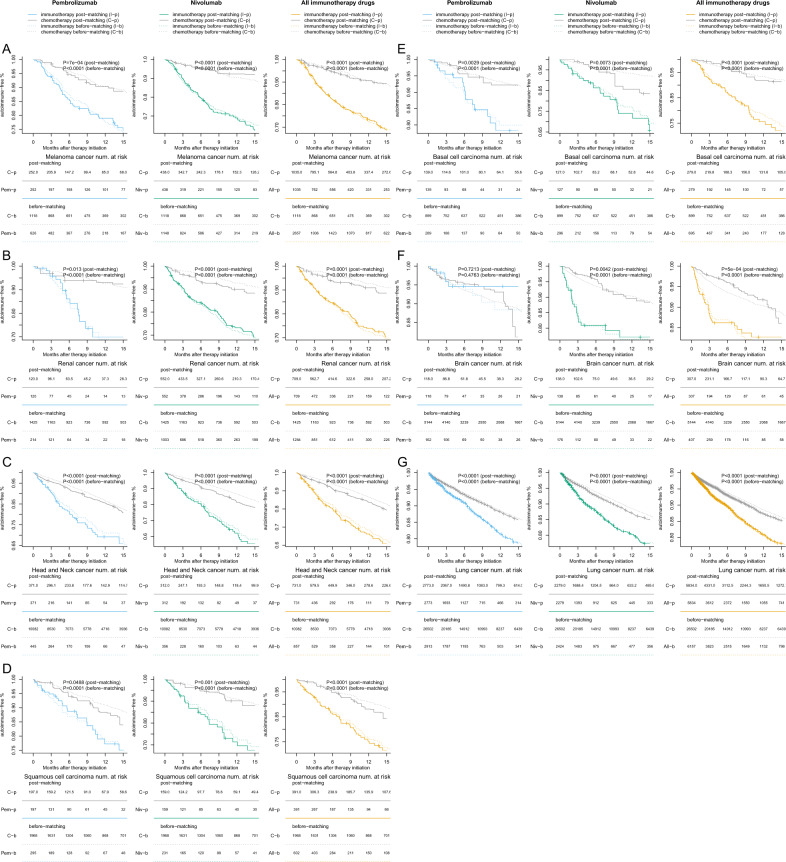
Time-to-event plot showing the time between treatment initiation and the development of irAEs in seven cancer types. **A** Melanoma. **B** Renal cancer. **C** Head and neck cancer. **D** Squamous cell carcinoma. **E** Basal cell carcinoma. **F** Brain cancer. **G** Lung cancer. The comparisons between chemotherapy with pembrolizumab, nivolumab, and all immune checkpoint inhibitors combined are shown in the three columns respectively. The top panel is the time-to-event plot for all irAEs, with a log-rank test *p*-value reported on the top right corner for both matched and unmatched samples. Each tick represents 50 censored patients. The curves are truncated in the 15th month after treatment initiation for better visualization.

We further conducted drug-specific analyses by comparing the risk for irAEs of the two most widely used immune checkpoint inhibitors (nivolumab and pembrolizumab) to that of chemotherapy or targeted therapy. Results showed that for patients with lung, melanoma, head and neck, brain, and renal cancers, those treated with nivolumab have a significantly higher risk of developing irAEs. However, for pembrolizumab, only the analyses for head and neck and lung cancer patients showed significantly increased risk for irAEs compared to conventional treatments, which is likely due to the reduced sample size in this analysis (Fig. 3).

We plotted the time-to-event curves for the different treatment groups with the seven cancer types (Fig. 3). Results showed that the difference in irAE-free survival between the immunotherapy group and that of either the chemotherapy or targeted therapy group is statistically significant. For chemotherapy, we have *P* < 0.0001 for head and neck cancers, melanoma, renal cancer, lung cancer, basal cell carcinoma, and squamous cell carcinoma of the skin; *P* = 0.0005 for brain cancer. For targeted therapy, we have *P* < 0.0001 for head and neck cancers, lung cancer, and renal cancers; *P* = 0.0396 for squamous cell carcinoma of the skin; *P* = 0.0027 for brain cancer.

The median onset time of irAEs in our analysis matches with those reported in the literature. After immunotherapy, gastrointestinal side effects usually occur within 6 weeks; hepatitis occurs from 1 to 49 weeks, with a median duration of 5 weeks; endocrine toxicity usually occurs in 7–10 weeks^31^. In all seven cancers we considered, the median onset time after immunotherapy is 8–16 weeks for inflammatory bowel disease (IBD), 13–22 weeks for hepatitis, 12–24 weeks for hypothyroidism, and 4–6 weeks for thyrotoxicosis.

To better compare the specific effects from each treatment group, we conducted a sensitivity analysis comparing patients who received immunotherapy only with those who were treated with chemotherapy only or targeted therapy only. Results showed that a significantly increased risk for irAEs persists in the immunotherapy group (Supplementary Fig. 17). In another sensitivity analysis, we excluded patients receiving more than one type of immune checkpoint inhibitor (12% of the immunotherapy group). We showed that the significantly increased autoimmune side effects in the immunotherapy group persist when compared with the chemotherapy group, with the exception of brain cancer due to reduced sample size. When compared with the targeted therapy group, lung and head and neck cancer patients undergoing single-agent immune checkpoint blockade also had a significantly increased autoimmune risk. These results indicated that single-agent immune checkpoint blockade may still increase the risk of developing autoimmune side effects.

### Identification of specific irAEs related to immunotherapy

**Fig. 4:**
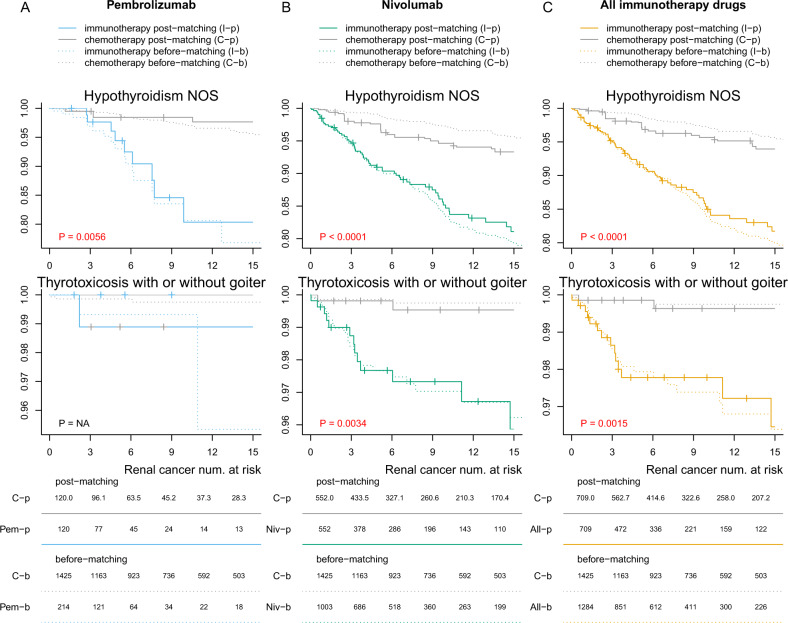
Time-to-event plot showing the time between treatment initiation and the development of specific irAEs. The comparisons between renal cancer patients receiving chemotherapy with **A** pembrolizumab, **B** nivolumab, and **C** all immune checkpoint inhibitors are shown in three separate columns. We examined 56 irAEs separately, with *p*-values corrected by the Benjamini–Hochberg procedure. Adjusted *p*-values < 0.05 were highlighted in red. Renal cancer patients receiving nivolumab have higher risks of developing hypothyroidism and thyrotoxicosis, but those receiving pembrolizumab did not have a significantly increased risk. Each tick represents 50 censored patients. The curves are truncated in the 15th month after treatment initiation for better visualization. The numbers at the bottom panel indicate the number of remaining patients at 0, 3, 6, 9, 12, and 15 months after treatment initiation. Plots for all other cancer types can be found in Supplementary Fig. 2; plots for a longer time horizon can be found in Supplementary Fig. 4.

**Fig. 5:**
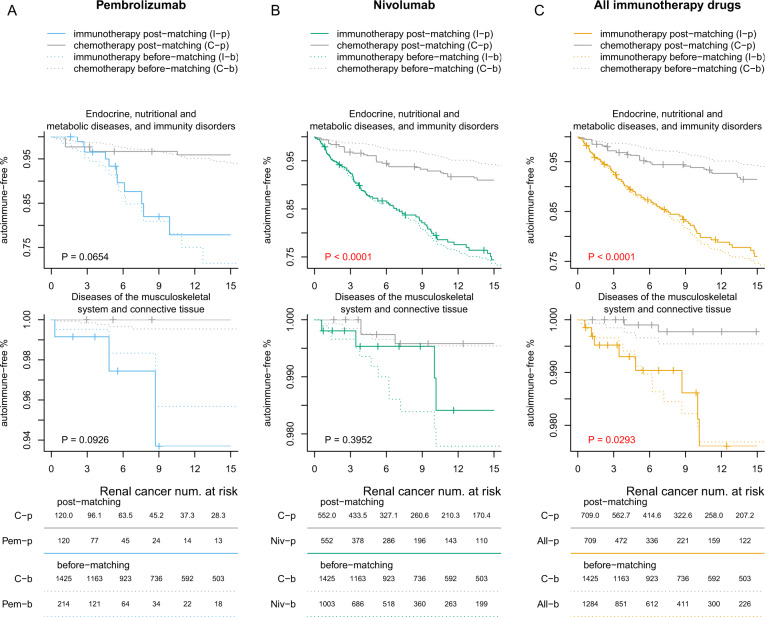
Time-to-event plot showing the time between treatment initiation and the development of irAE groups^32^. **A** Pembrolizumab. **B** Nivolumab. **C** All immune checkpoint inhibitors.

To identify the specific types of irAEs related to immunotherapy, we conducted a set of independent analyses that examined the risk of developing each of the 56 irAE categories and corrected for multiple tests by the Benjamini–Hochberg procedure (Fig. 4). We found that there was a significantly higher risk of developing hypothyroidism among patients receiving immunotherapy compared with patients receiving chemotherapy (*P* < 0.0001 for head and neck cancer, renal cancer, lung cancer, basal cell carcinoma, and melanoma, *P* = 0.0002 for squamous cell carcinoma, and *P* = 0.0006 for brain cancer) and patients receiving targeted therapy (*P* < 0.0001 for head and neck cancer and lung cancer, *P* = 0.0002 for renal cancer, and *P* = 0.0007 for brain cancer). Among patients with lung cancer, melanoma, and renal cancer, those receiving immunotherapy have a significantly higher risk of acquiring thyrotoxicosis when compared with chemotherapy (*P* < 0.0001 for lung cancer, *P* = 0.0095 for melanoma, and *P* = 0.0015 for renal cancer). In addition, we found that the immunotherapy group has a higher risk of acquiring autoimmune hepatitis (*P* = 0.0096) among lung cancer patients compared with the chemotherapy group. However, the risk of developing autoimmune hepatitis is not statistically significant among patients with other cancer types under study compared with the chemotherapy group. We also found significantly higher risk of several types of irAEs among patients with lung cancer when comparing with the targeted therapy group, including hypothyroidism (*P* < 0.0001), inflammatory and toxic neuropathy (*P* < 0.0001), psoriasis (*P* = 0.0040), thyrotoxicosis (*P* < 0.0001), RA (*P* = 0.0270), thyroiditis (*P* = 0.0270), pemphigus and pemphigoid (*P* = 0.0349), immune-related hepatitis (*P* = 0.0045), and myalgia and myositis (*P* = 0.0349). We employed the ICD chapters^32^ to group the adverse effects by their categories in Fig. 5 and plotted grouping thyroid disorders in Supplementary Fig. 15.

Interestingly, the immunotherapy group had a lower risk of getting inflammatory and toxic neuropathy (*P* < 0.0001) among patients with lung cancer compared with either the chemotherapy group or the targeted therapy group. We did not find lower risks of developing any other autoimmune disorders in the immunotherapy group in other cancer types.

## Discussion

Our RWD analyses successfully revealed the irAEs of immune checkpoint inhibitors across seven cancer types at the population level. Immune checkpoint inhibitors have gained a pivotal role in cancer treatment due to their significant clinical response in many cancer types^33^. However, since it is difficult to assemble a large cohort to study the long-term irAEs of immunotherapy across multiple cancer types, the existing literature mostly draws conclusions based on studies with relatively small sample sizes^8^. In this study, we highlight the important role of RWD analyses in providing the increased statistical power required to detect rare adverse events and provide reliable risk estimates. We leveraged a large-scale health insurance dataset to quantify the risk of developing irAEs among patients receiving immunotherapy, chemotherapy, and targeted therapy in seven cancer types. We demonstrated that patients undergoing immunotherapy had a significantly increased risk of acquiring irAEs, especially hypothyroidism and thyroid dysfunction^34^. While clinicians are aware of the associations between immunotherapy and irAEs, this study revealed the scope of these irAEs spanning across cancer types and different immunotherapy agents, confirming the need to proactively assess irAEs as part of management.

Immune checkpoint inhibitors specifically target molecules in the immune system, and thus they do not have significant cytotoxicity or other common adverse effects of conventional cancer treatments^8^. However, it has been reported that these novel treatment agents may result in irAEs^35^. Consistent with the previous reports, we found that hypothyroidism and thyroid dysfunction are frequently diagnosed after immune checkpoint blockade^36,37^. Our results on the irAEs among lung cancer patients are consistent with a previous study using observational data prior to 2017^25^. It is also suggested that the risk of developing irAEs is associated with the response to immunotherapy treatment^37^, highlighting the dual effects of manipulating immune checkpoint pathways in treating cancer.

Our analyses showed that immunotherapy presented a risk of developing irAEs in patients with all seven cancer types under study. However, we observed a slight difference in the risk for irAEs across the cancer types compared to a matched chemotherapy or targeted therapy groups. In the nivolumab group, patients with head and neck cancer had the highest risk of developing irAEs (15-month cumulative incidence = 43.63%), while the risk for irAEs among those with brain cancer is the lowest (15-month cumulative incidence = 21.79%). In the pembrolizumab group, patients with head and neck cancer had the highest risk of developing irAEs (15-month cumulative incidence = 32.36%), while the risk for irAEs among those with brain cancer is the lowest (15-month cumulative incidence = 12.07%). These differences may be related to the different susceptibility of patients with different cancer types.

With the exception of renal cancer, the hazard ratio for irAEs of the immunotherapy group compared to the chemotherapy group was similar to that of the immunotherapy group compared to the targeted therapy group. For renal cancer, we observed a lower hazard ratio for irAEs of immunotherapy versus targeted therapy compared with immunotherapy versus chemotherapy. These findings suggest a slightly higher risk for irAEs among patients receiving targeted therapy compared to those receiving chemotherapy in this cancer type. The higher risk may be due to the VEGF targeted agents, as they are commonly used in treating renal cell carcinoma and may increase the risk of developing autoimmune side effects^38^ due to inhibition of iodine uptake, hindrance of the proper functioning of VEGFR in the thyroid, or immune-mediated effects. With regards to individual types of irAEs, the risk of developing hypothyroidism was significantly higher in the immunotherapy group across all seven cancer types. In addition, we observed an increased risk of getting thyrotoxicosis among patients with melanoma, lung cancer, and renal cancer patients who received immunotherapy. These results highlight both shared clinical manifestations as well as cancer-specific differences in the irAEs of immune checkpoint inhibitors across cancer types. Our RWD analyses complement results from trials^39,40^ by demonstrating the extent of irAEs among patients in a representative nationwide cohort, many of whom may not be eligible to participate in clinical trials due to declining health conditions. The large number of patients in the claims data further enabled the detection of less common irAEs systematically. For example, we identified inflammatory neuropathy, psoriasis, pemphigus and pemphigoid, and myositis as significant adverse effects among lung cancer patients, all with Benjamini & Hochberg adjusted *p*-value < 0.05.

One limitation of the study is the level of details that can be obtained on disease status using ICD codes. Since the ICD codes cannot provide information on cancer stage, grade, genomic variations, tumor mutation burden, or PD-L1 expression levels, we cannot perform detailed subgroup analyses to pinpoint the specific predictors of susceptibility to irAEs. In addition, it is difficult to characterize the detailed severity of irAEs due to the limited granularity of ICD codes. Future studies using data from the electronic health records which contain laboratory results or medical notes can further provide details on the nature and extent of the identified adverse effects^12,41–44^. Furthermore, most of our study participants received either pembrolizumab or nivolumab. As the usage of other immune checkpoint inhibitors and combinatorial immunotherapy increases, future analyses can focus on the adverse effects of these treatments. Lastly, our study population is restricted to those in North America, and not every participant had available race information in the dataset. Future studies on diverse populations can further characterize the risk for irAEs of immunotherapy across different populations.

Our study demonstrated the utility of RWD analyses in tracking the long-term irAEs of immunotherapy in seven cancer types at the population level. Our results verified a strong connection between the use of immune checkpoint inhibitors and subsequent autoimmune phenotypes, especially hypothyroidism and thyrotoxicosis. In addition, using RWD allowed us to study the impact of immune checkpoint inhibitors on populations not eligible or included in phase 3 clinical trials, and in whom we have limited data to inform clinical management. In this study, we demonstrate the use of RWD analyses to fill in this gap in knowledge surrounding irAEs among a broad population of patients actually receiving immune checkpoint inhibitors. Future studies can focus on characterizing the molecular mechanisms leading to irAEs and designing novel molecules with high antitumor potency while minimizing adverse immune effects. The methods we developed for large-scale RWD analyses can reveal the effectiveness and adverse effects of other novel cancer treatment regimens, expediting the validation of biomedical hypotheses at scale.

## Methods

### Overview of the insurance claims dataset

Using un-identifiable member claims data from Aetna, a nationwide managed care plan, we constructed the study population for this RWD analysis. The whole dataset includes members’ claims from 1 January 2008 to 31 December 2019, with a total of 85.97 million unique U.S. members across this time period. Written informed consent was obtained from the participants at their insurance plan enrollment. This study was approved by the Harvard Medical School Institutional Review Board. The funding sources had no role in the design and conduct of the study; collection, management, analysis, and interpretation of the data; preparation, review, or approval of the manuscript; and the decision to submit the manuscript for publication.

The claims data include diagnostic codes encoded by the International Statistical Classification of Diseases and Related Health Problems ninth revision (ICD9) and tenth revision (ICD10) as well as the Current Procedural Terminology (CPT) and Healthcare Common Procedure Coding System (HCPCS) treatment procedure codes of the patients for every service and procedure, together with the date of service. In addition, the claims dataset contains National Drug Codes (NDC) for the drugs prescribed and the date of dispense for all available outpatient prescription data of our study cohort. We also obtained the participant enrollment data, including enrollment status, age, sex, race (15% available), and zip codes.

### Cancer type identification

Guided by the PheWAS codes and descriptions^45^, we curated a list of ICD9 and ICD10 codes to identify the seven cancer types with the largest number of patients undergoing immune checkpoint blockade. These include lung cancer, melanoma, renal cancer, head and neck cancers, basal cell carcinoma, brain cancer, and squamous cell carcinoma of the skin. With the exception of brain cancer, the U.S. FDA has approved the use of immune checkpoint inhibitors in patients with each of these cancer types. Table S1 shows the complete list of ICD codes we used for cancer type identification.

### Inclusion and exclusion criteria

Figure 1 summarized the inclusion and exclusion criteria of this study. To reduce the false-positive rate for cancer patient identification, we include a patient in our study only if he or she had at least three diagnostic codes of the same cancer type on different days within 18 consecutive months^25^. This procedure ensures that patients enrolled in our study have a high likelihood of harboring the cancer type of interest and reduces the probability of miscoding.

We excluded patients who have developed autoimmune phenotypes before they were diagnosed with cancer since we cannot reliably estimate the irAEs of cancer treatments in this subgroup of patients using the insurance claims dataset. We further excluded patients who had chemotherapy, targeted therapy, or immunotherapy within 60 days of insurance enrollment, because these patients may have been diagnosed with cancer and potentially received other treatments before they were enrolled in the insurance plan.

### Immunotherapy, chemotherapy, and targeted therapy identification

To identify the patients undergoing immunotherapy, chemotherapy, and targeted therapy, we manually curated lists of immunotherapy, chemotherapy, and targeted therapy agents for each cancer type of interest. We identified immunotherapy and chemotherapy drugs for all cancer types under investigation and targeted therapy drugs for renal cancer, head and neck cancers, brain cancer, lung cancer, and squamous cell carcinoma of the skin, since targeted therapy is commonly used in patients with these cancer types. We omitted the analyses of targeted therapy in basal cell carcinoma and melanoma because of the limited sample size (<200 patients in the dataset underwent targeted therapy). Table S2 shows the CPT/HCPCS and NDC codes for immunotherapy, and Tables S3–S6 summarized the CPT/HCPCS and NDC codes for chemotherapy and targeted therapy. To ensure that no major treatment code is missed, for each cancer type, we generated a list of CPT/HCPCS and NDC codes our participants received, grouped the entries of the list by their standardized description (the “CHEMOCAT” string, a standardized description of drugs based on their generic and brand names), sorted the codes by the numbers of patients receiving the treatment, and manually reviewed the top 30 most common treatments for each cancer type. This approach ensured that the common codes for immunotherapy, chemotherapy, and targeted therapy agents were correctly identified.

### irAE identification

Guided by the PheWAS codes and descriptions, we manually curated a list of irAEs and their corresponding ICD-9 and ICD-10 codes. We followed the conventions from previous RWD analyses^46^ in identifying the disease codes with autoimmune components. Although some of the codes may not be very specific, they enabled large-scale characterization of irAEs. Table S7 shows the complete list.

### Matching methods

We defined our treatment group as patients who received immunotherapy and control groups as those who received other forms of treatments (such as chemotherapy and targeted therapy) but have never received immunotherapy. We employed the matching methods from the causal inference literature^28^ to account for the potential covariate imbalance between the two groups.

Each patient in the treatment group is matched to multiple patients in the control group^47^. The matching criteria are as follows: we match exactly on both sex and race (when the information is available), and we require that the difference in age between treatment and control patients be <2 years. Three other features are also matched: the zip-code-derived median household income (derived from the Census Reporter; https://censusreporter.org/), the rate of hospital visits before treatment initialization, and the rate of ICD codes before treatment initialization. Matching on these three factors aimed at accounting for the income level, healthcare utilization rate, and baseline health status of the participants respectively. For the matching factors that are continuous by nature, including the zip-code-derived median household income, the rate of hospital visits before treatment initialization, and the rate of ICD codes before treatment initialization, we divided the traits into five quantile bins and matched the patients who belong to the exact same bin for every trait. These matching factors aimed to account for the potential differences in baseline health status and socioeconomic status between the treatment and the control groups.

After matching, we reweighted each of the matched control samples by inverse probability weighting. Specifically, if three participants in the control group were matched to the same patient in the treatment group, we assign the weight 1/3 to each of these three participants in the control group.

We compared the risk for irAEs among patients receiving any immunotherapy to that of patients receiving conventional treatments. We further conducted a drug-specific analysis to compare the risk of patients receiving specific immunotherapy agents (e.g., pembrolizumab and nivolumab) to those receiving conventional treatments. If patients used a combination of more than one immune checkpoint inhibitor, they are counted once in each separate immunotherapy group. We plotted the Kaplan–Meier curves of the matched samples and tested whether their distribution of irAE-free survival was different by the log-rank test. We further calculated the 95% confidence interval for the hazard ratios of both the matched and the unmatched analyses using the bootstrap method^48^.

### Sensitivity analyses

To ensure the robustness of our results, we conducted extensive sensitivity analyses to demonstrate that our results were not sensitive to the differences in the design of our analyses. In addition to the 60 days quiescence period after insurance enrollment (which was used to exclude patients who might have received cancer treatments before enrollment), we used 10 days, 30 days, 90 days, and 180 days as the quiescence periods in our sensitivity analyses. We also employed different numbers of bins (3, 4, and 5) for matching the continuous variables. Since most patients received the first immunotherapy after 2015, we further conducted the analyses using only the data after 2015. To identify the potential effect modifications among age, sex, and income groups, we conducted stratified analyses based on age, sex, and income.

### Reporting summary

Further information on research design is available in the Nature Research Reporting Summary linked to this article.

## Supplementary information

Supplementary Information

Reporting Summary

## Data Availability

The consent acquired did not permit making data publicly available. However, the summary data that support the findings of this study are available from the authors upon reasonable request.

## Code availability

The codes that support the findings of this study are available from the authors upon reasonable request.
