# Supplementary material for: Real-world data analyses unveiled the immune-related adverse effects of immune checkpoint inhibitors across cancer types
Source: NPJ Precis Oncol. 2021 Sep 10;5:82. doi: 10.1038/s41698-021-00223-x (PMC8433190; doi:10.1038/s41698-021-00223-x)
Supplement: Supplementary file 1 — Supplementary Information [file 41698_2021_223_MOESM1_ESM.pdf]

Real-World Data Analyses Unveiled the Immune-Related Adverse Effects of Immune Checkpoint Inhibitors across Cancer Types

Supporting Information

The organization of this supporting information is as follows: (1) the distribution of the initiation date of chemotherapy, targeted therapy, and immunotherapy for all seven cancer types (Figure S1); (2) time-to-event plot showing the time between treatment initiation and the development of specific autoimmune diseases within 15 months for all seven cancer types (Figure S2); (3) examination of covariate balance (Figure S3); (4) time to autoimmunity analysis with the full time horizon (Figures S4-S5); (5) detailed results of additional sensitivity and robustness studies (Figures S6-S9); (6) additional subpopulation analyses (Figures S10-S13); (7) the number of patients undergoing chemotherapy, targeted therapy, and immunotherapy across 7 different types of cancers (Figure S14); (8) time-to-event plot showing the time between treatment initiation and the development of immune adverse event groups (Figures S15-S16); (9) sensitivity analysis excluding the patients with multiple treatments (ex. immunotherapy and chemotherapy) and patients with multiple immunotherapies (ex. nivolumab and ipilimumab) (Figures S17-S18); (10) detailed data summary (Tables S1-S11).

Figure S1. The distribution of the initiation date of chemotherapy, targeted therapy, and immunotherapy for 7 different cancers after 2012.

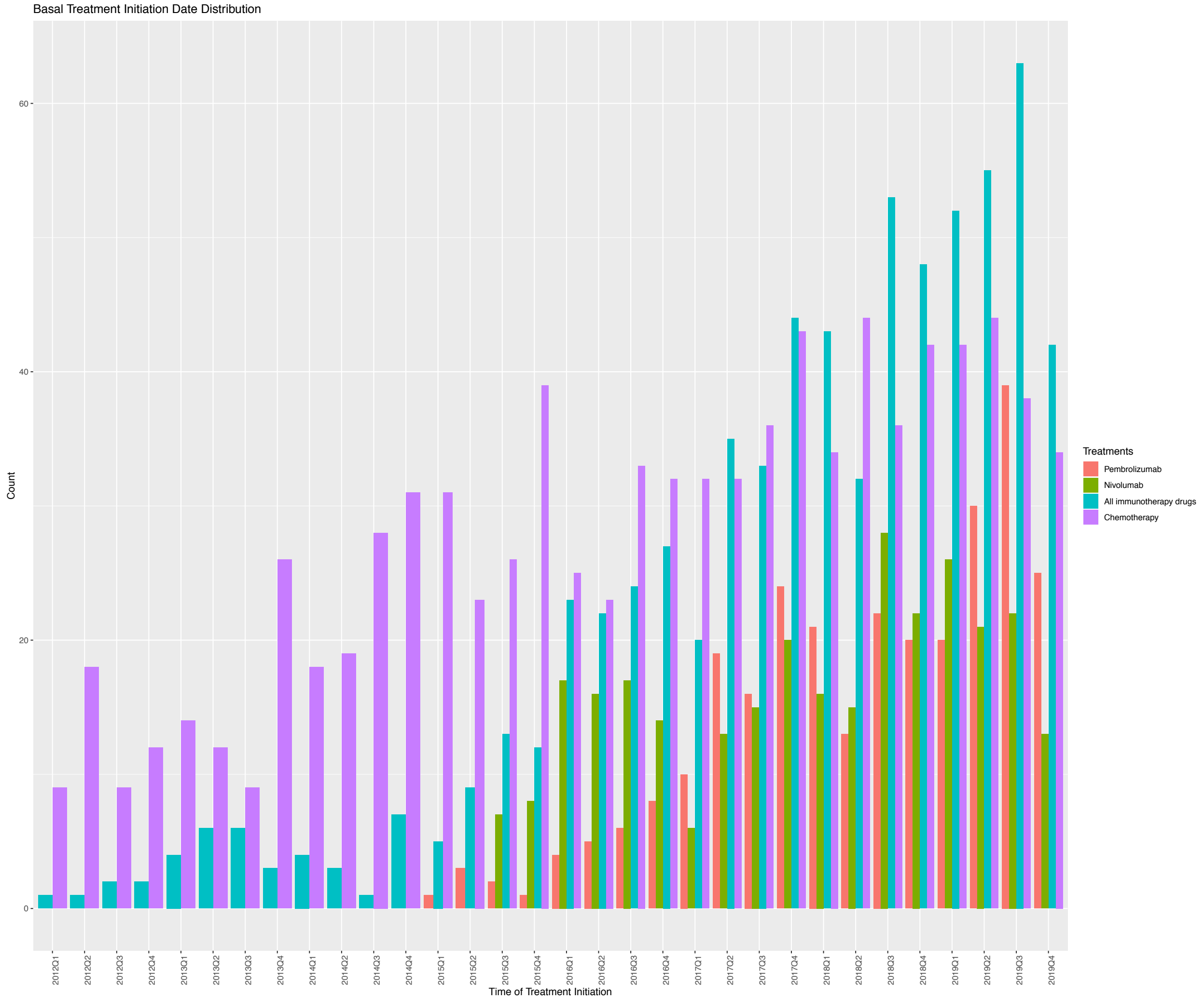

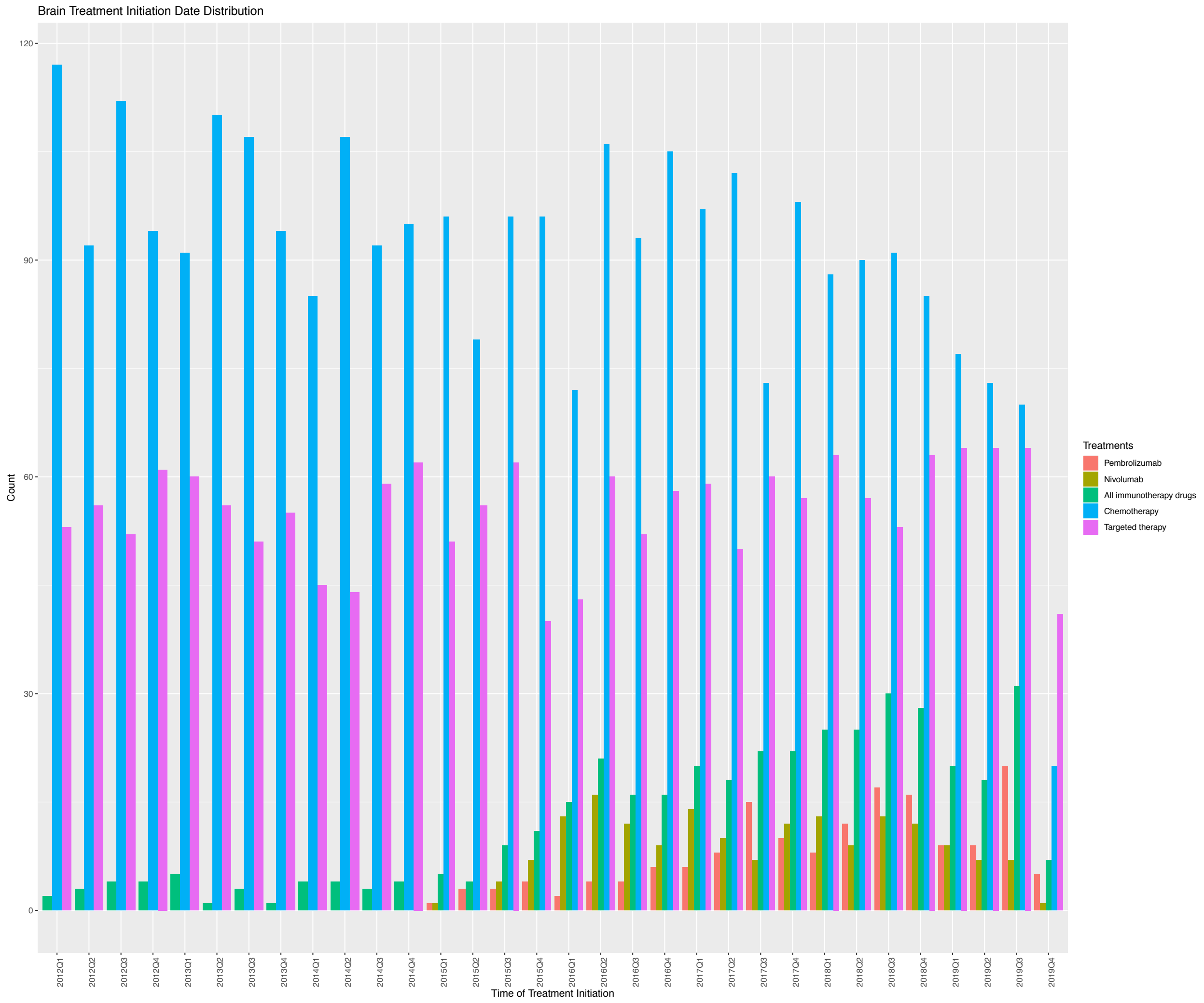
Brain Treatment Initiation Date Distribution
Count
Time of Treatment Initiation
0
30
60
90
120
2012Q1
2012Q2
2012Q3
2012Q4
2013Q1
2013Q2
2013Q3
2013Q4
2014Q1
2014Q2
2014Q3
2014Q4
2015Q1
2015Q2
2015Q3
2015Q4
2016Q1
2016Q2
2016Q3
2016Q4
2017Q1
2017Q2
2017Q3
2017Q4
2018Q1
2018Q2
2018Q3
2018Q4
2019Q1
2019Q2
2019Q3
2019Q4
Treatments
Pembrolizumab
Nivolumab
All immunotherapy drugs
Chemotherapy
Targeted therapy

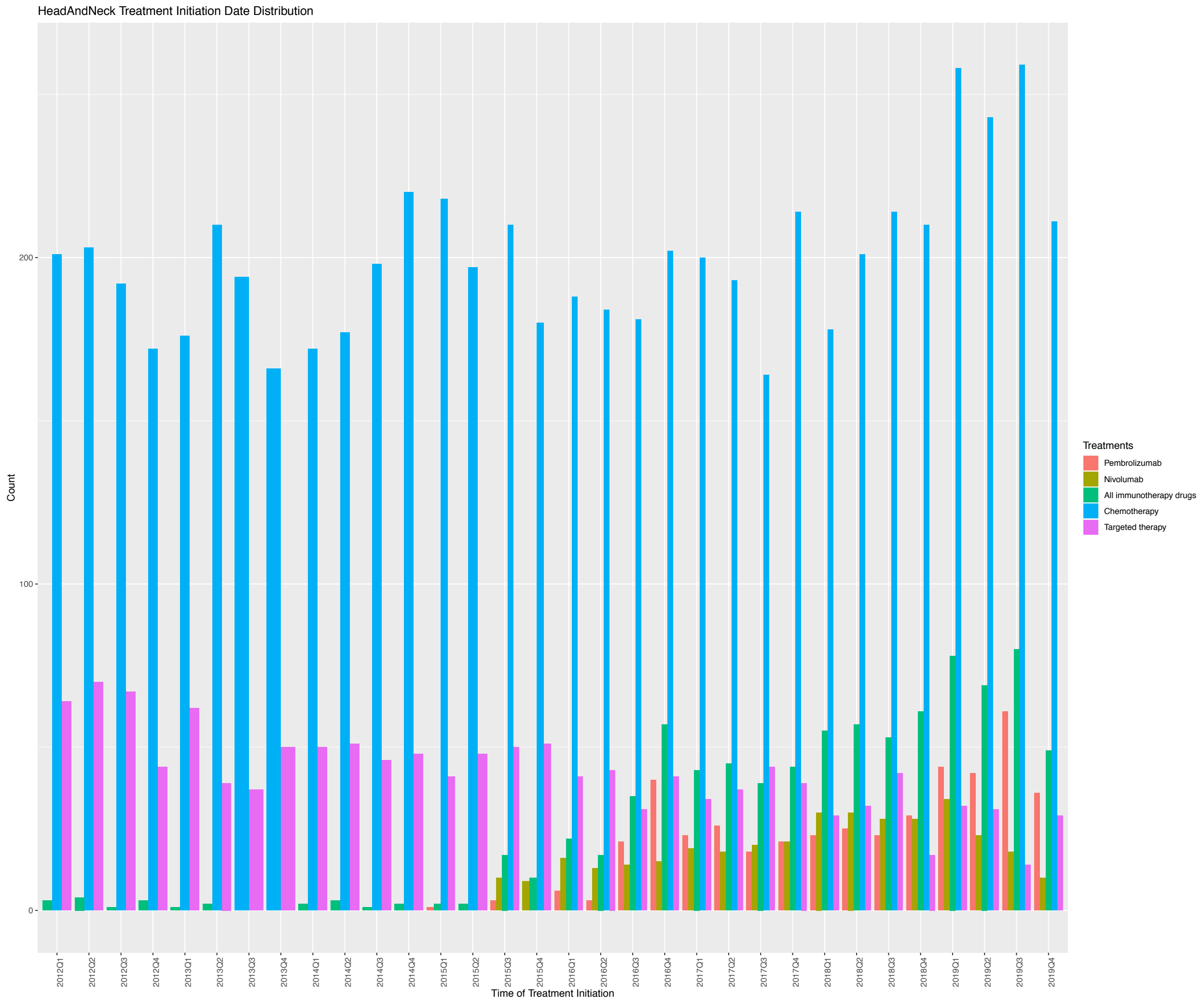
HeadAndNeck Treatment Initiation Date Distribution
Count
200
100
0
Time of Treatment Initiation
2012Q1
2012Q2
2012Q3
2012Q4
2013Q1
2013Q2
2013Q3
2013Q4
2014Q1
2014Q2
2014Q3
2014Q4
2015Q1
2015Q2
2015Q3
2015Q4
2016Q1
2016Q2
2016Q3
2016Q4
2017Q1
2017Q2
2017Q3
2017Q4
2018Q1
2018Q2
2018Q3
2018Q4
2019Q1
2019Q2
2019Q3
2019Q4
Treatments
Pembrolizumab
Nivolumab
All immunotherapy drugs
Chemotherapy
Targeted therapy

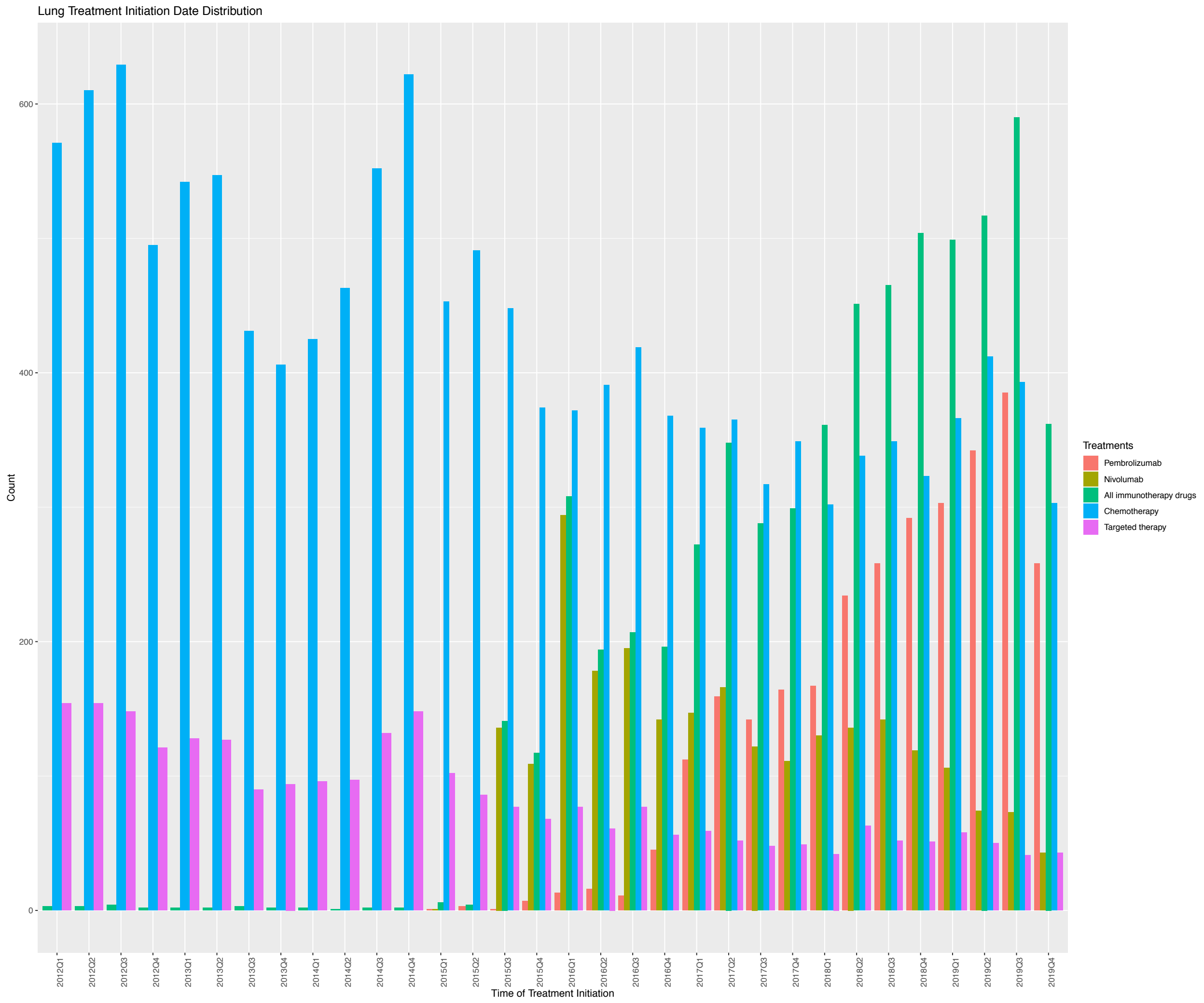

Lung Treatment Initiation Date Distribution
Count
0
200
400
600
Time of Treatment Initiation
2012Q1
2012Q2
2012Q3
2012Q4
2013Q1
2013Q2
2013Q3
2013Q4
2014Q1
2014Q2
2014Q3
2014Q4
2015Q1
2015Q2
2015Q3
2015Q4
2016Q1
2016Q2
2016Q3
2016Q4
2017Q1
2017Q2
2017Q3
2017Q4
2018Q1
2018Q2
2018Q3
2018Q4
2019Q1
2019Q2
2019Q3
2019Q4
Treatments
Pembrolizumab
Nivolumab
All immunotherapy drugs
Chemotherapy
Targeted therapy

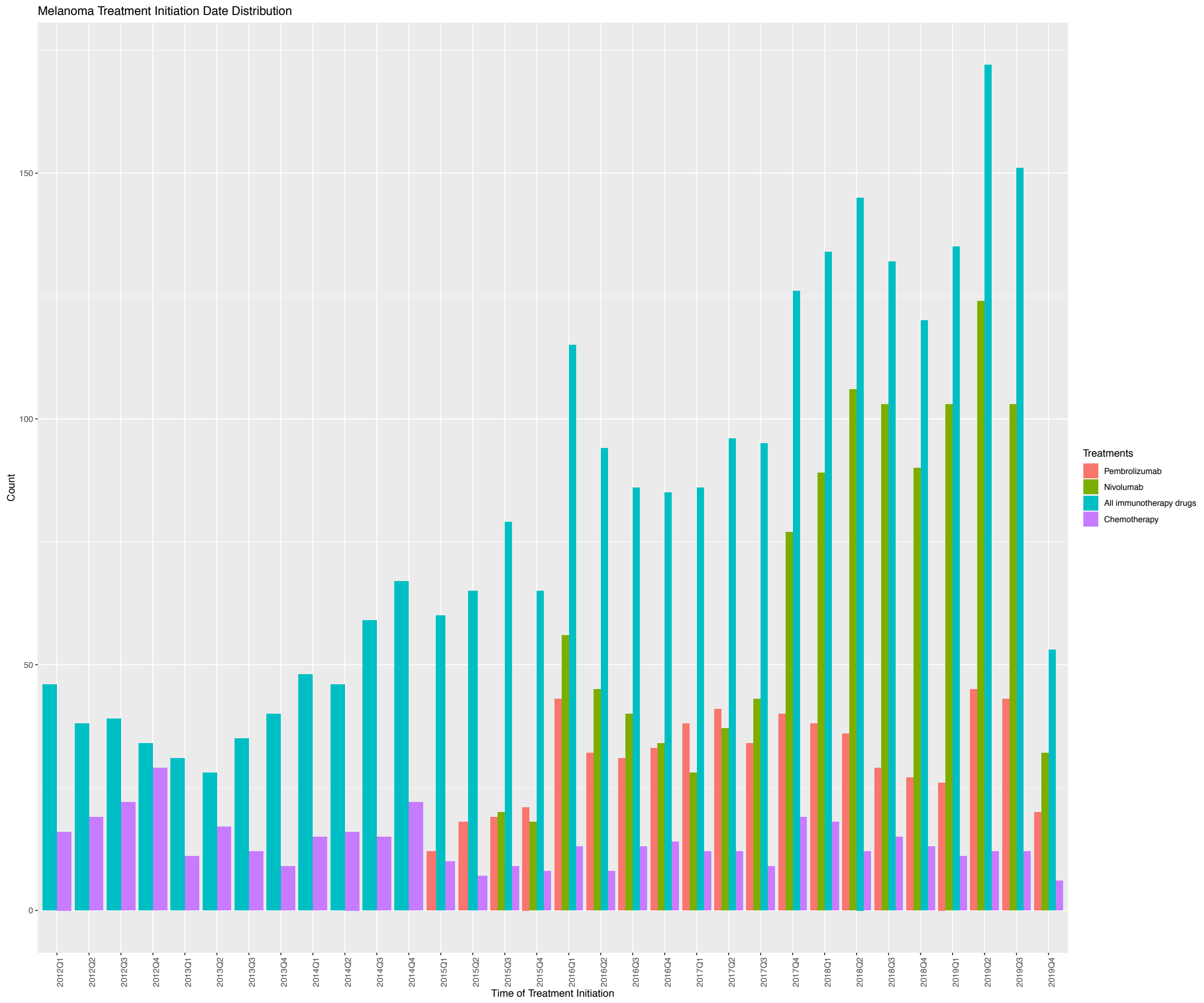

Melanoma Treatment Initiation Date Distribution
Count
0
50
100
150
Time of Treatment Initiation
2012Q1
2012Q2
2012Q3
2012Q4
2013Q1
2013Q2
2013Q3
2013Q4
2014Q1
2014Q2
2014Q3
2014Q4
2015Q1
2015Q2
2015Q3
2015Q4
2016Q1
2016Q2
2016Q3
2016Q4
2017Q1
2017Q2
2017Q3
2017Q4
2018Q1
2018Q2
2018Q3
2018Q4
2019Q1
2019Q2
2019Q3
2019Q4
Treatments
Pembrolizumab
Nivolumab
All immunotherapy drugs
Chemotherapy

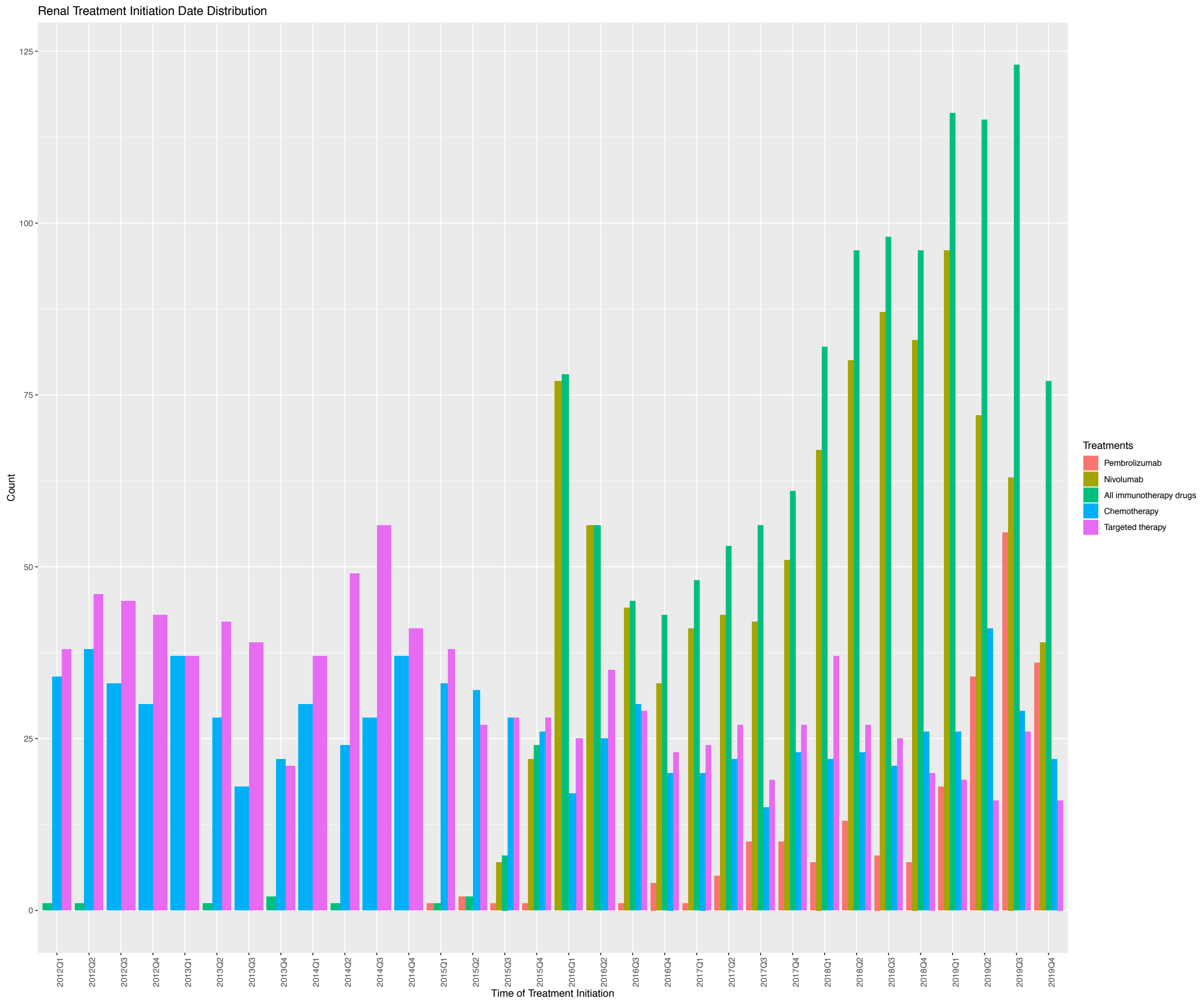

Renal Treatment Initiation Date Distribution
Count
Time of Treatment Initiation
Treatments
Pembrolizumab
Nivolumab
All immunotherapy drugs
Chemotherapy
Targeted therapy
0
25
50
75
100
125
2012Q1
2012Q2
2012Q3
2012Q4
2013Q1
2013Q2
2013Q3
2013Q4
2014Q1
2014Q2
2014Q3
2014Q4
2015Q1
2015Q2
2015Q3
2015Q4
2016Q1
2016Q2
2016Q3
2016Q4
2017Q1
2017Q2
2017Q3
2017Q4
2018Q1
2018Q2
2018Q3
2018Q4
2019Q1
2019Q2
2019Q3
2019Q4

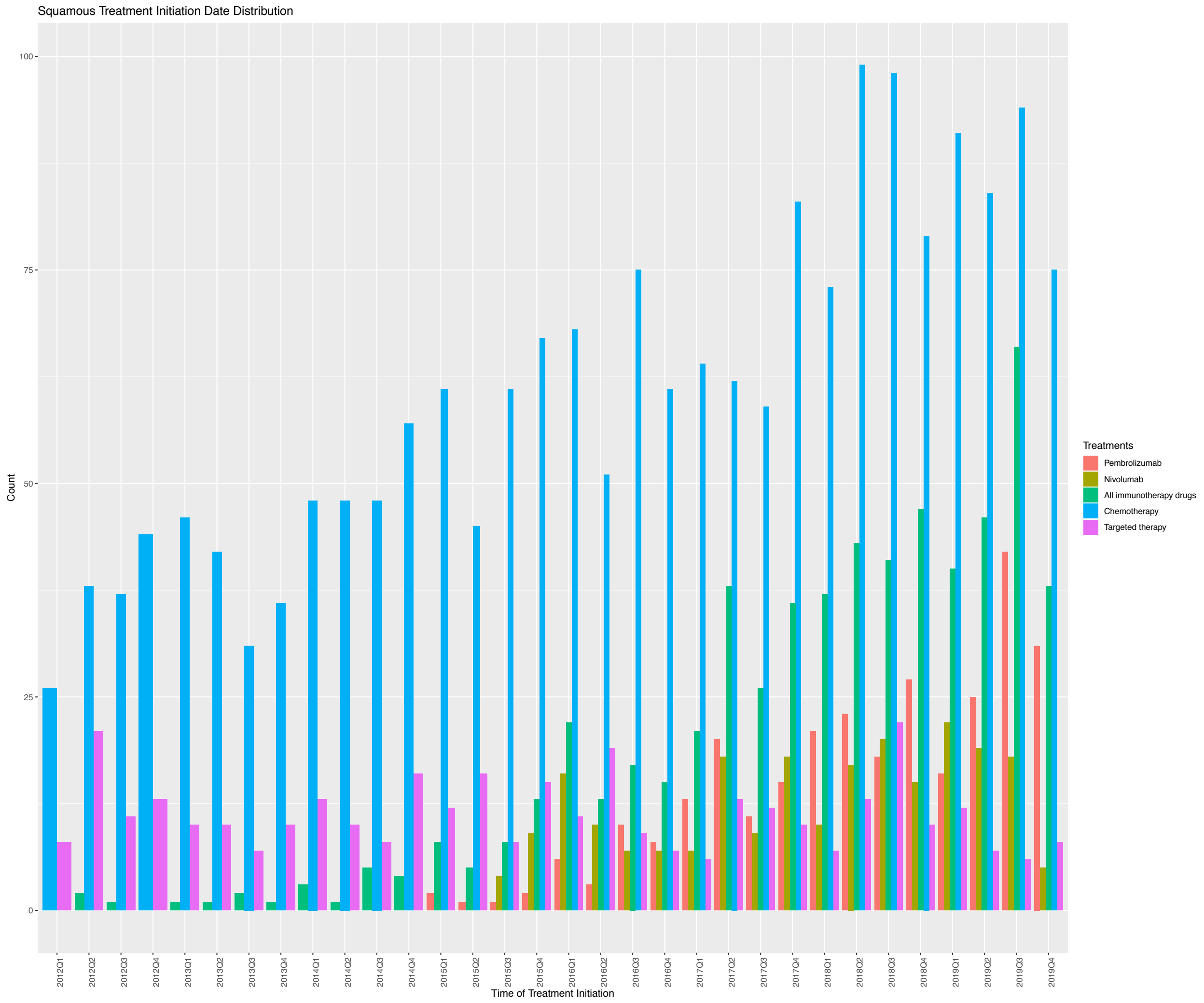

Squamous Treatment Initiation Date Distribution
Count
Time of Treatment Initiation
Treatments
Pembrolizumab
Nivolumab
All immunotherapy drugs
Chemotherapy
Targeted therapy
0
25
50
75
100
2012Q1
2012Q2
2012Q3
2012Q4
2013Q1
2013Q2
2013Q3
2013Q4
2014Q1
2014Q2
2014Q3
2014Q4
2015Q1
2015Q2
2015Q3
2015Q4
2016Q1
2016Q2
2016Q3
2016Q4
2017Q1
2017Q2
2017Q3
2017Q4
2018Q1
2018Q2
2018Q3
2018Q4
2019Q1
2019Q2
2019Q3
2019Q4

Figure S2. (Extending Figure 4 in the main text) Time-to-event plot showing the time between treatment initiation and the development of specific autoimmune diseases within 15 months.

Chemotherapy

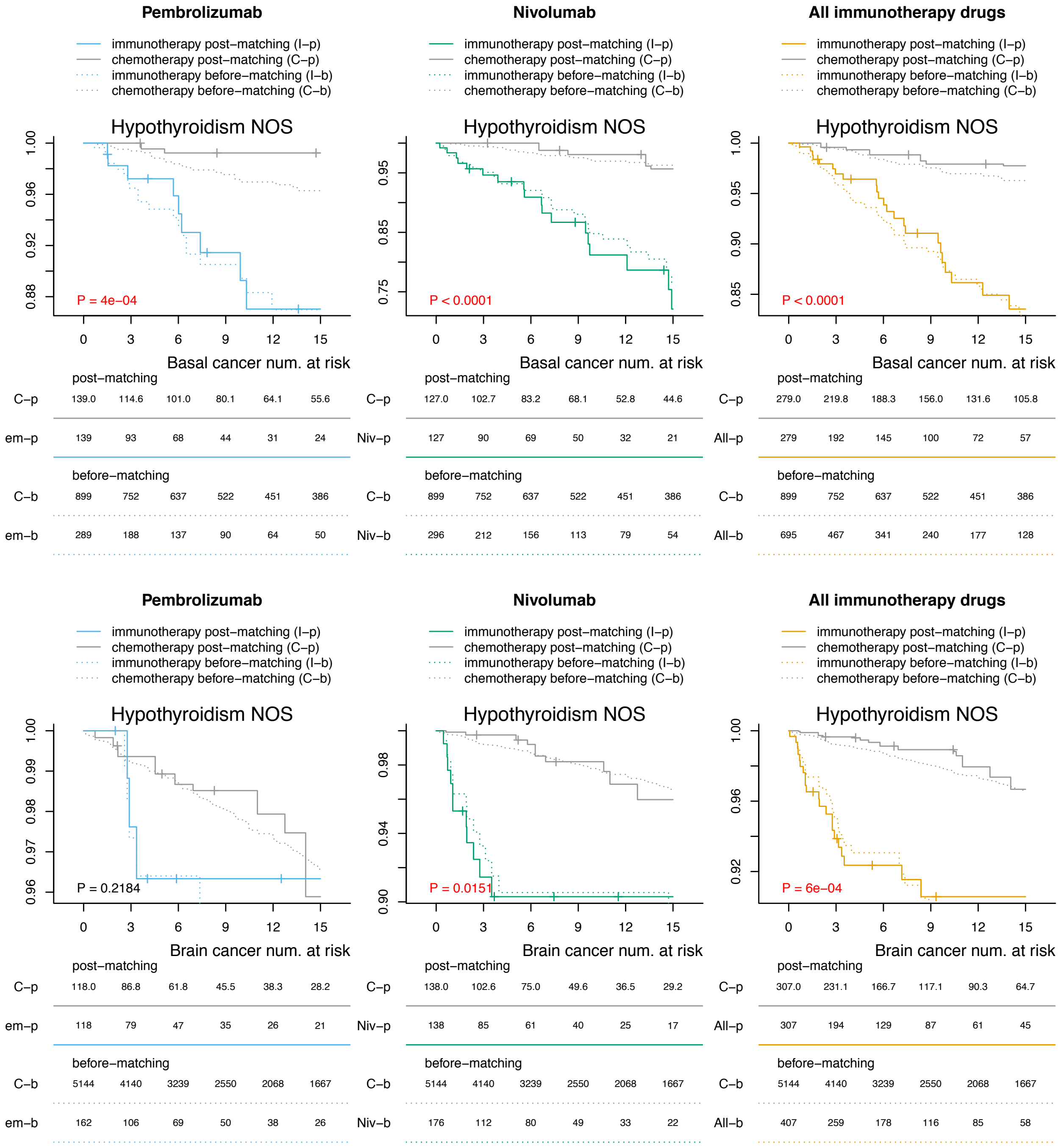

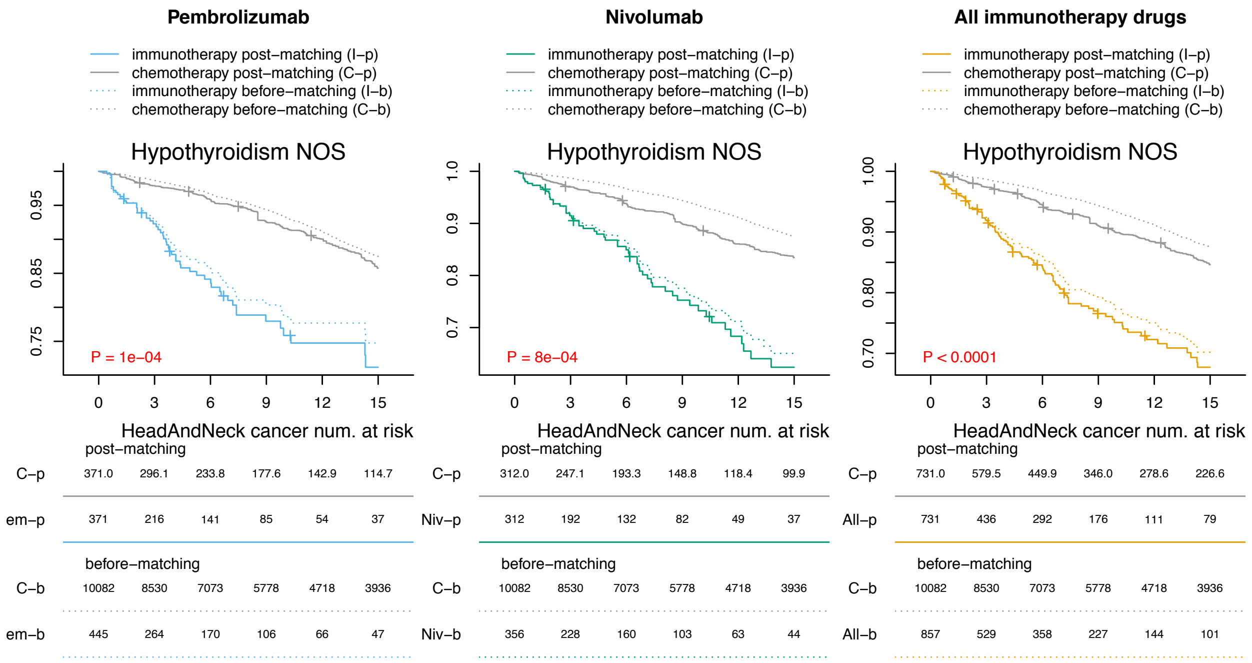
Pembrolizumab
immunotherapy post−matching (I−p)
chemotherapy post−matching (C−p)
immunotherapy before−matching (I−b)
chemotherapy before−matching (C−b)
Hypothyroidism NOS
0.95
0.85
0.75
P = 1e−04
0 3 6 9 12 15
HeadAndNeck cancer num. at risk
post−matching
C−p 371.0 296.1 233.8 177.6 142.9 114.7
em−p 371 216 141 85 54 37
before−matching
C−b 10082 8530 7073 5778 4718 3936
em−b 445 264 170 106 66 47
Nivolumab
immunotherapy post−matching (I−p)
chemotherapy post−matching (C−p)
immunotherapy before−matching (I−b)
chemotherapy before−matching (C−b)
Hypothyroidism NOS
1.0
0.9
0.8
0.7
P = 8e−04
0 3 6 9 12 15
HeadAndNeck cancer num. at risk
post−matching
C−p 312.0 247.1 193.3 148.8 118.4 99.9
Niv−p 312 192 132 82 49 37
before−matching
C−b 10082 8530 7073 5778 4718 3936
Niv−b 356 228 160 103 63 44
All immunotherapy drugs
immunotherapy post−matching (I−p)
chemotherapy post−matching (C−p)
immunotherapy before−matching (I−b)
chemotherapy before−matching (C−b)
Hypothyroidism NOS
1.00
0.90
0.80
0.70
P < 0.0001
0 3 6 9 12 15
HeadAndNeck cancer num. at risk
post−matching
C−p 731.0 579.5 449.9 346.0 278.6 226.6
All−p 731 436 292 176 111 79
before−matching
C−b 10082 8530 7073 5778 4718 3936
All−b 857 529 358 227 144 101

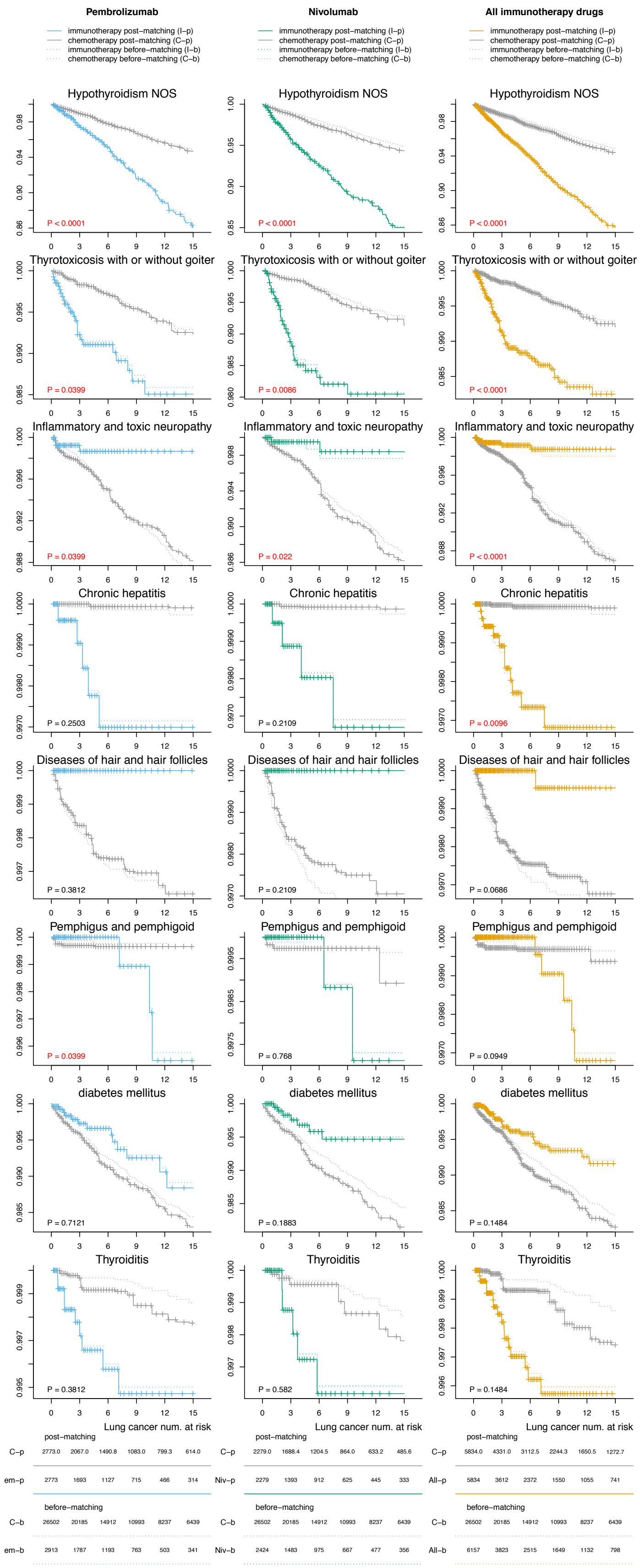

Pembrolizumab
Nivolumab
All immunotherapy drugs
immunotherapy post−matching (I−p)
chemotherapy post−matching (C−p)
immunotherapy before−matching (I−b)
chemotherapy before−matching (C−b)
Hypothyroidism NOS
P < 0.0001
Thyrotoxicosis with or without goiter
P = 0.0399
P = 0.0086
P < 0.0001
Inflammatory and toxic neuropathy
P = 0.0399
P = 0.022
P < 0.0001
Chronic hepatitis
P = 0.2503
P = 0.2109
P = 0.0096
Diseases of hair and hair follicles
P = 0.3812
P = 0.2109
P = 0.0686
Pemphigus and pemphigoid
P = 0.0399
P = 0.768
P = 0.0949
diabetes mellitus
P = 0.7121
P = 0.1883
P = 0.1484
Thyroiditis
P = 0.3812
P = 0.582
P = 0.1484
Lung cancer num. at risk
post−matching
C−p 2773.0 2067.0 1490.8 1083.0 799.3 614.0
em−p 2773 1693 1127 715 466 314
C−p 2279.0 1688.4 1204.5 864.0 633.2 485.6
Niv−p 2279 1393 912 625 445 333
C−p 5834.0 4331.0 3112.5 2244.3 1650.5 1272.7
All−p 5834 3612 2372 1550 1055 741
before−matching
C−b 26502 20185 14912 10993 8237 6439
em−b 2913 1787 1193 763 503 341
C−b 26502 20185 14912 10993 8237 6439
Niv−b 2424 1483 975 667 477 356
C−b 26502 20185 14912 10993 8237 6439
All−b 6157 3823 2515 1649 1132 798

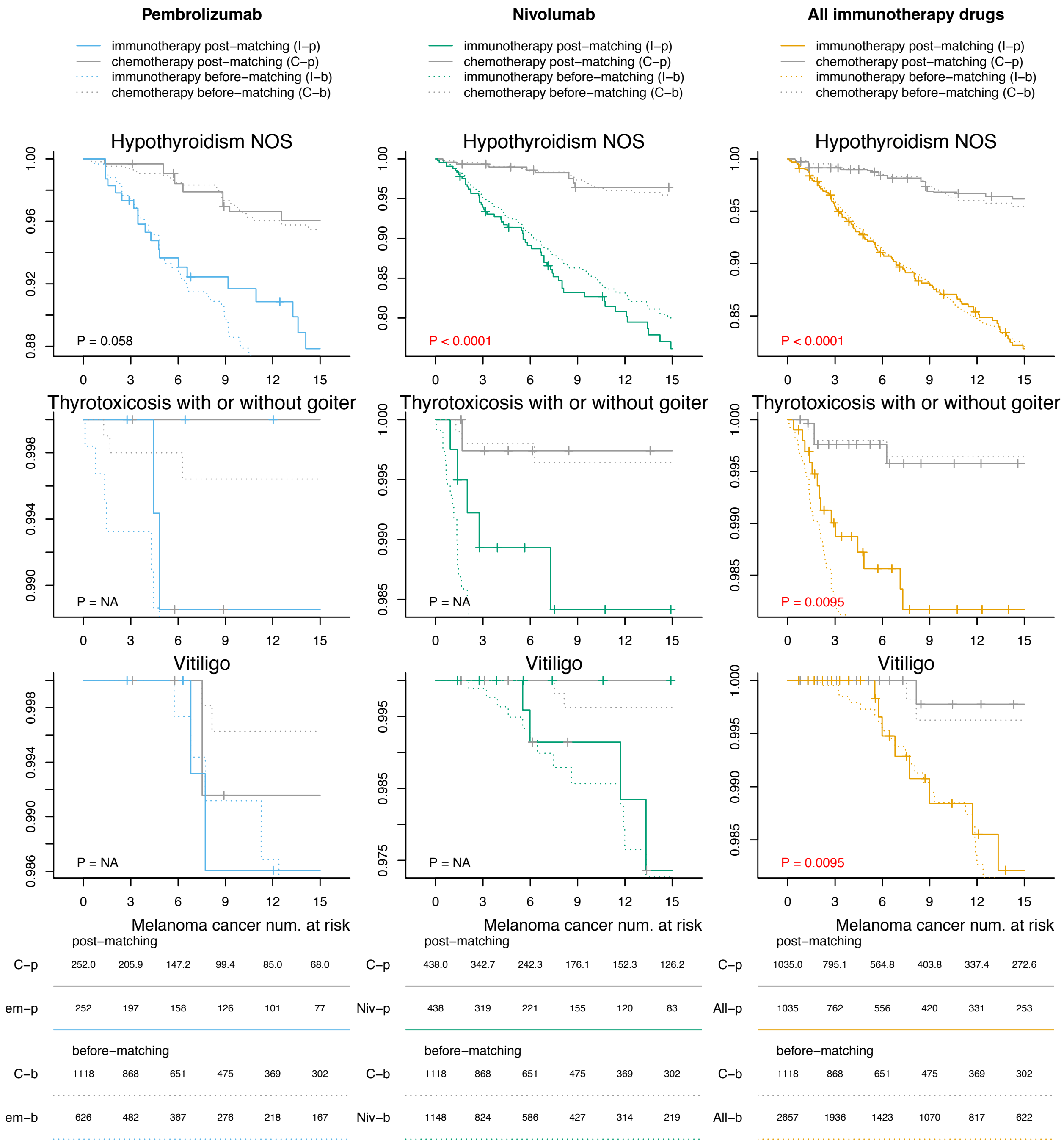
Pembrolizumab
Nivolumab
All immunotherapy drugs
immunotherapy post−matching (I−p)
chemotherapy post−matching (C−p)
immunotherapy before−matching (I−b)
chemotherapy before−matching (C−b)
Hypothyroidism NOS
P = 0.058
P < 0.0001
P < 0.0001
Thyrotoxicosis with or without goiter
P = NA
P = NA
P = 0.0095
Vitiligo
P = NA
P = NA
P = 0.0095
Melanoma cancer num. at risk
post−matching
C−p 252.0 205.9 147.2 99.4 85.0 68.0
em−p 252 197 158 126 101 77
before−matching
C−b 1118 868 651 475 369 302
em−b 626 482 367 276 218 167
C−p 438.0 342.7 242.3 176.1 152.3 126.2
Niv−p 438 319 221 155 120 83
C−b 1118 868 651 475 369 302
Niv−b 1148 824 586 427 314 219
C−p 1035.0 795.1 564.8 403.8 337.4 272.6
All−p 1035 762 556 420 331 253
C−b 1118 868 651 475 369 302
All−b 2657 1936 1423 1070 817 622

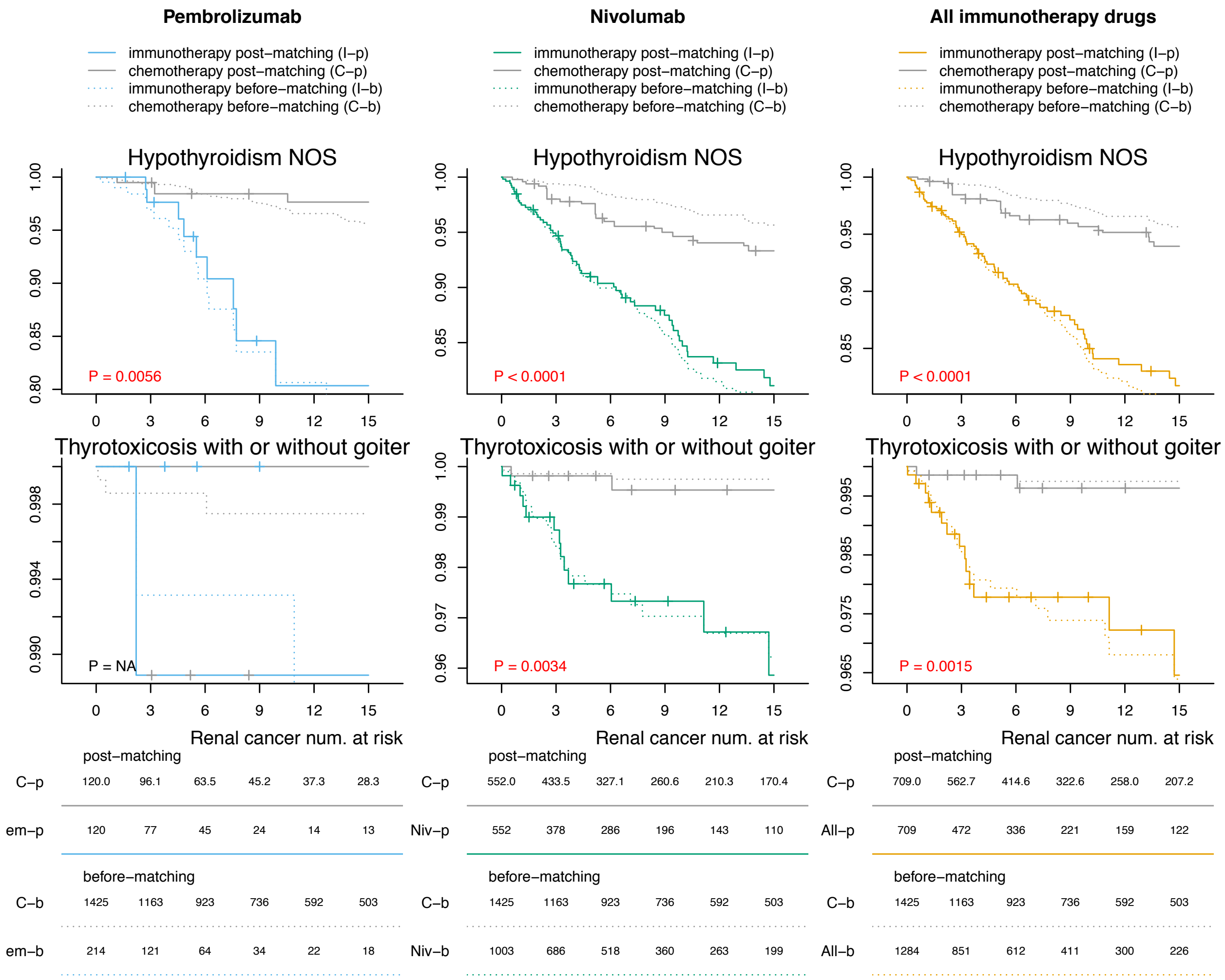

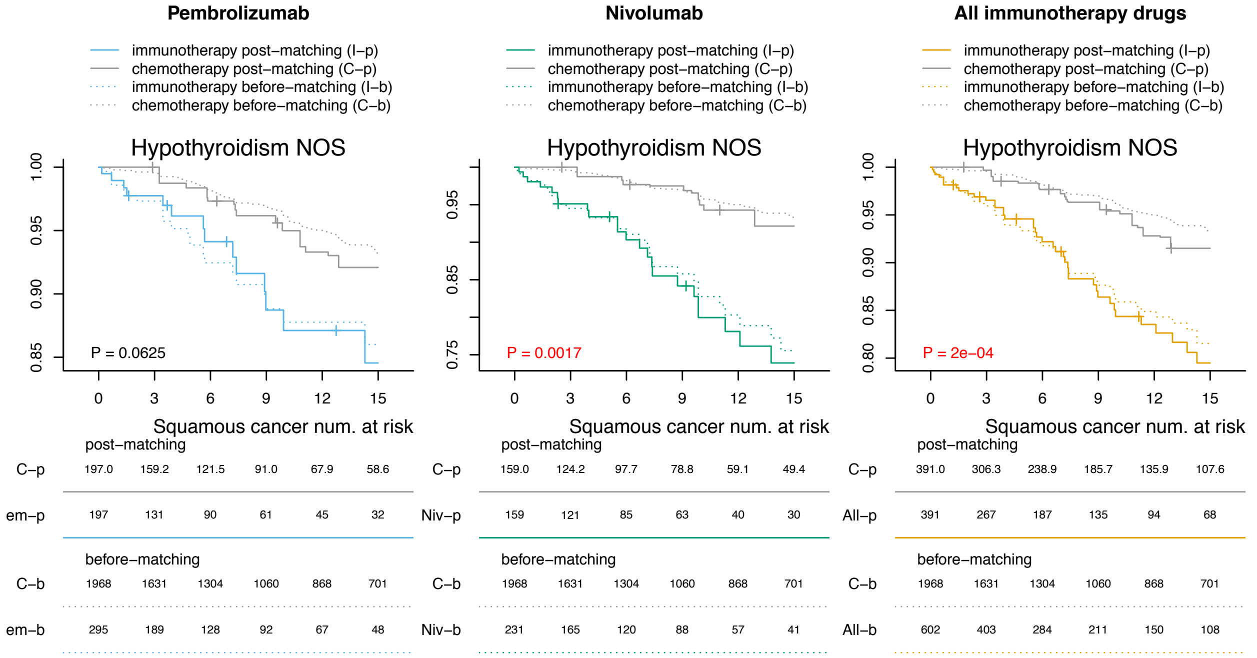

Targeted therapy

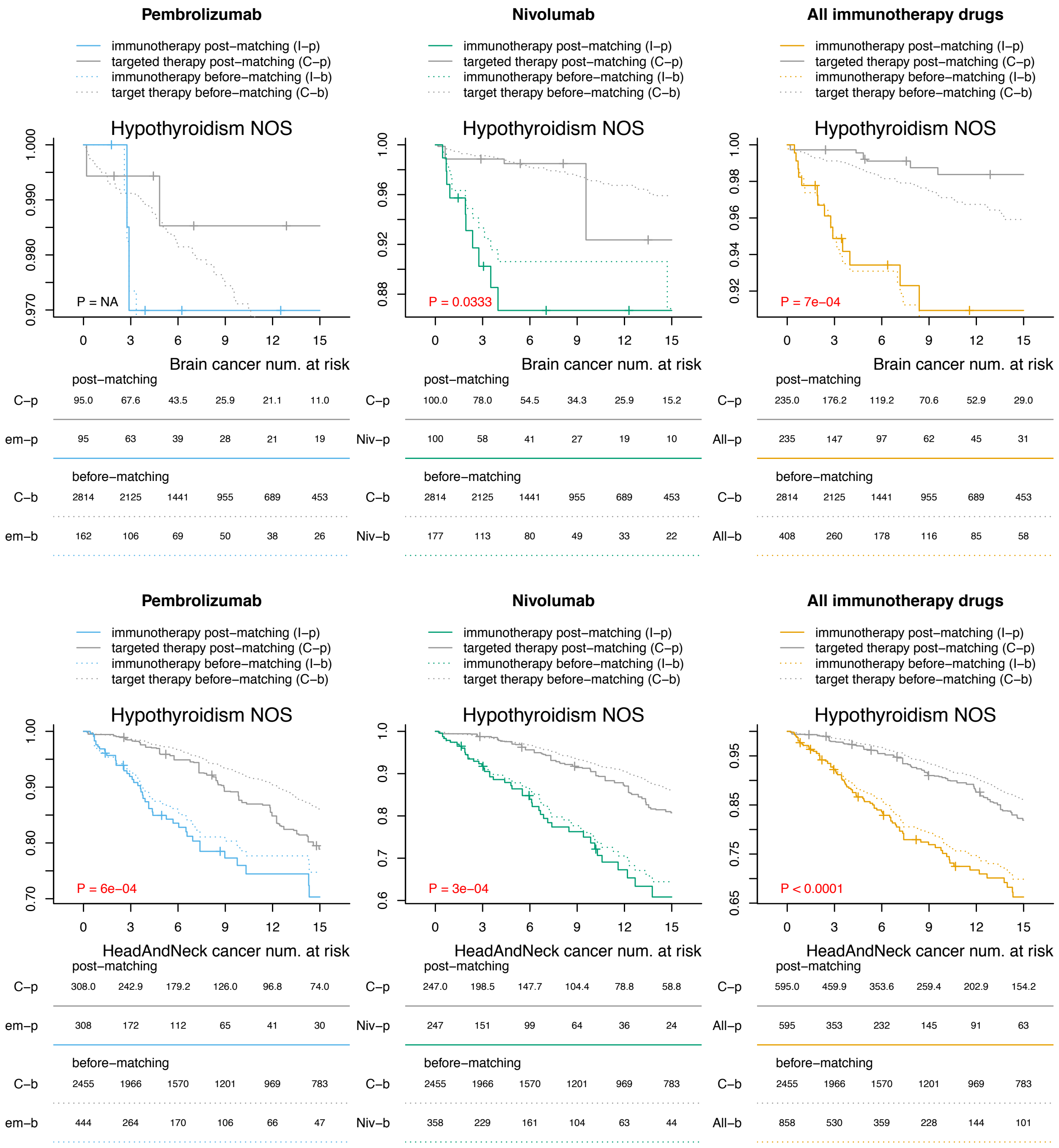
Pembrolizumab
immunotherapy post−matching (I−p)
targeted therapy post−matching (C−p)
immunotherapy before−matching (I−b)
target therapy before−matching (C−b)
Hypothyroidism NOS
1.000
0.990
0.980
0.970
P = NA
0 3 6 9 12 15
Brain cancer num. at risk
post−matching
C−p 95.0 67.6 43.5 25.9 21.1 11.0
em−p 95 63 39 28 21 19
before−matching
C−b 2814 2125 1441 955 689 453
em−b 162 106 69 50 38 26
Nivolumab
immunotherapy post−matching (I−p)
targeted therapy post−matching (C−p)
immunotherapy before−matching (I−b)
target therapy before−matching (C−b)
Hypothyroidism NOS
1.00
0.96
0.92
0.88
P = 0.0333
0 3 6 9 12 15
Brain cancer num. at risk
post−matching
C−p 100.0 78.0 54.5 34.3 25.9 15.2
Niv−p 100 58 41 27 19 10
before−matching
C−b 2814 2125 1441 955 689 453
Niv−b 177 113 80 49 33 22
All immunotherapy drugs
immunotherapy post−matching (I−p)
targeted therapy post−matching (C−p)
immunotherapy before−matching (I−b)
target therapy before−matching (C−b)
Hypothyroidism NOS
1.00
0.98
0.96
0.94
0.92
P = 7e−04
0 3 6 9 12 15
Brain cancer num. at risk
post−matching
C−p 235.0 176.2 119.2 70.6 52.9 29.0
All−p 235 147 97 62 45 31
before−matching
C−b 2814 2125 1441 955 689 453
All−b 408 260 178 116 85 58
Pembrolizumab
immunotherapy post−matching (I−p)
targeted therapy post−matching (C−p)
immunotherapy before−matching (I−b)
target therapy before−matching (C−b)
Hypothyroidism NOS
1.00
0.90
0.80
0.70
P = 6e−04
0 3 6 9 12 15
HeadAndNeck cancer num. at risk
post−matching
C−p 308.0 242.9 179.2 126.0 96.8 74.0
em−p 308 172 112 65 41 30
before−matching
C−b 2455 1966 1570 1201 969 783
em−b 444 264 170 106 66 47
Nivolumab
immunotherapy post−matching (I−p)
targeted therapy post−matching (C−p)
immunotherapy before−matching (I−b)
target therapy before−matching (C−b)
Hypothyroidism NOS
1.0
0.9
0.8
0.7
0.6
P = 3e−04
0 3 6 9 12 15
HeadAndNeck cancer num. at risk
post−matching
C−p 247.0 198.5 147.7 104.4 78.8 58.8
Niv−p 247 151 99 64 36 24
before−matching
C−b 2455 1966 1570 1201 969 783
Niv−b 358 229 161 104 63 44
All immunotherapy drugs
immunotherapy post−matching (I−p)
targeted therapy post−matching (C−p)
immunotherapy before−matching (I−b)
target therapy before−matching (C−b)
Hypothyroidism NOS
0.95
0.85
0.75
0.65
P < 0.0001
0 3 6 9 12 15
HeadAndNeck cancer num. at risk
post−matching
C−p 595.0 459.9 353.6 259.4 202.9 154.2
All−p 595 353 232 145 91 63
before−matching
C−b 2455 1966 1570 1201 969 783
All−b 858 530 359 228 144 101

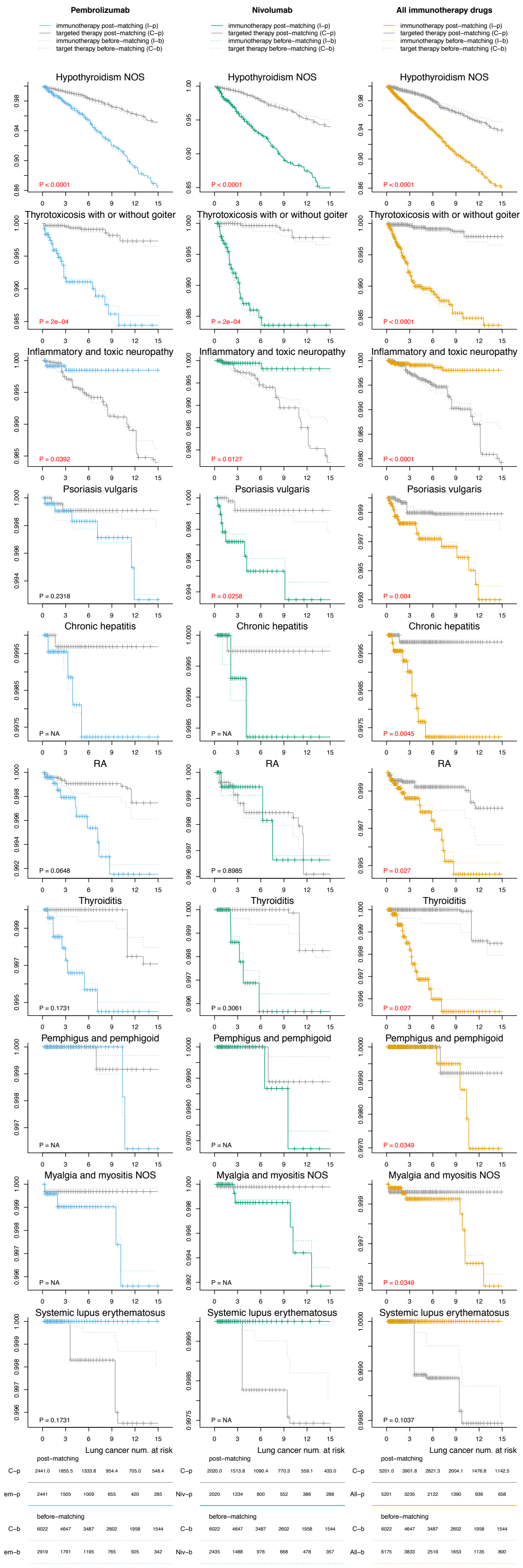

Pembrolizumab
Nivolumab
All immunotherapy drugs
immunotherapy post−matching (I−p)
targeted therapy post−matching (C−p)
immunotherapy before−matching (I−b)
target therapy before−matching (C−b)
Hypothyroidism NOS
P < 0.0001
Thyrotoxicosis with or without goiter
P = 2e−04
P = 2e−04
P < 0.0001
Inflammatory and toxic neuropathy
P = 0.0392
P = 0.0127
P < 0.0001
Psoriasis vulgaris
P = 0.2318
P = 0.0258
P = 0.004
Chronic hepatitis
P = NA
P = NA
P = 0.0045
RA
P = 0.0648
P = 0.8985
P = 0.027
Thyroiditis
P = 0.1731
P = 0.3061
P = 0.027
Pemphigus and pemphigoid
P = NA
P = NA
P = 0.0349
Myalgia and myositis NOS
P = NA
P = NA
P = 0.0349
Systemic lupus erythematosus
P = 0.1731
P = NA
P = 0.1037
Lung cancer num. at risk
post−matching
C−p 2441.0 1855.5 1333.8 954.4 705.0 548.4
em−p 2441 1505 1009 655 420 285
before−matching
C−b 6022 4647 3487 2602 1958 1544
em−b 2919 1791 1195 765 505 342
C−p 2020.0 1513.8 1090.4 770.3 559.1 433.0
Niv−p 2020 1234 800 552 386 288
C−b 6022 4647 3487 2602 1958 1544
Niv−b 2435 1488 976 668 478 357
C−p 5201.0 3901.8 2821.3 2004.1 1476.8 1142.5
All−p 5201 3235 2122 1390 936 658
C−b 6022 4647 3487 2602 1958 1544
All−b 6175 3833 2519 1653 1135 800

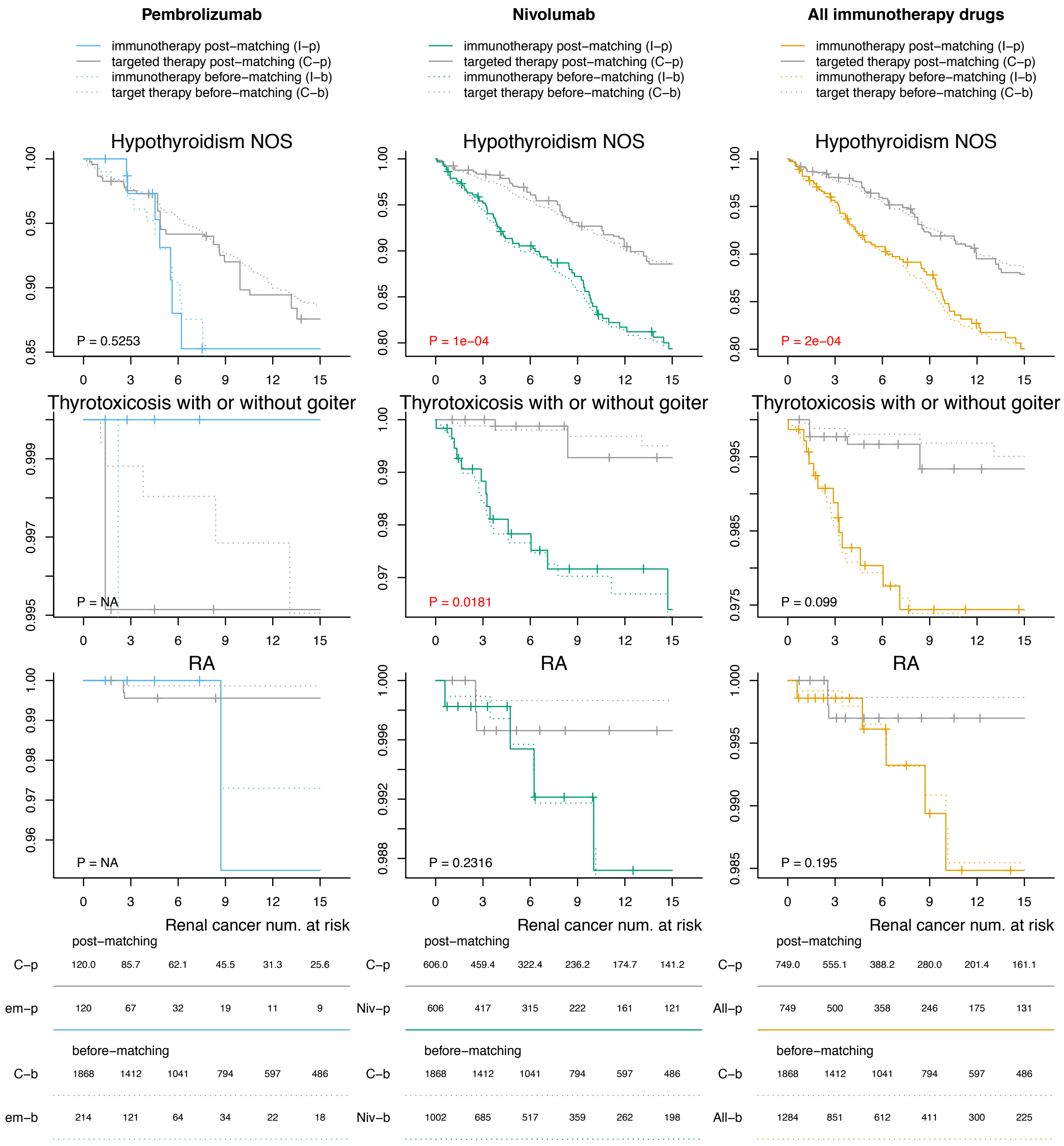
Pembrolizumab
Nivolumab
All immunotherapy drugs
immunotherapy post−matching (I−p)
targeted therapy post−matching (C−p)
immunotherapy before−matching (I−b)
target therapy before−matching (C−b)
Hypothyroidism NOS
P = 0.5253
P = 1e−04
P = 2e−04
Thyrotoxicosis with or without goiter
P = NA
P = 0.0181
P = 0.099
RA
P = NA
P = 0.2316
P = 0.195
Renal cancer num. at risk
post−matching
C−p 120.0 85.7 62.1 45.5 31.3 25.6
em−p 120 67 32 19 11 9
C−p 606.0 459.4 322.4 236.2 174.7 141.2
Niv−p 606 417 315 222 161 121
C−p 749.0 555.1 388.2 280.0 201.4 161.1
All−p 749 500 358 246 175 131
before−matching
C−b 1868 1412 1041 794 597 486
em−b 214 121 64 34 22 18
Niv−b 1002 685 517 359 262 198
All−b 1284 851 612 411 300 225

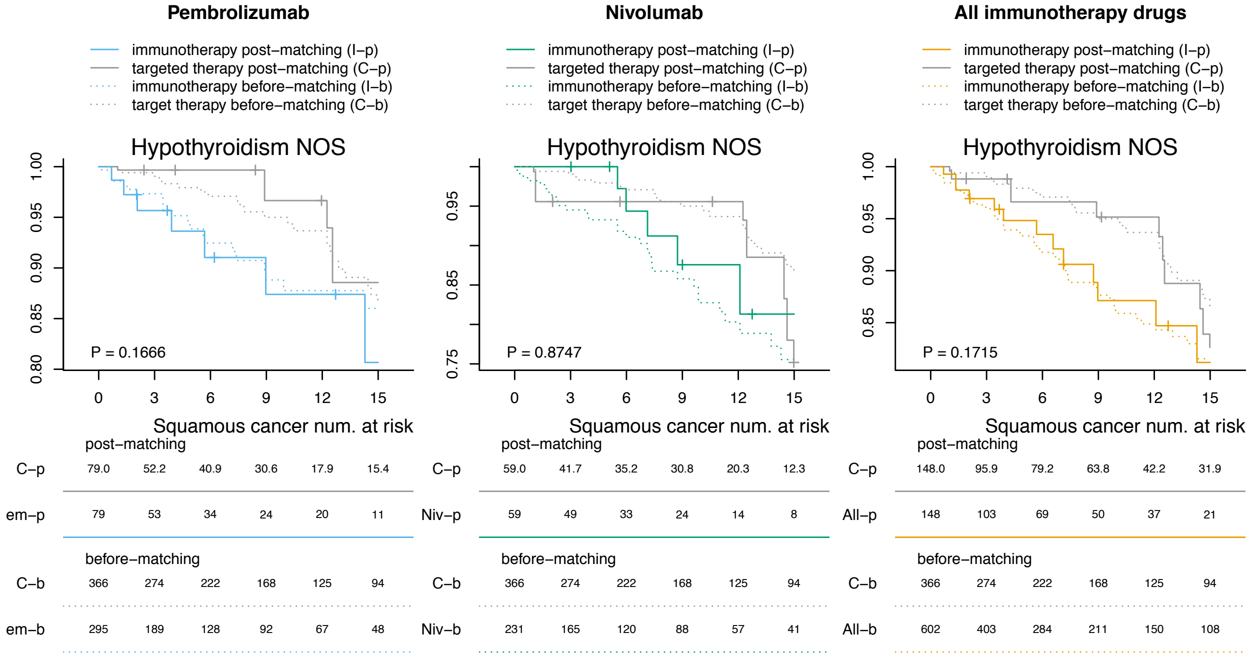
Pembrolizumab
immunotherapy post−matching (I−p)
targeted therapy post−matching (C−p)
immunotherapy before−matching (I−b)
target therapy before−matching (C−b)
Hypothyroidism NOS
1.00
0.95
0.90
0.85
0.80
P = 0.1666
0 3 6 9 12 15
Squamous cancer num. at risk
post−matching
C−p 79.0 52.2 40.9 30.6 17.9 15.4
em−p 79 53 34 24 20 11
before−matching
C−b 366 274 222 168 125 94
em−b 295 189 128 92 67 48
Nivolumab
immunotherapy post−matching (I−p)
targeted therapy post−matching (C−p)
immunotherapy before−matching (I−b)
target therapy before−matching (C−b)
Hypothyroidism NOS
0.95
0.85
0.75
P = 0.8747
0 3 6 9 12 15
Squamous cancer num. at risk
post−matching
C−p 59.0 41.7 35.2 30.8 20.3 12.3
Niv−p 59 49 33 24 14 8
before−matching
C−b 366 274 222 168 125 94
Niv−b 231 165 120 88 57 41
All immunotherapy drugs
immunotherapy post−matching (I−p)
targeted therapy post−matching (C−p)
immunotherapy before−matching (I−b)
target therapy before−matching (C−b)
Hypothyroidism NOS
1.00
0.95
0.90
0.85
P = 0.1715
0 3 6 9 12 15
Squamous cancer num. at risk
post−matching
C−p 148.0 95.9 79.2 63.8 42.2 31.9
All−p 148 103 69 50 37 21
before−matching
C−b 366 274 222 168 125 94
All−b 602 403 284 211 150 108

Figure S3. Covariate balance before and after matching. We achieved near perfect balance on gender, age, average income, the annual frequency of hospital visits prior to treatment initialization, and the annual frequency of ICD code counts prior to treatment initialization. Some ethnicity groups have extreme values because they are rare. Since we allowed patients with unknown ethnicity to match with participants in any ethnicity group, the results of covariate balance on ethnicity varied.

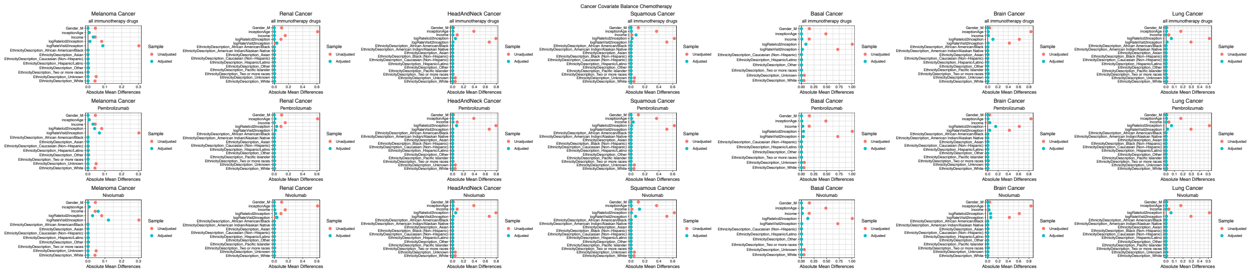

Figure S4. Time-to-event plot showing the time between treatment initiation and the development of specific autoimmune diseases within 5 years.

Chemotherapy

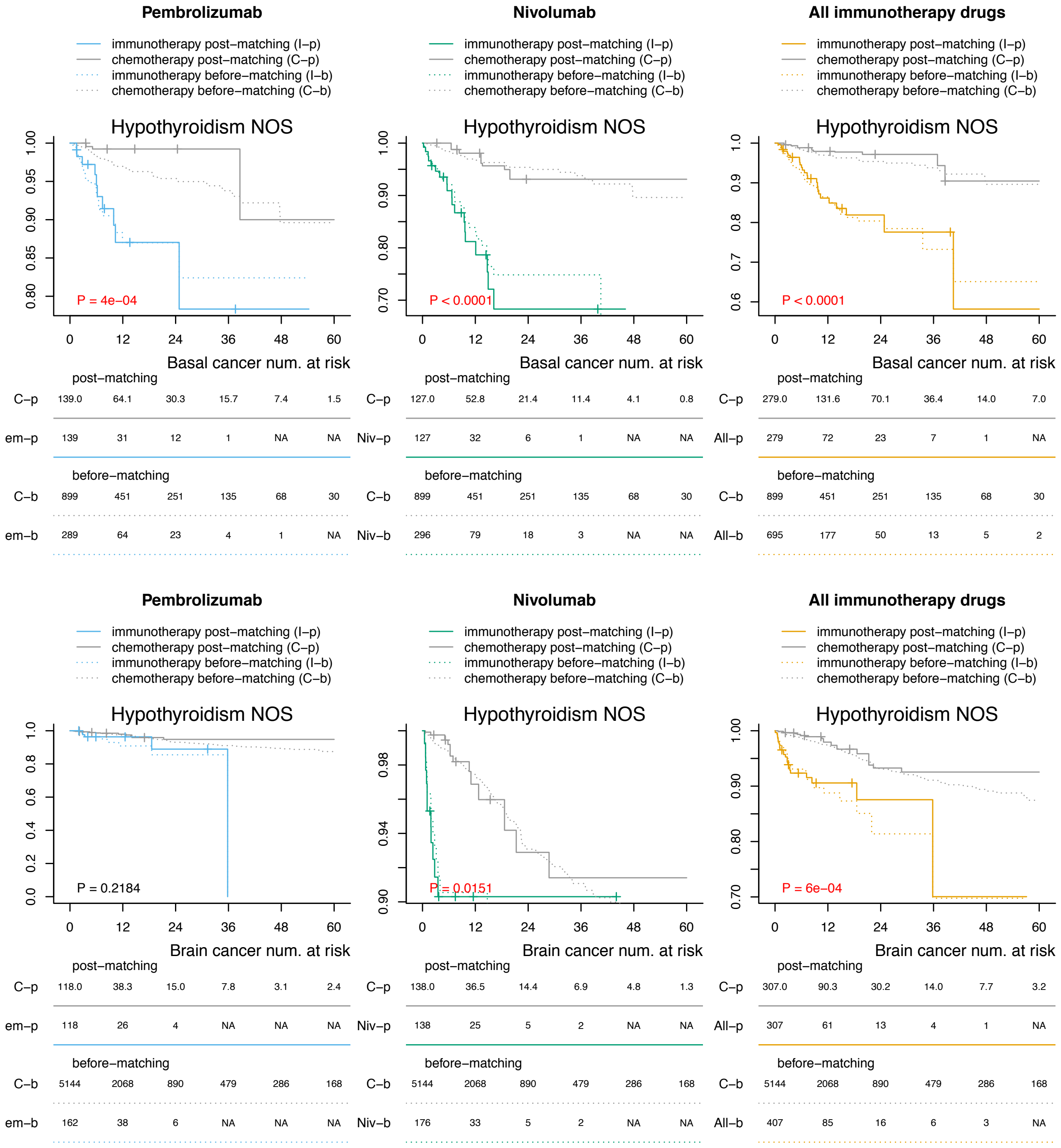

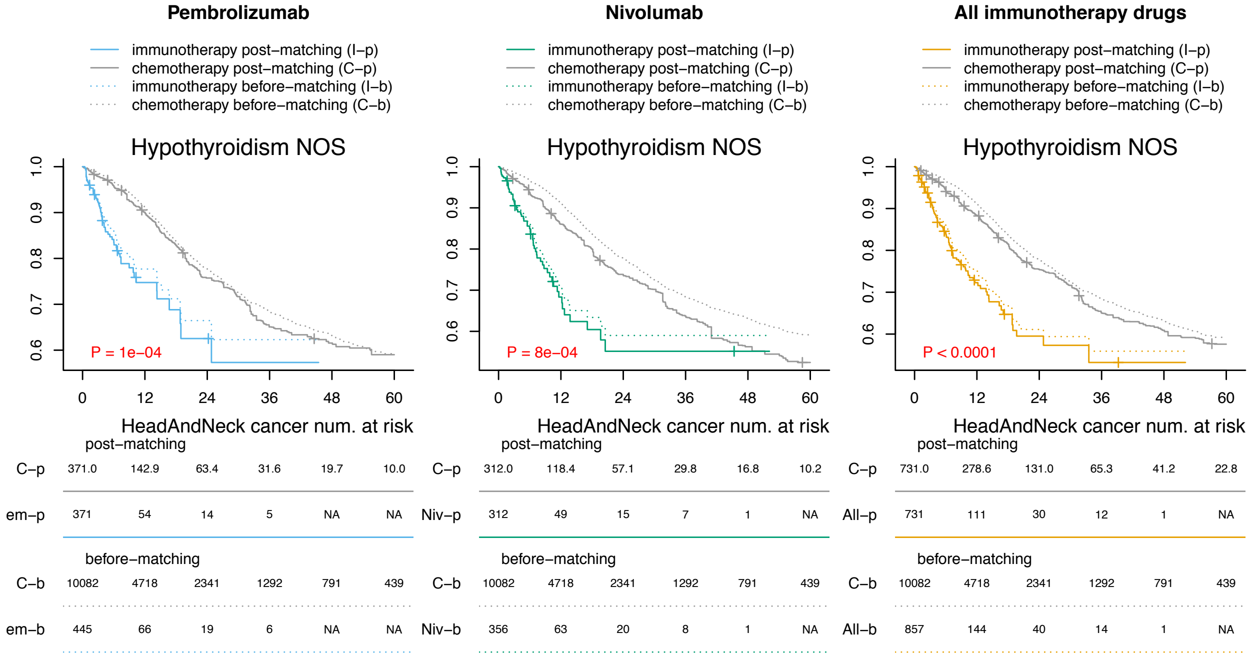
Pembrolizumab
immunotherapy post−matching (I−p)
chemotherapy post−matching (C−p)
immunotherapy before−matching (I−b)
chemotherapy before−matching (C−b)
Hypothyroidism NOS
P = 1e−04
HeadAndNeck cancer num. at risk
post−matching
C−p 371.0 142.9 63.4 31.6 19.7 10.0
em−p 371 54 14 5 NA NA
before−matching
C−b 10082 4718 2341 1292 791 439
em−b 445 66 19 6 NA NA
Nivolumab
immunotherapy post−matching (I−p)
chemotherapy post−matching (C−p)
immunotherapy before−matching (I−b)
chemotherapy before−matching (C−b)
Hypothyroidism NOS
P = 8e−04
HeadAndNeck cancer num. at risk
post−matching
C−p 312.0 118.4 57.1 29.8 16.8 10.2
Niv−p 312 49 15 7 1 NA
before−matching
C−b 10082 4718 2341 1292 791 439
Niv−b 356 63 20 8 1 NA
All immunotherapy drugs
immunotherapy post−matching (I−p)
chemotherapy post−matching (C−p)
immunotherapy before−matching (I−b)
chemotherapy before−matching (C−b)
Hypothyroidism NOS
P < 0.0001
HeadAndNeck cancer num. at risk
post−matching
C−p 731.0 278.6 131.0 65.3 41.2 22.8
All−p 731 111 30 12 1 NA
before−matching
C−b 10082 4718 2341 1292 791 439
All−b 857 144 40 14 1 NA

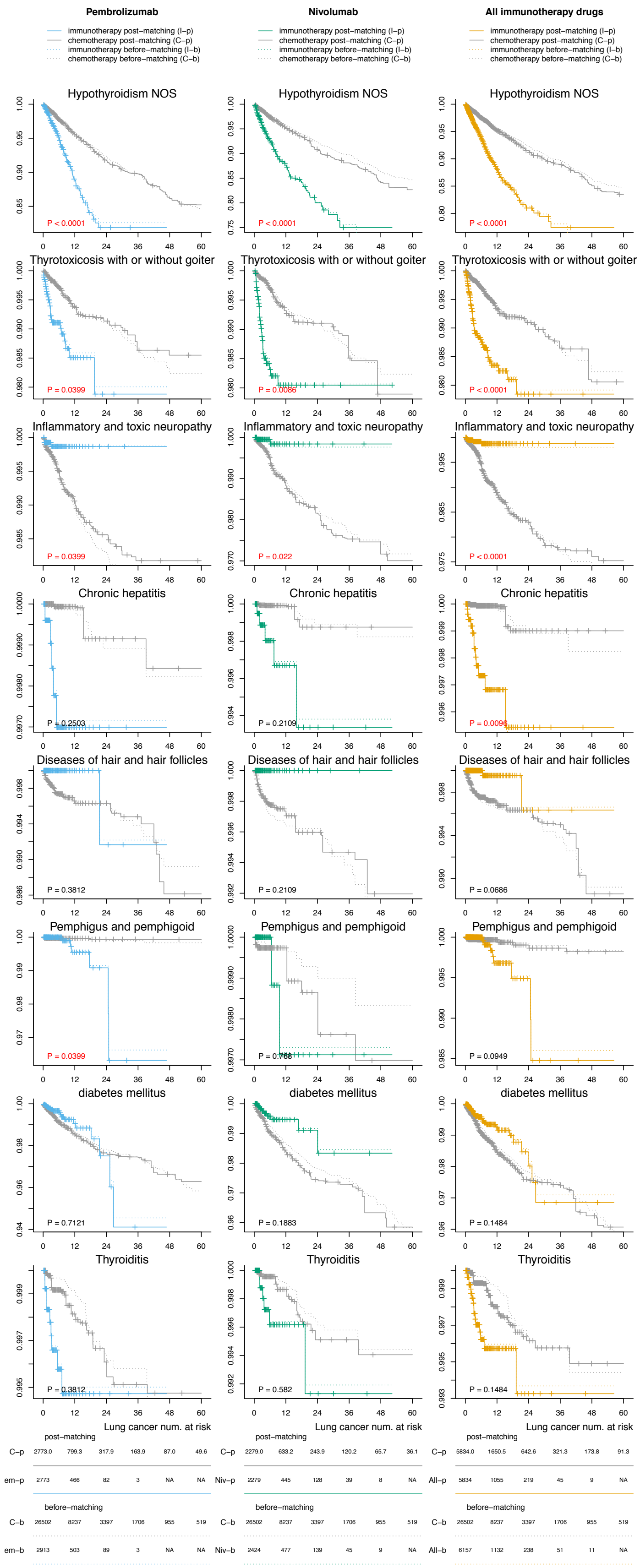
Pembrolizumab
Nivolumab
All immunotherapy drugs
immunotherapy post−matching (I−p)
chemotherapy post−matching (C−p)
immunotherapy before−matching (I−b)
chemotherapy before−matching (C−b)
Hypothyroidism NOS
P < 0.0001
Thyrotoxicosis with or without goiter
P = 0.0399
P = 0.0086
P < 0.0001
Inflammatory and toxic neuropathy
P = 0.0399
P = 0.022
P < 0.0001
Chronic hepatitis
P = 0.2503
P = 0.2109
P = 0.0096
Diseases of hair and hair follicles
P = 0.3812
P = 0.2109
P = 0.0686
Pemphigus and pemphigoid
P = 0.0399
P = 0.768
P = 0.0949
diabetes mellitus
P = 0.7121
P = 0.1883
P = 0.1484
Thyroiditis
P = 0.3812
P = 0.582
P = 0.1484
Lung cancer num. at risk
post−matching
C−p 2773.0 799.3 317.9 163.9 87.0 49.6
em−p 2773 466 82 3 NA NA
before−matching
C−b 26502 8237 3397 1706 955 519
em−b 2913 503 89 3 NA NA
C−p 2279.0 633.2 243.9 120.2 65.7 36.1
Niv−p 2279 445 128 39 8 NA
C−b 26502 8237 3397 1706 955 519
Niv−b 2424 477 139 45 9 NA
C−p 5834.0 1650.5 642.6 321.3 173.8 91.3
All−p 5834 1055 219 45 9 NA
C−b 26502 8237 3397 1706 955 519
All−b 6157 1132 238 51 11 NA

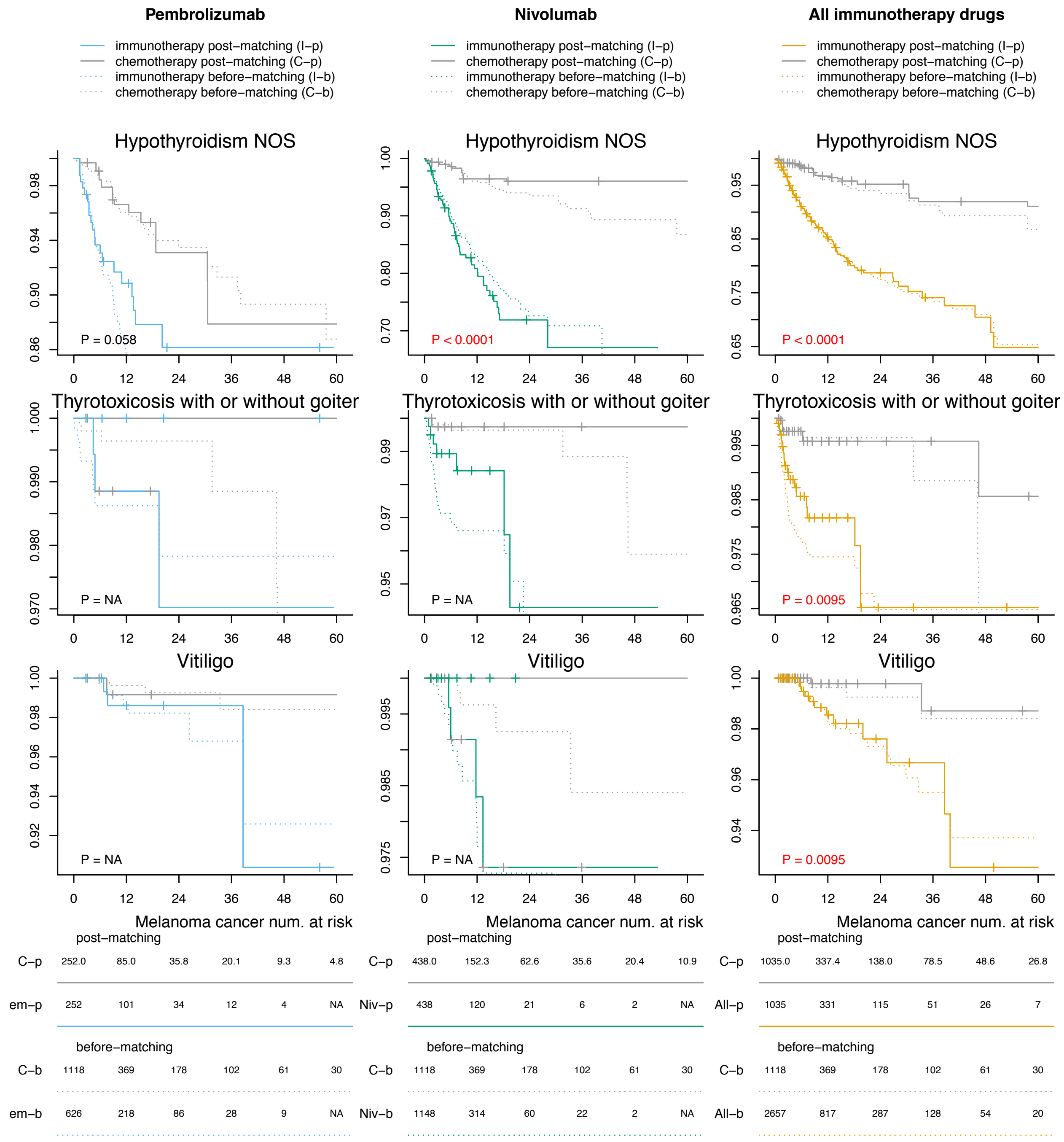
Pembrolizumab
Nivolumab
All immunotherapy drugs
immunotherapy post−matching (I−p)
chemotherapy post−matching (C−p)
immunotherapy before−matching (I−b)
chemotherapy before−matching (C−b)
Hypothyroidism NOS
P = 0.058
P < 0.0001
P < 0.0001
Thyrotoxicosis with or without goiter
P = NA
P = NA
P = 0.0095
Vitiligo
P = NA
P = NA
P = 0.0095
Melanoma cancer num. at risk
post−matching
C−p 252.0 85.0 35.8 20.1 9.3 4.8
em−p 252 101 34 12 4 NA
before−matching
C−b 1118 369 178 102 61 30
em−b 626 218 86 28 9 NA
Melanoma cancer num. at risk
post−matching
C−p 438.0 152.3 62.6 35.6 20.4 10.9
Niv−p 438 120 21 6 2 NA
before−matching
C−b 1118 369 178 102 61 30
Niv−b 1148 314 60 22 2 NA
Melanoma cancer num. at risk
post−matching
C−p 1035.0 337.4 138.0 78.5 48.6 26.8
All−p 1035 331 115 51 26 7
before−matching
C−b 1118 369 178 102 61 30
All−b 2657 817 287 128 54 20

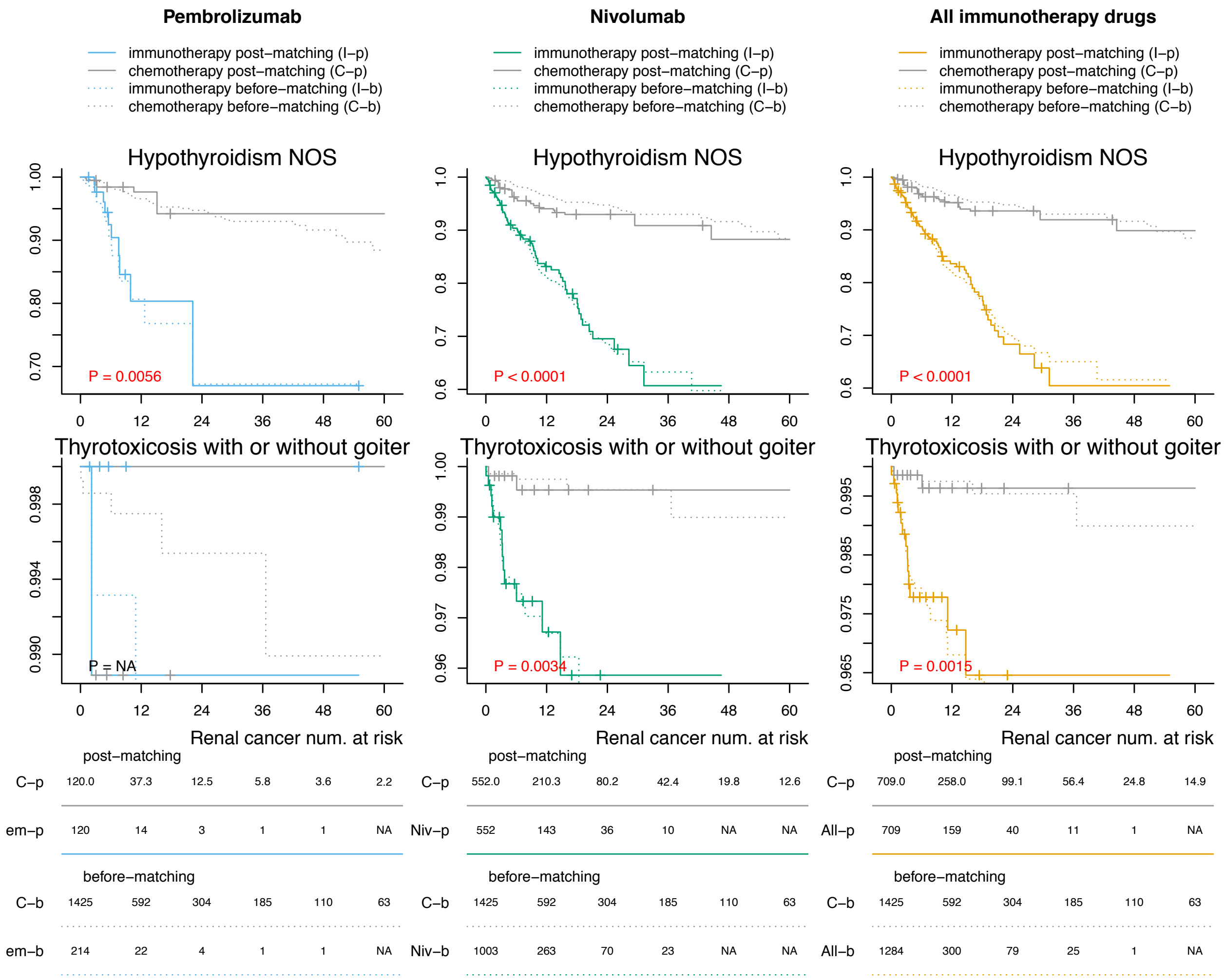

Pembrolizumab
Nivolumab
All immunotherapy drugs
immunotherapy post−matching (I−p)
chemotherapy post−matching (C−p)
immunotherapy before−matching (I−b)
chemotherapy before−matching (C−b)
Hypothyroidism NOS
P = 0.0056
P < 0.0001
P < 0.0001
Thyrotoxicosis with or without goiter
P = NA
P = 0.0034
P = 0.0015
0 12 24 36 48 60
Renal cancer num. at risk
post−matching
C−p 120.0 37.3 12.5 5.8 3.6 2.2
em−p 120 14 3 1 1 NA
C−p 552.0 210.3 80.2 42.4 19.8 12.6
Niv−p 552 143 36 10 NA NA
C−p 709.0 258.0 99.1 56.4 24.8 14.9
All−p 709 159 40 11 1 NA
before−matching
C−b 1425 592 304 185 110 63
em−b 214 22 4 1 1 NA
C−b 1425 592 304 185 110 63
Niv−b 1003 263 70 23 NA NA
C−b 1425 592 304 185 110 63
All−b 1284 300 79 25 1 NA

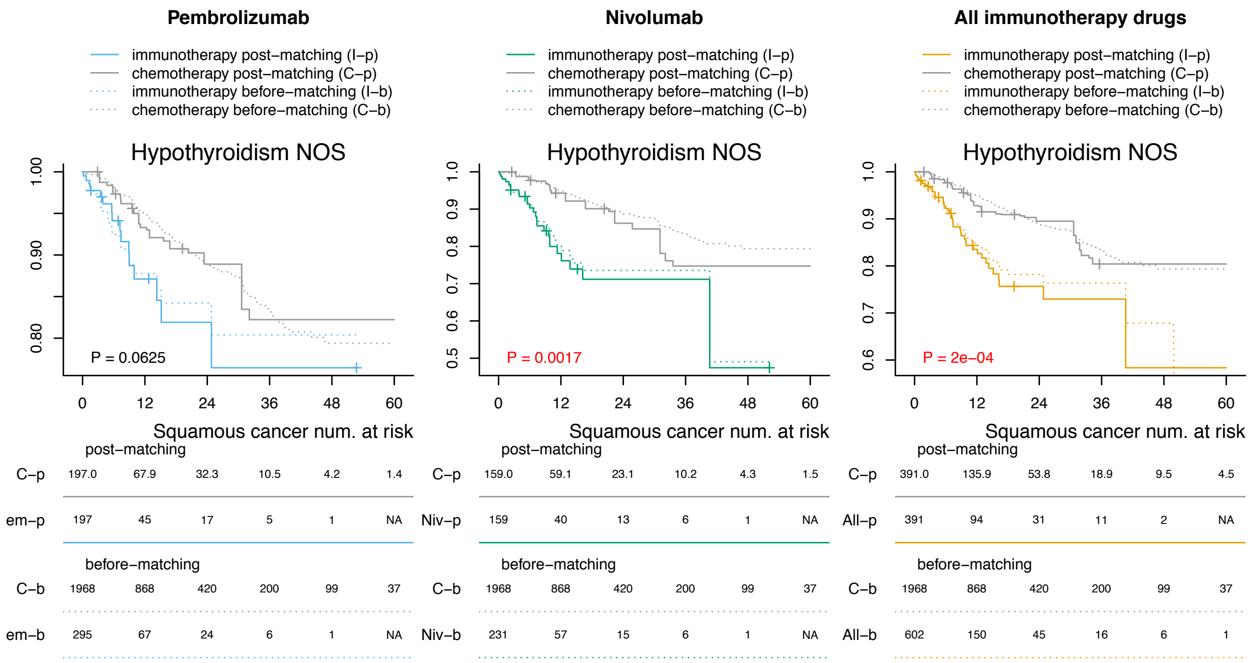

Pembrolizumab
Nivolumab
All immunotherapy drugs
immunotherapy post−matching (I−p)
chemotherapy post−matching (C−p)
immunotherapy before−matching (I−b)
chemotherapy before−matching (C−b)
Hypothyroidism NOS
P = 0.0625
P = 0.0017
P = 2e−04
0 12 24 36 48 60
Squamous cancer num. at risk
post−matching
C−p 197.0 67.9 32.3 10.5 4.2 1.4
em−p 197 45 17 5 1 NA
C−p 159.0 59.1 23.1 10.2 4.3 1.5
Niv−p 159 40 13 6 1 NA
C−p 391.0 135.9 53.8 18.9 9.5 4.5
All−p 391 94 31 11 2 NA
before−matching
C−b 1968 868 420 200 99 37
em−b 295 67 24 6 1 NA
C−b 1968 868 420 200 99 37
Niv−b 231 57 15 6 1 NA
C−b 1968 868 420 200 99 37
All−b 602 150 45 16 6 1

Targeted therapy

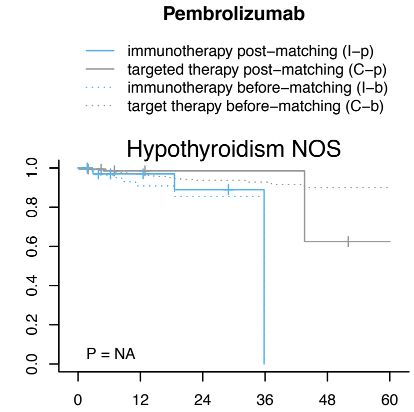

Brain cancer num. at risk

	0	12	24	36	48	60
post−matching						
C−p	95.0	21.1	4.6	1.7	0.6	NA
em−p	95	21	4	NA	NA	NA
before−matching						
C−b	2814	689	183	77	49	27
em−b	162	38	6	NA	NA	NA

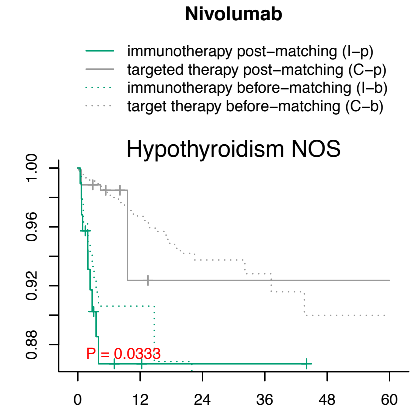

Brain cancer num. at risk

	0	12	24	36	48	60
post−matching						
C−p	100.0	25.9	4.4	1.1	1.0	0.9
Niv−p	100	19	2	1	NA	NA
before−matching						
C−b	2814	689	183	77	49	27
Niv−b	177	33	5	2	NA	NA

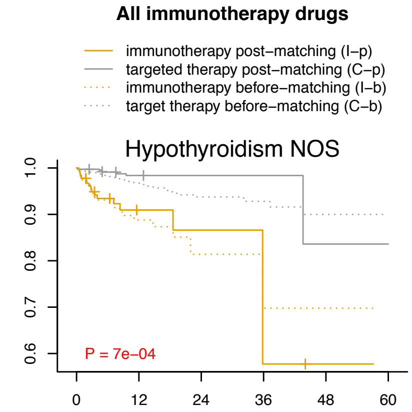

Brain cancer num. at risk

	0	12	24	36	48	60
post−matching						
C−p	235.0	52.9	9.8	3.1	1.9	1.0
All−p	235	45	7	2	NA	NA
before−matching						
C−b	2814	689	183	77	49	27
All−b	408	85	16	6	3	NA

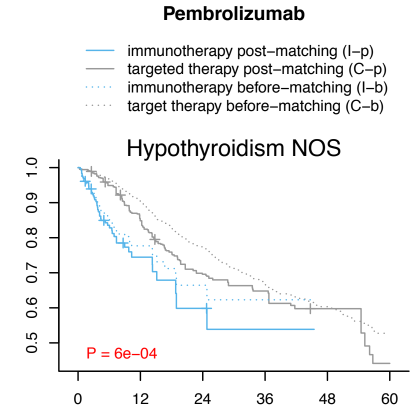

HeadAndNeck cancer num. at risk

	0	12	24	36	48	60
post−matching						
C−p	308.0	96.8	41.2	22.3	11.3	5.8
em−p	308	41	11	3	NA	NA
before−matching						
C−b	2455	969	471	237	140	83
em−b	444	66	19	6	NA	NA

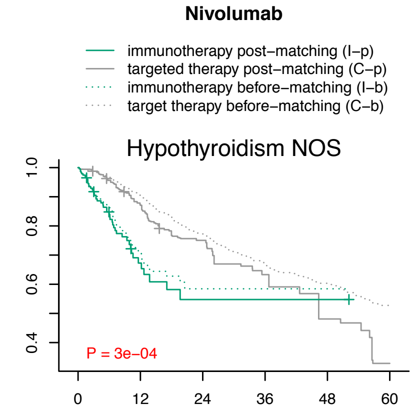

HeadAndNeck cancer num. at risk

	0	12	24	36	48	60
post−matching						
C−p	247.0	78.8	32.6	15.3	7.3	4.0
Niv−p	247	36	12	5	1	NA
before−matching						
C−b	2455	969	471	237	140	83
Niv−b	358	63	20	8	1	NA

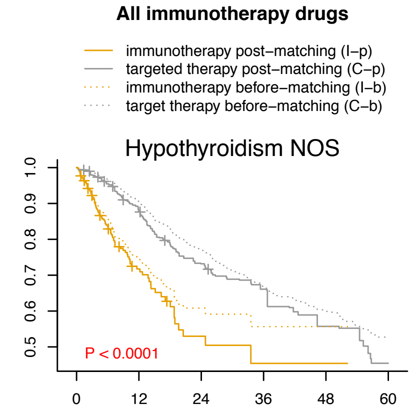

HeadAndNeck cancer num. at risk

	0	12	24	36	48	60
post−matching						
C−p	595.0	202.9	86.0	43.7	24.3	15.1
All−p	595	91	23	8	1	NA
before−matching						
C−b	2455	969	471	237	140	83
All−b	858	144	40	14	1	NA

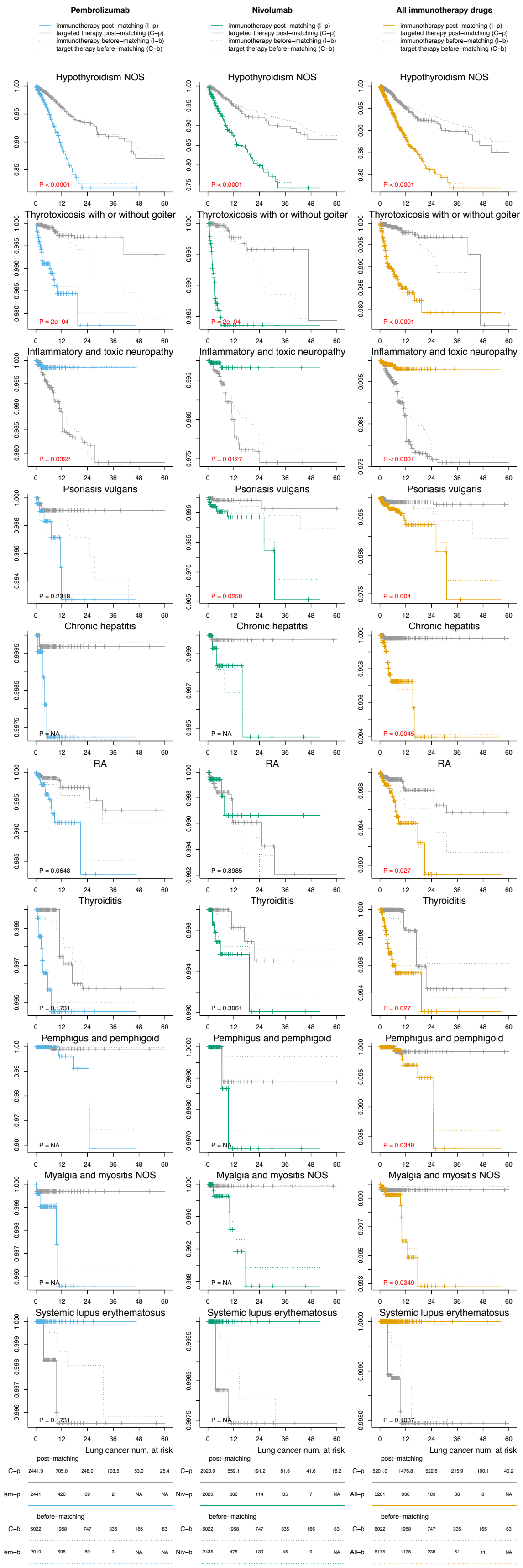
Pembrolizumab
Nivolumab
All immunotherapy drugs
immunotherapy post−matching (I−p)
targeted therapy post−matching (C−p)
immunotherapy before−matching (I−b)
target therapy before−matching (C−b)
Hypothyroidism NOS
P < 0.0001
Thyrotoxicosis with or without goiter
P = 2e−04
P < 0.0001
Inflammatory and toxic neuropathy
P = 0.0392
P = 0.0127
P < 0.0001
Psoriasis vulgaris
P = 0.2318
P = 0.0258
P = 0.004
Chronic hepatitis
P = NA
P = 0.0045
RA
P = 0.0648
P = 0.8985
P = 0.027
Thyroiditis
P = 0.1731
P = 0.3061
P = 0.027
Pemphigus and pemphigoid
P = NA
P = 0.0349
Myalgia and myositis NOS
P = NA
P = 0.0349
Systemic lupus erythematosus
P = 0.1731
P = NA
P = 0.1037
Lung cancer num. at risk
post−matching
C−p 2441.0 705.0 248.0 103.5 53.0 25.4
em−p 2441 420 69 2 NA NA
before−matching
C−b 6022 1958 747 335 166 83
em−b 2919 505 89 3 NA NA
C−p 2020.0 559.1 191.2 81.6 41.6 18.2
Niv−p 2020 386 114 35 7 NA
C−b 6022 1958 747 335 166 83
Niv−b 2435 478 139 45 9 NA
C−p 5201.0 1476.8 522.6 215.9 100.1 40.2
All−p 5201 936 189 38 8 NA
C−b 6022 1958 747 335 166 83
All−b 6175 1135 238 51 11 NA

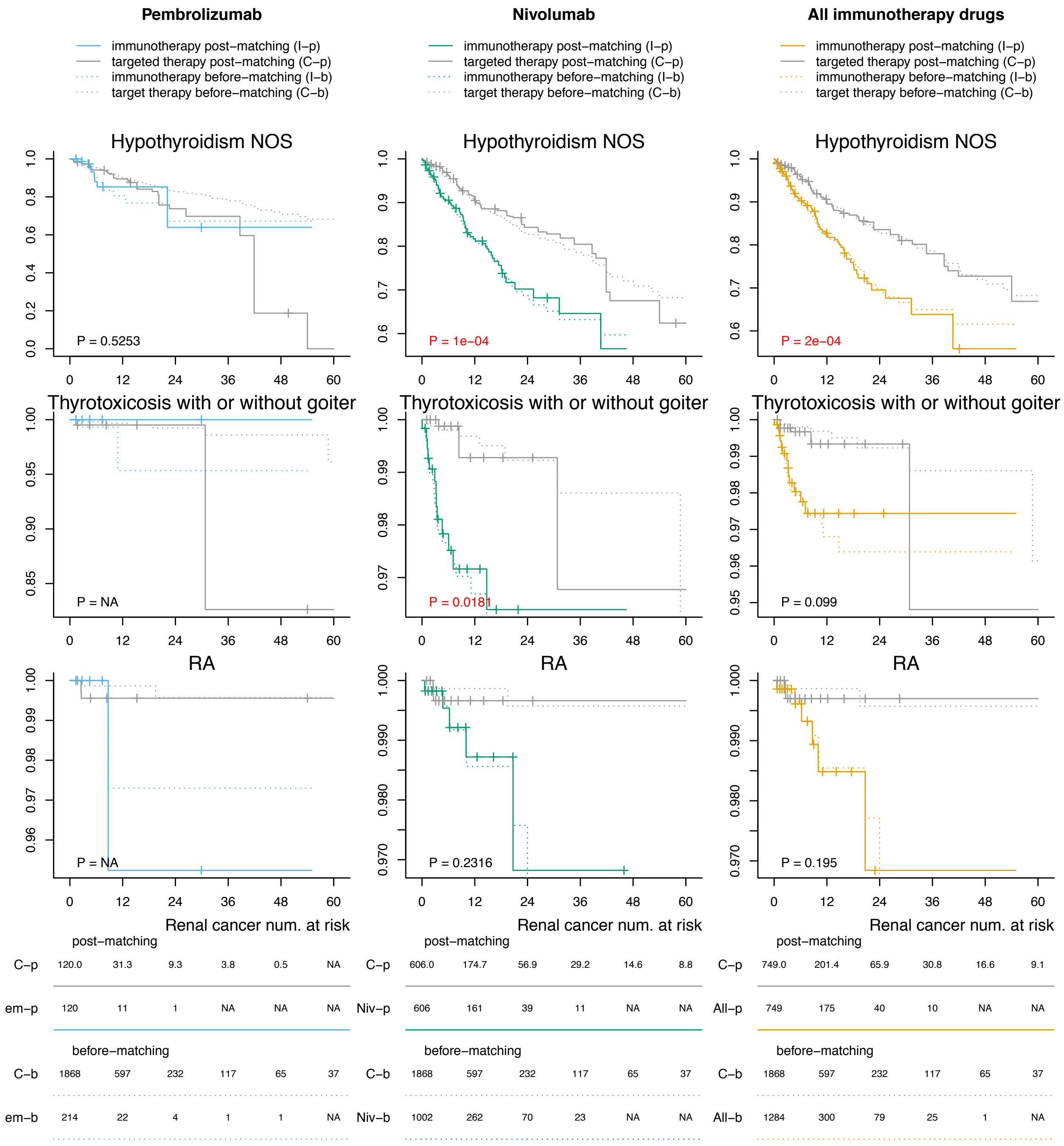
Pembrolizumab
Nivolumab
All immunotherapy drugs
immunotherapy post−matching (I−p)
targeted therapy post−matching (C−p)
immunotherapy before−matching (I−b)
target therapy before−matching (C−b)
Hypothyroidism NOS
P = 0.5253
P = 1e−04
P = 2e−04
Thyrotoxicosis with or without goiter
P = NA
P = 0.0181
P = 0.099
RA
P = NA
P = 0.2316
P = 0.195
0 12 24 36 48 60
Renal cancer num. at risk
post−matching
C−p 120.0 31.3 9.3 3.8 0.5 NA
em−p 120 11 1 NA NA NA
before−matching
C−b 1868 597 232 117 65 37
em−b 214 22 4 1 1 NA
C−p 606.0 174.7 56.9 29.2 14.6 8.8
Niv−p 606 161 39 11 NA NA
C−b 1868 597 232 117 65 37
Niv−b 1002 262 70 23 NA NA
C−p 749.0 201.4 65.9 30.8 16.6 9.1
All−p 749 175 40 10 NA NA
C−b 1868 597 232 117 65 37
All−b 1284 300 79 25 1 NA

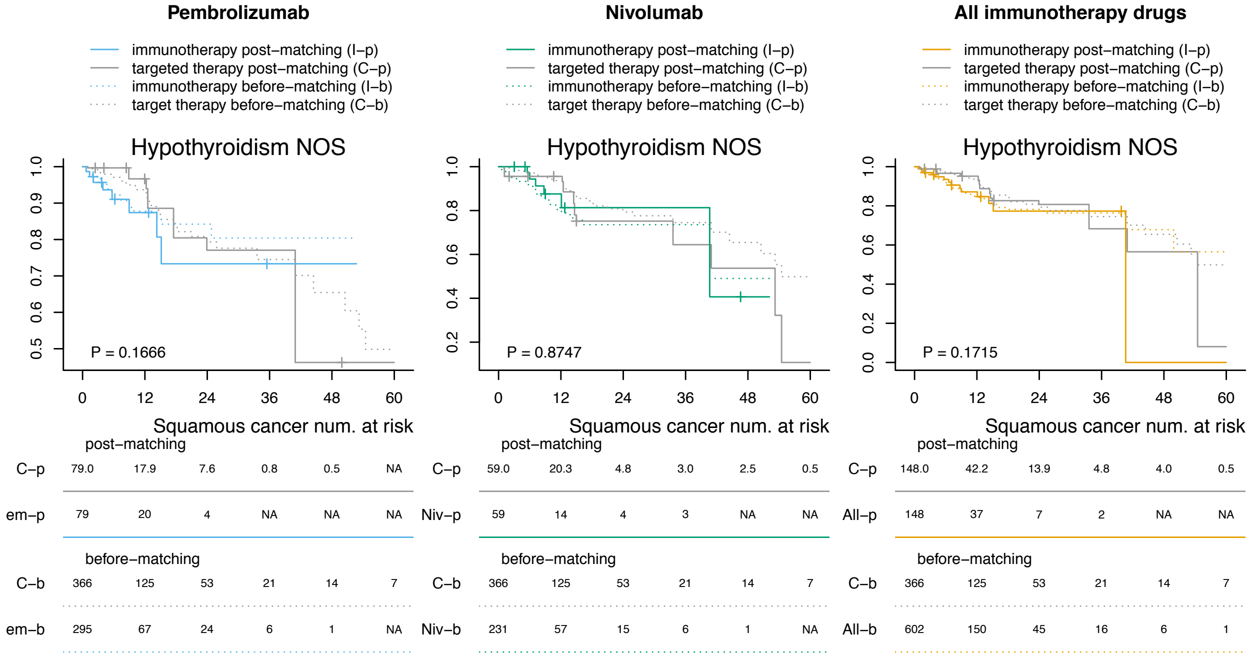
Pembrolizumab
immunotherapy post−matching (I−p)
targeted therapy post−matching (C−p)
immunotherapy before−matching (I−b)
target therapy before−matching (C−b)
Hypothyroidism NOS
P = 0.1666
0 12 24 36 48 60
Squamous cancer num. at risk
post−matching
C−p 79.0 17.9 7.6 0.8 0.5 NA
em−p 79 20 4 NA NA NA
before−matching
C−b 366 125 53 21 14 7
em−b 295 67 24 6 1 NA
Nivolumab
immunotherapy post−matching (I−p)
targeted therapy post−matching (C−p)
immunotherapy before−matching (I−b)
target therapy before−matching (C−b)
Hypothyroidism NOS
P = 0.8747
0 12 24 36 48 60
Squamous cancer num. at risk
post−matching
C−p 59.0 20.3 4.8 3.0 2.5 0.5
Niv−p 59 14 4 3 NA NA
before−matching
C−b 366 125 53 21 14 7
Niv−b 231 57 15 6 1 NA
All immunotherapy drugs
immunotherapy post−matching (I−p)
targeted therapy post−matching (C−p)
immunotherapy before−matching (I−b)
target therapy before−matching (C−b)
Hypothyroidism NOS
P = 0.1715
0 12 24 36 48 60
Squamous cancer num. at risk
post−matching
C−p 148.0 42.2 13.9 4.8 4.0 0.5
All−p 148 37 7 2 NA NA
before−matching
C−b 366 125 53 21 14 7
All−b 602 150 45 16 6 1

Figure S5. Time-to-event plot showing the time between treatment initiation and the development of autoimmune diseases in seven cancer types. The comparisons between chemotherapy with pembrolizumab, nivolumab, and all immune checkpoint inhibitors combined are shown in the three columns respectively. For each cancer type, we showed the time-to-event plot for all autoimmune diseases, with a log-rank test p-value reported in the top right corner for both matched and unmatched samples. (The p-values are the same as the 15 months horizon in the main text because they are both calculated based on all observations.) Each tick represents 50 censored patients. The curves offer a full time horizon up to 5 years after treatment initiation. The tabulate information at the bottom shows the number of patients without censored information at 0, 1, 2, 3, 4, and 5 years.

Chemotherapy

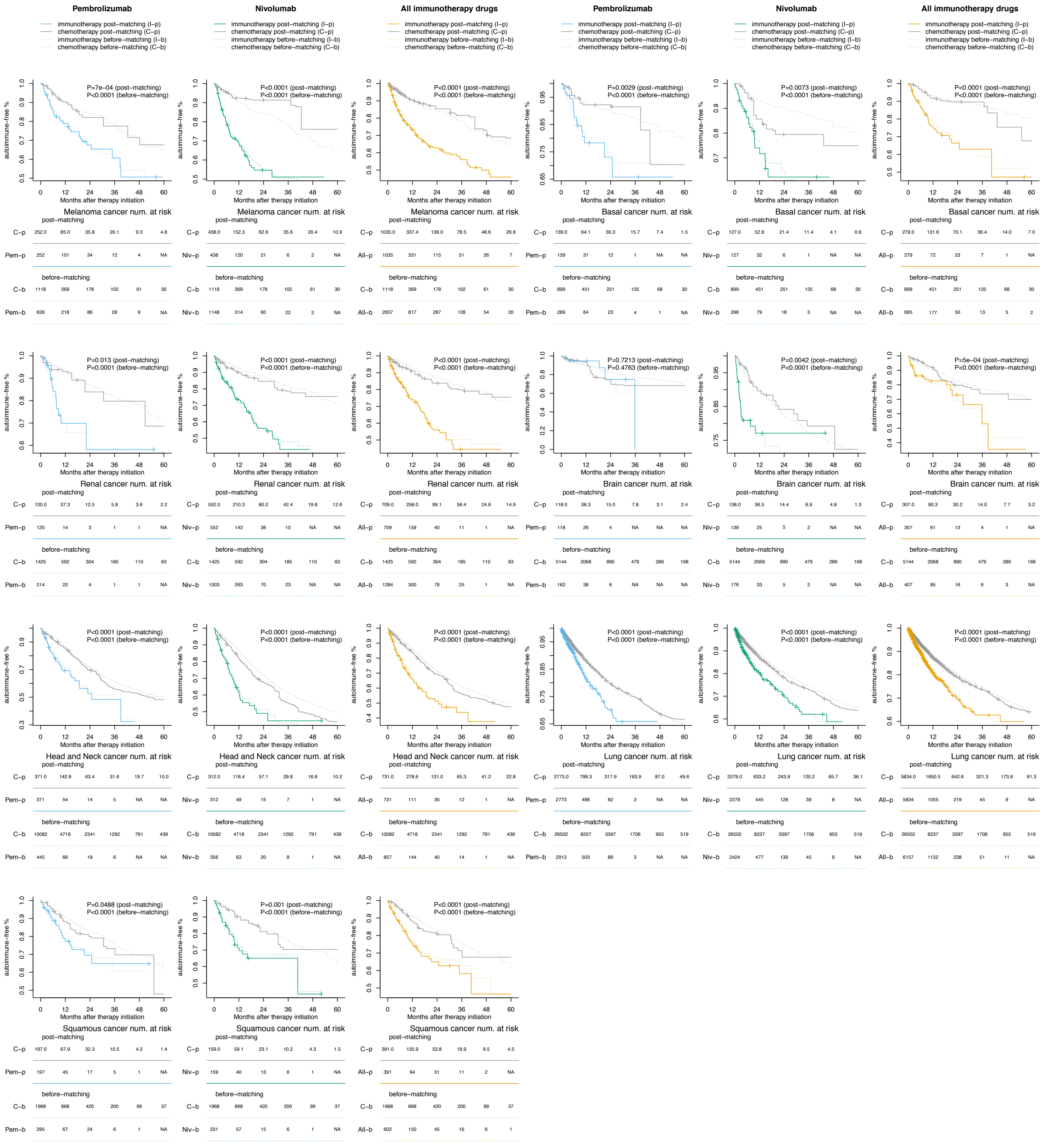

Targeted therapy

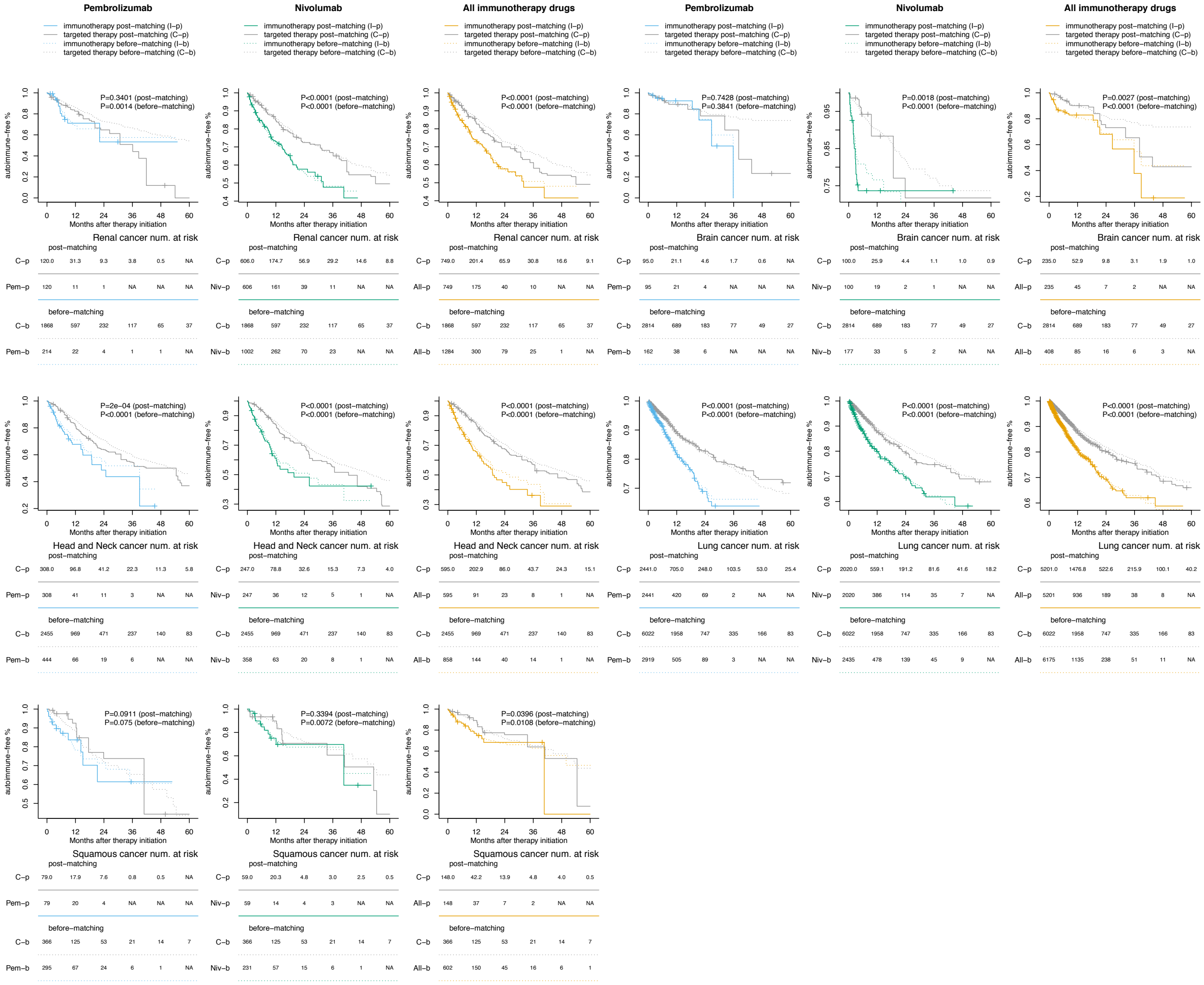

Sensitivity Analyses

Different Categorization of Continuous Variables for Matching

In the main analyses, we categorized the continuous variables (i.e., zip-code-defined median income, the annual frequency of hospital visits prior to treatment initialization, and the annual frequency of ICD code counts prior to treatment initialization) into 5 categories with equal proportions (20%) for matching. In the following sensitivity analyses, we compared the hazard ratio results when using 3, 4, and 5 bins to categorize the continuous variables.

Figure S6. Sensitivity to the number of categories for binning the continuous variables. The point estimates of the hazard ratios comparing the immunotherapy group with the conventional treatment groups in 3, 6, 9, 12, and 15 months after treatment initiation are shown. The number on the top right indicates the number of matched patients in the treatment group. The left, middle and right panels correspond to 3, 4, and 5 categories, respectively. The top panel displays the hazard ratio estimates, and the bottom panel displays the 95% CI. The hazard ratio estimates did not vary much when we used different numbers of bins.

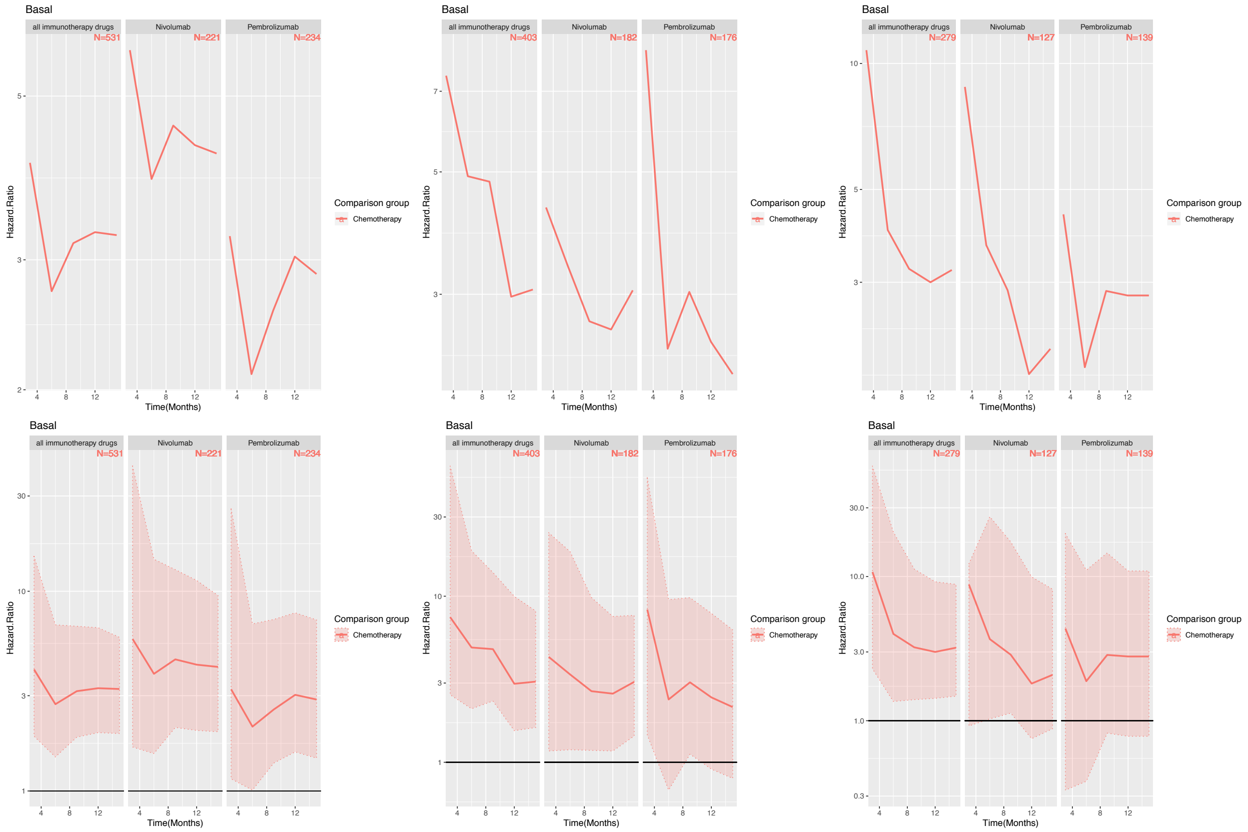

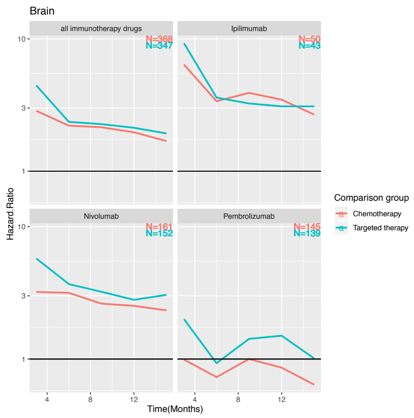
Brain
all immunotherapy drugs
N=368
N=347
Ipilimumab
N=50
N=43
Nivolumab
N=161
N=152
Pembrolizumab
N=145
N=139
Hazard.Ratio
Time(Months)
Comparison group
Chemotherapy
Targeted therapy

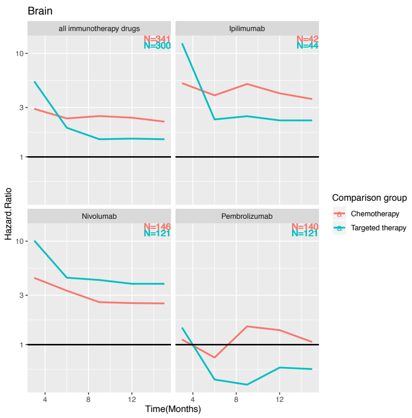
Brain
all immunotherapy drugs
N=341
N=300
Ipilimumab
N=42
N=44
Nivolumab
N=146
N=121
Pembrolizumab
N=140
N=121
Hazard.Ratio
Time(Months)
Comparison group
Chemotherapy
Targeted therapy

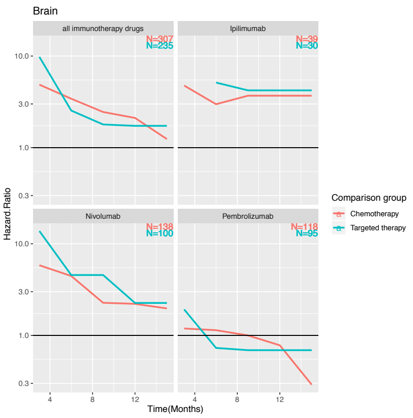
Brain
all immunotherapy drugs
N=307
N=235
Ipilimumab
N=39
N=30
Nivolumab
N=138
N=100
Pembrolizumab
N=118
N=95
Hazard.Ratio
Time(Months)
Comparison group
Chemotherapy
Targeted therapy

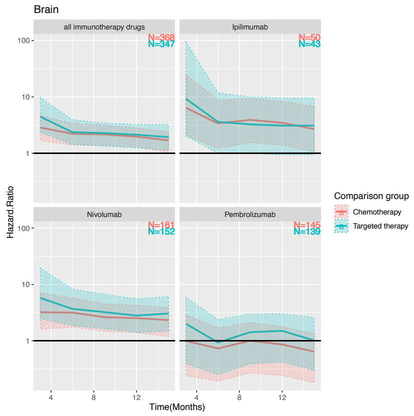
Brain
all immunotherapy drugs
N=368
N=347
Ipilimumab
N=50
N=43
Nivolumab
N=161
N=152
Pembrolizumab
N=145
N=139
Hazard.Ratio
Time(Months)
Comparison group
Chemotherapy
Targeted therapy

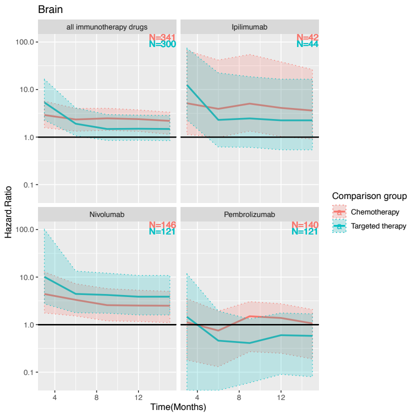
Brain
all immunotherapy drugs
N=341
N=300
Ipilimumab
N=42
N=44
Nivolumab
N=146
N=121
Pembrolizumab
N=140
N=121
Hazard.Ratio
Time(Months)
Comparison group
Chemotherapy
Targeted therapy

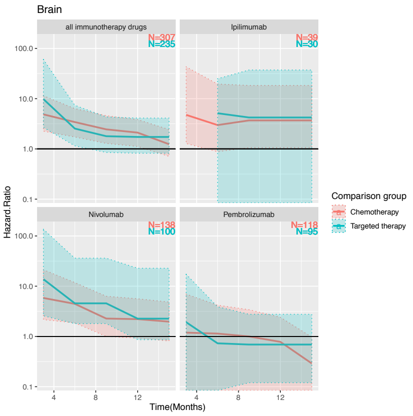
Brain
all immunotherapy drugs
N=307
N=235
Ipilimumab
N=39
N=30
Nivolumab
N=138
N=100
Pembrolizumab
N=118
N=95
Hazard.Ratio
Time(Months)
Comparison group
Chemotherapy
Targeted therapy

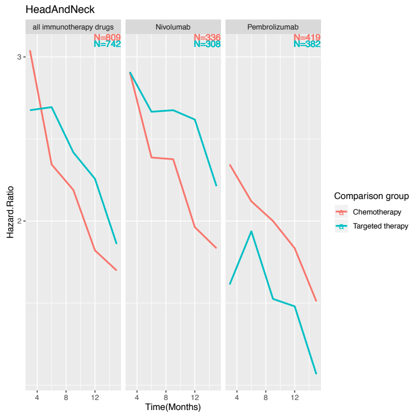
HeadAndNeck
all immunotherapy drugs
N=809
N=742
Nivolumab
N=336
N=308
Pembrolizumab
N=419
N=382
Hazard.Ratio
Time(Months)
Comparison group
Chemotherapy
Targeted therapy

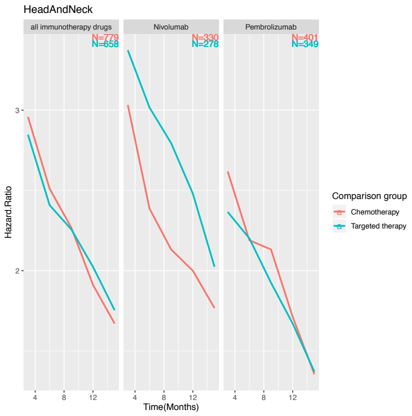
HeadAndNeck
all immunotherapy drugs
N=779
N=658
Nivolumab
N=330
N=278
Pembrolizumab
N=401
N=349
Hazard.Ratio
Time(Months)
Comparison group
Chemotherapy
Targeted therapy

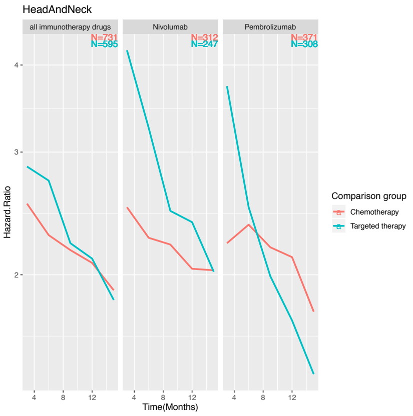
HeadAndNeck
all immunotherapy drugs
N=731
N=595
Nivolumab
N=312
N=247
Pembrolizumab
N=371
N=308
Hazard.Ratio
Time(Months)
Comparison group
Chemotherapy
Targeted therapy

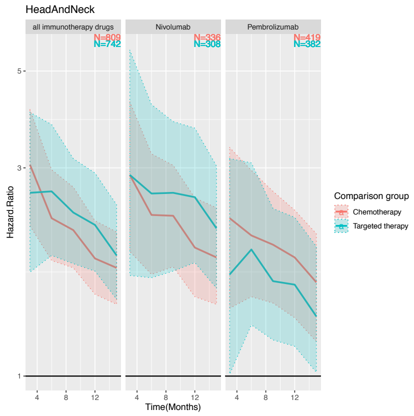
HeadAndNeck
all immunotherapy drugs
N=809
N=742
Nivolumab
N=336
N=308
Pembrolizumab
N=419
N=382
Hazard.Ratio
Time(Months)
Comparison group
Chemotherapy
Targeted therapy

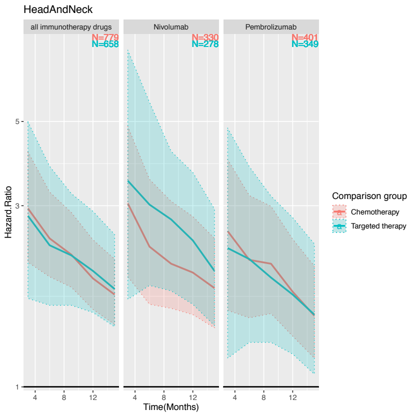
HeadAndNeck
all immunotherapy drugs
N=779
N=658
Nivolumab
N=330
N=278
Pembrolizumab
N=401
N=349
Hazard.Ratio
Time(Months)
Comparison group
Chemotherapy
Targeted therapy

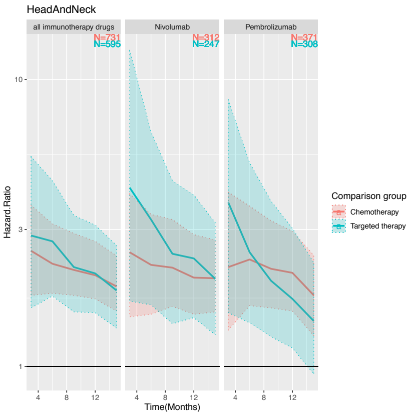
HeadAndNeck
all immunotherapy drugs
N=731
N=595
Nivolumab
N=312
N=247
Pembrolizumab
N=371
N=308
Hazard.Ratio
Time(Months)
Comparison group
Chemotherapy
Targeted therapy

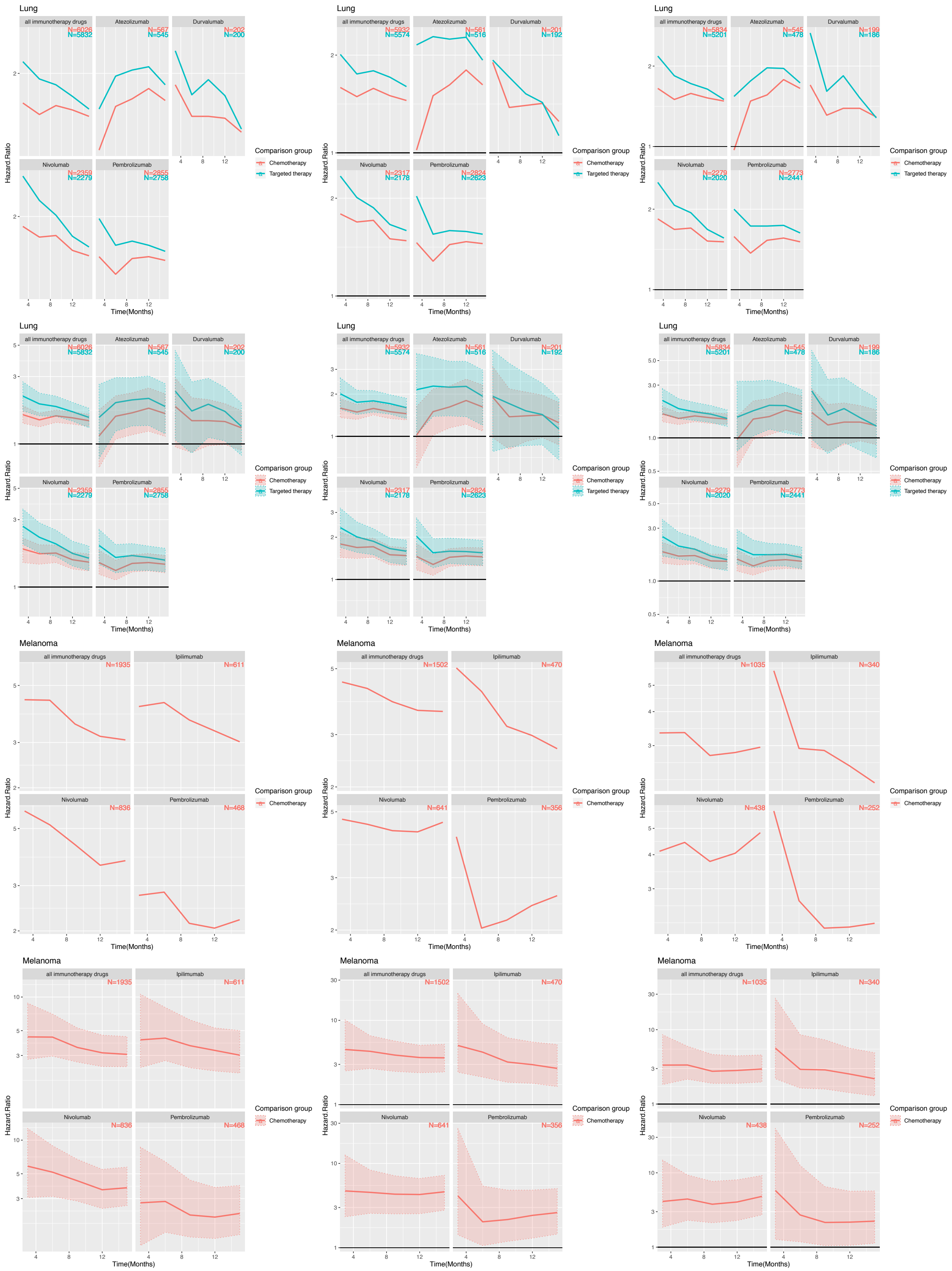
Lung
all immunotherapy drugs
N=6026
N=5832
Atezolizumab
N=567
N=545
Durvalumab
N=202
N=200
Nivolumab
N=2359
N=2279
Pembrolizumab
N=2855
N=2758
Hazard Ratio
Time(Months)
Comparison group
Chemotherapy
Targeted therapy
Lung
all immunotherapy drugs
N=5932
N=5574
Atezolizumab
N=561
N=516
Durvalumab
N=201
N=192
Nivolumab
N=2317
N=2178
Pembrolizumab
N=2824
N=2623
Hazard Ratio
Time(Months)
Comparison group
Chemotherapy
Targeted therapy
Lung
all immunotherapy drugs
N=5834
N=5201
Atezolizumab
N=545
N=478
Durvalumab
N=199
N=186
Nivolumab
N=2279
N=2020
Pembrolizumab
N=2773
N=2441
Hazard Ratio
Time(Months)
Comparison group
Chemotherapy
Targeted therapy
Melanoma
all immunotherapy drugs
N=1935
Ipilimumab
N=611
Nivolumab
N=836
Pembrolizumab
N=468
Hazard Ratio
Time(Months)
Comparison group
Chemotherapy
Melanoma
all immunotherapy drugs
N=1502
Ipilimumab
N=470
Nivolumab
N=641
Pembrolizumab
N=356
Hazard Ratio
Time(Months)
Comparison group
Chemotherapy
Melanoma
all immunotherapy drugs
N=1035
Ipilimumab
N=340
Nivolumab
N=438
Pembrolizumab
N=252
Hazard Ratio
Time(Months)
Comparison group
Chemotherapy

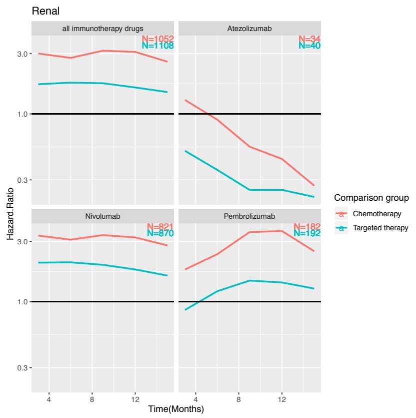
Renal
all immunotherapy drugs
N=1052
N=1108
Atezolizumab
N=34
N=40
Nivolumab
N=821
N=870
Pembrolizumab
N=182
N=192
Hazard.Ratio
Time(Months)
Comparison group
Chemotherapy
Targeted therapy
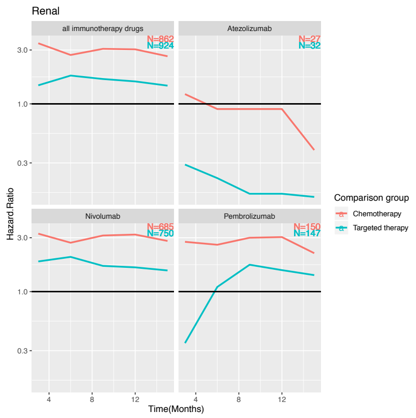
Renal
all immunotherapy drugs
N=862
N=924
Atezolizumab
N=27
N=32
Nivolumab
N=685
N=750
Pembrolizumab
N=150
N=147
Hazard.Ratio
Time(Months)
Comparison group
Chemotherapy
Targeted therapy
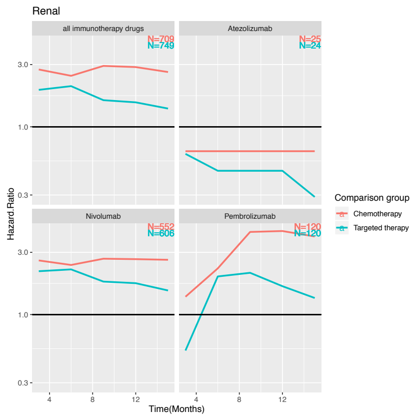
Renal
all immunotherapy drugs
N=709
N=749
Atezolizumab
N=25
N=24
Nivolumab
N=552
N=606
Pembrolizumab
N=120
N=120
Hazard.Ratio
Time(Months)
Comparison group
Chemotherapy
Targeted therapy
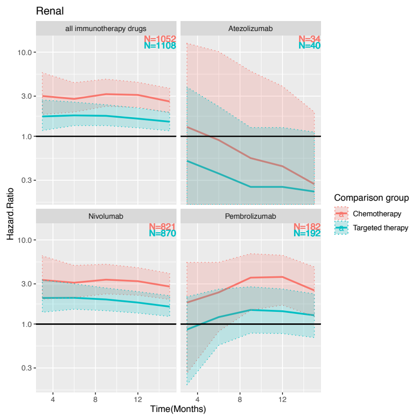
Renal
all immunotherapy drugs
N=1052
N=1108
Atezolizumab
N=34
N=40
Nivolumab
N=821
N=870
Pembrolizumab
N=182
N=192
Hazard.Ratio
Time(Months)
Comparison group
Chemotherapy
Targeted therapy
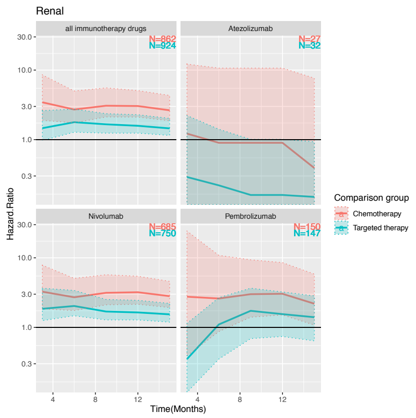
Renal
all immunotherapy drugs
N=862
N=924
Atezolizumab
N=27
N=32
Nivolumab
N=685
N=750
Pembrolizumab
N=150
N=147
Hazard.Ratio
Time(Months)
Comparison group
Chemotherapy
Targeted therapy
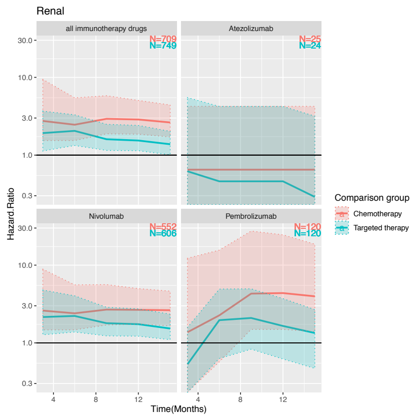
Renal
all immunotherapy drugs
N=709
N=749
Atezolizumab
N=25
N=24
Nivolumab
N=552
N=606
Pembrolizumab
N=120
N=120
Hazard.Ratio
Time(Months)
Comparison group
Chemotherapy
Targeted therapy
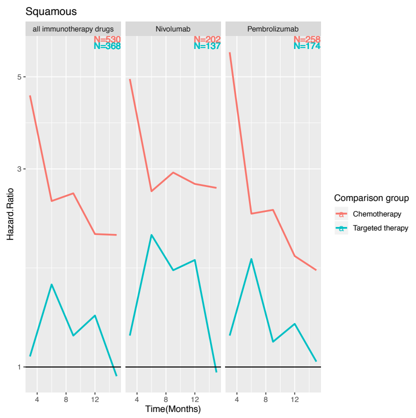
Squamous
all immunotherapy drugs
N=530
N=368
Nivolumab
N=202
N=137
Pembrolizumab
N=258
N=174
Hazard.Ratio
Time(Months)
Comparison group
Chemotherapy
Targeted therapy
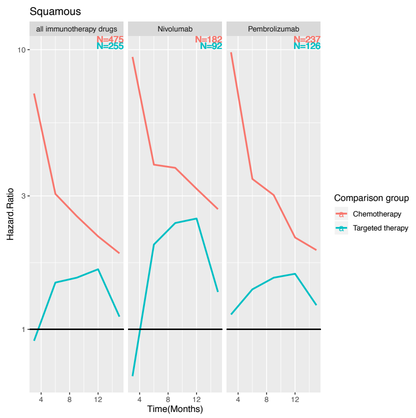
Squamous
all immunotherapy drugs
N=475
N=255
Nivolumab
N=182
N=92
Pembrolizumab
N=237
N=126
Hazard.Ratio
Time(Months)
Comparison group
Chemotherapy
Targeted therapy
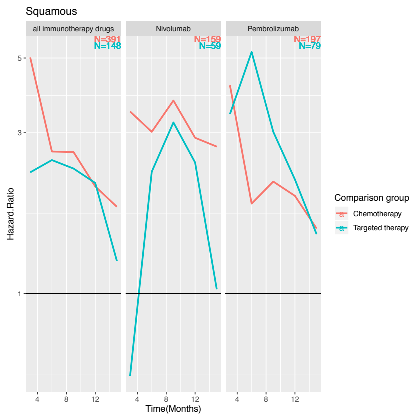
Squamous
all immunotherapy drugs
N=391
N=148
Nivolumab
N=159
N=59
Pembrolizumab
N=197
N=79
Hazard.Ratio
Time(Months)
Comparison group
Chemotherapy
Targeted therapy
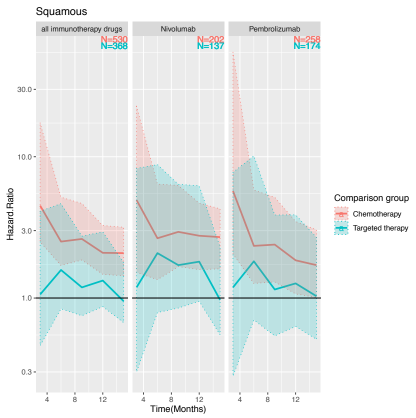
Squamous
all immunotherapy drugs
N=530
N=368
Nivolumab
N=202
N=137
Pembrolizumab
N=258
N=174
Hazard.Ratio
Time(Months)
Comparison group
Chemotherapy
Targeted therapy
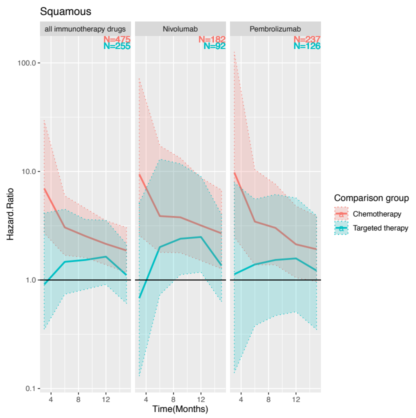
Squamous
all immunotherapy drugs
N=475
N=255
Nivolumab
N=182
N=92
Pembrolizumab
N=237
N=126
Hazard.Ratio
Time(Months)
Comparison group
Chemotherapy
Targeted therapy
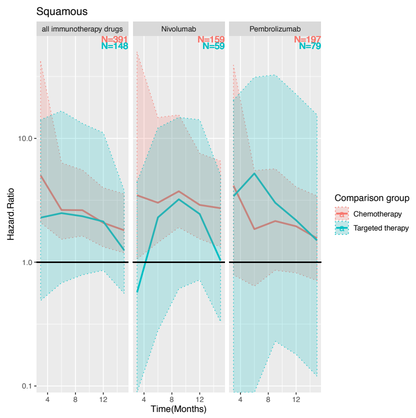
Squamous
all immunotherapy drugs
N=391
N=148
Nivolumab
N=159
N=59
Pembrolizumab
N=197
N=79
Hazard.Ratio
Time(Months)
Comparison group
Chemotherapy
Targeted therapy

Different Quiescence Period Requirement

We examined the statistical models with different quiescence period requirements in this section. In particular, we fitted the same time-to-autoimmunity models and require the treatment initiation dates to be at least 10, 30, 60, 90, 180 days after the insurance enrollment. Figure S7 shows the sensitivity analysis results. The increased hazards for autoimmune diseases in the immunotherapy group are consistent throughout different quiescence period requirements.

Figure S7. Sensitivity analyses on the quiescence period requirement for renal cancer.

Chemotherapy

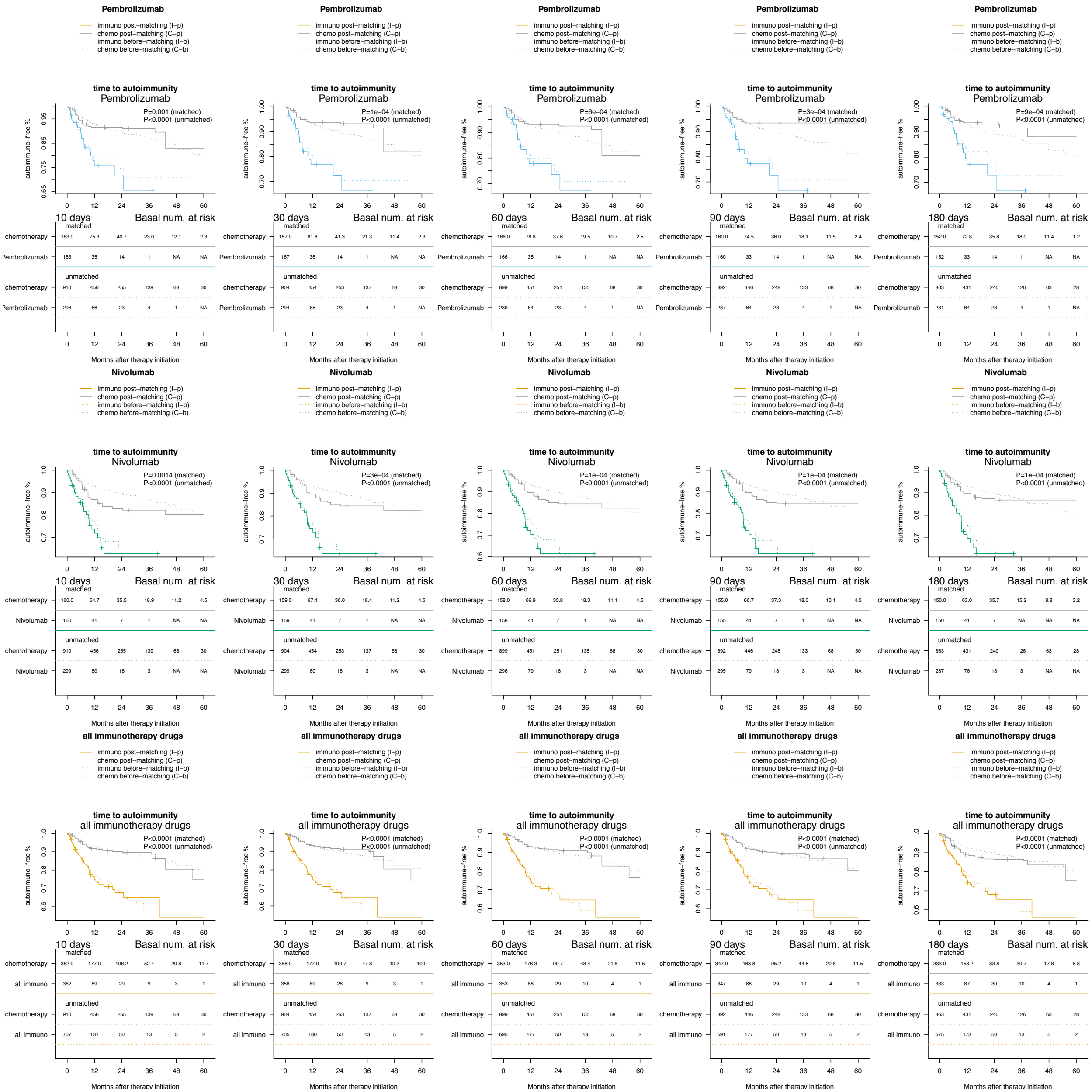

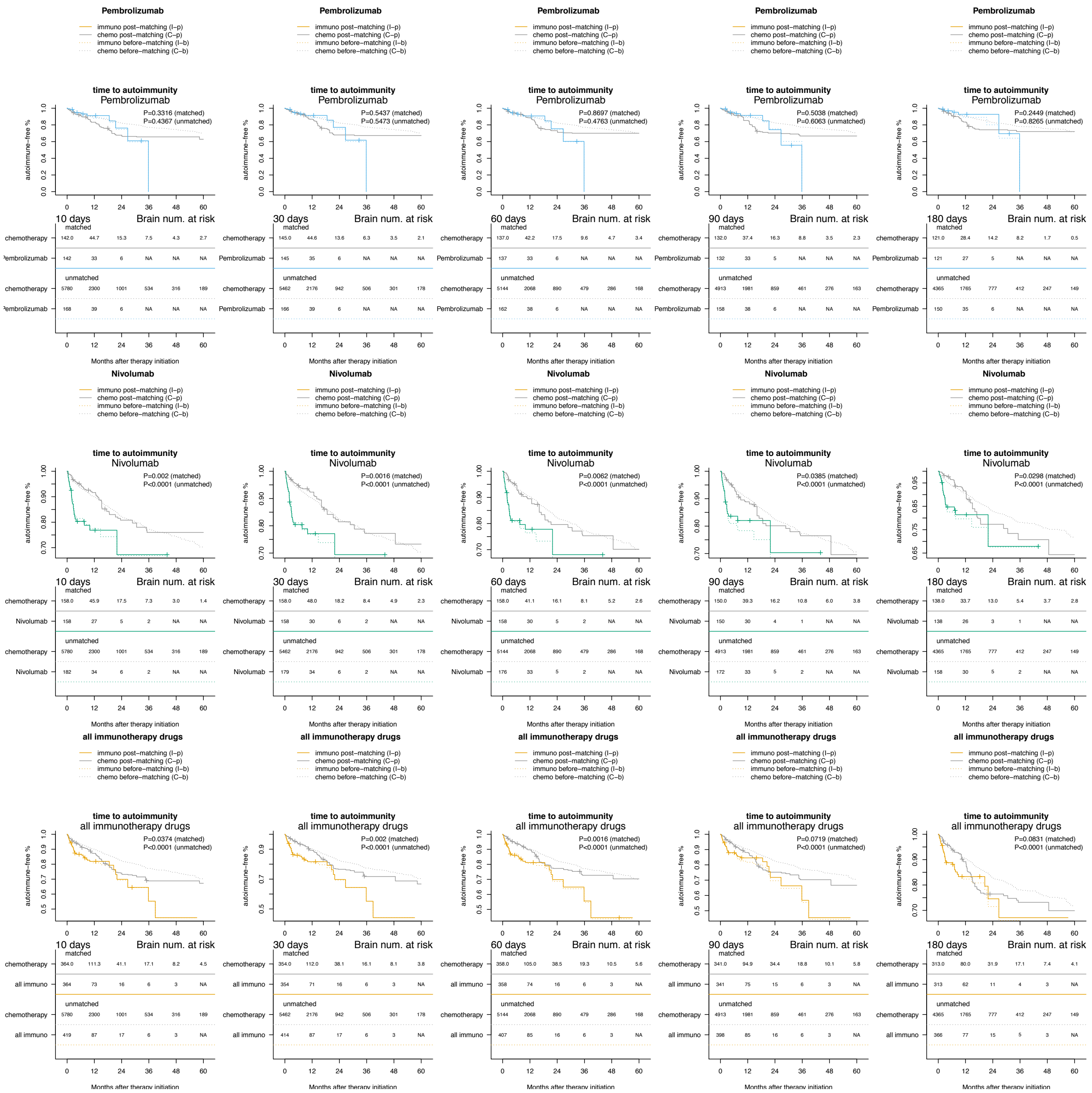
Pembrolizumab
immuno post−matching (I−p)
chemo post−matching (C−p)
immuno before−matching (I−b)
chemo before−matching (C−b)
time to autoimmunity
Pembrolizumab
autoimmune−free %
Months after therapy initiation
Brain num. at risk
matched
unmatched
10 days
P=0.3316 (matched)
P=0.4367 (unmatched)
chemotherapy 142.0 44.7 15.3 7.5 4.3 2.7
Pembrolizumab 142 33 6 NA NA NA
chemotherapy 5780 2300 1001 534 316 189
Pembrolizumab 168 39 6 NA NA NA
30 days
P=0.5437 (matched)
P=0.5473 (unmatched)
chemotherapy 145.0 44.6 13.6 6.3 3.5 2.1
Pembrolizumab 145 35 6 NA NA NA
chemotherapy 5462 2176 942 506 301 178
Pembrolizumab 166 39 6 NA NA NA
60 days
P=0.8697 (matched)
P=0.4763 (unmatched)
chemotherapy 137.0 42.2 17.5 9.6 4.7 3.4
Pembrolizumab 137 33 6 NA NA NA
chemotherapy 5144 2068 890 479 286 168
Pembrolizumab 162 38 6 NA NA NA
90 days
P=0.5038 (matched)
P=0.6063 (unmatched)
chemotherapy 132.0 37.4 16.3 8.8 3.5 2.3
Pembrolizumab 132 33 5 NA NA NA
chemotherapy 4913 1981 859 461 276 163
Pembrolizumab 158 38 6 NA NA NA
180 days
P=0.2449 (matched)
P=0.8265 (unmatched)
chemotherapy 121.0 28.4 14.2 8.2 1.7 0.5
Pembrolizumab 121 27 5 NA NA NA
chemotherapy 4365 1765 777 412 247 149
Pembrolizumab 150 35 6 NA NA NA
Nivolumab
time to autoimmunity
Nivolumab
10 days
P=0.002 (matched)
P<0.0001 (unmatched)
chemotherapy 158.0 45.9 17.5 7.3 3.0 1.4
Nivolumab 158 27 5 2 NA NA
chemotherapy 5780 2300 1001 534 316 189
Nivolumab 182 34 6 2 NA NA
30 days
P=0.0016 (matched)
P<0.0001 (unmatched)
chemotherapy 158.0 48.0 18.2 8.4 4.9 2.3
Nivolumab 158 30 6 2 NA NA
chemotherapy 5462 2176 942 506 301 178
Nivolumab 179 34 6 2 NA NA
60 days
P=0.0062 (matched)
P<0.0001 (unmatched)
chemotherapy 158.0 41.1 16.1 8.1 5.2 2.6
Nivolumab 158 30 5 2 NA NA
chemotherapy 5144 2068 890 479 286 168
Nivolumab 176 33 5 2 NA NA
90 days
P=0.0385 (matched)
P<0.0001 (unmatched)
chemotherapy 150.0 39.3 16.2 10.8 6.0 3.8
Nivolumab 150 30 4 1 NA NA
chemotherapy 4913 1981 859 461 276 163
Nivolumab 172 33 5 2 NA NA
180 days
P=0.0298 (matched)
P<0.0001 (unmatched)
chemotherapy 138.0 33.7 13.0 5.4 3.7 2.8
Nivolumab 138 26 3 1 NA NA
chemotherapy 4365 1765 777 412 247 149
Nivolumab 158 30 5 2 NA NA
all immunotherapy drugs
time to autoimmunity
all immunotherapy drugs
10 days
P=0.0374 (matched)
P<0.0001 (unmatched)
chemotherapy 364.0 111.3 41.1 17.1 8.2 4.5
all immuno 364 73 16 6 3 NA
chemotherapy 5780 2300 1001 534 316 189
all immuno 419 87 17 6 3 NA
30 days
P=0.002 (matched)
P<0.0001 (unmatched)
chemotherapy 354.0 112.0 38.1 16.1 8.1 3.8
all immuno 354 71 16 6 3 NA
chemotherapy 5462 2176 942 506 301 178
all immuno 414 87 17 6 3 NA
60 days
P=0.0016 (matched)
P<0.0001 (unmatched)
chemotherapy 358.0 105.0 38.5 19.3 10.5 5.6
all immuno 358 74 16 6 3 NA
chemotherapy 5144 2068 890 479 286 168
all immuno 407 85 16 6 3 NA
90 days
P=0.0719 (matched)
P<0.0001 (unmatched)
chemotherapy 341.0 94.9 34.4 18.8 10.1 5.8
all immuno 341 75 15 6 3 NA
chemotherapy 4913 1981 859 461 276 163
all immuno 398 85 16 6 3 NA
180 days
P=0.0831 (matched)
P<0.0001 (unmatched)
chemotherapy 313.0 80.0 31.9 17.1 7.4 4.1
all immuno 313 62 11 4 3 NA
chemotherapy 4365 1765 777 412 247 149
all immuno 366 77 15 5 3 NA

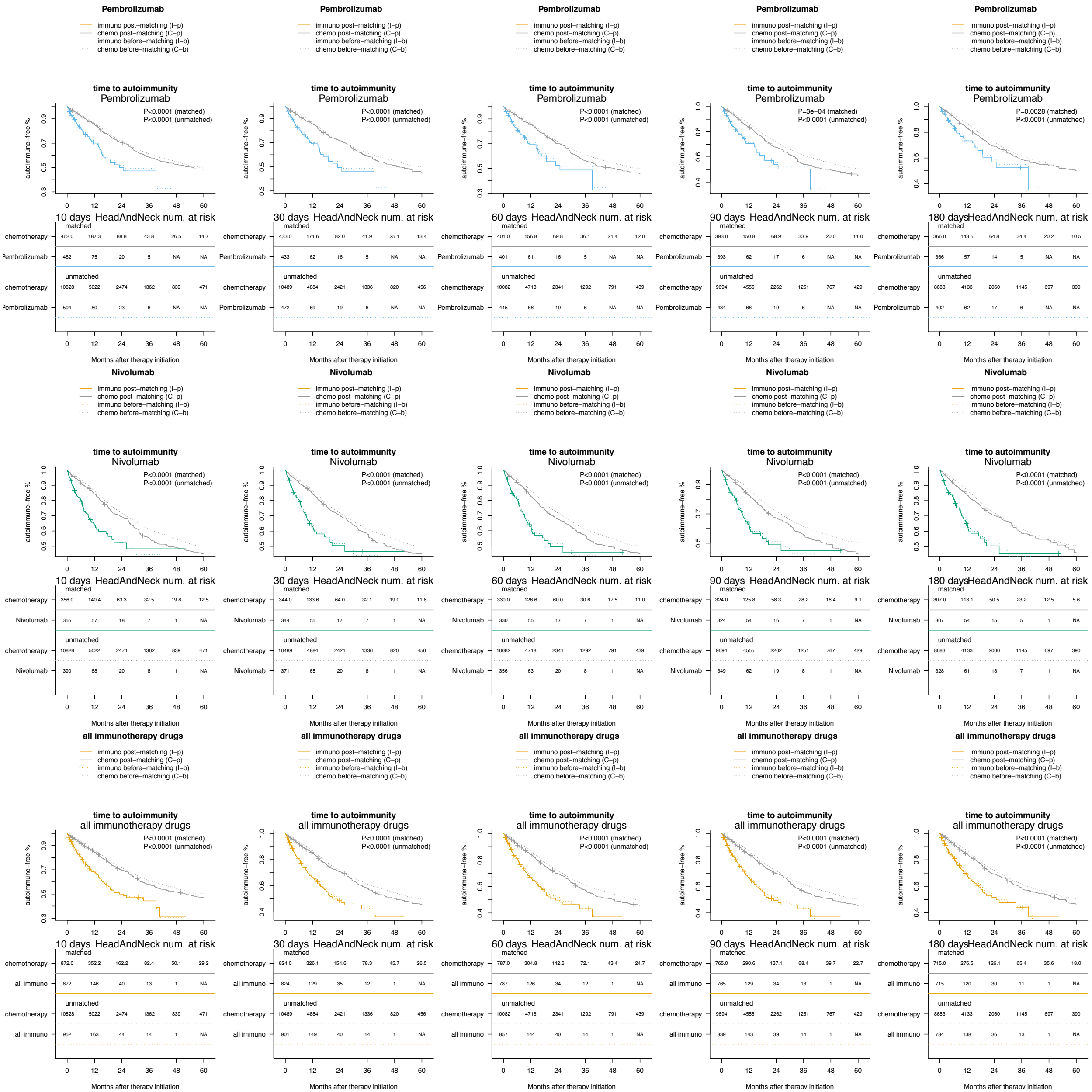

Pembrolizumab
immuno post−matching (I−p)
chemo post−matching (C−p)
immuno before−matching (I−b)
chemo before−matching (C−b)
time to autoimmunity
Pembrolizumab
autoimmune−free %
P<0.0001 (matched)
P<0.0001 (unmatched)
P=3e−04 (matched)
P=0.0028 (matched)
10 days HeadAndNeck num. at risk
matched
chemotherapy 462.0 187.3 88.8 43.8 26.5 14.7
Pembrolizumab 462 75 20 5 NA NA
unmatched
chemotherapy 10828 5022 2474 1362 839 471
Pembrolizumab 504 80 23 6 NA NA
30 days HeadAndNeck num. at risk
matched
chemotherapy 433.0 171.6 82.0 41.9 25.1 13.4
Pembrolizumab 433 62 16 5 NA NA
unmatched
chemotherapy 10489 4884 2421 1336 820 456
Pembrolizumab 472 69 19 6 NA NA
60 days HeadAndNeck num. at risk
matched
chemotherapy 401.0 156.8 69.8 36.1 21.4 12.0
Pembrolizumab 401 61 16 5 NA NA
unmatched
chemotherapy 10082 4718 2341 1292 791 439
Pembrolizumab 445 66 19 6 NA NA
90 days HeadAndNeck num. at risk
matched
chemotherapy 393.0 150.8 68.9 33.9 20.0 11.0
Pembrolizumab 393 62 17 6 NA NA
unmatched
chemotherapy 9694 4555 2262 1251 767 429
Pembrolizumab 434 66 19 6 NA NA
180 days HeadAndNeck num. at risk
matched
chemotherapy 366.0 143.5 64.8 34.4 20.2 10.5
Pembrolizumab 366 57 14 5 NA NA
unmatched
chemotherapy 8683 4133 2060 1145 697 390
Pembrolizumab 402 62 17 6 NA NA
Months after therapy initiation
Nivolumab
time to autoimmunity
Nivolumab
10 days HeadAndNeck num. at risk
matched
chemotherapy 356.0 140.4 63.3 32.5 19.8 12.5
Nivolumab 356 57 18 7 1 NA
unmatched
chemotherapy 10828 5022 2474 1362 839 471
Nivolumab 390 68 20 8 1 NA
30 days HeadAndNeck num. at risk
matched
chemotherapy 344.0 133.6 64.0 32.1 19.0 11.8
Nivolumab 344 55 17 7 1 NA
unmatched
chemotherapy 10489 4884 2421 1336 820 456
Nivolumab 371 65 20 8 1 NA
60 days HeadAndNeck num. at risk
matched
chemotherapy 330.0 126.6 60.0 30.6 17.5 11.0
Nivolumab 330 55 17 7 1 NA
unmatched
chemotherapy 10082 4718 2341 1292 791 439
Nivolumab 356 63 20 8 1 NA
90 days HeadAndNeck num. at risk
matched
chemotherapy 324.0 125.8 58.3 28.2 16.4 9.1
Nivolumab 324 54 16 7 1 NA
unmatched
chemotherapy 9694 4555 2262 1251 767 429
Nivolumab 349 62 19 8 1 NA
180 days HeadAndNeck num. at risk
matched
chemotherapy 307.0 113.1 50.5 23.2 12.5 5.6
Nivolumab 307 54 15 5 1 NA
unmatched
chemotherapy 8683 4133 2060 1145 697 390
Nivolumab 328 61 18 7 1 NA
all immunotherapy drugs
time to autoimmunity
all immunotherapy drugs
10 days HeadAndNeck num. at risk
matched
chemotherapy 872.0 352.2 162.2 82.4 50.1 29.2
all immuno 872 146 40 13 1 NA
unmatched
chemotherapy 10828 5022 2474 1362 839 471
all immuno 952 163 44 14 1 NA
30 days HeadAndNeck num. at risk
matched
chemotherapy 824.0 326.1 154.6 78.3 45.7 26.5
all immuno 824 129 35 12 1 NA
unmatched
chemotherapy 10489 4884 2421 1336 820 456
all immuno 901 149 40 14 1 NA
60 days HeadAndNeck num. at risk
matched
chemotherapy 787.0 304.8 142.6 72.1 43.4 24.7
all immuno 787 126 34 12 1 NA
unmatched
chemotherapy 10082 4718 2341 1292 791 439
all immuno 857 144 40 14 1 NA
90 days HeadAndNeck num. at risk
matched
chemotherapy 765.0 290.6 137.1 68.4 39.7 22.7
all immuno 765 129 34 13 1 NA
unmatched
chemotherapy 9694 4555 2262 1251 767 429
all immuno 839 143 39 14 1 NA
180 days HeadAndNeck num. at risk
matched
chemotherapy 715.0 276.5 126.1 65.4 35.6 18.0
all immuno 715 120 30 11 1 NA
unmatched
chemotherapy 8683 4133 2060 1145 697 390
all immuno 784 138 36 13 1 NA
Months after therapy initiation

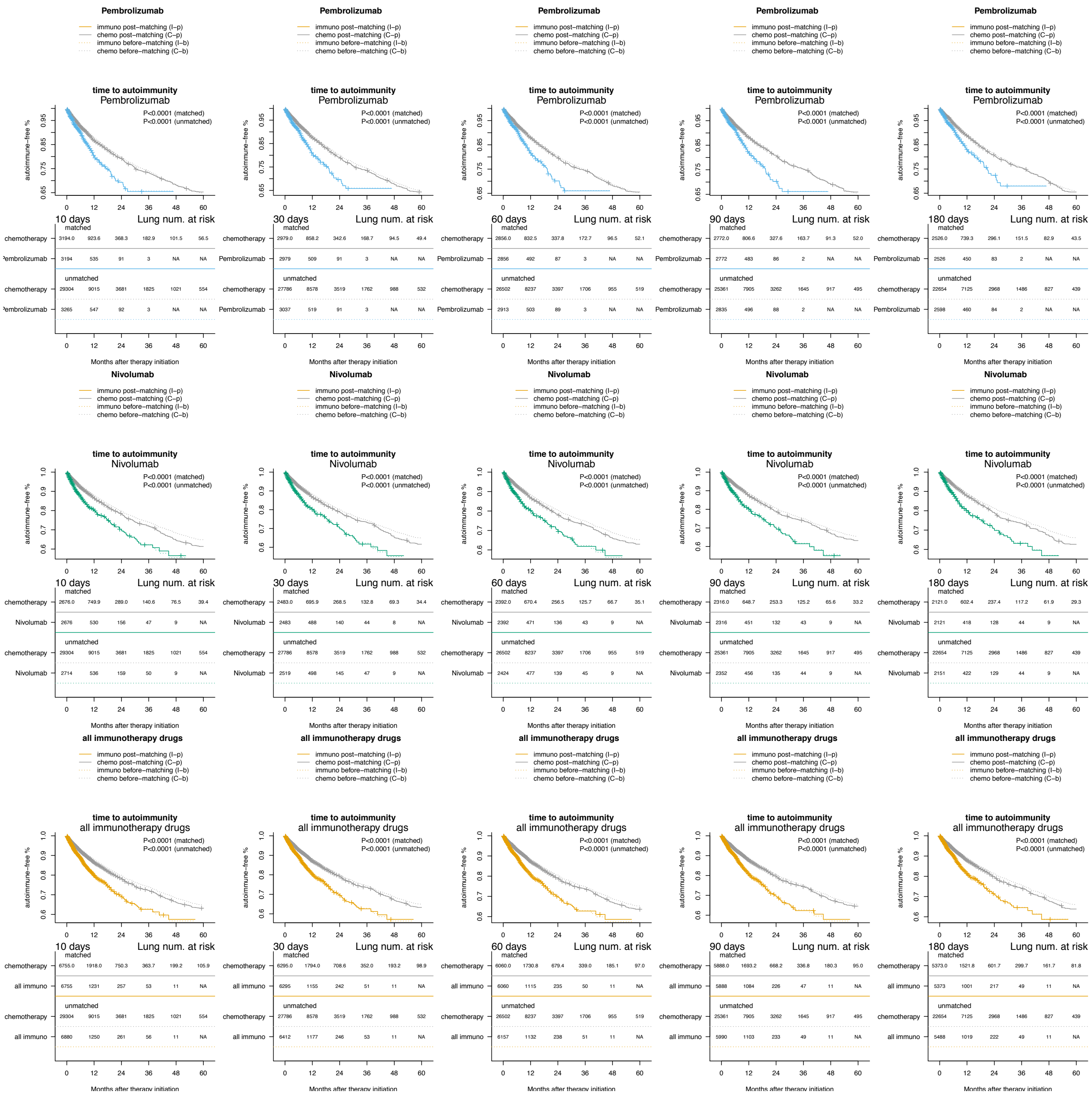
Pembrolizumab
immuno post−matching (I−p)
chemo post−matching (C−p)
immuno before−matching (I−b)
chemo before−matching (C−b)
time to autoimmunity
Pembrolizumab
P<0.0001 (matched)
P<0.0001 (unmatched)
autoimmune−free %
10 days
30 days
60 days
90 days
180 days
Lung num. at risk
matched
unmatched
chemotherapy
Pembrolizumab
Months after therapy initiation
Nivolumab
time to autoimmunity
Nivolumab
all immunotherapy drugs
time to autoimmunity
all immunotherapy drugs
all immuno

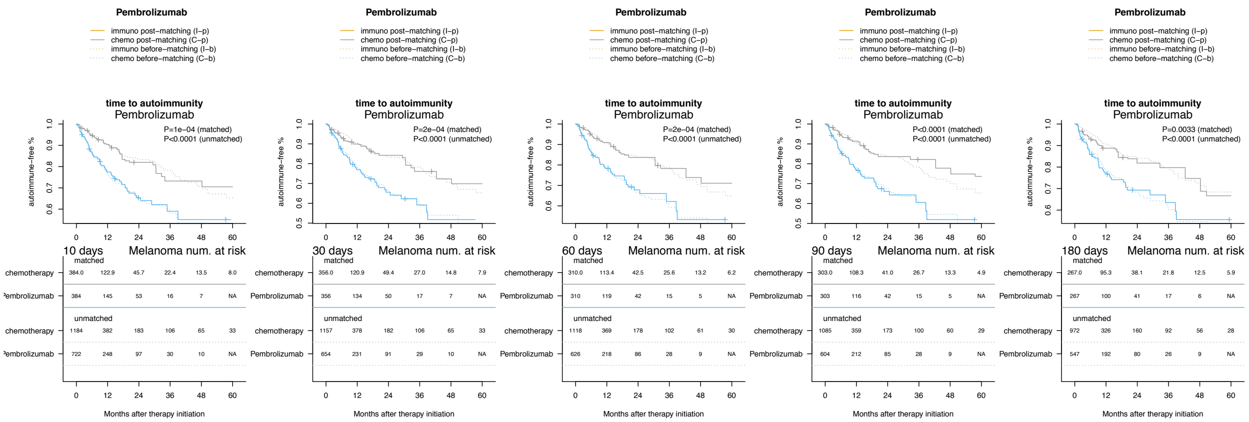

Pembrolizumab
immuno post−matching (I−p)
chemo post−matching (C−p)
immuno before−matching (I−b)
chemo before−matching (C−b)
time to autoimmunity
Pembrolizumab
autoimmune−free %
10 days Melanoma num. at risk
P=1e−04 (matched)
P<0.0001 (unmatched)
matched
chemotherapy 384.0 122.9 45.7 22.4 13.5 8.0
Pembrolizumab 384 145 53 16 7 NA
unmatched
chemotherapy 1184 382 183 106 65 33
Pembrolizumab 722 248 97 30 10 NA
30 days Melanoma num. at risk
P=2e−04 (matched)
P<0.0001 (unmatched)
matched
chemotherapy 356.0 120.9 49.4 27.0 14.8 7.9
Pembrolizumab 356 134 50 17 7 NA
unmatched
chemotherapy 1157 378 182 106 65 33
Pembrolizumab 654 231 91 29 10 NA
60 days Melanoma num. at risk
P=2e−04 (matched)
P<0.0001 (unmatched)
matched
chemotherapy 310.0 113.4 42.5 25.6 13.2 6.2
Pembrolizumab 310 119 42 15 5 NA
unmatched
chemotherapy 1118 369 178 102 61 30
Pembrolizumab 626 218 86 28 9 NA
90 days Melanoma num. at risk
P<0.0001 (matched)
P<0.0001 (unmatched)
matched
chemotherapy 303.0 108.3 41.0 26.7 13.3 4.9
Pembrolizumab 303 116 42 15 5 NA
unmatched
chemotherapy 1085 359 173 100 60 29
Pembrolizumab 604 212 85 28 9 NA
180 days Melanoma num. at risk
P=0.0033 (matched)
P<0.0001 (unmatched)
matched
chemotherapy 267.0 95.3 38.1 21.8 12.5 5.9
Pembrolizumab 267 100 41 17 6 NA
unmatched
chemotherapy 972 326 160 92 56 28
Pembrolizumab 547 192 80 26 9 NA
0 12 24 36 48 60
Months after therapy initiation

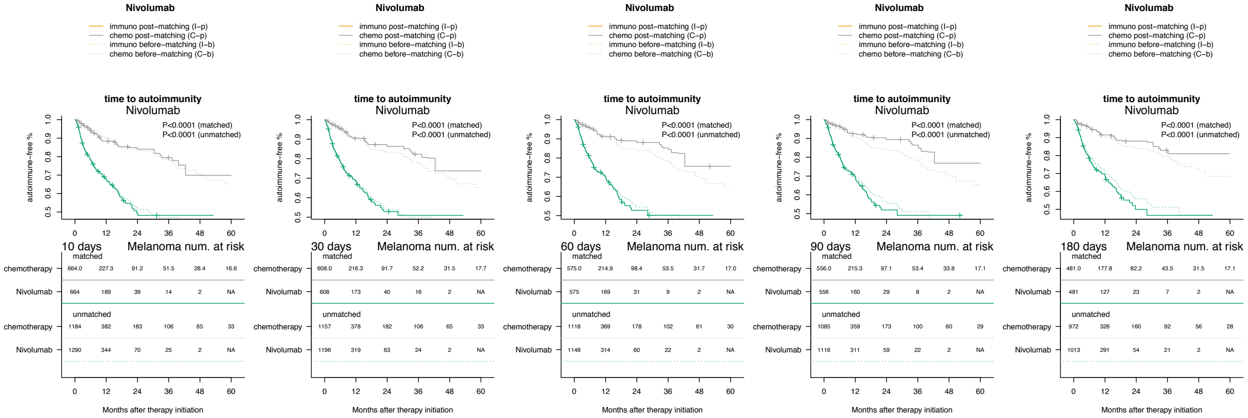

Nivolumab
immuno post−matching (I−p)
chemo post−matching (C−p)
immuno before−matching (I−b)
chemo before−matching (C−b)
time to autoimmunity
Nivolumab
autoimmune−free %
P<0.0001 (matched)
P<0.0001 (unmatched)
10 days Melanoma num. at risk
matched
chemotherapy 664.0 227.3 91.2 51.5 28.4 16.6
Nivolumab 664 189 39 14 2 NA
unmatched
chemotherapy 1184 382 183 106 65 33
Nivolumab 1290 344 70 25 2 NA
30 days Melanoma num. at risk
matched
chemotherapy 608.0 216.3 91.7 52.2 31.5 17.7
Nivolumab 608 173 40 16 2 NA
unmatched
chemotherapy 1157 378 182 106 65 33
Nivolumab 1196 319 63 24 2 NA
60 days Melanoma num. at risk
matched
chemotherapy 575.0 214.9 98.4 53.5 31.7 17.0
Nivolumab 575 169 31 9 2 NA
unmatched
chemotherapy 1118 369 178 102 61 30
Nivolumab 1148 314 60 22 2 NA
90 days Melanoma num. at risk
matched
chemotherapy 556.0 215.3 97.1 53.4 33.8 17.1
Nivolumab 556 160 29 8 2 NA
unmatched
chemotherapy 1085 359 173 100 60 29
Nivolumab 1116 311 59 22 2 NA
180 days Melanoma num. at risk
matched
chemotherapy 481.0 177.8 82.2 43.5 31.5 17.1
Nivolumab 481 127 23 7 2 NA
unmatched
chemotherapy 972 326 160 92 56 28
Nivolumab 1013 291 54 21 2 NA
0 12 24 36 48 60
Months after therapy initiation

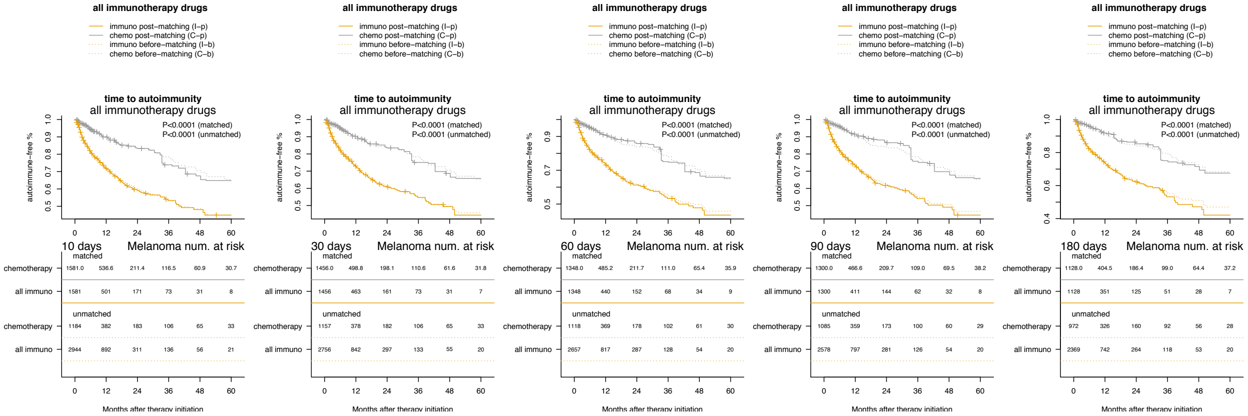

all immunotherapy drugs
immuno post−matching (I−p)
chemo post−matching (C−p)
immuno before−matching (I−b)
chemo before−matching (C−b)
time to autoimmunity
all immunotherapy drugs
autoimmune−free %
P<0.0001 (matched)
P<0.0001 (unmatched)
10 days Melanoma num. at risk
matched
chemotherapy 1581.0 536.6 211.4 116.5 60.9 30.7
all immuno 1581 501 171 73 31 8
unmatched
chemotherapy 1184 382 183 106 65 33
all immuno 2944 892 311 136 56 21
30 days Melanoma num. at risk
matched
chemotherapy 1456.0 498.8 198.1 110.6 61.6 31.8
all immuno 1456 463 161 73 31 7
unmatched
chemotherapy 1157 378 182 106 65 33
all immuno 2756 842 297 133 55 20
60 days Melanoma num. at risk
matched
chemotherapy 1348.0 485.2 211.7 111.0 65.4 35.9
all immuno 1348 440 152 68 34 9
unmatched
chemotherapy 1118 369 178 102 61 30
all immuno 2657 817 287 128 54 20
90 days Melanoma num. at risk
matched
chemotherapy 1300.0 466.6 209.7 109.0 69.5 38.2
all immuno 1300 411 144 62 32 8
unmatched
chemotherapy 1085 359 173 100 60 29
all immuno 2578 797 281 126 54 20
180 days Melanoma num. at risk
matched
chemotherapy 1128.0 404.5 186.4 99.0 64.4 37.2
all immuno 1128 351 125 51 28 7
unmatched
chemotherapy 972 326 160 92 56 28
all immuno 2369 742 264 118 53 20
0 12 24 36 48 60
Months after therapy initiation

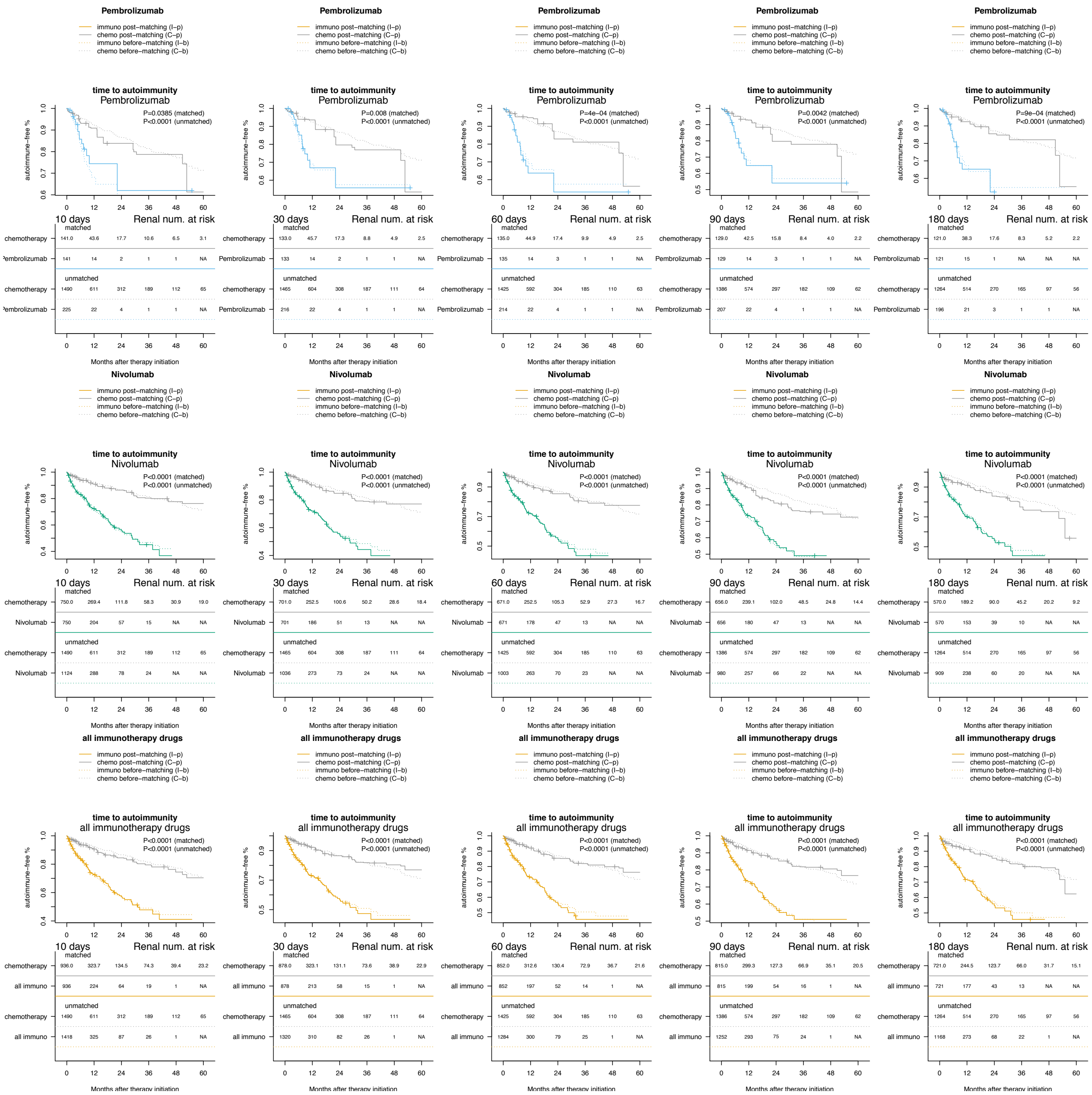
Pembrolizumab
immuno post–matching (I–p)
chemo post–matching (C–p)
immuno before–matching (I–b)
chemo before–matching (C–b)
time to autoimmunity
Pembrolizumab
autoimmune–free %
Months after therapy initiation
Renal num. at risk
matched
unmatched
chemotherapy
Pembrolizumab
10 days
P=0.0385 (matched)
P<0.0001 (unmatched)
141.0 43.6 17.7 10.6 6.5 3.1
141 14 2 1 1 NA
1490 611 312 189 112 65
225 22 4 1 1 NA
30 days
P=0.008 (matched)
P<0.0001 (unmatched)
133.0 45.7 17.3 8.8 4.9 2.5
133 14 2 1 1 NA
1465 604 308 187 111 64
216 22 4 1 1 NA
60 days
P=4e–04 (matched)
P<0.0001 (unmatched)
135.0 44.9 17.4 9.9 4.9 2.5
135 14 3 1 1 NA
1425 592 304 185 110 63
214 22 4 1 1 NA
90 days
P=0.0042 (matched)
P<0.0001 (unmatched)
129.0 42.5 15.8 8.4 4.0 2.2
129 14 3 1 1 NA
1386 574 297 182 109 62
207 22 4 1 1 NA
180 days
P=9e–04 (matched)
P<0.0001 (unmatched)
121.0 38.3 17.6 8.3 5.2 2.2
121 15 1 NA NA NA
1264 514 270 165 97 56
196 21 3 1 1 NA
Nivolumab
time to autoimmunity
Nivolumab
10 days
P<0.0001 (matched)
P<0.0001 (unmatched)
750.0 269.4 111.8 58.3 30.9 19.0
750 204 57 15 NA NA
1490 611 312 189 112 65
1124 288 78 24 NA NA
30 days
701.0 252.5 100.6 50.2 28.6 18.4
701 186 51 13 NA NA
1465 604 308 187 111 64
1036 273 73 24 NA NA
60 days
671.0 252.5 105.3 52.9 27.3 16.7
671 178 47 13 NA NA
1425 592 304 185 110 63
1003 263 70 23 NA NA
90 days
656.0 239.1 102.0 48.5 24.8 14.4
656 180 47 13 NA NA
1386 574 297 182 109 62
980 257 66 22 NA NA
180 days
570.0 189.2 90.0 45.2 20.2 9.2
570 153 39 10 NA NA
1264 514 270 165 97 56
909 238 60 20 NA NA
all immunotherapy drugs
time to autoimmunity
all immunotherapy drugs
all immuno
10 days
P<0.0001 (matched)
P<0.0001 (unmatched)
936.0 323.7 134.5 74.3 39.4 23.2
936 224 64 19 1 NA
1490 611 312 189 112 65
1418 325 87 26 1 NA
30 days
878.0 323.1 131.1 73.6 38.9 22.9
878 213 58 15 1 NA
1465 604 308 187 111 64
1320 310 82 26 1 NA
60 days
852.0 312.6 130.4 72.9 36.7 21.6
852 197 52 14 1 NA
1425 592 304 185 110 63
1284 300 79 25 1 NA
90 days
815.0 299.3 127.3 66.9 35.1 20.5
815 199 54 16 1 NA
1386 574 297 182 109 62
1252 293 75 24 1 NA
180 days
721.0 244.5 123.7 66.0 31.7 15.1
721 177 43 13 NA NA
1264 514 270 165 97 56
1168 273 68 22 1 NA

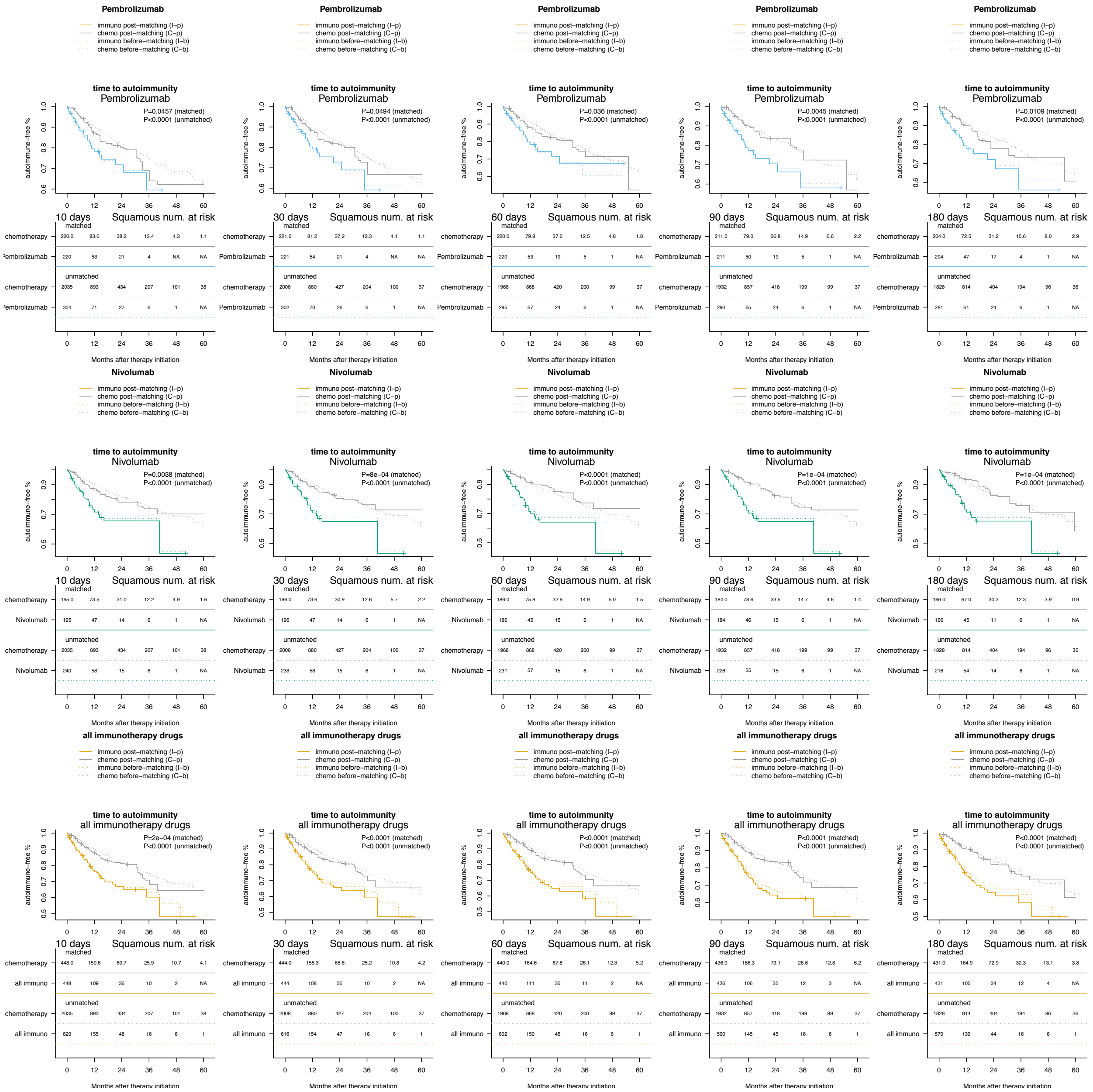
Pembrolizumab
immuno post−matching (I−p)
chemo post−matching (C−p)
immuno before−matching (I−b)
chemo before−matching (C−b)
time to autoimmunity
Pembrolizumab
autoimmune−free %
P=0.0457 (matched)
P<0.0001 (unmatched)
P=0.0494 (matched)
P<0.0001 (unmatched)
P=0.036 (matched)
P<0.0001 (unmatched)
P=0.0045 (matched)
P<0.0001 (unmatched)
P=0.0109 (matched)
P<0.0001 (unmatched)
10 days Squamous num. at risk
30 days Squamous num. at risk
60 days Squamous num. at risk
90 days Squamous num. at risk
180 days Squamous num. at risk
matched
chemotherapy 220.0 83.6 38.2 13.4 4.3 1.1
Pembrolizumab 220 53 21 4 NA NA
unmatched
chemotherapy 2035 893 434 207 101 38
Pembrolizumab 304 71 27 6 1 NA
chemotherapy 221.0 81.2 37.2 12.3 4.1 1.1
Pembrolizumab 221 54 21 4 NA NA
chemotherapy 2008 880 427 204 100 37
Pembrolizumab 302 70 26 6 1 NA
chemotherapy 220.0 79.8 37.0 12.5 4.8 1.8
Pembrolizumab 220 53 19 5 1 NA
chemotherapy 1968 868 420 200 99 37
Pembrolizumab 295 67 24 6 1 NA
chemotherapy 211.0 79.0 36.8 14.9 6.6 2.2
Pembrolizumab 211 50 19 5 1 NA
chemotherapy 1932 857 418 199 99 37
Pembrolizumab 290 65 24 6 1 NA
chemotherapy 204.0 72.5 31.2 15.6 8.0 2.9
Pembrolizumab 204 47 17 4 1 NA
chemotherapy 1828 814 404 194 96 36
Pembrolizumab 281 61 24 6 1 NA
Months after therapy initiation
Nivolumab
time to autoimmunity
Nivolumab
P=0.0038 (matched)
P<0.0001 (unmatched)
P=8e−04 (matched)
P<0.0001 (unmatched)
P<0.0001 (matched)
P<0.0001 (unmatched)
P=1e−04 (matched)
P<0.0001 (unmatched)
P=1e−04 (matched)
P<0.0001 (unmatched)
chemotherapy 195.0 73.5 31.0 12.2 4.9 1.6
Nivolumab 195 47 14 6 1 NA
chemotherapy 2035 893 434 207 101 38
Nivolumab 240 58 15 6 1 NA
chemotherapy 196.0 73.6 30.9 12.6 5.7 2.2
Nivolumab 196 47 14 6 1 NA
chemotherapy 2008 880 427 204 100 37
Nivolumab 238 58 15 6 1 NA
chemotherapy 186.0 75.8 32.9 14.9 5.0 1.5
Nivolumab 186 45 15 6 1 NA
chemotherapy 1968 868 420 200 99 37
Nivolumab 231 57 15 6 1 NA
chemotherapy 184.0 78.6 33.5 14.7 4.6 1.4
Nivolumab 184 46 15 6 1 NA
chemotherapy 1932 857 418 199 99 37
Nivolumab 226 55 15 6 1 NA
chemotherapy 166.0 67.0 30.3 12.3 3.9 0.9
Nivolumab 166 45 11 6 1 NA
chemotherapy 1828 814 404 194 96 36
Nivolumab 216 54 14 6 1 NA
all immunotherapy drugs
time to autoimmunity
all immunotherapy drugs
P=2e−04 (matched)
P<0.0001 (unmatched)
P<0.0001 (matched)
P<0.0001 (unmatched)
chemotherapy 448.0 159.6 69.7 25.9 10.7 4.1
all immuno 448 109 36 10 2 NA
chemotherapy 2035 893 434 207 101 38
all immuno 620 155 48 16 6 1
chemotherapy 444.0 155.3 65.6 25.2 10.8 4.2
all immuno 444 108 35 10 2 NA
chemotherapy 2008 880 427 204 100 37
all immuno 616 154 47 16 6 1
chemotherapy 440.0 164.6 67.8 26.1 12.3 5.2
all immuno 440 111 35 11 2 NA
chemotherapy 1968 868 420 200 99 37
all immuno 602 150 45 16 6 1
chemotherapy 436.0 166.3 73.1 28.6 12.8 6.2
all immuno 436 106 35 12 3 NA
chemotherapy 1932 857 418 199 99 37
all immuno 590 145 45 16 6 1
chemotherapy 431.0 164.9 72.9 32.3 13.1 3.8
all immuno 431 105 34 12 4 NA
chemotherapy 1828 814 404 194 96 36
all immuno 570 139 44 16 6 1
Months after therapy initiation

Targeted therapy

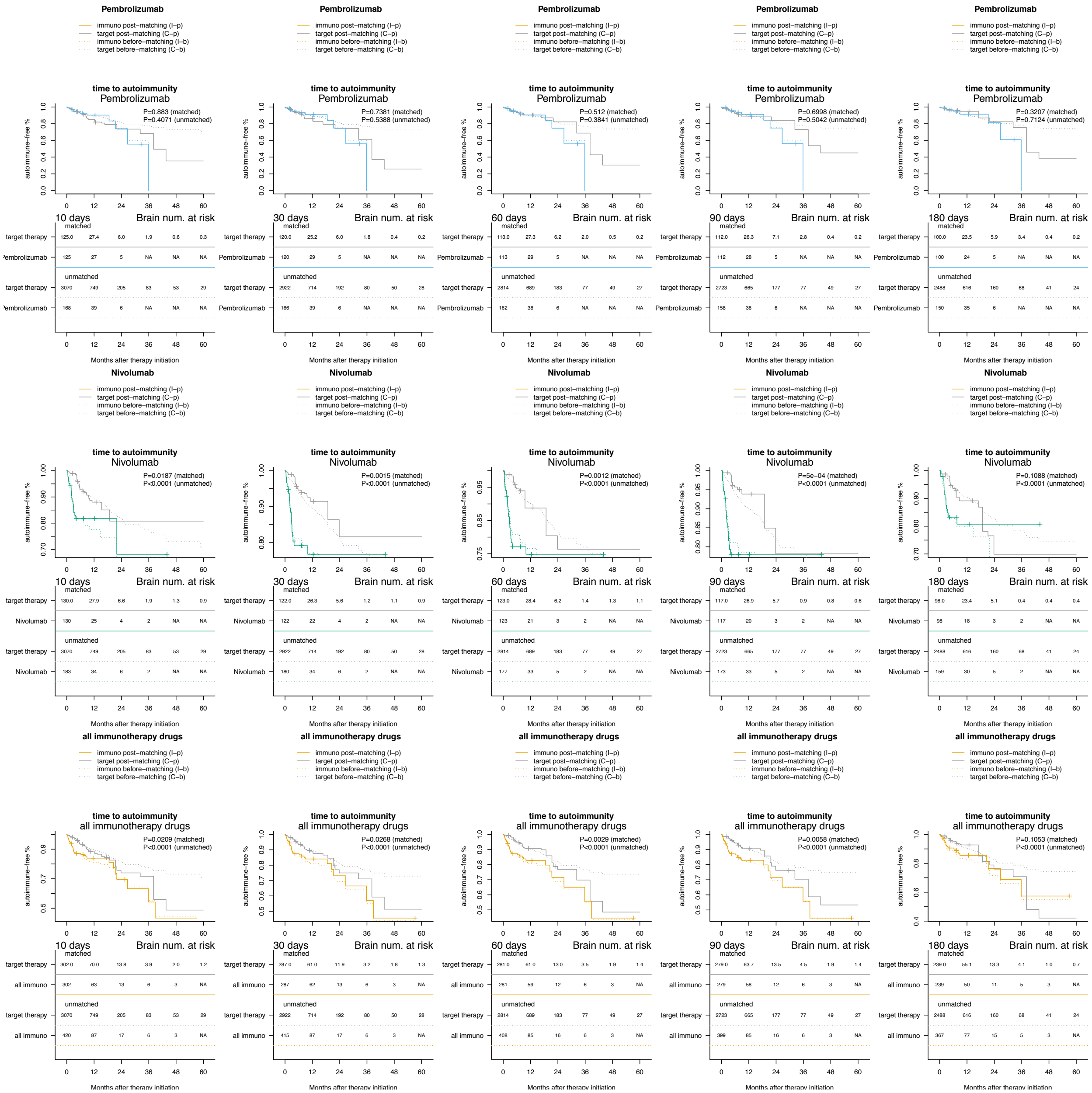

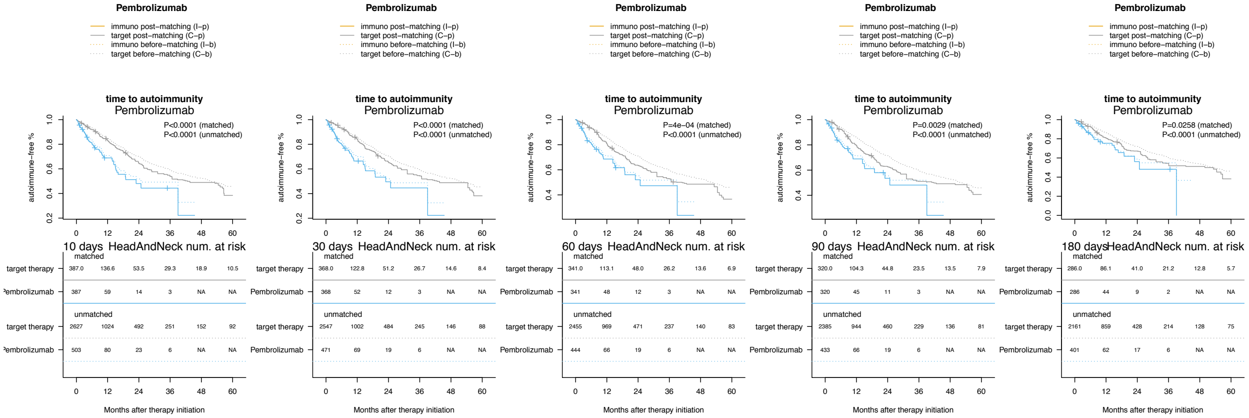

Pembrolizumab
immuno post−matching (I−p)
target post−matching (C−p)
immuno before−matching (I−b)
target before−matching (C−b)
time to autoimmunity
Pembrolizumab
autoimmune−free %
P<0.0001 (matched)
P<0.0001 (unmatched)
P=4e−04 (matched)
P<0.0001 (unmatched)
P=0.0029 (matched)
P<0.0001 (unmatched)
P=0.0258 (matched)
P<0.0001 (unmatched)
10 days HeadAndNeck num. at risk
30 days HeadAndNeck num. at risk
60 days HeadAndNeck num. at risk
90 days HeadAndNeck num. at risk
180 days HeadAndNeck num. at risk
matched
target therapy 387.0 136.6 53.5 29.3 18.9 10.5
Pembrolizumab 387 59 14 3 NA NA
unmatched
target therapy 2627 1024 492 251 152 92
Pembrolizumab 503 80 23 6 NA NA
target therapy 368.0 122.8 51.2 26.7 14.6 8.4
Pembrolizumab 368 52 12 3 NA NA
target therapy 2547 1002 484 245 146 88
Pembrolizumab 471 69 19 6 NA NA
target therapy 341.0 113.1 48.0 26.2 13.6 6.9
Pembrolizumab 341 48 12 3 NA NA
target therapy 2455 969 471 237 140 83
Pembrolizumab 444 66 19 6 NA NA
target therapy 320.0 104.3 44.8 23.5 13.5 7.9
Pembrolizumab 320 45 11 3 NA NA
target therapy 2385 944 460 229 136 81
Pembrolizumab 433 66 19 6 NA NA
target therapy 286.0 86.1 41.0 21.2 12.8 5.7
Pembrolizumab 286 44 9 2 NA NA
target therapy 2161 859 428 214 128 75
Pembrolizumab 401 62 17 6 NA NA
Months after therapy initiation

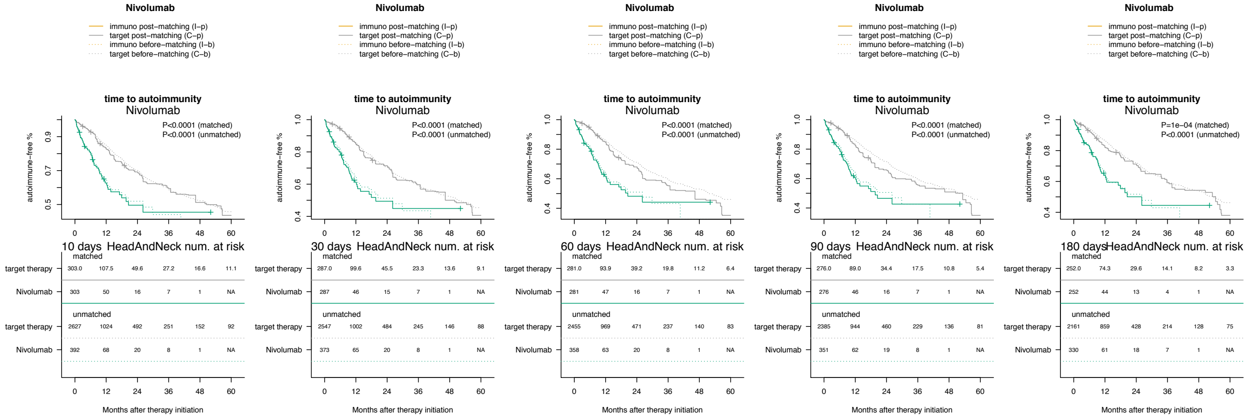

Nivolumab
immuno post−matching (I−p)
target post−matching (C−p)
immuno before−matching (I−b)
target before−matching (C−b)
time to autoimmunity
Nivolumab
autoimmune−free %
P<0.0001 (matched)
P<0.0001 (unmatched)
P=1e−04 (matched)
P<0.0001 (unmatched)
10 days HeadAndNeck num. at risk
30 days HeadAndNeck num. at risk
60 days HeadAndNeck num. at risk
90 days HeadAndNeck num. at risk
180 days HeadAndNeck num. at risk
matched
target therapy 303.0 107.5 49.6 27.2 16.6 11.1
Nivolumab 303 50 16 7 1 NA
unmatched
target therapy 2627 1024 492 251 152 92
Nivolumab 392 68 20 8 1 NA
target therapy 287.0 99.6 45.5 23.3 13.6 9.1
Nivolumab 287 46 15 7 1 NA
target therapy 2547 1002 484 245 146 88
Nivolumab 373 65 20 8 1 NA
target therapy 281.0 93.9 39.2 19.8 11.2 6.4
Nivolumab 281 47 16 7 1 NA
target therapy 2455 969 471 237 140 83
Nivolumab 358 63 20 8 1 NA
target therapy 276.0 89.0 34.4 17.5 10.8 5.4
Nivolumab 276 46 16 7 1 NA
target therapy 2385 944 460 229 136 81
Nivolumab 351 62 19 8 1 NA
target therapy 252.0 74.3 29.6 14.1 8.2 3.3
Nivolumab 252 44 13 4 1 NA
target therapy 2161 859 428 214 128 75
Nivolumab 330 61 18 7 1 NA
Months after therapy initiation

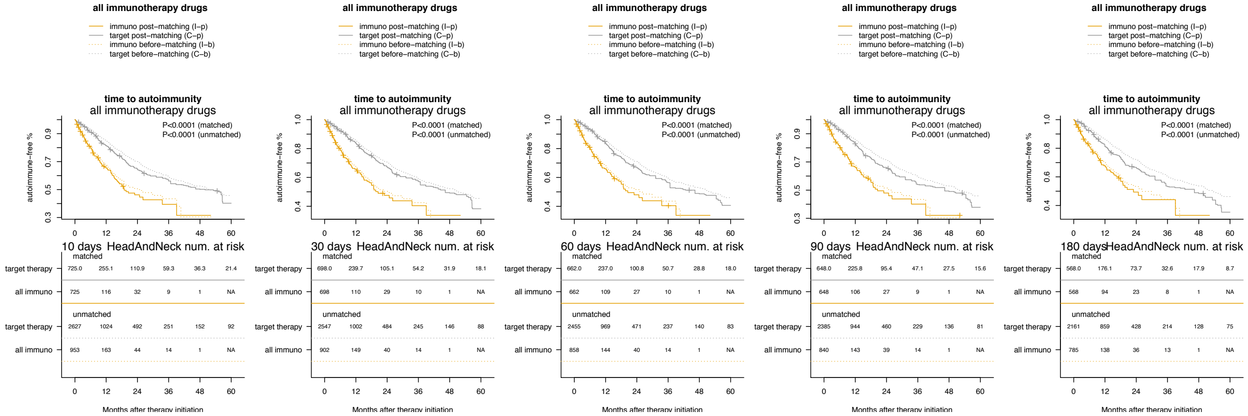

all immunotherapy drugs
immuno post−matching (I−p)
target post−matching (C−p)
immuno before−matching (I−b)
target before−matching (C−b)
time to autoimmunity
all immunotherapy drugs
autoimmune−free %
P<0.0001 (matched)
P<0.0001 (unmatched)
10 days HeadAndNeck num. at risk
30 days HeadAndNeck num. at risk
60 days HeadAndNeck num. at risk
90 days HeadAndNeck num. at risk
180 days HeadAndNeck num. at risk
matched
target therapy 725.0 255.1 110.9 59.3 36.3 21.4
all immuno 725 116 32 9 1 NA
unmatched
target therapy 2627 1024 492 251 152 92
all immuno 953 163 44 14 1 NA
target therapy 698.0 239.7 105.1 54.2 31.9 18.1
all immuno 698 110 29 10 1 NA
target therapy 2547 1002 484 245 146 88
all immuno 902 149 40 14 1 NA
target therapy 662.0 237.0 100.8 50.7 28.8 18.0
all immuno 662 109 27 10 1 NA
target therapy 2455 969 471 237 140 83
all immuno 858 144 40 14 1 NA
target therapy 648.0 225.8 95.4 47.1 27.5 15.6
all immuno 648 106 27 9 1 NA
target therapy 2385 944 460 229 136 81
all immuno 840 143 39 14 1 NA
target therapy 568.0 176.1 73.7 32.6 17.9 8.7
all immuno 568 94 23 8 1 NA
target therapy 2161 859 428 214 128 75
all immuno 785 138 36 13 1 NA
Months after therapy initiation

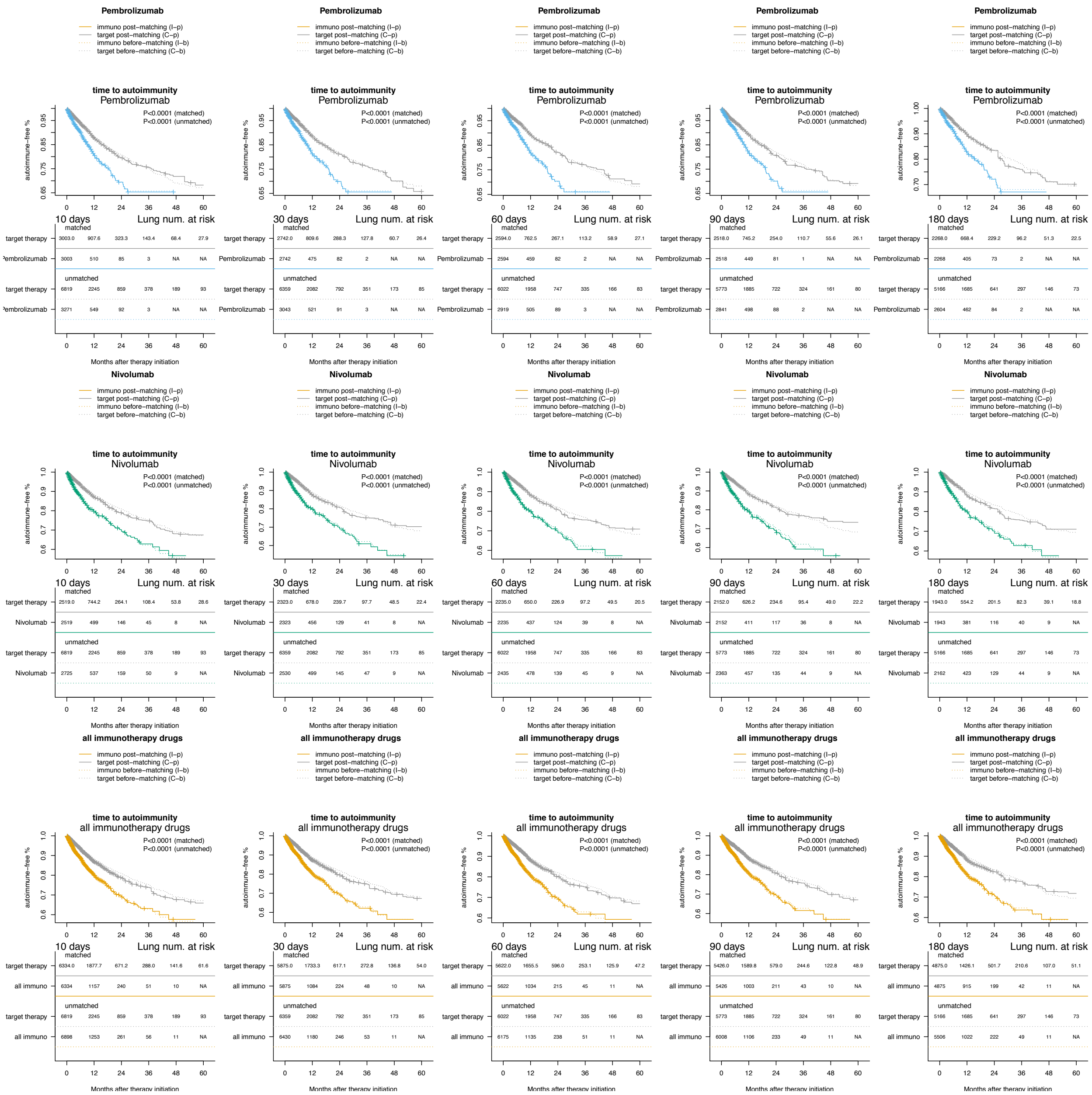
Pembrolizumab
immuno post–matching (I–p)
target post–matching (C–p)
immuno before–matching (I–b)
target before–matching (C–b)
time to autoimmunity
Pembrolizumab
P<0.0001 (matched)
P<0.0001 (unmatched)
autoimmune–free %
10 days Lung num. at risk
matched
target therapy 3003.0 907.6 323.3 143.4 68.4 27.9
Pembrolizumab 3003 510 85 3 NA NA
unmatched
target therapy 6819 2245 859 378 189 93
Pembrolizumab 3271 549 92 3 NA NA
Months after therapy initiation
30 days Lung num. at risk
matched
target therapy 2742.0 809.6 288.3 127.8 60.7 26.4
Pembrolizumab 2742 475 82 2 NA NA
unmatched
target therapy 6359 2082 792 351 173 85
Pembrolizumab 3043 521 91 3 NA NA
60 days Lung num. at risk
matched
target therapy 2594.0 762.5 267.1 113.2 58.9 27.1
Pembrolizumab 2594 459 82 2 NA NA
unmatched
target therapy 6022 1958 747 335 166 83
Pembrolizumab 2919 505 89 3 NA NA
90 days Lung num. at risk
matched
target therapy 2518.0 745.2 254.0 110.7 55.6 26.1
Pembrolizumab 2518 449 81 1 NA NA
unmatched
target therapy 5773 1885 722 324 161 80
Pembrolizumab 2841 498 88 2 NA NA
180 days Lung num. at risk
matched
target therapy 2268.0 668.4 229.2 96.2 51.3 22.5
Pembrolizumab 2268 405 73 2 NA NA
unmatched
target therapy 5166 1685 641 297 146 73
Pembrolizumab 2604 462 84 2 NA NA
Nivolumab
time to autoimmunity
Nivolumab
10 days Lung num. at risk
matched
target therapy 2519.0 744.2 264.1 108.4 53.8 28.6
Nivolumab 2519 499 146 45 8 NA
unmatched
target therapy 6819 2245 859 378 189 93
Nivolumab 2725 537 159 50 9 NA
30 days Lung num. at risk
matched
target therapy 2323.0 678.0 239.7 97.7 48.5 22.4
Nivolumab 2323 456 129 41 8 NA
unmatched
target therapy 6359 2082 792 351 173 85
Nivolumab 2530 499 145 47 9 NA
60 days Lung num. at risk
matched
target therapy 2235.0 650.0 226.9 97.2 49.5 20.5
Nivolumab 2235 437 124 39 8 NA
unmatched
target therapy 6022 1958 747 335 166 83
Nivolumab 2435 478 139 45 9 NA
90 days Lung num. at risk
matched
target therapy 2152.0 626.2 234.6 95.4 49.0 22.2
Nivolumab 2152 411 117 36 8 NA
unmatched
target therapy 5773 1885 722 324 161 80
Nivolumab 2363 457 135 44 9 NA
180 days Lung num. at risk
matched
target therapy 1943.0 554.2 201.5 82.3 39.1 18.8
Nivolumab 1943 381 116 40 9 NA
unmatched
target therapy 5166 1685 641 297 146 73
Nivolumab 2162 423 129 44 9 NA
all immunotherapy drugs
time to autoimmunity
all immunotherapy drugs
10 days Lung num. at risk
matched
target therapy 6334.0 1877.7 671.2 288.0 141.6 61.6
all immuno 6334 1157 240 51 10 NA
unmatched
target therapy 6819 2245 859 378 189 93
all immuno 6898 1253 261 56 11 NA
30 days Lung num. at risk
matched
target therapy 5875.0 1733.3 617.1 272.8 136.8 54.0
all immuno 5875 1084 224 48 10 NA
unmatched
target therapy 6359 2082 792 351 173 85
all immuno 6430 1180 246 53 11 NA
60 days Lung num. at risk
matched
target therapy 5622.0 1655.5 596.0 253.1 125.9 47.2
all immuno 5622 1034 215 45 11 NA
unmatched
target therapy 6022 1958 747 335 166 83
all immuno 6175 1135 238 51 11 NA
90 days Lung num. at risk
matched
target therapy 5426.0 1589.8 579.0 244.6 122.8 48.9
all immuno 5426 1003 211 43 10 NA
unmatched
target therapy 5773 1885 722 324 161 80
all immuno 6008 1106 233 49 11 NA
180 days Lung num. at risk
matched
target therapy 4875.0 1426.1 501.7 210.6 107.0 51.1
all immuno 4875 915 199 42 11 NA
unmatched
target therapy 5166 1685 641 297 146 73
all immuno 5506 1022 222 49 11 NA

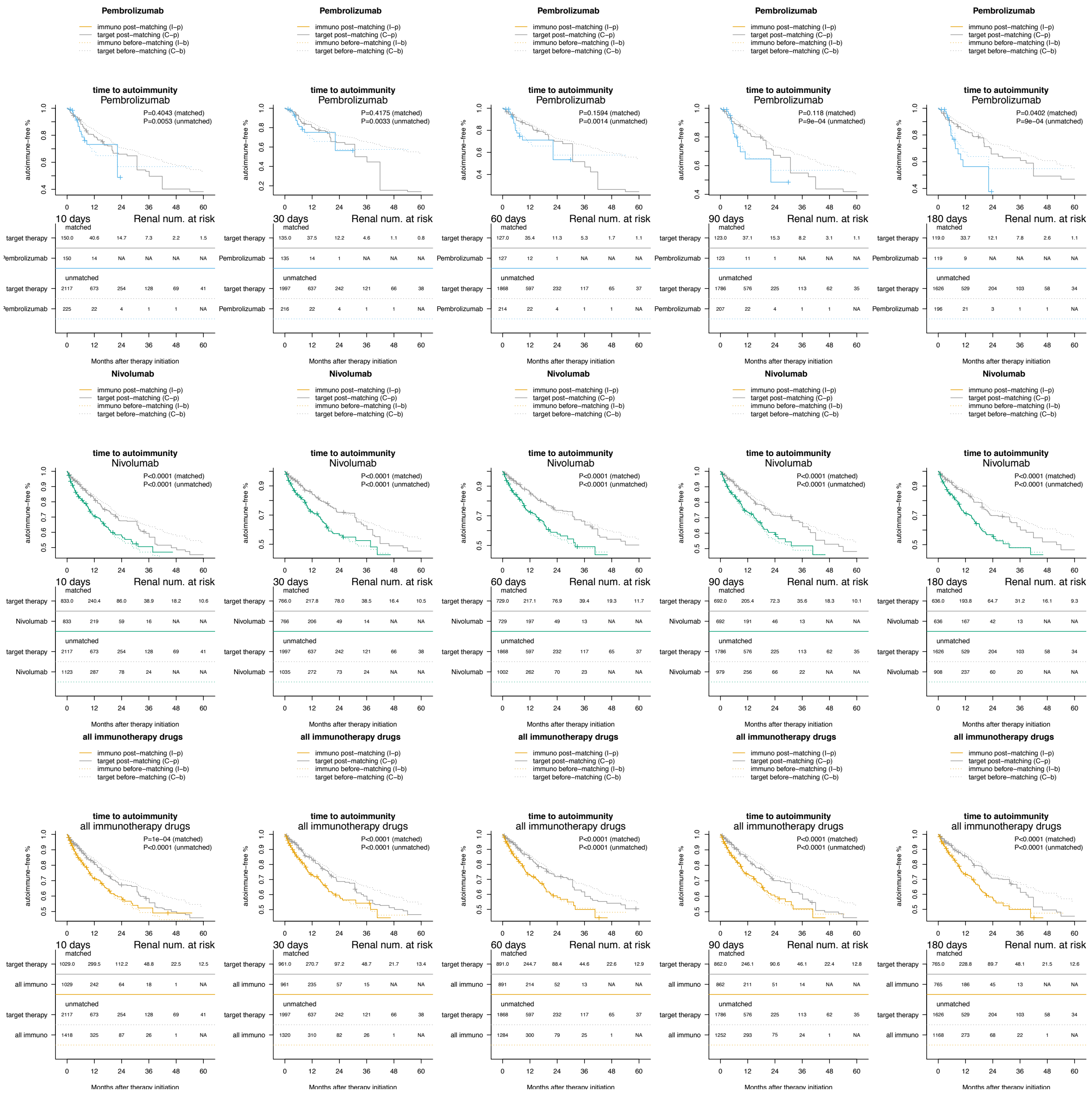
Pembrolizumab
immuno post−matching (I−p)
target post−matching (C−p)
immuno before−matching (I−b)
target before−matching (C−b)
time to autoimmunity
Pembrolizumab
P=0.4043 (matched)
P=0.0053 (unmatched)
P=0.4175 (matched)
P=0.0033 (unmatched)
P=0.1594 (matched)
P=0.0014 (unmatched)
P=0.118 (matched)
P=9e−04 (unmatched)
P=0.0402 (matched)
P=9e−04 (unmatched)
autoimmune−free %
10 days
30 days
60 days
90 days
180 days
Renal num. at risk
matched
target therapy 150.0 40.6 14.7 7.3 2.2 1.5
Pembrolizumab 150 14 NA NA NA NA
unmatched
target therapy 2117 673 254 128 69 41
Pembrolizumab 225 22 4 1 1 NA
target therapy 135.0 37.5 12.2 4.6 1.1 0.8
Pembrolizumab 135 14 1 NA NA NA
target therapy 1997 637 242 121 66 38
Pembrolizumab 216 22 4 1 1 NA
target therapy 127.0 35.4 11.3 5.3 1.7 1.1
Pembrolizumab 127 12 1 NA NA NA
target therapy 1868 597 232 117 65 37
Pembrolizumab 214 22 4 1 1 NA
target therapy 123.0 37.1 15.3 8.2 3.1 1.1
Pembrolizumab 123 11 1 NA NA NA
target therapy 1786 576 225 113 62 35
Pembrolizumab 207 22 4 1 1 NA
target therapy 119.0 33.7 12.1 7.8 2.6 1.1
Pembrolizumab 119 9 NA NA NA NA
target therapy 1626 529 204 103 58 34
Pembrolizumab 196 21 3 1 1 NA
Months after therapy initiation
Nivolumab
P<0.0001 (matched)
P<0.0001 (unmatched)
target therapy 833.0 240.4 86.0 38.9 18.2 10.6
Nivolumab 833 219 59 16 NA NA
target therapy 2117 673 254 128 69 41
Nivolumab 1123 287 78 24 NA NA
target therapy 766.0 217.8 78.0 38.5 16.4 10.5
Nivolumab 766 206 49 14 NA NA
target therapy 1997 637 242 121 66 38
Nivolumab 1035 272 73 24 NA NA
target therapy 729.0 217.1 76.9 39.4 19.3 11.7
Nivolumab 729 197 49 13 NA NA
target therapy 1868 597 232 117 65 37
Nivolumab 1002 262 70 23 NA NA
target therapy 692.0 205.4 72.3 35.6 18.3 10.1
Nivolumab 692 191 46 13 NA NA
target therapy 1786 576 225 113 62 35
Nivolumab 979 256 66 22 NA NA
target therapy 636.0 193.8 64.7 31.2 16.1 9.3
Nivolumab 636 167 42 13 NA NA
target therapy 1626 529 204 103 58 34
Nivolumab 908 237 60 20 NA NA
all immunotherapy drugs
P=1e−04 (matched)
P<0.0001 (unmatched)
target therapy 1029.0 299.5 112.2 48.8 22.5 12.5
all immuno 1029 242 64 18 1 NA
target therapy 2117 673 254 128 69 41
all immuno 1418 325 87 26 1 NA
target therapy 961.0 270.7 97.2 48.7 21.7 13.4
all immuno 961 235 57 15 NA NA
target therapy 1997 637 242 121 66 38
all immuno 1320 310 82 26 1 NA
target therapy 891.0 244.7 88.4 44.6 22.6 12.9
all immuno 891 214 52 13 NA NA
target therapy 1868 597 232 117 65 37
all immuno 1284 300 79 25 1 NA
target therapy 862.0 246.1 90.6 46.1 22.4 12.8
all immuno 862 211 51 14 NA NA
target therapy 1786 576 225 113 62 35
all immuno 1252 293 75 24 1 NA
target therapy 765.0 228.8 89.7 48.1 21.5 12.6
all immuno 765 186 45 13 NA NA
target therapy 1626 529 204 103 58 34
all immuno 1168 273 68 22 1 NA

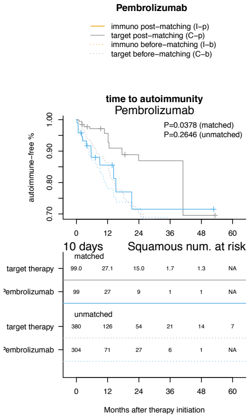
Pembrolizumab
immuno post–matching (I–p)
target post–matching (C–p)
immuno before–matching (I–b)
target before–matching (C–b)
time to autoimmunity
Pembrolizumab
P=0.0378 (matched)
P=0.2646 (unmatched)
autoimmune–free %
10 days
Squamous num. at risk
matched
target therapy 99.0 27.1 15.0 1.7 1.3 NA
Pembrolizumab 99 27 9 1 1 NA
unmatched
target therapy 380 126 54 21 14 7
Pembrolizumab 304 71 27 6 1 NA
Months after therapy initiation

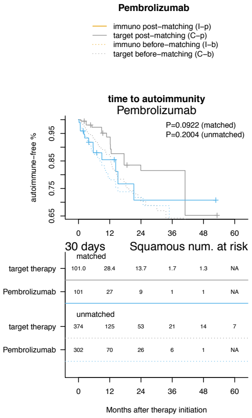
Pembrolizumab
immuno post–matching (I–p)
target post–matching (C–p)
immuno before–matching (I–b)
target before–matching (C–b)
time to autoimmunity
Pembrolizumab
P=0.0922 (matched)
P=0.2004 (unmatched)
autoimmune–free %
30 days
Squamous num. at risk
matched
target therapy 101.0 28.4 13.7 1.7 1.3 NA
Pembrolizumab 101 27 9 1 1 NA
unmatched
target therapy 374 125 53 21 14 7
Pembrolizumab 302 70 26 6 1 NA
Months after therapy initiation

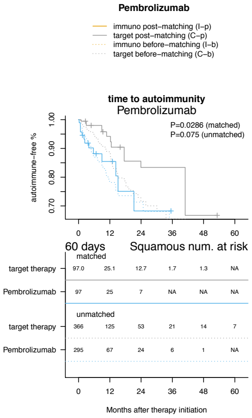
Pembrolizumab
immuno post–matching (I–p)
target post–matching (C–p)
immuno before–matching (I–b)
target before–matching (C–b)
time to autoimmunity
Pembrolizumab
P=0.0286 (matched)
P=0.075 (unmatched)
autoimmune–free %
60 days
Squamous num. at risk
matched
target therapy 97.0 25.1 12.7 1.7 1.3 NA
Pembrolizumab 97 25 7 NA NA NA
unmatched
target therapy 366 125 53 21 14 7
Pembrolizumab 295 67 24 6 1 NA
Months after therapy initiation

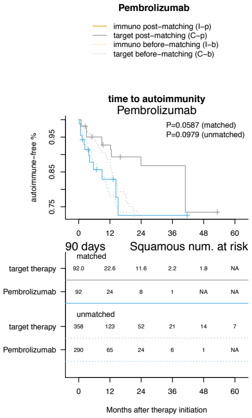
Pembrolizumab
immuno post–matching (I–p)
target post–matching (C–p)
immuno before–matching (I–b)
target before–matching (C–b)
time to autoimmunity
Pembrolizumab
P=0.0587 (matched)
P=0.0979 (unmatched)
autoimmune–free %
90 days
Squamous num. at risk
matched
target therapy 92.0 22.6 11.6 2.2 1.8 NA
Pembrolizumab 92 24 8 1 NA NA
unmatched
target therapy 358 123 52 21 14 7
Pembrolizumab 290 65 24 6 1 NA
Months after therapy initiation

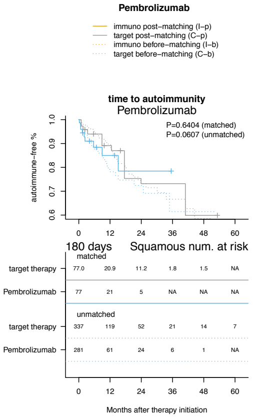
Pembrolizumab
immuno post–matching (I–p)
target post–matching (C–p)
immuno before–matching (I–b)
target before–matching (C–b)
time to autoimmunity
Pembrolizumab
P=0.6404 (matched)
P=0.0607 (unmatched)
autoimmune–free %
180 days
Squamous num. at risk
matched
target therapy 77.0 20.9 11.2 1.8 1.5 NA
Pembrolizumab 77 21 5 NA NA NA
unmatched
target therapy 337 119 52 21 14 7
Pembrolizumab 281 61 24 6 1 NA
Months after therapy initiation

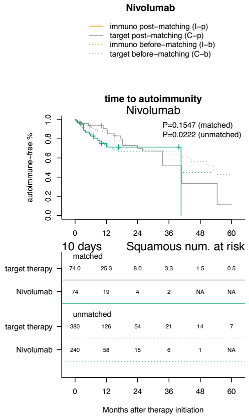
Nivolumab
immuno post–matching (I–p)
target post–matching (C–p)
immuno before–matching (I–b)
target before–matching (C–b)
time to autoimmunity
Nivolumab
P=0.1547 (matched)
P=0.0222 (unmatched)
autoimmune–free %
10 days
Squamous num. at risk
matched
target therapy 74.0 25.3 8.0 3.3 1.5 0.5
Nivolumab 74 19 4 2 NA NA
unmatched
target therapy 380 126 54 21 14 7
Nivolumab 240 58 15 6 1 NA
Months after therapy initiation

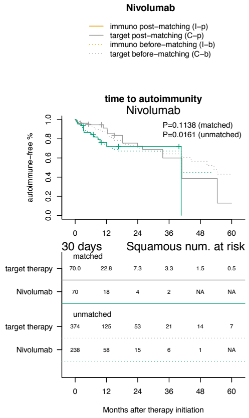
Nivolumab
immuno post–matching (I–p)
target post–matching (C–p)
immuno before–matching (I–b)
target before–matching (C–b)
time to autoimmunity
Nivolumab
P=0.1138 (matched)
P=0.0161 (unmatched)
autoimmune–free %
30 days
Squamous num. at risk
matched
target therapy 70.0 22.8 7.3 3.3 1.5 0.5
Nivolumab 70 18 4 2 NA NA
unmatched
target therapy 374 125 53 21 14 7
Nivolumab 238 58 15 6 1 NA
Months after therapy initiation

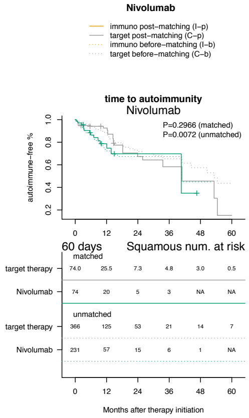
Nivolumab
immuno post–matching (I–p)
target post–matching (C–p)
immuno before–matching (I–b)
target before–matching (C–b)
time to autoimmunity
Nivolumab
P=0.2966 (matched)
P=0.0072 (unmatched)
autoimmune–free %
60 days
Squamous num. at risk
matched
target therapy 74.0 25.5 7.3 4.8 3.0 0.5
Nivolumab 74 20 5 3 NA NA
unmatched
target therapy 366 125 53 21 14 7
Nivolumab 231 57 15 6 1 NA
Months after therapy initiation

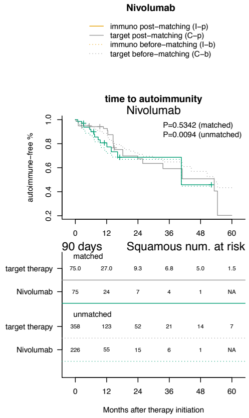
Nivolumab
immuno post–matching (I–p)
target post–matching (C–p)
immuno before–matching (I–b)
target before–matching (C–b)
time to autoimmunity
Nivolumab
P=0.5342 (matched)
P=0.0094 (unmatched)
autoimmune–free %
90 days
Squamous num. at risk
matched
target therapy 75.0 27.0 9.3 6.8 5.0 1.5
Nivolumab 75 24 7 4 1 NA
unmatched
target therapy 358 123 52 21 14 7
Nivolumab 226 55 15 6 1 NA
Months after therapy initiation

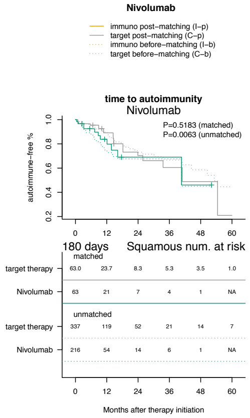
Nivolumab
immuno post–matching (I–p)
target post–matching (C–p)
immuno before–matching (I–b)
target before–matching (C–b)
time to autoimmunity
Nivolumab
P=0.5183 (matched)
P=0.0063 (unmatched)
autoimmune–free %
180 days
Squamous num. at risk
matched
target therapy 63.0 23.7 8.3 5.3 3.5 1.0
Nivolumab 63 21 7 4 1 NA
unmatched
target therapy 337 119 52 21 14 7
Nivolumab 216 54 14 6 1 NA
Months after therapy initiation

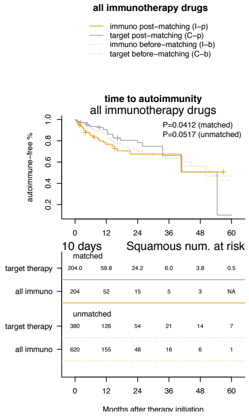
all immunotherapy drugs
immuno post–matching (I–p)
target post–matching (C–p)
immuno before–matching (I–b)
target before–matching (C–b)
time to autoimmunity
all immunotherapy drugs
P=0.0412 (matched)
P=0.0517 (unmatched)
autoimmune–free %
10 days
Squamous num. at risk
matched
target therapy 204.0 59.8 24.2 6.0 3.8 0.5
all immuno 204 52 15 5 3 NA
unmatched
target therapy 380 126 54 21 14 7
all immuno 620 155 48 16 6 1
Months after therapy initiation

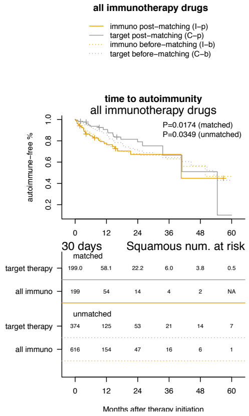
all immunotherapy drugs
immuno post–matching (I–p)
target post–matching (C–p)
immuno before–matching (I–b)
target before–matching (C–b)
time to autoimmunity
all immunotherapy drugs
P=0.0174 (matched)
P=0.0349 (unmatched)
autoimmune–free %
30 days
Squamous num. at risk
matched
target therapy 199.0 58.1 22.2 6.0 3.8 0.5
all immuno 199 54 14 4 2 NA
unmatched
target therapy 374 125 53 21 14 7
all immuno 616 154 47 16 6 1
Months after therapy initiation

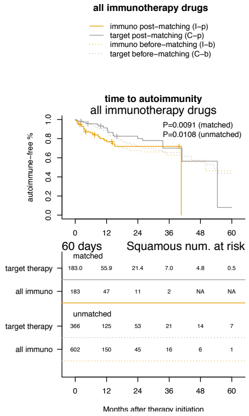
all immunotherapy drugs
immuno post–matching (I–p)
target post–matching (C–p)
immuno before–matching (I–b)
target before–matching (C–b)
time to autoimmunity
all immunotherapy drugs
P=0.0091 (matched)
P=0.0108 (unmatched)
autoimmune–free %
60 days
Squamous num. at risk
matched
target therapy 183.0 55.9 21.4 7.0 4.8 0.5
all immuno 183 47 11 2 NA NA
unmatched
target therapy 366 125 53 21 14 7
all immuno 602 150 45 16 6 1
Months after therapy initiation

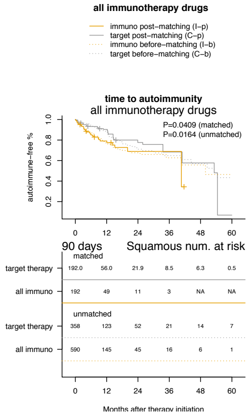
all immunotherapy drugs
immuno post–matching (I–p)
target post–matching (C–p)
immuno before–matching (I–b)
target before–matching (C–b)
time to autoimmunity
all immunotherapy drugs
P=0.0409 (matched)
P=0.0164 (unmatched)
autoimmune–free %
90 days
Squamous num. at risk
matched
target therapy 192.0 56.0 21.9 8.5 6.3 0.5
all immuno 192 49 11 3 NA NA
unmatched
target therapy 358 123 52 21 14 7
all immuno 590 145 45 16 6 1
Months after therapy initiation

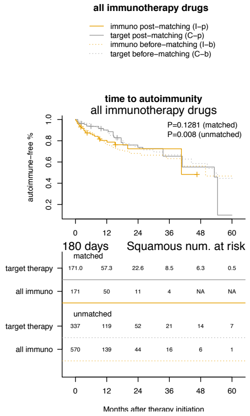
all immunotherapy drugs
immuno post–matching (I–p)
target post–matching (C–p)
immuno before–matching (I–b)
target before–matching (C–b)
time to autoimmunity
all immunotherapy drugs
P=0.1281 (matched)
P=0.008 (unmatched)
autoimmune–free %
180 days
Squamous num. at risk
matched
target therapy 171.0 57.3 22.6 8.5 6.3 0.5
all immuno 171 50 11 4 NA NA
unmatched
target therapy 337 119 52 21 14 7
all immuno 570 139 44 16 6 1
Months after therapy initiation

Additional Time-Frame Requirement

Most patients in the treatment group started their first therapy only after 2015. However, the control group patients spread out quite evenly throughout the entire study period. To address the potential impact of comparing the treatment group after 2015 with the control group in 2008-2019, we conducted additional analyses restricting both the control and the treatment group to those who started treatment after 2015.

Figure S8 presents the results in detail. We observe similar estimates as in the main text but with reduced statistical power due to the exclusion of pre-2015 patients.

Figure S8. Imposing an additional time-frame requirement that the patients must have chemotherapy or immunotherapy initiation dates after 2015.

Chemotherapy

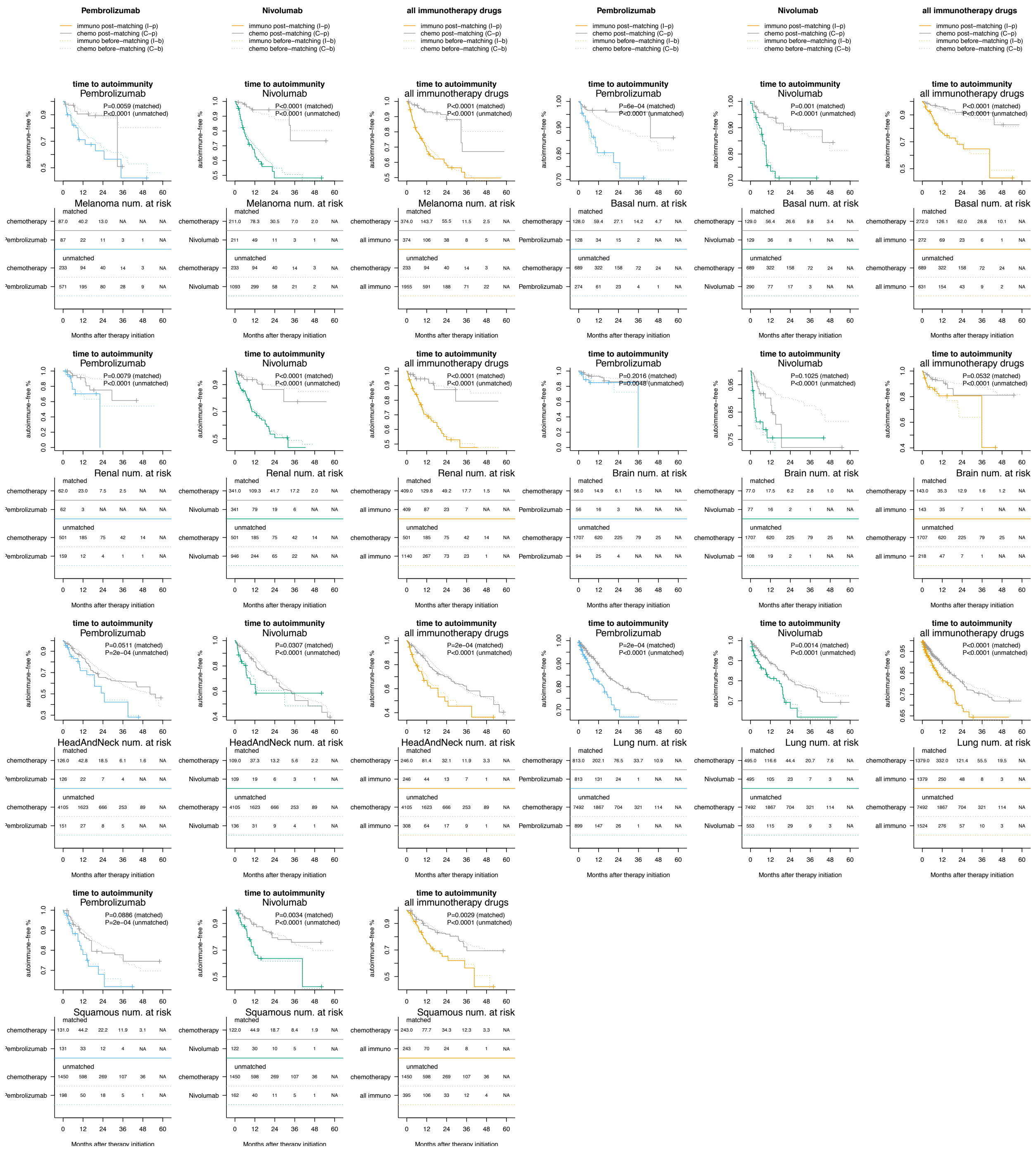

Targeted therapy

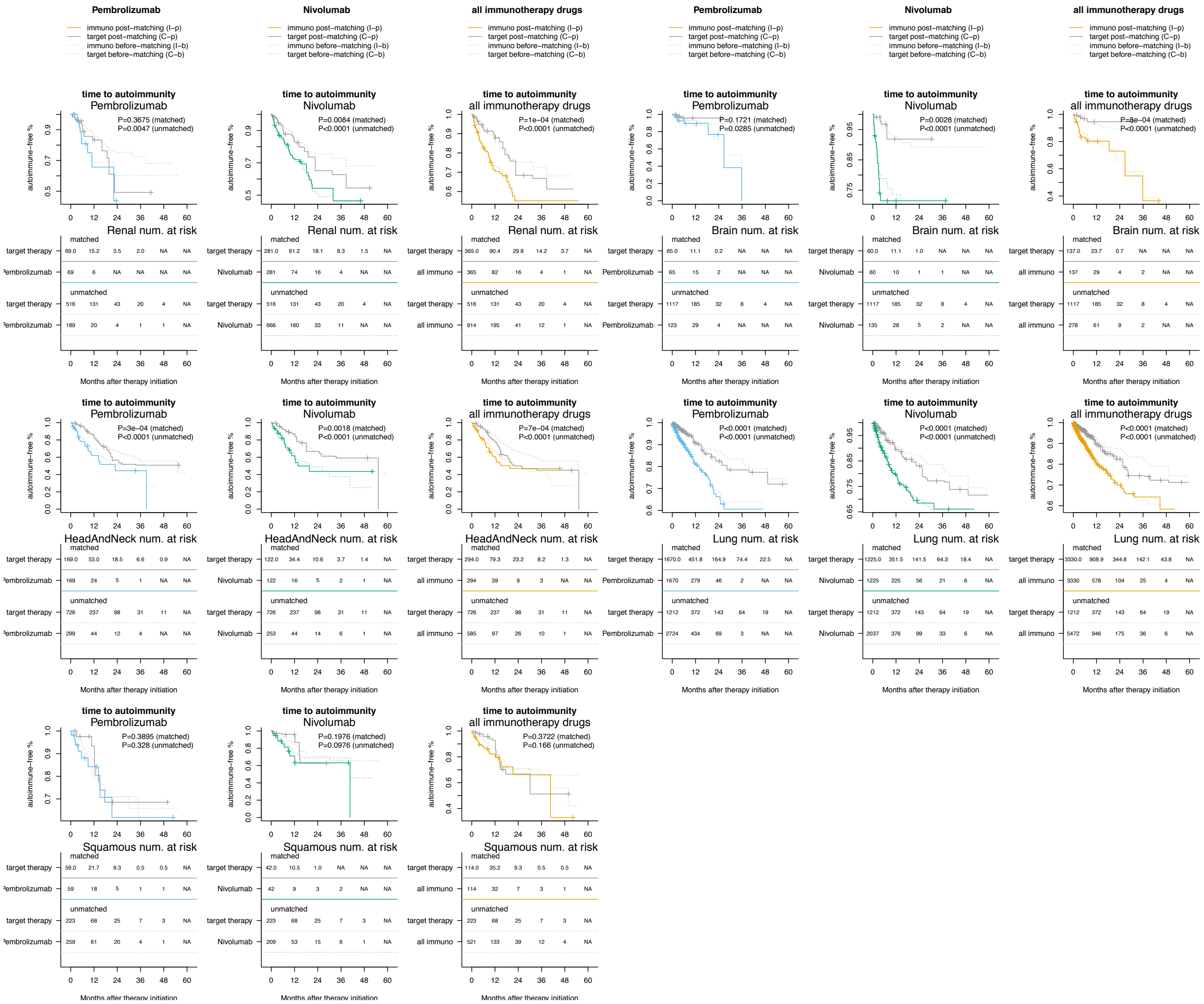

Excluding patients from the immunotherapy group if they have received chemotherapy or targeted therapy before immunotherapy

In our main results, the treatment initiation date of immunotherapy patients is defined to be the date of the first immunotherapy administration, even in the case where the immunotherapy is used after chemotherapy as second-line therapy. One might argue if the previous chemotherapy (or targeted therapy) would affect the hazard ratio after immunotherapy. In this section, we address this issue by excluding patients from the immunotherapy group if they have received chemotherapy or targeted therapy before immunotherapy.

Figure S9 shows a consistent risk difference between the treatment group and the control group when these additional exclusion criteria are applied. Due to the smaller sample size, the statistical power is weaker in this analysis.

Figure S9. Sensitivity studies on the impact of excluding patients from the immunotherapy group if they have received chemotherapy or targeted therapy before immunotherapy.

Chemotherapy

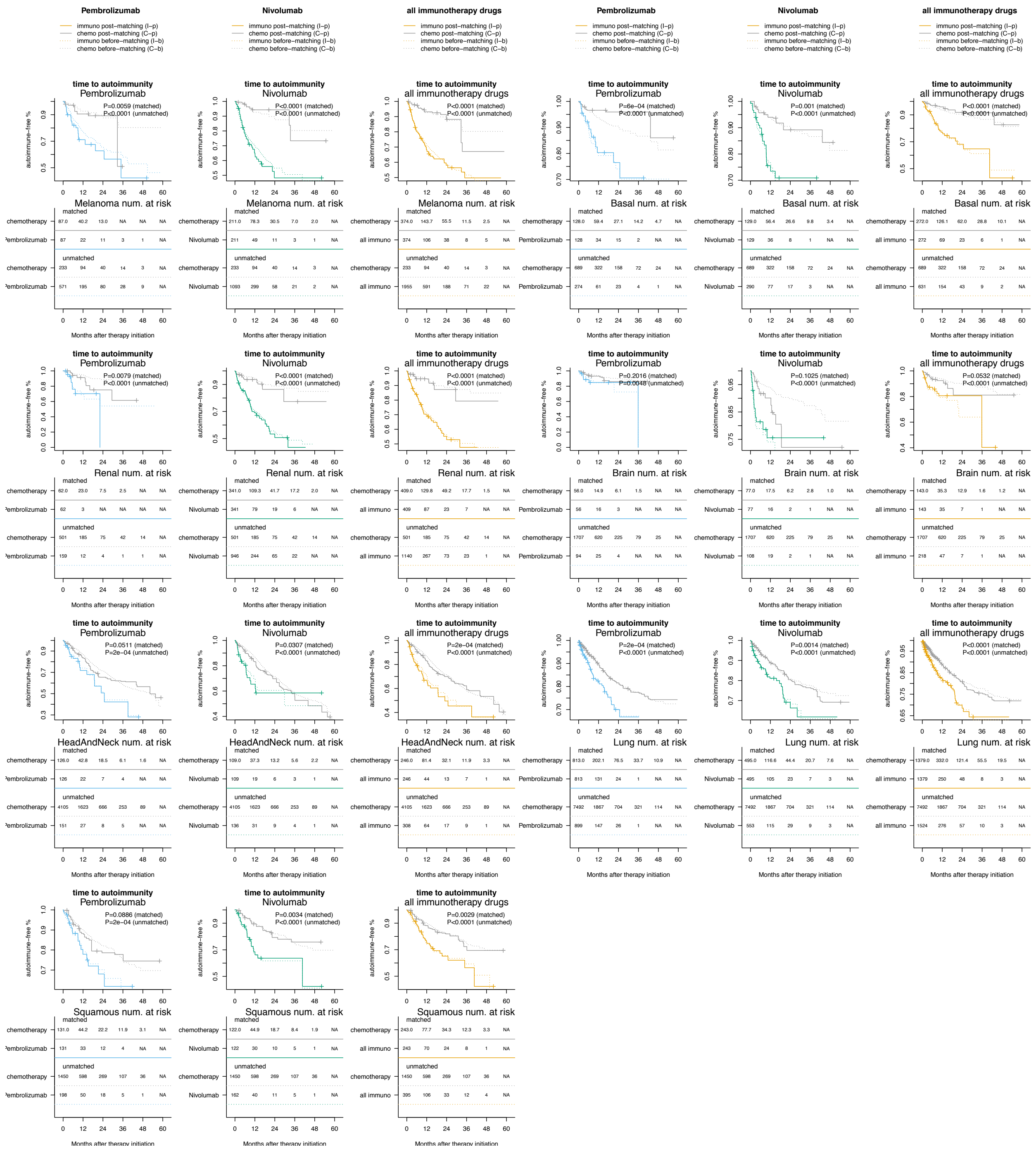

Targeted therapy

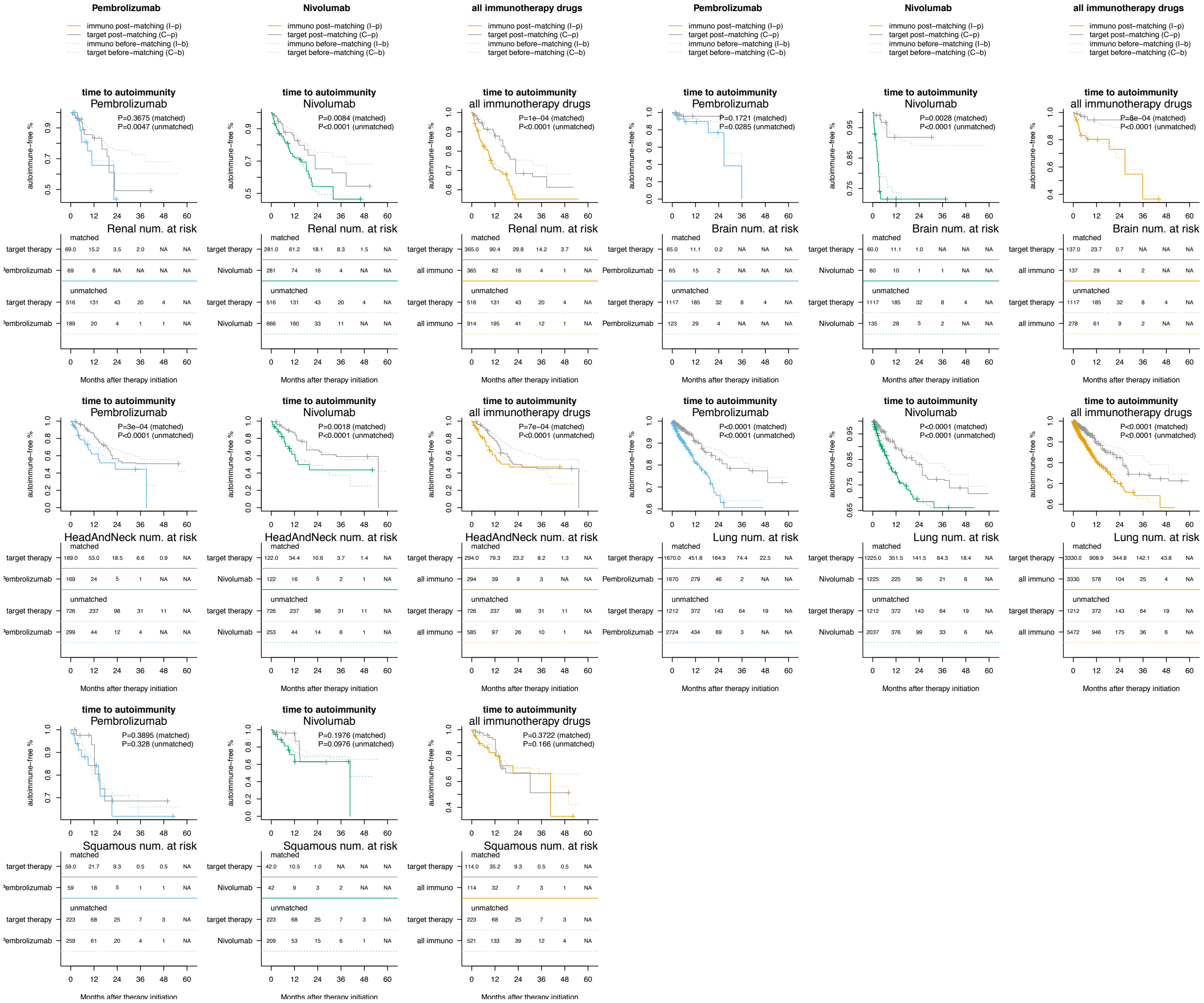

Subpopulation Analyses

Lines of Immunotherapy

We conducted stratified analyses by separating the patients into two sub-groups based on the lines of immunotherapy. Those receiving immunotherapy without prior chemotherapy are classified as the first-line immunotherapy group, and those receiving immunotherapy after chemotherapy administration are classified as the second-line immunotherapy group. Figure S10 presents the results in detail. Nivolumab is more frequently used as a second-line therapy, while Pembrolizumab is more often used as first-line therapy. The difference might be due to the recency of Pembrolizumab approval and that the drug is approved as a first-line treatment for metastatic non-small cell lung cancer. The risk of autoimmune disease is similar regardless of the lines of immunotherapy.

Figure S10. Results stratified by the lines of immunotherapy. The top panel compares the first-line immunotherapy group with the chemotherapy group, and the bottom panel compares the second-line immunotherapy group with the chemotherapy group. It turns out that we have most patients from the first-line immunotherapy group, and in that case, we don't need to worry about the first or second-line because the proportion of people in the second-line is negligible compared to the whole immunotherapy group. For lung cancer and renal cancer, the population is quite evenly distributed between two categories, and the risk of autoimmune disease is similar regardless of the lines of immunotherapy.

Chemotherapy

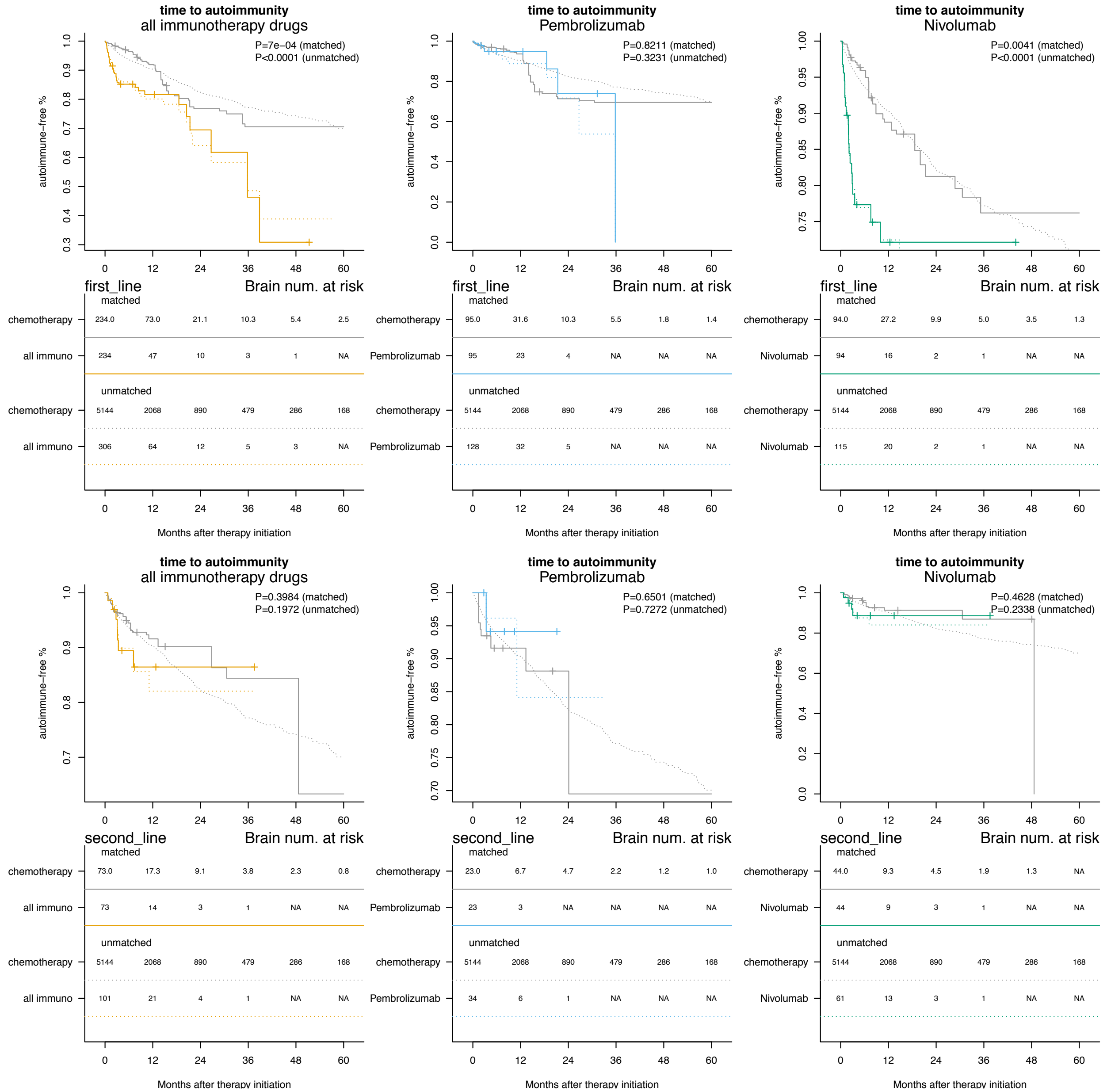

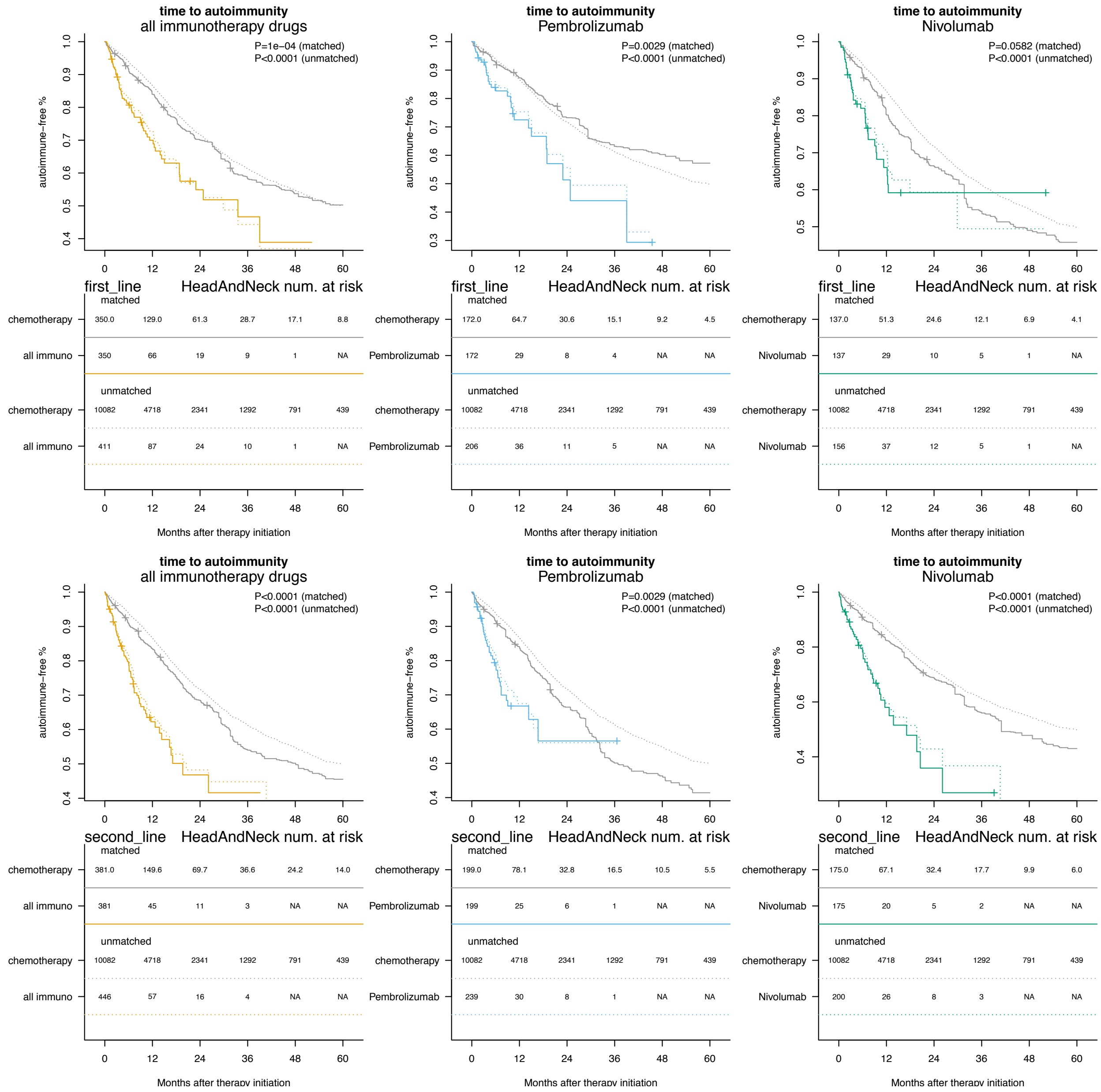
time to autoimmunity
all immunotherapy drugs
P=1e−04 (matched)
P<0.0001 (unmatched)
autoimmune−free %
first_line
HeadAndNeck num. at risk
matched
chemotherapy 350.0 129.0 61.3 28.7 17.1 8.8
all immuno 350 66 19 9 1 NA
unmatched
chemotherapy 10082 4718 2341 1292 791 439
all immuno 411 87 24 10 1 NA
0 12 24 36 48 60
Months after therapy initiation
time to autoimmunity
Pembrolizumab
P=0.0029 (matched)
P<0.0001 (unmatched)
autoimmune−free %
first_line
HeadAndNeck num. at risk
matched
chemotherapy 172.0 64.7 30.6 15.1 9.2 4.5
Pembrolizumab 172 29 8 4 NA NA
unmatched
chemotherapy 10082 4718 2341 1292 791 439
Pembrolizumab 206 36 11 5 NA NA
Months after therapy initiation
time to autoimmunity
Nivolumab
P=0.0582 (matched)
P<0.0001 (unmatched)
autoimmune−free %
first_line
HeadAndNeck num. at risk
matched
chemotherapy 137.0 51.3 24.6 12.1 6.9 4.1
Nivolumab 137 29 10 5 1 NA
unmatched
chemotherapy 10082 4718 2341 1292 791 439
Nivolumab 156 37 12 5 1 NA
Months after therapy initiation
time to autoimmunity
all immunotherapy drugs
P<0.0001 (matched)
P<0.0001 (unmatched)
autoimmune−free %
second_line
HeadAndNeck num. at risk
matched
chemotherapy 381.0 149.6 69.7 36.6 24.2 14.0
all immuno 381 45 11 3 NA NA
unmatched
chemotherapy 10082 4718 2341 1292 791 439
all immuno 446 57 16 4 NA NA
Months after therapy initiation
time to autoimmunity
Pembrolizumab
P=0.0029 (matched)
P<0.0001 (unmatched)
autoimmune−free %
second_line
HeadAndNeck num. at risk
matched
chemotherapy 199.0 78.1 32.8 16.5 10.5 5.5
Pembrolizumab 199 25 6 1 NA NA
unmatched
chemotherapy 10082 4718 2341 1292 791 439
Pembrolizumab 239 30 8 1 NA NA
Months after therapy initiation
time to autoimmunity
Nivolumab
P<0.0001 (matched)
P<0.0001 (unmatched)
autoimmune−free %
second_line
HeadAndNeck num. at risk
matched
chemotherapy 175.0 67.1 32.4 17.7 9.9 6.0
Nivolumab 175 20 5 2 NA NA
unmatched
chemotherapy 10082 4718 2341 1292 791 439
Nivolumab 200 26 8 3 NA NA
Months after therapy initiation

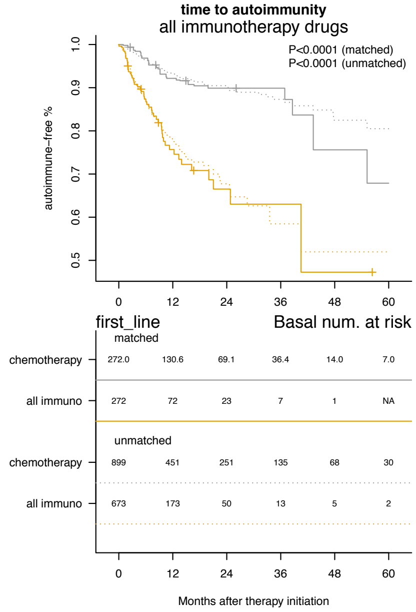

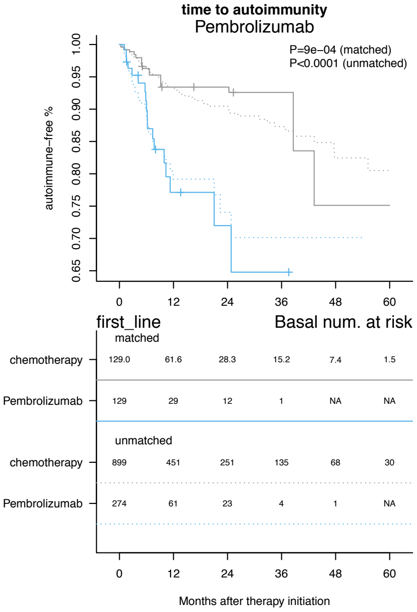

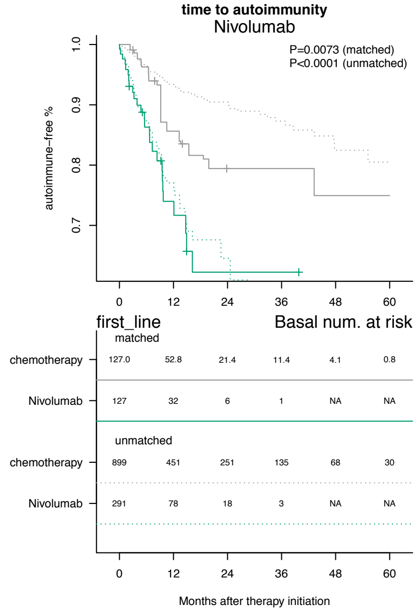

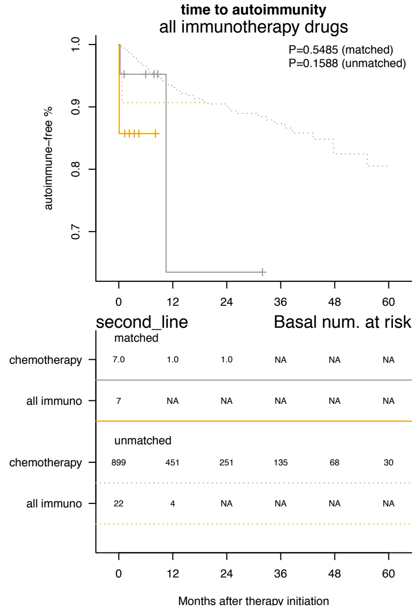

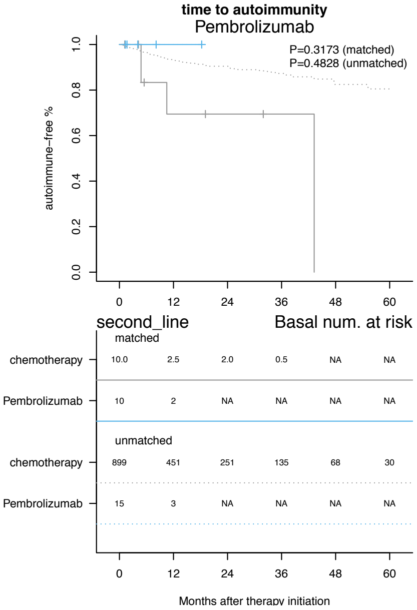

no matched member

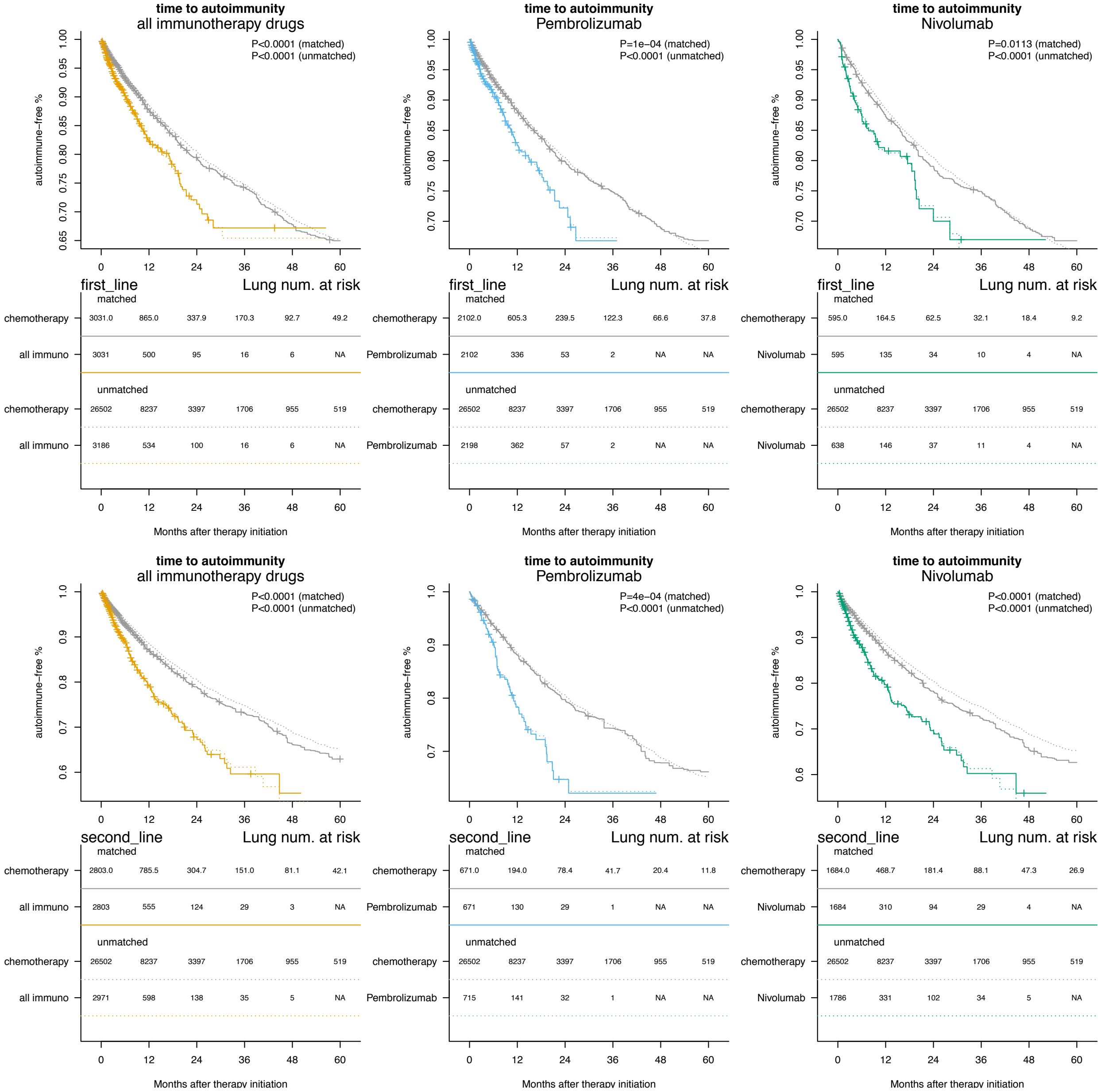
time to autoimmunity
all immunotherapy drugs
P<0.0001 (matched)
P<0.0001 (unmatched)
autoimmune−free %
first_line
Lung num. at risk
matched
chemotherapy
3031.0 865.0 337.9 170.3 92.7 49.2
all immuno
3031 500 95 16 6 NA
unmatched
chemotherapy
26502 8237 3397 1706 955 519
all immuno
3186 534 100 16 6 NA
Months after therapy initiation
time to autoimmunity
Pembrolizumab
P=1e−04 (matched)
P<0.0001 (unmatched)
autoimmune−free %
first_line
Lung num. at risk
matched
chemotherapy
2102.0 605.3 239.5 122.3 66.6 37.8
Pembrolizumab
2102 336 53 2 NA NA
unmatched
chemotherapy
26502 8237 3397 1706 955 519
Pembrolizumab
2198 362 57 2 NA NA
Months after therapy initiation
time to autoimmunity
Nivolumab
P=0.0113 (matched)
P<0.0001 (unmatched)
autoimmune−free %
first_line
Lung num. at risk
matched
chemotherapy
595.0 164.5 62.5 32.1 18.4 9.2
Nivolumab
595 135 34 10 4 NA
unmatched
chemotherapy
26502 8237 3397 1706 955 519
Nivolumab
638 146 37 11 4 NA
Months after therapy initiation
time to autoimmunity
all immunotherapy drugs
P<0.0001 (matched)
P<0.0001 (unmatched)
autoimmune−free %
second_line
Lung num. at risk
matched
chemotherapy
2803.0 785.5 304.7 151.0 81.1 42.1
all immuno
2803 555 124 29 3 NA
unmatched
chemotherapy
26502 8237 3397 1706 955 519
all immuno
2971 598 138 35 5 NA
Months after therapy initiation
time to autoimmunity
Pembrolizumab
P=4e−04 (matched)
P<0.0001 (unmatched)
autoimmune−free %
second_line
Lung num. at risk
matched
chemotherapy
671.0 194.0 78.4 41.7 20.4 11.8
Pembrolizumab
671 130 29 1 NA NA
unmatched
chemotherapy
26502 8237 3397 1706 955 519
Pembrolizumab
715 141 32 1 NA NA
Months after therapy initiation
time to autoimmunity
Nivolumab
P<0.0001 (matched)
P<0.0001 (unmatched)
autoimmune−free %
second_line
Lung num. at risk
matched
chemotherapy
1684.0 468.7 181.4 88.1 47.3 26.9
Nivolumab
1684 310 94 29 4 NA
unmatched
chemotherapy
26502 8237 3397 1706 955 519
Nivolumab
1786 331 102 34 5 NA
Months after therapy initiation

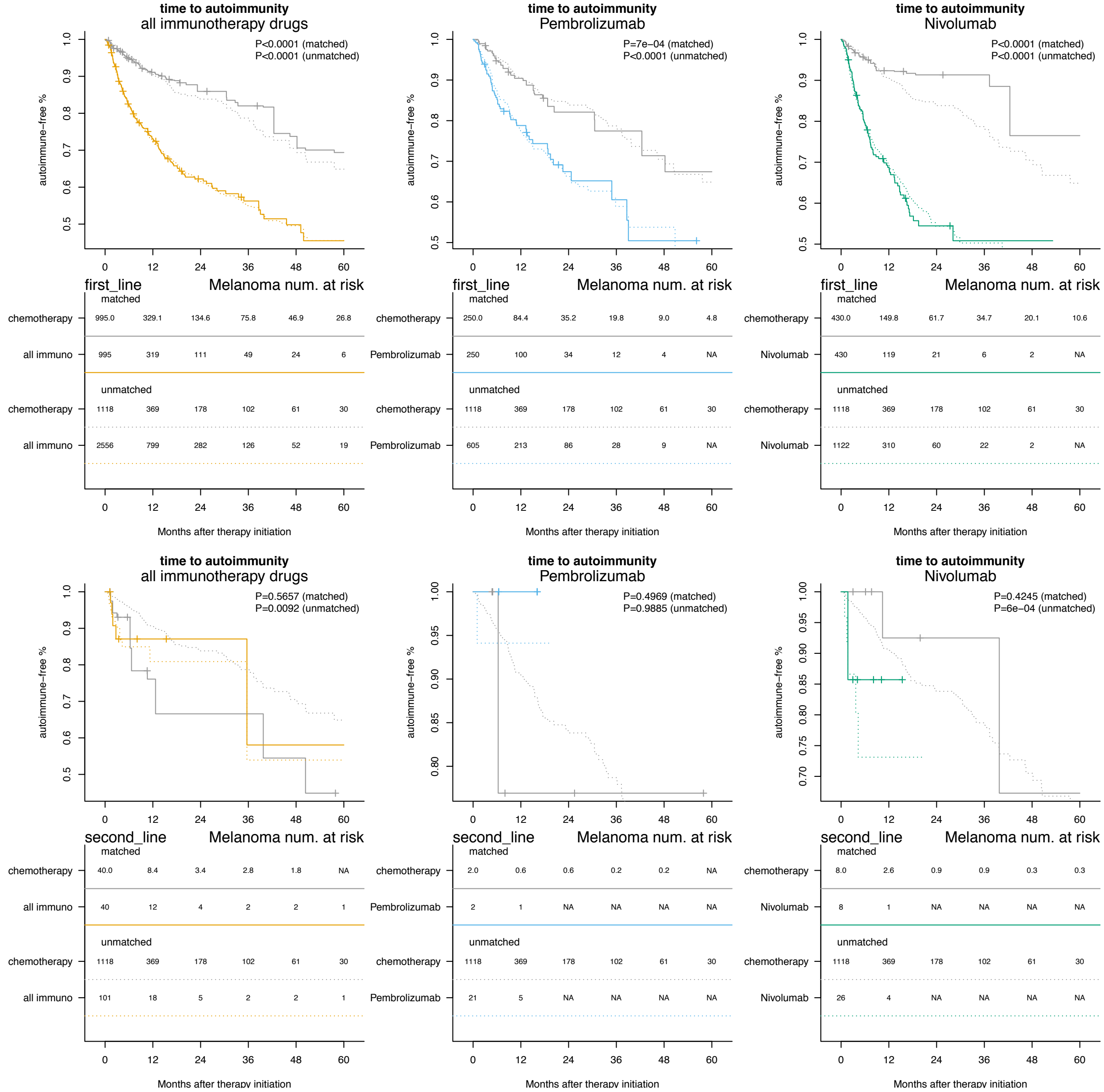

time to autoimmunity
all immunotherapy drugs
P<0.0001 (matched)
P<0.0001 (unmatched)
autoimmune−free %
first_line Melanoma num. at risk
matched
chemotherapy 995.0 329.1 134.6 75.8 46.9 26.8
all immuno 995 319 111 49 24 6
unmatched
chemotherapy 1118 369 178 102 61 30
all immuno 2556 799 282 126 52 19
Months after therapy initiation
time to autoimmunity
Pembrolizumab
P=7e−04 (matched)
P<0.0001 (unmatched)
autoimmune−free %
first_line Melanoma num. at risk
matched
chemotherapy 250.0 84.4 35.2 19.8 9.0 4.8
Pembrolizumab 250 100 34 12 4 NA
unmatched
chemotherapy 1118 369 178 102 61 30
Pembrolizumab 605 213 86 28 9 NA
Months after therapy initiation
time to autoimmunity
Nivolumab
P<0.0001 (matched)
P<0.0001 (unmatched)
autoimmune−free %
first_line Melanoma num. at risk
matched
chemotherapy 430.0 149.8 61.7 34.7 20.1 10.6
Nivolumab 430 119 21 6 2 NA
unmatched
chemotherapy 1118 369 178 102 61 30
Nivolumab 1122 310 60 22 2 NA
Months after therapy initiation
time to autoimmunity
all immunotherapy drugs
P=0.5657 (matched)
P=0.0092 (unmatched)
autoimmune−free %
second_line Melanoma num. at risk
matched
chemotherapy 40.0 8.4 3.4 2.8 1.8 NA
all immuno 40 12 4 2 2 1
unmatched
chemotherapy 1118 369 178 102 61 30
all immuno 101 18 5 2 2 1
Months after therapy initiation
time to autoimmunity
Pembrolizumab
P=0.4969 (matched)
P=0.9885 (unmatched)
autoimmune−free %
second_line Melanoma num. at risk
matched
chemotherapy 2.0 0.6 0.6 0.2 0.2 NA
Pembrolizumab 2 1 NA NA NA NA
unmatched
chemotherapy 1118 369 178 102 61 30
Pembrolizumab 21 5 NA NA NA NA
Months after therapy initiation
time to autoimmunity
Nivolumab
P=0.4245 (matched)
P=6e−04 (unmatched)
autoimmune−free %
second_line Melanoma num. at risk
matched
chemotherapy 8.0 2.6 0.9 0.9 0.3 0.3
Nivolumab 8 1 NA NA NA NA
unmatched
chemotherapy 1118 369 178 102 61 30
Nivolumab 26 4 NA NA NA NA
Months after therapy initiation

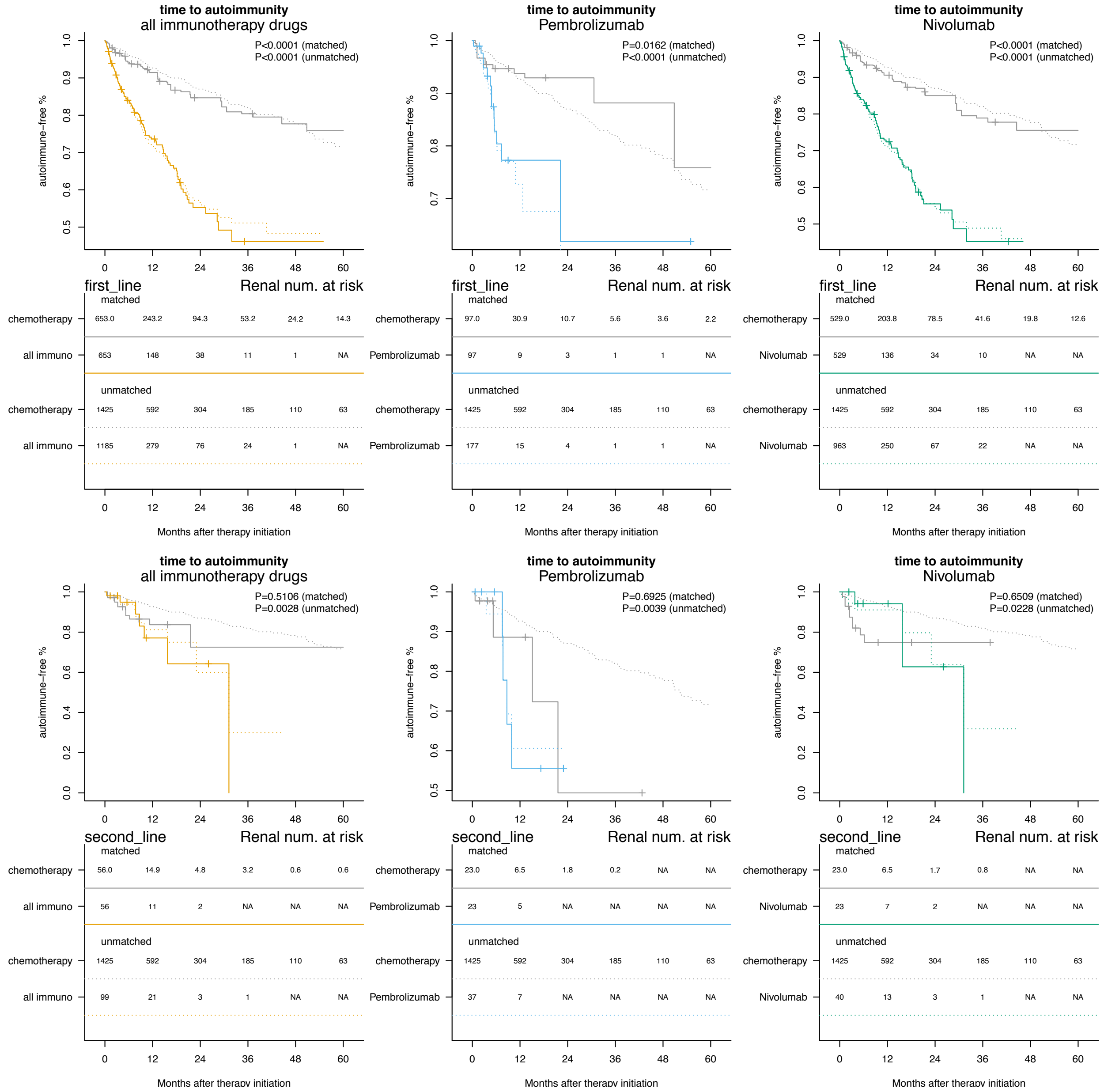

time to autoimmunity
all immunotherapy drugs
P<0.0001 (matched)
P<0.0001 (unmatched)
autoimmune−free %
first_line
Renal num. at risk
matched
chemotherapy 653.0 243.2 94.3 53.2 24.2 14.3
all immuno 653 148 38 11 1 NA
unmatched
chemotherapy 1425 592 304 185 110 63
all immuno 1185 279 76 24 1 NA
Months after therapy initiation
time to autoimmunity
Pembrolizumab
P=0.0162 (matched)
P<0.0001 (unmatched)
autoimmune−free %
first_line
Renal num. at risk
matched
chemotherapy 97.0 30.9 10.7 5.6 3.6 2.2
Pembrolizumab 97 9 3 1 1 NA
unmatched
chemotherapy 1425 592 304 185 110 63
Pembrolizumab 177 15 4 1 1 NA
Months after therapy initiation
time to autoimmunity
Nivolumab
P<0.0001 (matched)
P<0.0001 (unmatched)
autoimmune−free %
first_line
Renal num. at risk
matched
chemotherapy 529.0 203.8 78.5 41.6 19.8 12.6
Nivolumab 529 136 34 10 NA NA
unmatched
chemotherapy 1425 592 304 185 110 63
Nivolumab 963 250 67 22 NA NA
Months after therapy initiation
time to autoimmunity
all immunotherapy drugs
P=0.5106 (matched)
P=0.0028 (unmatched)
autoimmune−free %
second_line
Renal num. at risk
matched
chemotherapy 56.0 14.9 4.8 3.2 0.6 0.6
all immuno 56 11 2 NA NA NA
unmatched
chemotherapy 1425 592 304 185 110 63
all immuno 99 21 3 1 NA NA
Months after therapy initiation
time to autoimmunity
Pembrolizumab
P=0.6925 (matched)
P=0.0039 (unmatched)
autoimmune−free %
second_line
Renal num. at risk
matched
chemotherapy 23.0 6.5 1.8 0.2 NA NA
Pembrolizumab 23 5 NA NA NA NA
unmatched
chemotherapy 1425 592 304 185 110 63
Pembrolizumab 37 7 NA NA NA NA
Months after therapy initiation
time to autoimmunity
Nivolumab
P=0.6509 (matched)
P=0.0228 (unmatched)
autoimmune−free %
second_line
Renal num. at risk
matched
chemotherapy 23.0 6.5 1.7 0.8 NA NA
Nivolumab 23 7 2 NA NA NA
unmatched
chemotherapy 1425 592 304 185 110 63
Nivolumab 40 13 3 1 NA NA
Months after therapy initiation

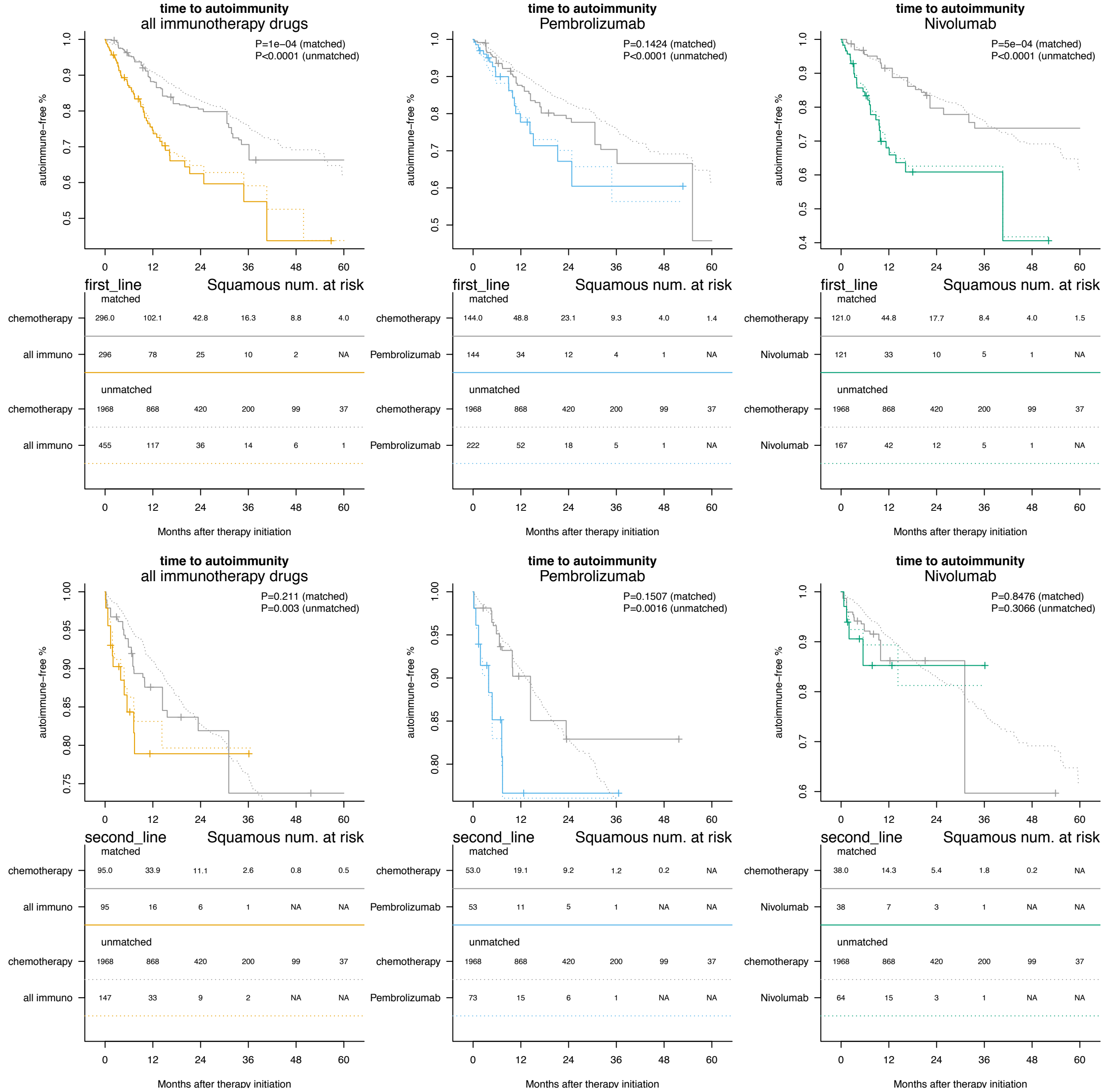
time to autoimmunity
all immunotherapy drugs
P=1e−04 (matched)
P<0.0001 (unmatched)
autoimmune−free %
first_line
Squamous num. at risk
matched
chemotherapy 296.0 102.1 42.8 16.3 8.8 4.0
all immuno 296 78 25 10 2 NA
unmatched
chemotherapy 1968 868 420 200 99 37
all immuno 455 117 36 14 6 1
Months after therapy initiation
time to autoimmunity
Pembrolizumab
P=0.1424 (matched)
P<0.0001 (unmatched)
autoimmune−free %
first_line
Squamous num. at risk
matched
chemotherapy 144.0 48.8 23.1 9.3 4.0 1.4
Pembrolizumab 144 34 12 4 1 NA
unmatched
chemotherapy 1968 868 420 200 99 37
Pembrolizumab 222 52 18 5 1 NA
Months after therapy initiation
time to autoimmunity
Nivolumab
P=5e−04 (matched)
P<0.0001 (unmatched)
autoimmune−free %
first_line
Squamous num. at risk
matched
chemotherapy 121.0 44.8 17.7 8.4 4.0 1.5
Nivolumab 121 33 10 5 1 NA
unmatched
chemotherapy 1968 868 420 200 99 37
Nivolumab 167 42 12 5 1 NA
Months after therapy initiation
time to autoimmunity
all immunotherapy drugs
P=0.211 (matched)
P=0.003 (unmatched)
autoimmune−free %
second_line
Squamous num. at risk
matched
chemotherapy 95.0 33.9 11.1 2.6 0.8 0.5
all immuno 95 16 6 1 NA NA
unmatched
chemotherapy 1968 868 420 200 99 37
all immuno 147 33 9 2 NA NA
Months after therapy initiation
time to autoimmunity
Pembrolizumab
P=0.1507 (matched)
P=0.0016 (unmatched)
autoimmune−free %
second_line
Squamous num. at risk
matched
chemotherapy 53.0 19.1 9.2 1.2 0.2 NA
Pembrolizumab 53 11 5 1 NA NA
unmatched
chemotherapy 1968 868 420 200 99 37
Pembrolizumab 73 15 6 1 NA NA
Months after therapy initiation
time to autoimmunity
Nivolumab
P=0.8476 (matched)
P=0.3066 (unmatched)
autoimmune−free %
second_line
Squamous num. at risk
matched
chemotherapy 38.0 14.3 5.4 1.8 0.2 NA
Nivolumab 38 7 3 1 NA NA
unmatched
chemotherapy 1968 868 420 200 99 37
Nivolumab 64 15 3 1 NA NA
Months after therapy initiation

Targeted therapy

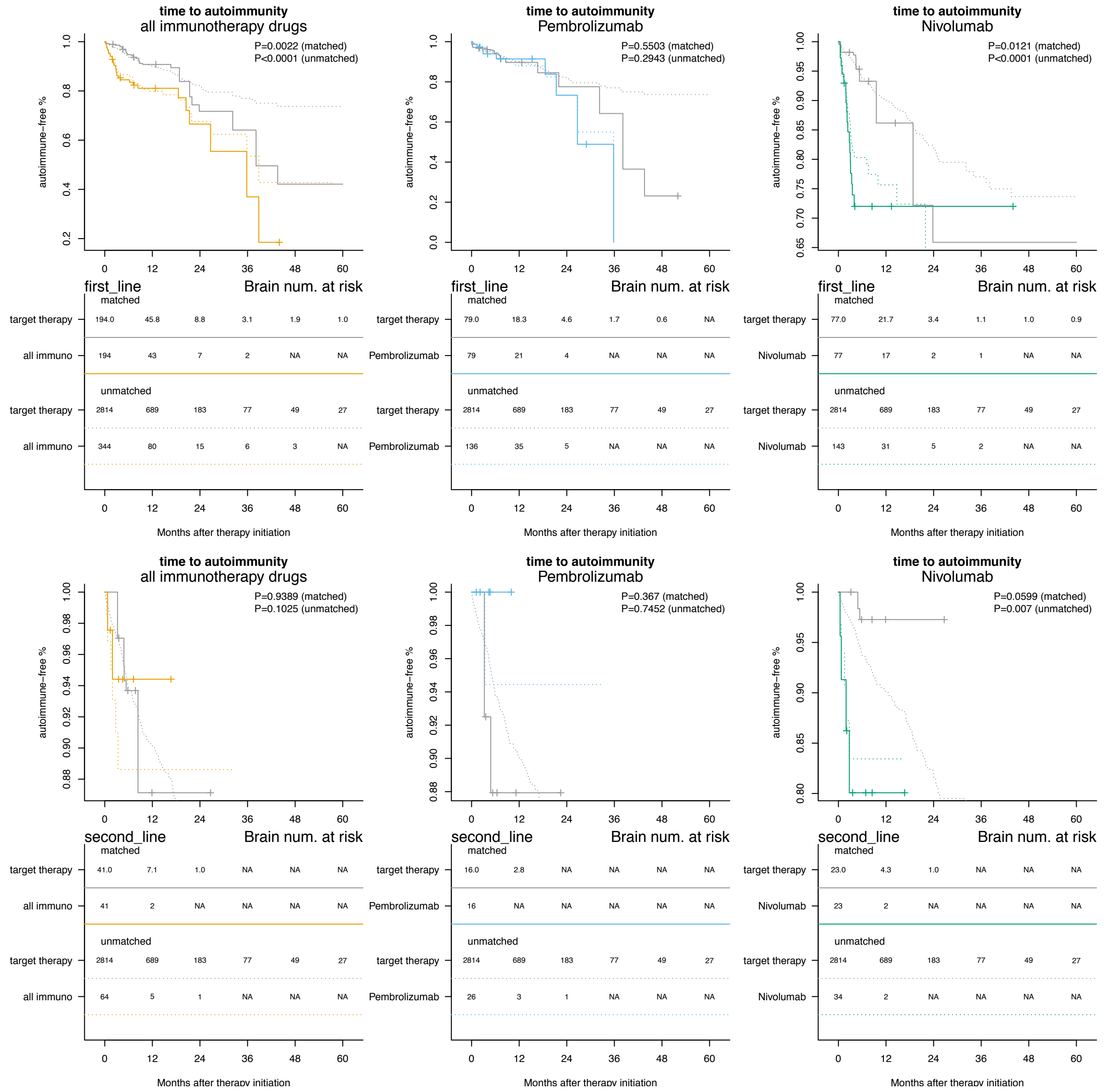

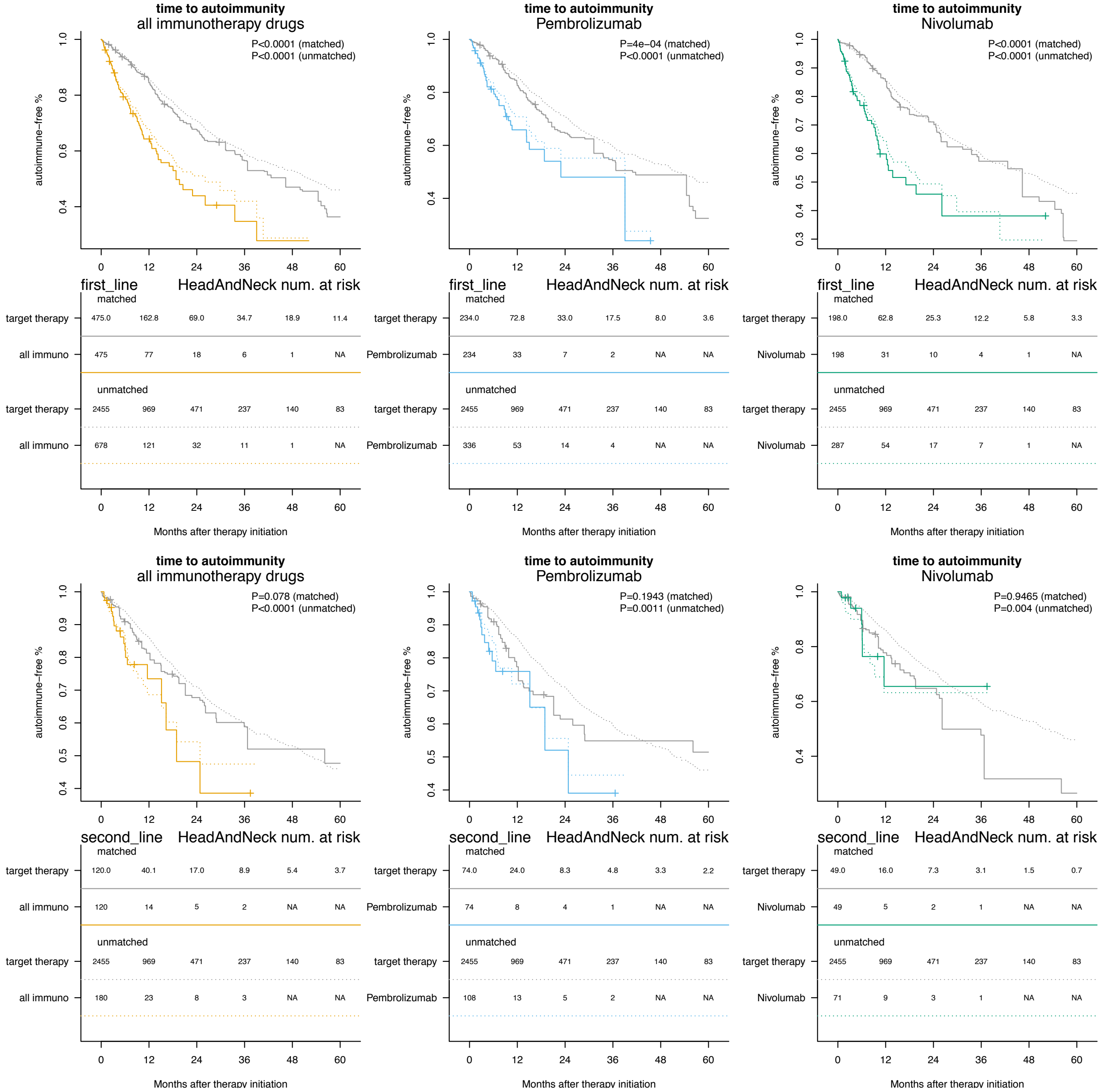
time to autoimmunity
all immunotherapy drugs
P<0.0001 (matched)
P<0.0001 (unmatched)
autoimmune−free %
first_line HeadAndNeck num. at risk
matched
target therapy 475.0 162.8 69.0 34.7 18.9 11.4
all immuno 475 77 18 6 1 NA
unmatched
target therapy 2455 969 471 237 140 83
all immuno 678 121 32 11 1 NA
Months after therapy initiation
time to autoimmunity
Pembrolizumab
P=4e−04 (matched)
P<0.0001 (unmatched)
first_line HeadAndNeck num. at risk
matched
target therapy 234.0 72.8 33.0 17.5 8.0 3.6
Pembrolizumab 234 33 7 2 NA NA
unmatched
target therapy 2455 969 471 237 140 83
Pembrolizumab 336 53 14 4 NA NA
Months after therapy initiation
time to autoimmunity
Nivolumab
P<0.0001 (matched)
P<0.0001 (unmatched)
first_line HeadAndNeck num. at risk
matched
target therapy 198.0 62.8 25.3 12.2 5.8 3.3
Nivolumab 198 31 10 4 1 NA
unmatched
target therapy 2455 969 471 237 140 83
Nivolumab 287 54 17 7 1 NA
Months after therapy initiation
time to autoimmunity
all immunotherapy drugs
P=0.078 (matched)
P<0.0001 (unmatched)
second_line HeadAndNeck num. at risk
matched
target therapy 120.0 40.1 17.0 8.9 5.4 3.7
all immuno 120 14 5 2 NA NA
unmatched
target therapy 2455 969 471 237 140 83
all immuno 180 23 8 3 NA NA
Months after therapy initiation
time to autoimmunity
Pembrolizumab
P=0.1943 (matched)
P=0.0011 (unmatched)
second_line HeadAndNeck num. at risk
matched
target therapy 74.0 24.0 8.3 4.8 3.3 2.2
Pembrolizumab 74 8 4 1 NA NA
unmatched
target therapy 2455 969 471 237 140 83
Pembrolizumab 108 13 5 2 NA NA
Months after therapy initiation
time to autoimmunity
Nivolumab
P=0.9465 (matched)
P=0.004 (unmatched)
second_line HeadAndNeck num. at risk
matched
target therapy 49.0 16.0 7.3 3.1 1.5 0.7
Nivolumab 49 5 2 1 NA NA
unmatched
target therapy 2455 969 471 237 140 83
Nivolumab 71 9 3 1 NA NA
Months after therapy initiation

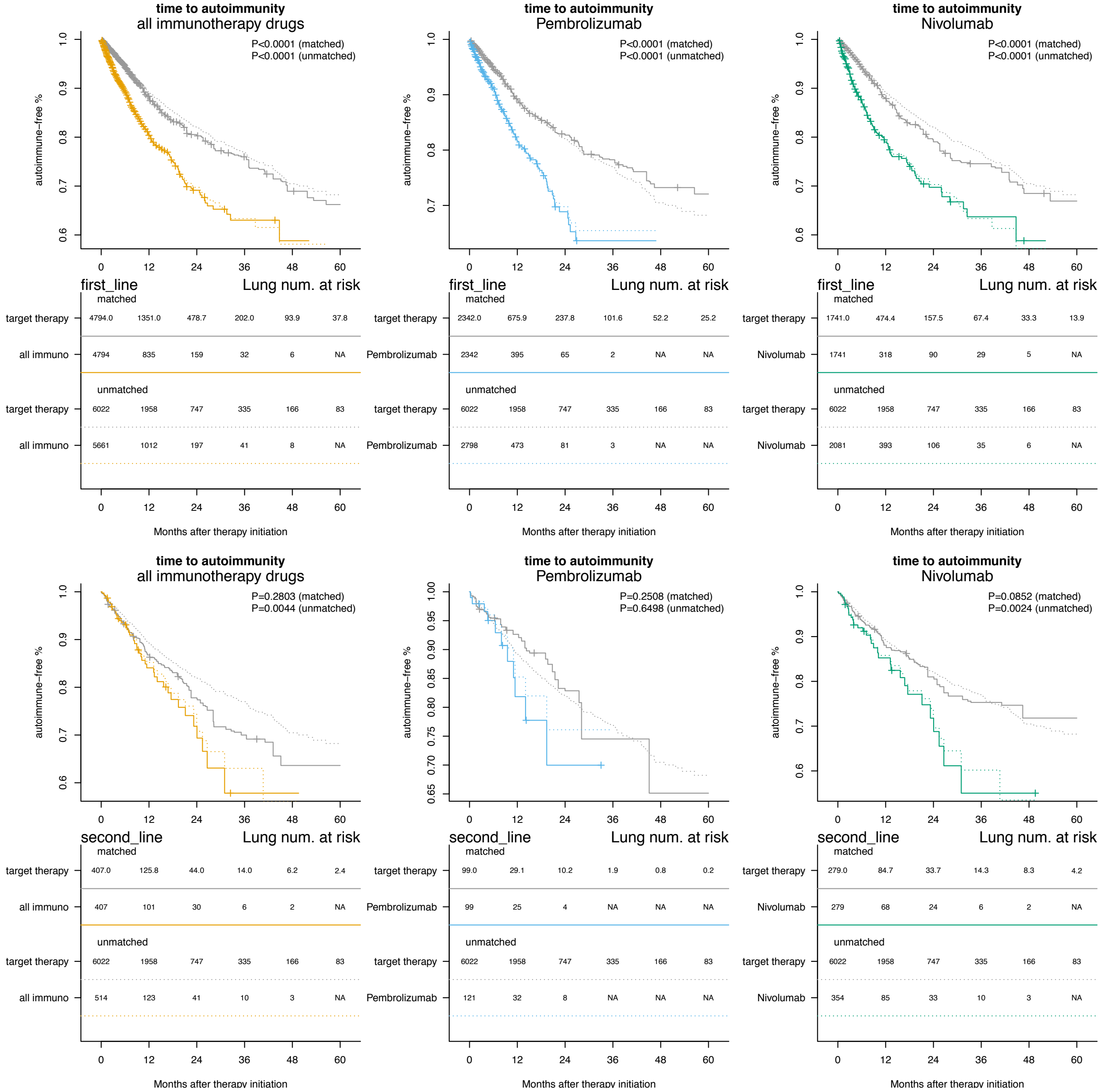
time to autoimmunity
all immunotherapy drugs
P<0.0001 (matched)
P<0.0001 (unmatched)
autoimmune−free %
first_line
Lung num. at risk
matched
target therapy 4794.0 1351.0 478.7 202.0 93.9 37.8
all immuno 4794 835 159 32 6 NA
unmatched
target therapy 6022 1958 747 335 166 83
all immuno 5661 1012 197 41 8 NA
Months after therapy initiation
time to autoimmunity
Pembrolizumab
P<0.0001 (matched)
P<0.0001 (unmatched)
autoimmune−free %
first_line
Lung num. at risk
matched
target therapy 2342.0 675.9 237.8 101.6 52.2 25.2
Pembrolizumab 2342 395 65 2 NA NA
unmatched
target therapy 6022 1958 747 335 166 83
Pembrolizumab 2798 473 81 3 NA NA
Months after therapy initiation
time to autoimmunity
Nivolumab
P<0.0001 (matched)
P<0.0001 (unmatched)
autoimmune−free %
first_line
Lung num. at risk
matched
target therapy 1741.0 474.4 157.5 67.4 33.3 13.9
Nivolumab 1741 318 90 29 5 NA
unmatched
target therapy 6022 1958 747 335 166 83
Nivolumab 2081 393 106 35 6 NA
Months after therapy initiation
time to autoimmunity
all immunotherapy drugs
P=0.2803 (matched)
P=0.0044 (unmatched)
autoimmune−free %
second_line
Lung num. at risk
matched
target therapy 407.0 125.8 44.0 14.0 6.2 2.4
all immuno 407 101 30 6 2 NA
unmatched
target therapy 6022 1958 747 335 166 83
all immuno 514 123 41 10 3 NA
Months after therapy initiation
time to autoimmunity
Pembrolizumab
P=0.2508 (matched)
P=0.6498 (unmatched)
autoimmune−free %
second_line
Lung num. at risk
matched
target therapy 99.0 29.1 10.2 1.9 0.8 0.2
Pembrolizumab 99 25 4 NA NA NA
unmatched
target therapy 6022 1958 747 335 166 83
Pembrolizumab 121 32 8 NA NA NA
Months after therapy initiation
time to autoimmunity
Nivolumab
P=0.0852 (matched)
P=0.0024 (unmatched)
autoimmune−free %
second_line
Lung num. at risk
matched
target therapy 279.0 84.7 33.7 14.3 8.3 4.2
Nivolumab 279 68 24 6 2 NA
unmatched
target therapy 6022 1958 747 335 166 83
Nivolumab 354 85 33 10 3 NA
Months after therapy initiation

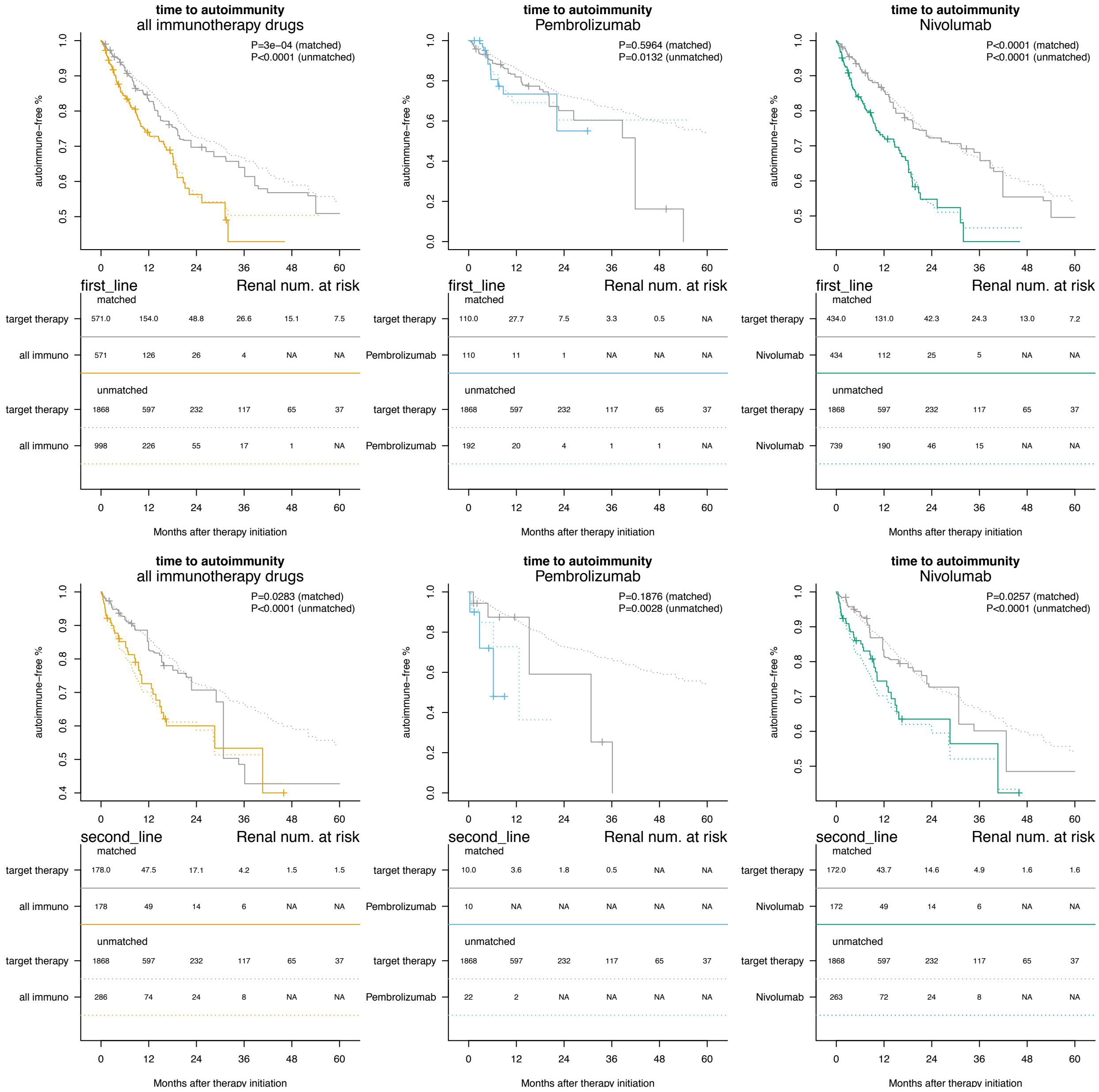
time to autoimmunity
all immunotherapy drugs
P=3e−04 (matched)
P<0.0001 (unmatched)
autoimmune−free %
first_line
Renal num. at risk
matched
target therapy 571.0 154.0 48.8 26.6 15.1 7.5
all immuno 571 126 26 4 NA NA
unmatched
target therapy 1868 597 232 117 65 37
all immuno 998 226 55 17 1 NA
Months after therapy initiation
time to autoimmunity
Pembrolizumab
P=0.5964 (matched)
P=0.0132 (unmatched)
autoimmune−free %
first_line
Renal num. at risk
matched
target therapy 110.0 27.7 7.5 3.3 0.5 NA
Pembrolizumab 110 11 1 NA NA NA
unmatched
target therapy 1868 597 232 117 65 37
Pembrolizumab 192 20 4 1 1 NA
Months after therapy initiation
time to autoimmunity
Nivolumab
P<0.0001 (matched)
P<0.0001 (unmatched)
autoimmune−free %
first_line
Renal num. at risk
matched
target therapy 434.0 131.0 42.3 24.3 13.0 7.2
Nivolumab 434 112 25 5 NA NA
unmatched
target therapy 1868 597 232 117 65 37
Nivolumab 739 190 46 15 NA NA
Months after therapy initiation
time to autoimmunity
all immunotherapy drugs
P=0.0283 (matched)
P<0.0001 (unmatched)
autoimmune−free %
second_line
Renal num. at risk
matched
target therapy 178.0 47.5 17.1 4.2 1.5 1.5
all immuno 178 49 14 6 NA NA
unmatched
target therapy 1868 597 232 117 65 37
all immuno 286 74 24 8 NA NA
Months after therapy initiation
time to autoimmunity
Pembrolizumab
P=0.1876 (matched)
P=0.0028 (unmatched)
autoimmune−free %
second_line
Renal num. at risk
matched
target therapy 10.0 3.6 1.8 0.5 NA NA
Pembrolizumab 10 NA NA NA NA NA
unmatched
target therapy 1868 597 232 117 65 37
Pembrolizumab 22 2 NA NA NA NA
Months after therapy initiation
time to autoimmunity
Nivolumab
P=0.0257 (matched)
P<0.0001 (unmatched)
autoimmune−free %
second_line
Renal num. at risk
matched
target therapy 172.0 43.7 14.6 4.9 1.6 1.6
Nivolumab 172 49 14 6 NA NA
unmatched
target therapy 1868 597 232 117 65 37
Nivolumab 263 72 24 8 NA NA
Months after therapy initiation

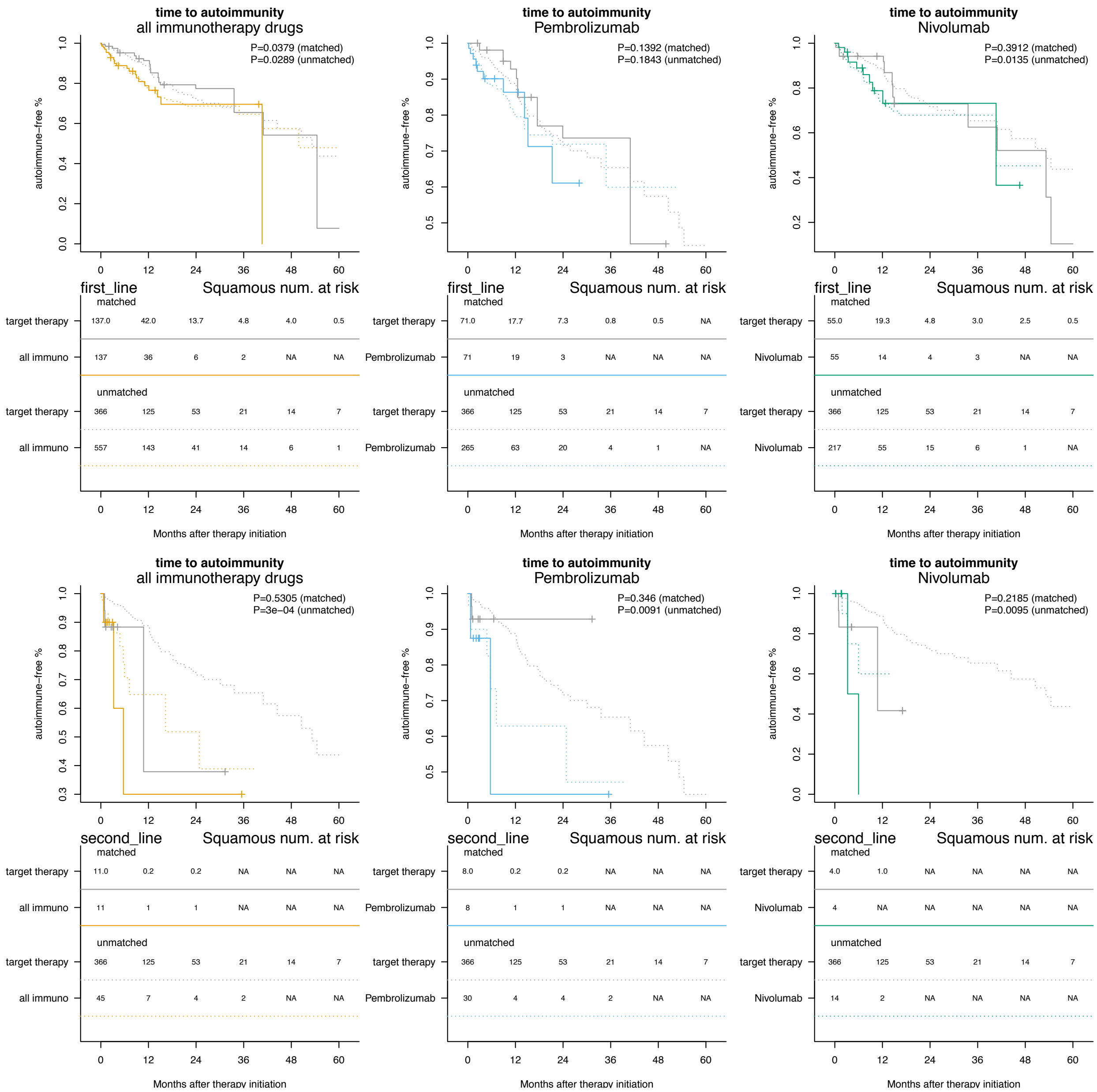
time to autoimmunity
all immunotherapy drugs
P=0.0379 (matched)
P=0.0289 (unmatched)
autoimmune−free %
first_line
Squamous num. at risk
matched
target therapy 137.0 42.0 13.7 4.8 4.0 0.5
all immuno 137 36 6 2 NA NA
unmatched
target therapy 366 125 53 21 14 7
all immuno 557 143 41 14 6 1
Months after therapy initiation
time to autoimmunity
Pembrolizumab
P=0.1392 (matched)
P=0.1843 (unmatched)
autoimmune−free %
first_line
Squamous num. at risk
matched
target therapy 71.0 17.7 7.3 0.8 0.5 NA
Pembrolizumab 71 19 3 NA NA NA
unmatched
target therapy 366 125 53 21 14 7
Pembrolizumab 265 63 20 4 1 NA
Months after therapy initiation
time to autoimmunity
Nivolumab
P=0.3912 (matched)
P=0.0135 (unmatched)
autoimmune−free %
first_line
Squamous num. at risk
matched
target therapy 55.0 19.3 4.8 3.0 2.5 0.5
Nivolumab 55 14 4 3 NA NA
unmatched
target therapy 366 125 53 21 14 7
Nivolumab 217 55 15 6 1 NA
Months after therapy initiation
time to autoimmunity
all immunotherapy drugs
P=0.5305 (matched)
P=3e−04 (unmatched)
autoimmune−free %
second_line
Squamous num. at risk
matched
target therapy 11.0 0.2 0.2 NA NA NA
all immuno 11 1 1 NA NA NA
unmatched
target therapy 366 125 53 21 14 7
all immuno 45 7 4 2 NA NA
Months after therapy initiation
time to autoimmunity
Pembrolizumab
P=0.346 (matched)
P=0.0091 (unmatched)
autoimmune−free %
second_line
Squamous num. at risk
matched
target therapy 8.0 0.2 0.2 NA NA NA
Pembrolizumab 8 1 1 NA NA NA
unmatched
target therapy 366 125 53 21 14 7
Pembrolizumab 30 4 4 2 NA NA
Months after therapy initiation
time to autoimmunity
Nivolumab
P=0.2185 (matched)
P=0.0095 (unmatched)
autoimmune−free %
second_line
Squamous num. at risk
matched
target therapy 4.0 1.0 NA NA NA NA
Nivolumab 4 NA NA NA NA NA
unmatched
target therapy 366 125 53 21 14 7
Nivolumab 14 2 NA NA NA NA
Months after therapy initiation

Sex Stratification

We conducted a stratified analysis based on sex. The risk of developing autoimmune adverse events in the immunotherapy group is similar in male and female patients (Figure S11).

Figure S11. Results stratified by gender.

Chemotherapy

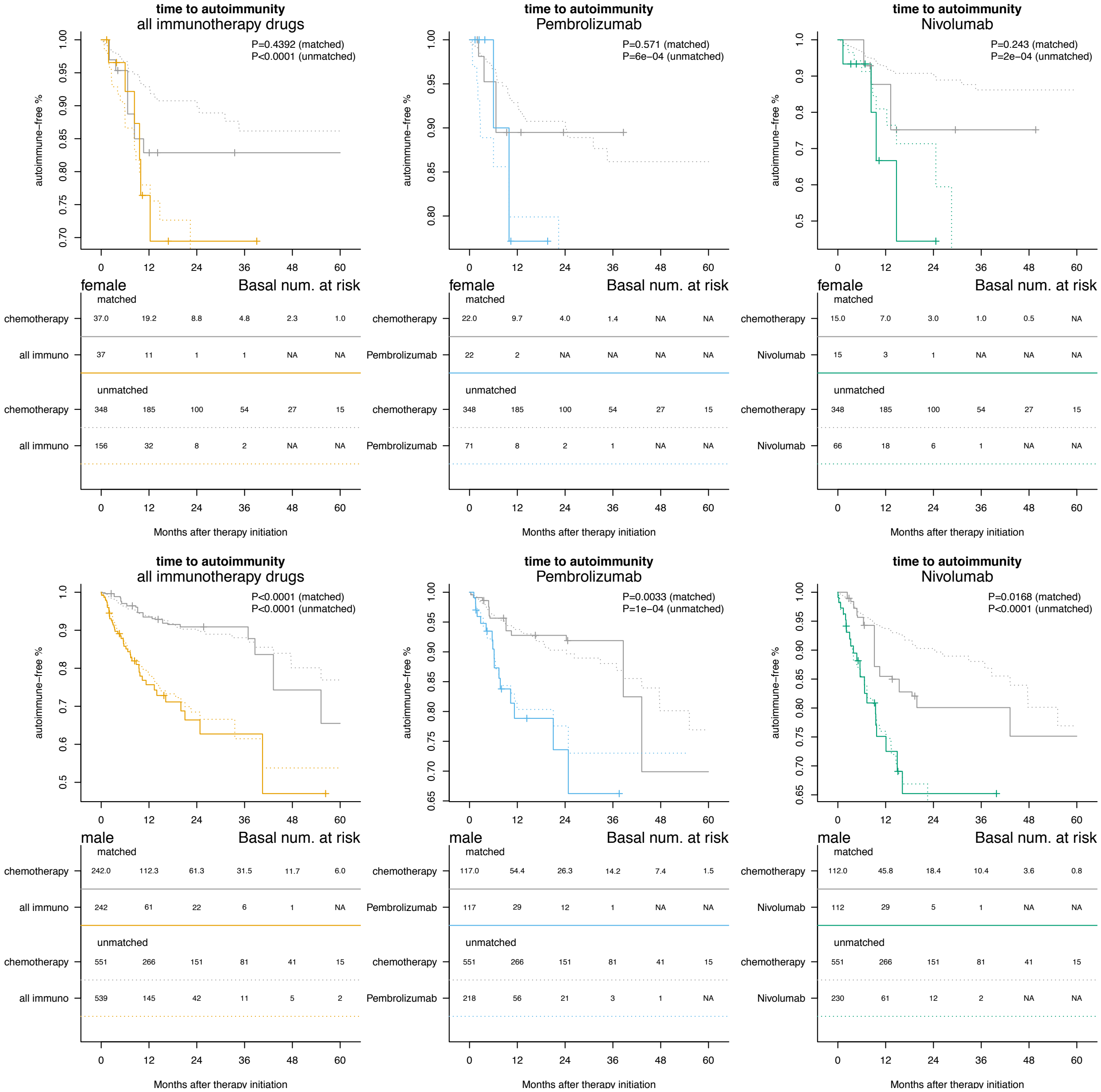

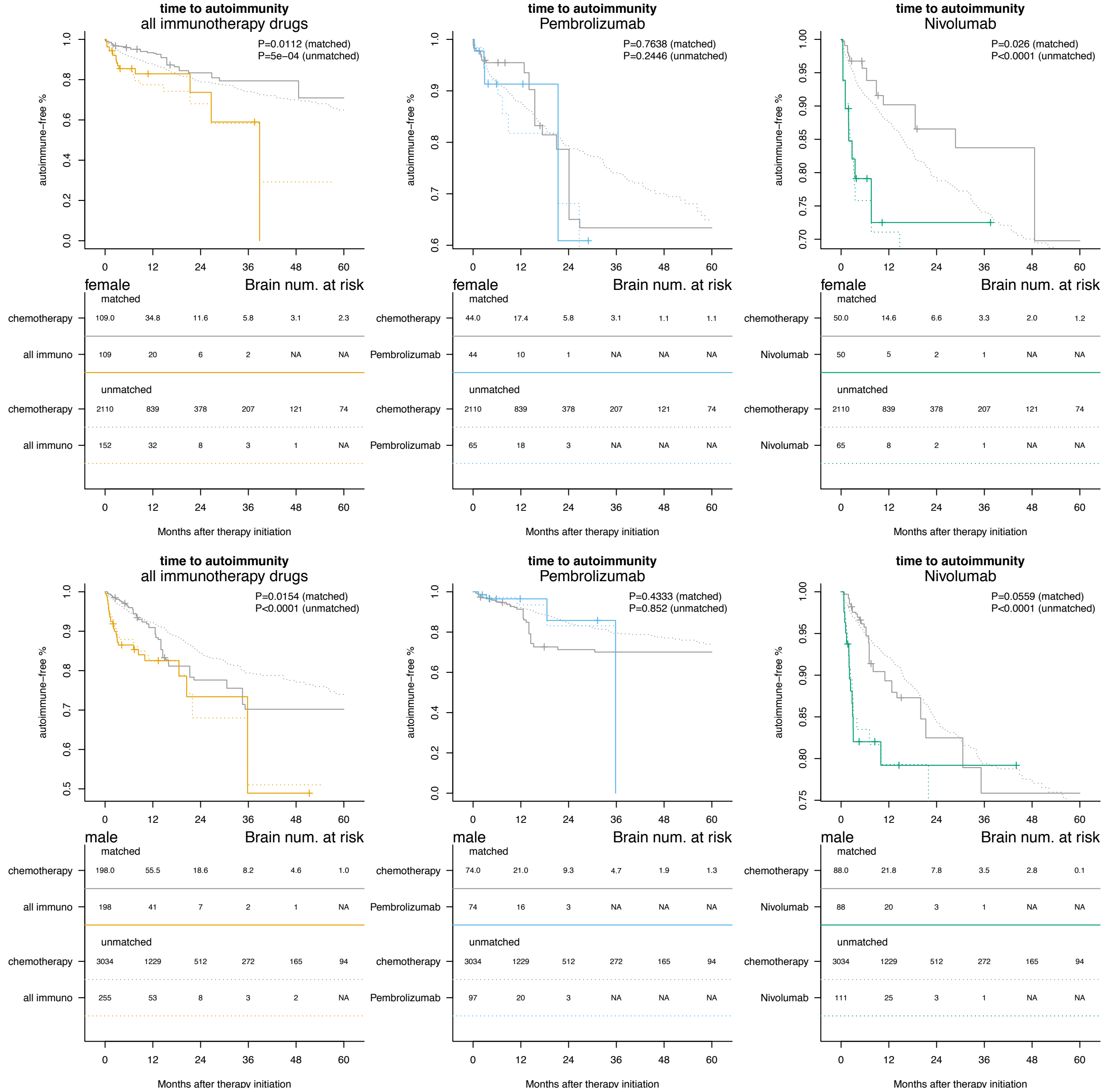
time to autoimmunity
all immunotherapy drugs
P=0.0112 (matched)
P=5e−04 (unmatched)
autoimmune−free %
female
Brain num. at risk
matched
chemotherapy 109.0 34.8 11.6 5.8 3.1 2.3
all immuno 109 20 6 2 NA NA
unmatched
chemotherapy 2110 839 378 207 121 74
all immuno 152 32 8 3 1 NA
Months after therapy initiation
time to autoimmunity
Pembrolizumab
P=0.7638 (matched)
P=0.2446 (unmatched)
autoimmune−free %
female
Brain num. at risk
matched
chemotherapy 44.0 17.4 5.8 3.1 1.1 1.1
Pembrolizumab 44 10 1 NA NA NA
unmatched
chemotherapy 2110 839 378 207 121 74
Pembrolizumab 65 18 3 NA NA NA
Months after therapy initiation
time to autoimmunity
Nivolumab
P=0.026 (matched)
P<0.0001 (unmatched)
autoimmune−free %
female
Brain num. at risk
matched
chemotherapy 50.0 14.6 6.6 3.3 2.0 1.2
Nivolumab 50 5 2 1 NA NA
unmatched
chemotherapy 2110 839 378 207 121 74
Nivolumab 65 8 2 1 NA NA
Months after therapy initiation
time to autoimmunity
all immunotherapy drugs
P=0.0154 (matched)
P<0.0001 (unmatched)
autoimmune−free %
male
Brain num. at risk
matched
chemotherapy 198.0 55.5 18.6 8.2 4.6 1.0
all immuno 198 41 7 2 1 NA
unmatched
chemotherapy 3034 1229 512 272 165 94
all immuno 255 53 8 3 2 NA
Months after therapy initiation
time to autoimmunity
Pembrolizumab
P=0.4333 (matched)
P=0.852 (unmatched)
autoimmune−free %
male
Brain num. at risk
matched
chemotherapy 74.0 21.0 9.3 4.7 1.9 1.3
Pembrolizumab 74 16 3 NA NA NA
unmatched
chemotherapy 3034 1229 512 272 165 94
Pembrolizumab 97 20 3 NA NA NA
Months after therapy initiation
time to autoimmunity
Nivolumab
P=0.0559 (matched)
P<0.0001 (unmatched)
autoimmune−free %
male
Brain num. at risk
matched
chemotherapy 88.0 21.8 7.8 3.5 2.8 0.1
Nivolumab 88 20 3 1 NA NA
unmatched
chemotherapy 3034 1229 512 272 165 94
Nivolumab 111 25 3 1 NA NA
Months after therapy initiation

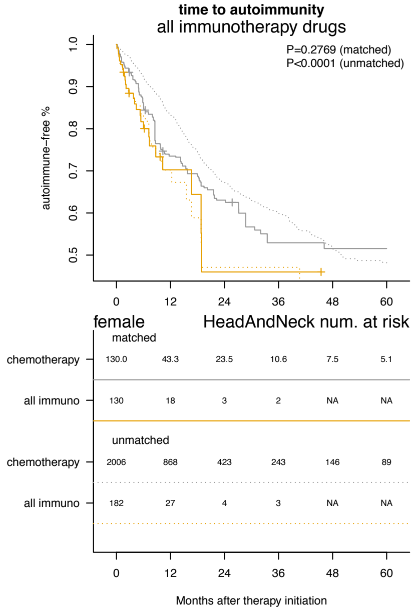
time to autoimmunity
all immunotherapy drugs
P=0.2769 (matched)
P<0.0001 (unmatched)
autoimmune−free %
female
HeadAndNeck num. at risk
matched
chemotherapy 130.0 43.3 23.5 10.6 7.5 5.1
all immuno 130 18 3 2 NA NA
unmatched
chemotherapy 2006 868 423 243 146 89
all immuno 182 27 4 3 NA NA
0 12 24 36 48 60
Months after therapy initiation

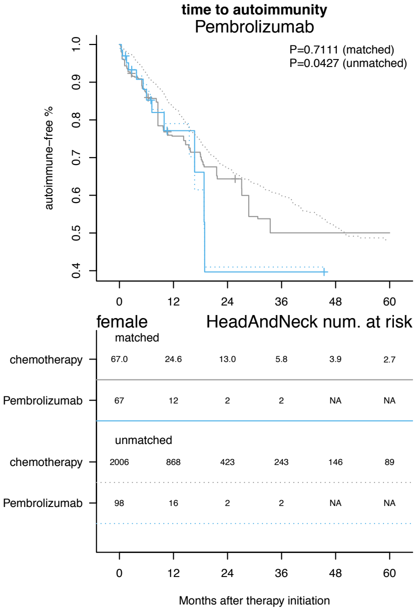
time to autoimmunity
Pembrolizumab
P=0.7111 (matched)
P=0.0427 (unmatched)
autoimmune−free %
female
HeadAndNeck num. at risk
matched
chemotherapy 67.0 24.6 13.0 5.8 3.9 2.7
Pembrolizumab 67 12 2 2 NA NA
unmatched
chemotherapy 2006 868 423 243 146 89
Pembrolizumab 98 16 2 2 NA NA
0 12 24 36 48 60
Months after therapy initiation

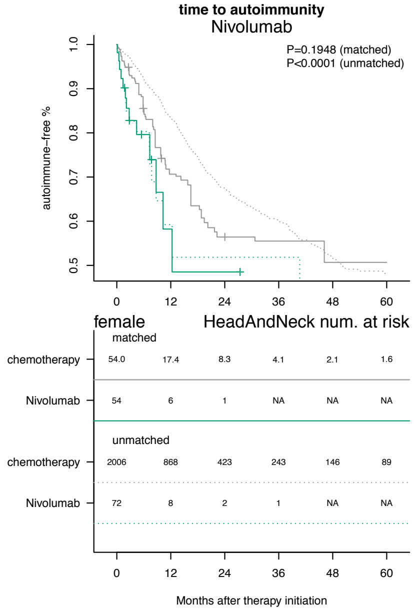
time to autoimmunity
Nivolumab
P=0.1948 (matched)
P<0.0001 (unmatched)
autoimmune−free %
female
HeadAndNeck num. at risk
matched
chemotherapy 54.0 17.4 8.3 4.1 2.1 1.6
Nivolumab 54 6 1 NA NA NA
unmatched
chemotherapy 2006 868 423 243 146 89
Nivolumab 72 8 2 1 NA NA
0 12 24 36 48 60
Months after therapy initiation

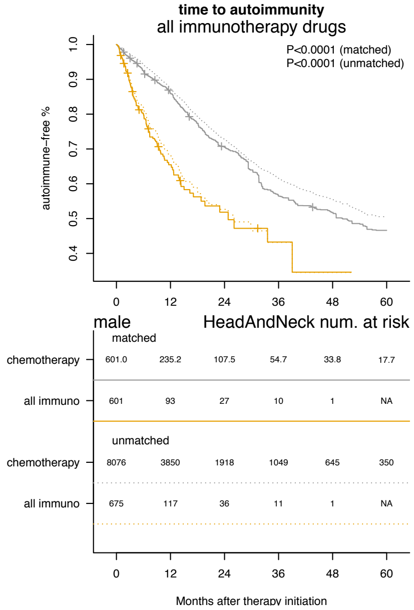
time to autoimmunity
all immunotherapy drugs
P<0.0001 (matched)
P<0.0001 (unmatched)
autoimmune−free %
male
HeadAndNeck num. at risk
matched
chemotherapy 601.0 235.2 107.5 54.7 33.8 17.7
all immuno 601 93 27 10 1 NA
unmatched
chemotherapy 8076 3850 1918 1049 645 350
all immuno 675 117 36 11 1 NA
0 12 24 36 48 60
Months after therapy initiation

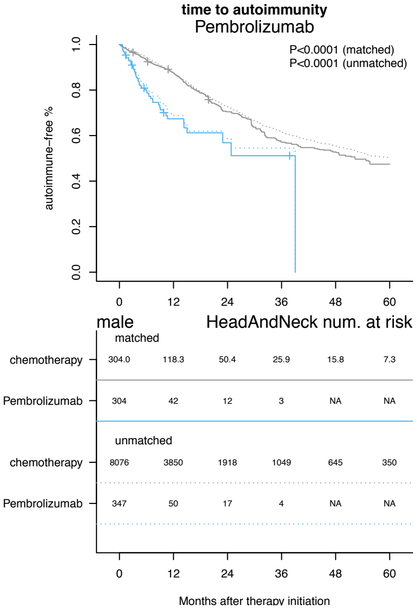
time to autoimmunity
Pembrolizumab
P<0.0001 (matched)
P<0.0001 (unmatched)
autoimmune−free %
male
HeadAndNeck num. at risk
matched
chemotherapy 304.0 118.3 50.4 25.9 15.8 7.3
Pembrolizumab 304 42 12 3 NA NA
unmatched
chemotherapy 8076 3850 1918 1049 645 350
Pembrolizumab 347 50 17 4 NA NA
0 12 24 36 48 60
Months after therapy initiation

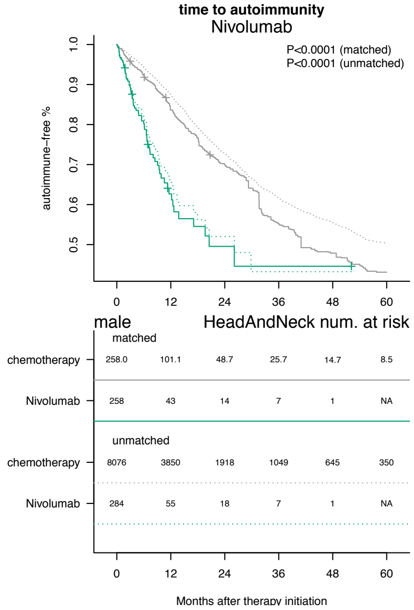
time to autoimmunity
Nivolumab
P<0.0001 (matched)
P<0.0001 (unmatched)
autoimmune−free %
male
HeadAndNeck num. at risk
matched
chemotherapy 258.0 101.1 48.7 25.7 14.7 8.5
Nivolumab 258 43 14 7 1 NA
unmatched
chemotherapy 8076 3850 1918 1049 645 350
Nivolumab 284 55 18 7 1 NA
0 12 24 36 48 60
Months after therapy initiation

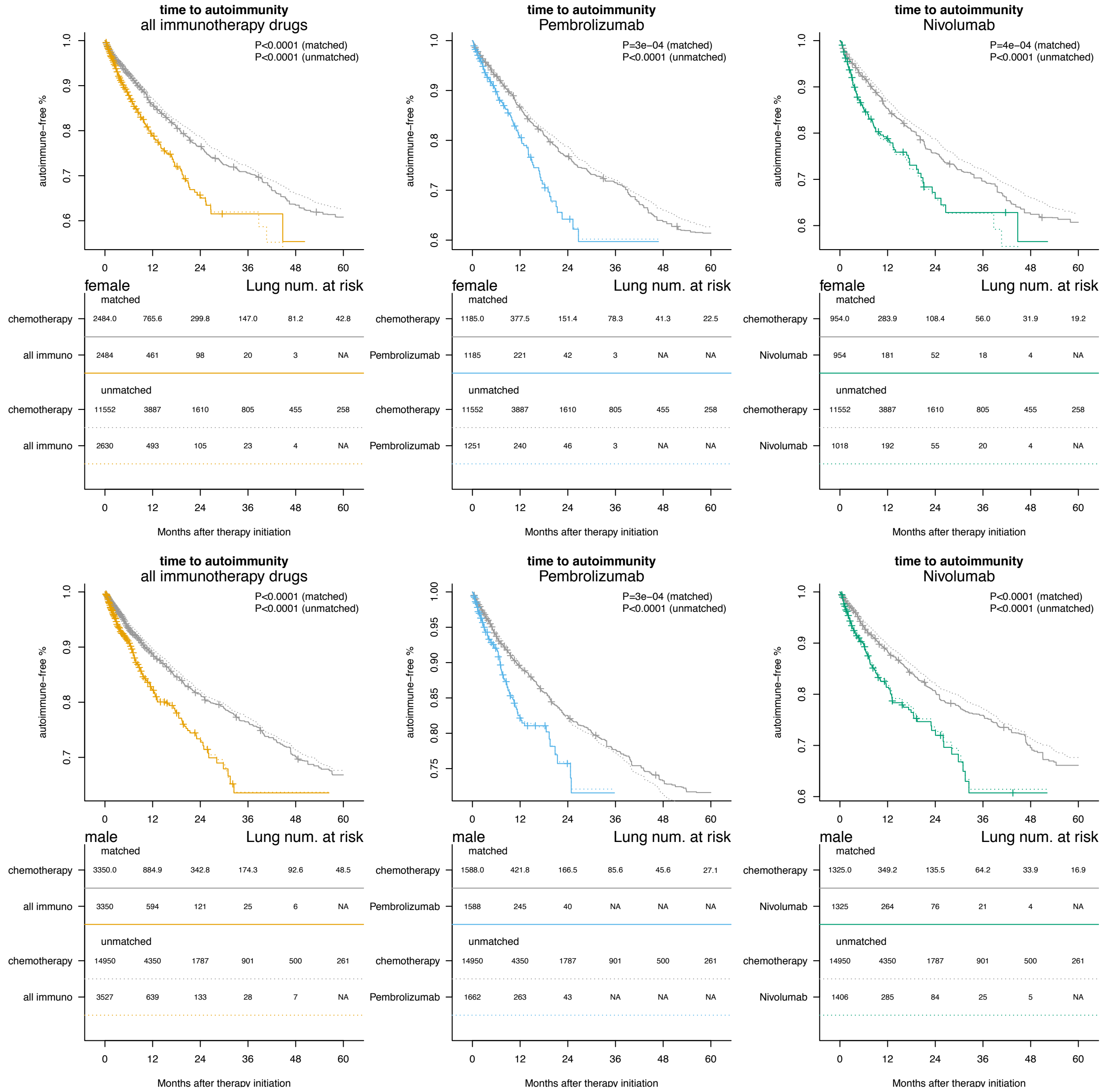
time to autoimmunity
all immunotherapy drugs
P<0.0001 (matched)
P<0.0001 (unmatched)
autoimmune-free %
female
Lung num. at risk
matched
chemotherapy 2484.0 765.6 299.8 147.0 81.2 42.8
all immuno 2484 461 98 20 3 NA
unmatched
chemotherapy 11552 3887 1610 805 455 258
all immuno 2630 493 105 23 4 NA
Months after therapy initiation
time to autoimmunity
Pembrolizumab
P=3e−04 (matched)
P<0.0001 (unmatched)
autoimmune-free %
female
Lung num. at risk
matched
chemotherapy 1185.0 377.5 151.4 78.3 41.3 22.5
Pembrolizumab 1185 221 42 3 NA NA
unmatched
chemotherapy 11552 3887 1610 805 455 258
Pembrolizumab 1251 240 46 3 NA NA
Months after therapy initiation
time to autoimmunity
Nivolumab
P=4e−04 (matched)
P<0.0001 (unmatched)
autoimmune-free %
female
Lung num. at risk
matched
chemotherapy 954.0 283.9 108.4 56.0 31.9 19.2
Nivolumab 954 181 52 18 4 NA
unmatched
chemotherapy 11552 3887 1610 805 455 258
Nivolumab 1018 192 55 20 4 NA
Months after therapy initiation
time to autoimmunity
all immunotherapy drugs
P<0.0001 (matched)
P<0.0001 (unmatched)
autoimmune-free %
male
Lung num. at risk
matched
chemotherapy 3350.0 884.9 342.8 174.3 92.6 48.5
all immuno 3350 594 121 25 6 NA
unmatched
chemotherapy 14950 4350 1787 901 500 261
all immuno 3527 639 133 28 7 NA
Months after therapy initiation
time to autoimmunity
Pembrolizumab
P=3e−04 (matched)
P<0.0001 (unmatched)
autoimmune-free %
male
Lung num. at risk
matched
chemotherapy 1588.0 421.8 166.5 85.6 45.6 27.1
Pembrolizumab 1588 245 40 NA NA NA
unmatched
chemotherapy 14950 4350 1787 901 500 261
Pembrolizumab 1662 263 43 NA NA NA
Months after therapy initiation
time to autoimmunity
Nivolumab
P<0.0001 (matched)
P<0.0001 (unmatched)
autoimmune-free %
male
Lung num. at risk
matched
chemotherapy 1325.0 349.2 135.5 64.2 33.9 16.9
Nivolumab 1325 264 76 21 4 NA
unmatched
chemotherapy 14950 4350 1787 901 500 261
Nivolumab 1406 285 84 25 5 NA
Months after therapy initiation

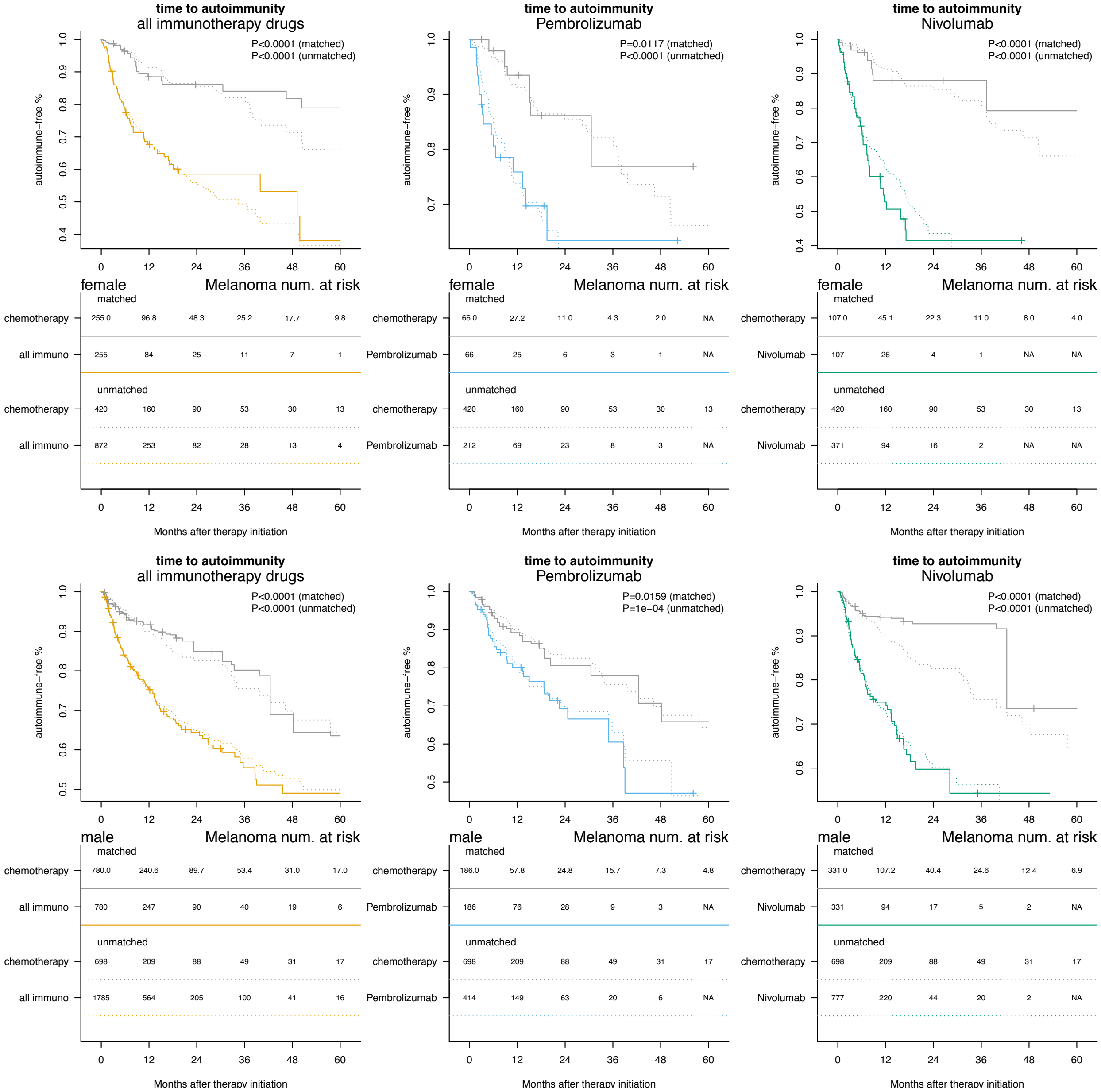
time to autoimmunity
all immunotherapy drugs
P<0.0001 (matched)
P<0.0001 (unmatched)
autoimmune−free %
female
Melanoma num. at risk
matched
chemotherapy 255.0 96.8 48.3 25.2 17.7 9.8
all immuno 255 84 25 11 7 1
unmatched
chemotherapy 420 160 90 53 30 13
all immuno 872 253 82 28 13 4
Months after therapy initiation
time to autoimmunity
Pembrolizumab
P=0.0117 (matched)
P<0.0001 (unmatched)
autoimmune−free %
female
Melanoma num. at risk
matched
chemotherapy 66.0 27.2 11.0 4.3 2.0 NA
Pembrolizumab 66 25 6 3 1 NA
unmatched
chemotherapy 420 160 90 53 30 13
Pembrolizumab 212 69 23 8 3 NA
Months after therapy initiation
time to autoimmunity
Nivolumab
P<0.0001 (matched)
P<0.0001 (unmatched)
autoimmune−free %
female
Melanoma num. at risk
matched
chemotherapy 107.0 45.1 22.3 11.0 8.0 4.0
Nivolumab 107 26 4 1 NA NA
unmatched
chemotherapy 420 160 90 53 30 13
Nivolumab 371 94 16 2 NA NA
Months after therapy initiation
time to autoimmunity
all immunotherapy drugs
P<0.0001 (matched)
P<0.0001 (unmatched)
autoimmune−free %
male
Melanoma num. at risk
matched
chemotherapy 780.0 240.6 89.7 53.4 31.0 17.0
all immuno 780 247 90 40 19 6
unmatched
chemotherapy 698 209 88 49 31 17
all immuno 1785 564 205 100 41 16
Months after therapy initiation
time to autoimmunity
Pembrolizumab
P=0.0159 (matched)
P=1e−04 (unmatched)
autoimmune−free %
male
Melanoma num. at risk
matched
chemotherapy 186.0 57.8 24.8 15.7 7.3 4.8
Pembrolizumab 186 76 28 9 3 NA
unmatched
chemotherapy 698 209 88 49 31 17
Pembrolizumab 414 149 63 20 6 NA
Months after therapy initiation
time to autoimmunity
Nivolumab
P<0.0001 (matched)
P<0.0001 (unmatched)
autoimmune−free %
male
Melanoma num. at risk
matched
chemotherapy 331.0 107.2 40.4 24.6 12.4 6.9
Nivolumab 331 94 17 5 2 NA
unmatched
chemotherapy 698 209 88 49 31 17
Nivolumab 777 220 44 20 2 NA
Months after therapy initiation

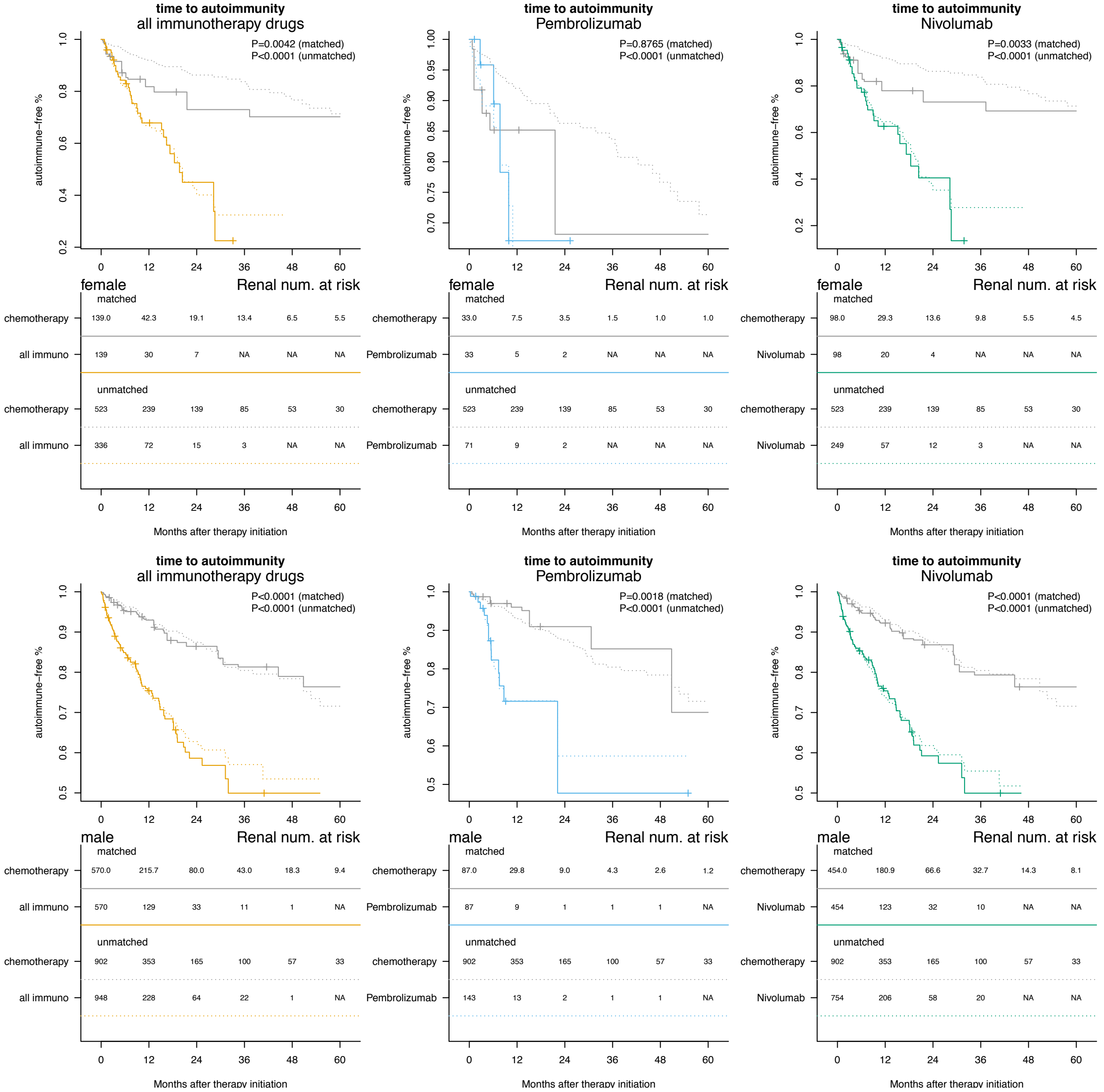
time to autoimmunity
all immunotherapy drugs
P=0.0042 (matched)
P<0.0001 (unmatched)
time to autoimmunity
Pembrolizumab
P=0.8765 (matched)
P<0.0001 (unmatched)
time to autoimmunity
Nivolumab
P=0.0033 (matched)
P<0.0001 (unmatched)
autoimmune−free %
female
Renal num. at risk
matched
chemotherapy 139.0 42.3 19.1 13.4 6.5 5.5
all immuno 139 30 7 NA NA NA
unmatched
chemotherapy 523 239 139 85 53 30
all immuno 336 72 15 3 NA NA
chemotherapy 33.0 7.5 3.5 1.5 1.0 1.0
Pembrolizumab 33 5 2 NA NA NA
chemotherapy 523 239 139 85 53 30
Pembrolizumab 71 9 2 NA NA NA
chemotherapy 98.0 29.3 13.6 9.8 5.5 4.5
Nivolumab 98 20 4 NA NA NA
chemotherapy 523 239 139 85 53 30
Nivolumab 249 57 12 3 NA NA
Months after therapy initiation
time to autoimmunity
all immunotherapy drugs
P<0.0001 (matched)
P<0.0001 (unmatched)
time to autoimmunity
Pembrolizumab
P=0.0018 (matched)
P<0.0001 (unmatched)
time to autoimmunity
Nivolumab
P<0.0001 (matched)
P<0.0001 (unmatched)
male
Renal num. at risk
matched
chemotherapy 570.0 215.7 80.0 43.0 18.3 9.4
all immuno 570 129 33 11 1 NA
unmatched
chemotherapy 902 353 165 100 57 33
all immuno 948 228 64 22 1 NA
chemotherapy 87.0 29.8 9.0 4.3 2.6 1.2
Pembrolizumab 87 9 1 1 1 NA
chemotherapy 902 353 165 100 57 33
Pembrolizumab 143 13 2 1 1 NA
chemotherapy 454.0 180.9 66.6 32.7 14.3 8.1
Nivolumab 454 123 32 10 NA NA
chemotherapy 902 353 165 100 57 33
Nivolumab 754 206 58 20 NA NA
Months after therapy initiation

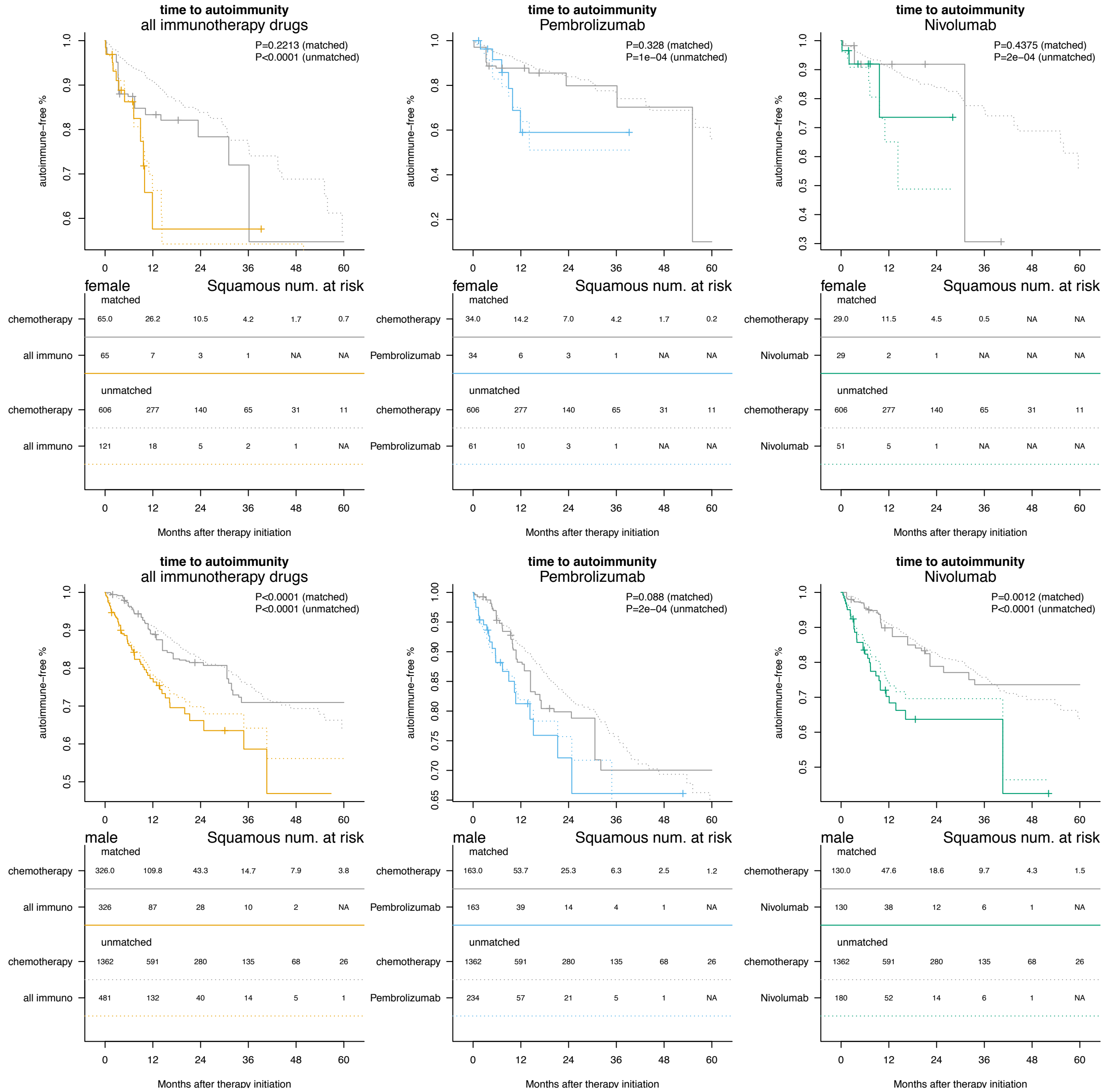
time to autoimmunity
all immunotherapy drugs
P=0.2213 (matched)
P<0.0001 (unmatched)
autoimmune−free %
female
Squamous num. at risk
matched
chemotherapy 65.0 26.2 10.5 4.2 1.7 0.7
all immuno 65 7 3 1 NA NA
unmatched
chemotherapy 606 277 140 65 31 11
all immuno 121 18 5 2 1 NA
Months after therapy initiation
time to autoimmunity
Pembrolizumab
P=0.328 (matched)
P=1e−04 (unmatched)
female
Squamous num. at risk
matched
chemotherapy 34.0 14.2 7.0 4.2 1.7 0.2
Pembrolizumab 34 6 3 1 NA NA
unmatched
chemotherapy 606 277 140 65 31 11
Pembrolizumab 61 10 3 1 NA NA
time to autoimmunity
Nivolumab
P=0.4375 (matched)
P=2e−04 (unmatched)
female
Squamous num. at risk
matched
chemotherapy 29.0 11.5 4.5 0.5 NA NA
Nivolumab 29 2 1 NA NA NA
unmatched
chemotherapy 606 277 140 65 31 11
Nivolumab 51 5 1 NA NA NA
time to autoimmunity
all immunotherapy drugs
P<0.0001 (matched)
P<0.0001 (unmatched)
male
Squamous num. at risk
matched
chemotherapy 326.0 109.8 43.3 14.7 7.9 3.8
all immuno 326 87 28 10 2 NA
unmatched
chemotherapy 1362 591 280 135 68 26
all immuno 481 132 40 14 5 1
time to autoimmunity
Pembrolizumab
P=0.088 (matched)
P=2e−04 (unmatched)
male
Squamous num. at risk
matched
chemotherapy 163.0 53.7 25.3 6.3 2.5 1.2
Pembrolizumab 163 39 14 4 1 NA
unmatched
chemotherapy 1362 591 280 135 68 26
Pembrolizumab 234 57 21 5 1 NA
time to autoimmunity
Nivolumab
P=0.0012 (matched)
P<0.0001 (unmatched)
male
Squamous num. at risk
matched
chemotherapy 130.0 47.6 18.6 9.7 4.3 1.5
Nivolumab 130 38 12 6 1 NA
unmatched
chemotherapy 1362 591 280 135 68 26
Nivolumab 180 52 14 6 1 NA
Months after therapy initiation

Targeted therapy

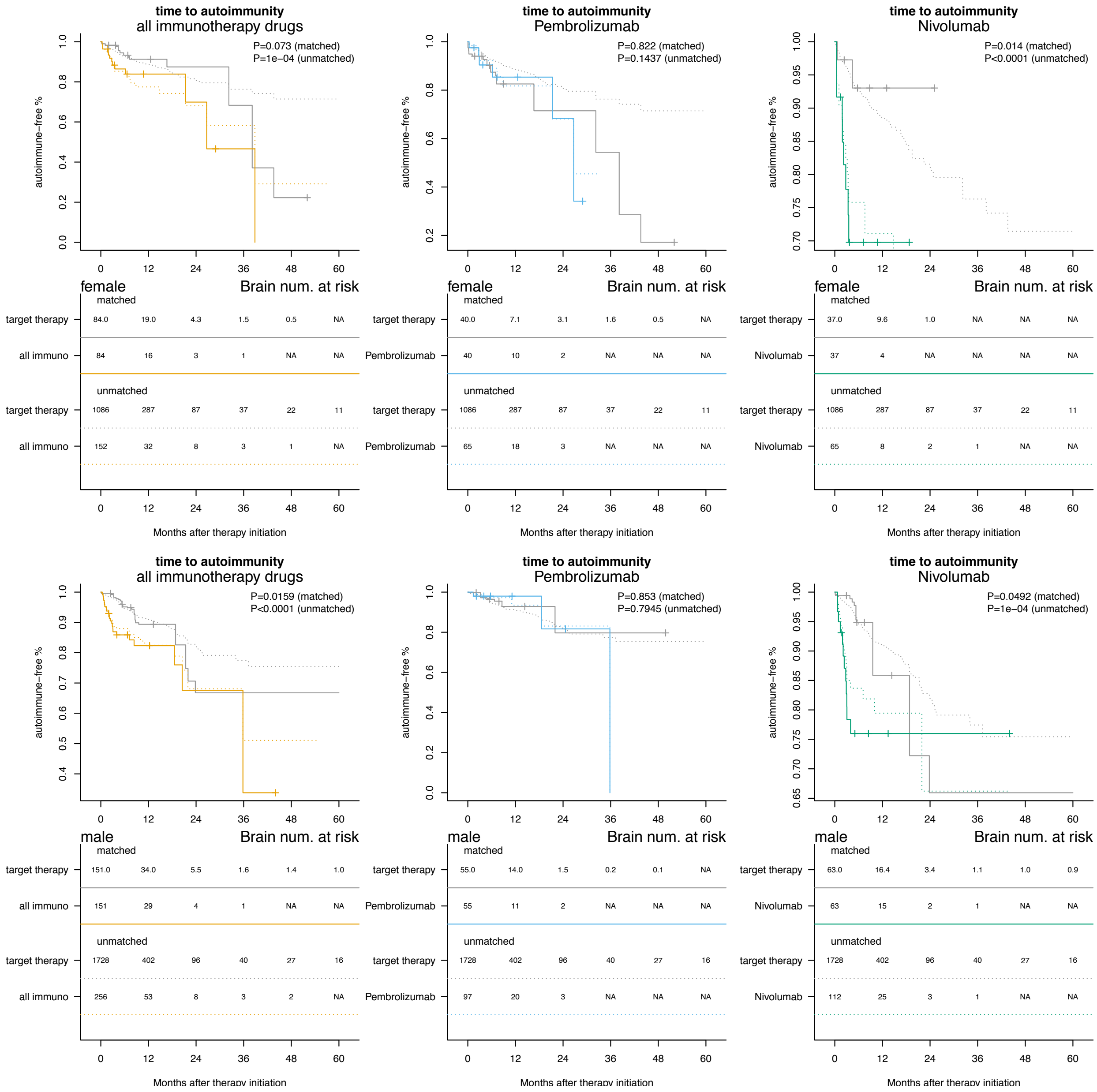

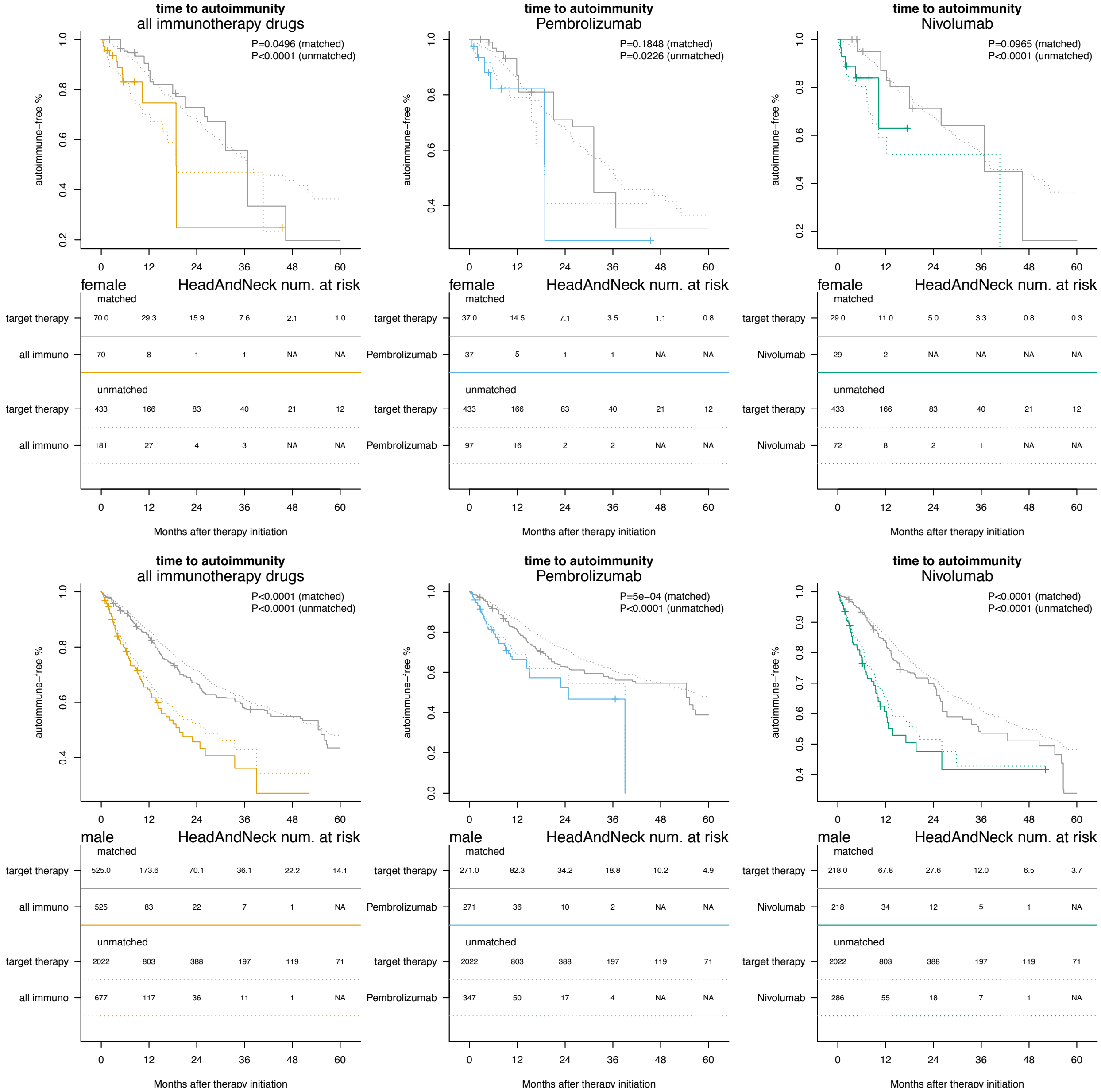

time to autoimmunity
all immunotherapy drugs
P=0.0496 (matched)
P<0.0001 (unmatched)
autoimmune−free %
female
HeadAndNeck num. at risk
matched
target therapy 70.0 29.3 15.9 7.6 2.1 1.0
all immuno 70 8 1 1 NA NA
unmatched
target therapy 433 166 83 40 21 12
all immuno 181 27 4 3 NA NA
Months after therapy initiation
time to autoimmunity
Pembrolizumab
P=0.1848 (matched)
P=0.0226 (unmatched)
female
HeadAndNeck num. at risk
matched
target therapy 37.0 14.5 7.1 3.5 1.1 0.8
Pembrolizumab 37 5 1 1 NA NA
unmatched
target therapy 433 166 83 40 21 12
Pembrolizumab 97 16 2 2 NA NA
time to autoimmunity
Nivolumab
P=0.0965 (matched)
P<0.0001 (unmatched)
female
HeadAndNeck num. at risk
matched
target therapy 29.0 11.0 5.0 3.3 0.8 0.3
Nivolumab 29 2 NA NA NA NA
unmatched
target therapy 433 166 83 40 21 12
Nivolumab 72 8 2 1 NA NA
time to autoimmunity
all immunotherapy drugs
P<0.0001 (matched)
P<0.0001 (unmatched)
male
HeadAndNeck num. at risk
matched
target therapy 525.0 173.6 70.1 36.1 22.2 14.1
all immuno 525 83 22 7 1 NA
unmatched
target therapy 2022 803 388 197 119 71
all immuno 677 117 36 11 1 NA
time to autoimmunity
Pembrolizumab
P=5e−04 (matched)
P<0.0001 (unmatched)
male
HeadAndNeck num. at risk
matched
target therapy 271.0 82.3 34.2 18.8 10.2 4.9
Pembrolizumab 271 36 10 2 NA NA
unmatched
target therapy 2022 803 388 197 119 71
Pembrolizumab 347 50 17 4 NA NA
time to autoimmunity
Nivolumab
P<0.0001 (matched)
P<0.0001 (unmatched)
male
HeadAndNeck num. at risk
matched
target therapy 218.0 67.8 27.6 12.0 6.5 3.7
Nivolumab 218 34 12 5 1 NA
unmatched
target therapy 2022 803 388 197 119 71
Nivolumab 286 55 18 7 1 NA
Months after therapy initiation

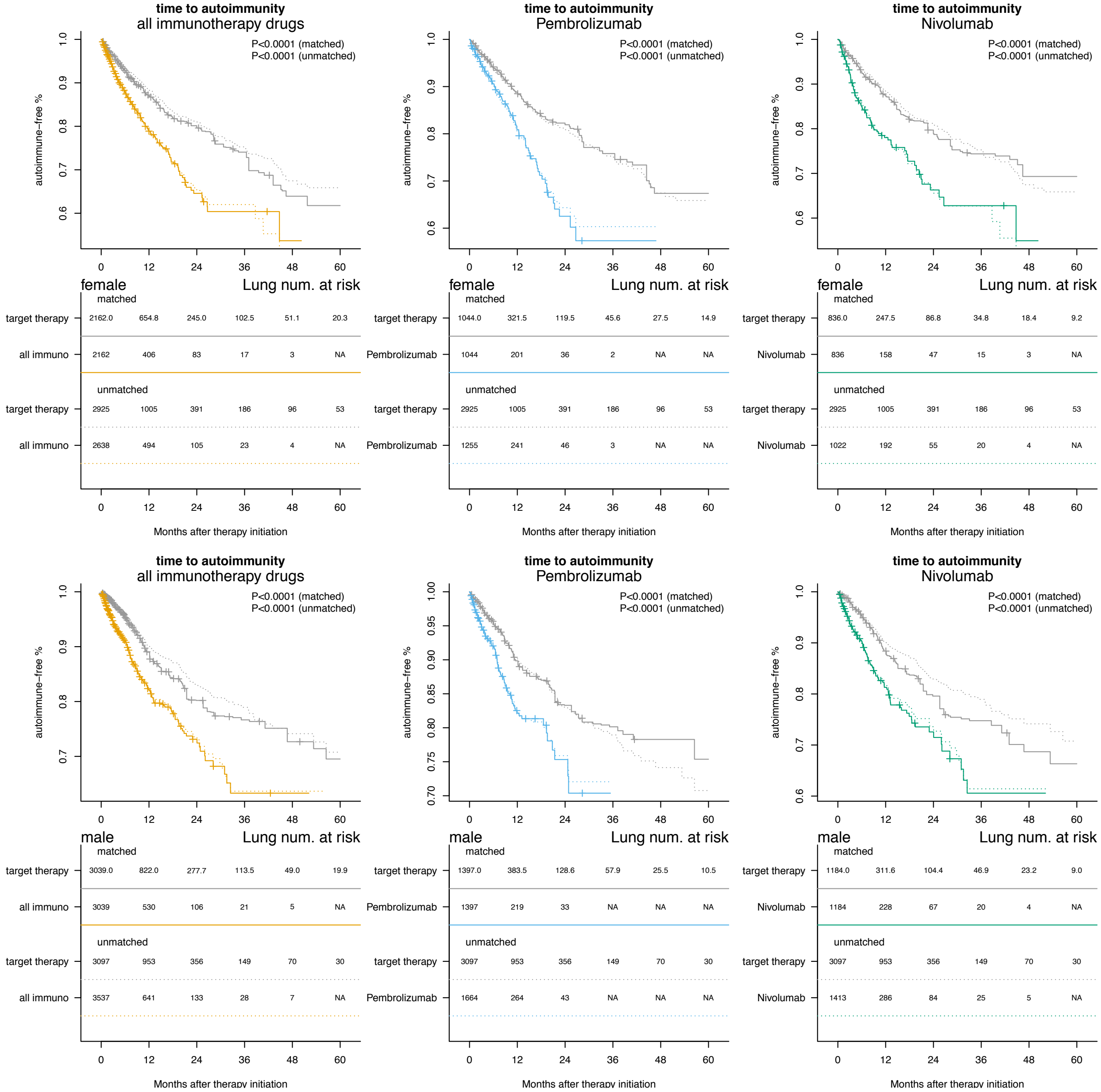
time to autoimmunity
all immunotherapy drugs
P<0.0001 (matched)
P<0.0001 (unmatched)
autoimmune−free %
female
Lung num. at risk
matched
target therapy 2162.0 654.8 245.0 102.5 51.1 20.3
all immuno 2162 406 83 17 3 NA
unmatched
target therapy 2925 1005 391 186 96 53
all immuno 2638 494 105 23 4 NA
Months after therapy initiation
time to autoimmunity
Pembrolizumab
P<0.0001 (matched)
P<0.0001 (unmatched)
autoimmune−free %
female
Lung num. at risk
matched
target therapy 1044.0 321.5 119.5 45.6 27.5 14.9
Pembrolizumab 1044 201 36 2 NA NA
unmatched
target therapy 2925 1005 391 186 96 53
Pembrolizumab 1255 241 46 3 NA NA
Months after therapy initiation
time to autoimmunity
Nivolumab
P<0.0001 (matched)
P<0.0001 (unmatched)
autoimmune−free %
female
Lung num. at risk
matched
target therapy 836.0 247.5 86.8 34.8 18.4 9.2
Nivolumab 836 158 47 15 3 NA
unmatched
target therapy 2925 1005 391 186 96 53
Nivolumab 1022 192 55 20 4 NA
Months after therapy initiation
time to autoimmunity
all immunotherapy drugs
P<0.0001 (matched)
P<0.0001 (unmatched)
autoimmune−free %
male
Lung num. at risk
matched
target therapy 3039.0 822.0 277.7 113.5 49.0 19.9
all immuno 3039 530 106 21 5 NA
unmatched
target therapy 3097 953 356 149 70 30
all immuno 3537 641 133 28 7 NA
Months after therapy initiation
time to autoimmunity
Pembrolizumab
P<0.0001 (matched)
P<0.0001 (unmatched)
autoimmune−free %
male
Lung num. at risk
matched
target therapy 1397.0 383.5 128.6 57.9 25.5 10.5
Pembrolizumab 1397 219 33 NA NA NA
unmatched
target therapy 3097 953 356 149 70 30
Pembrolizumab 1664 264 43 NA NA NA
Months after therapy initiation
time to autoimmunity
Nivolumab
P<0.0001 (matched)
P<0.0001 (unmatched)
autoimmune−free %
male
Lung num. at risk
matched
target therapy 1184.0 311.6 104.4 46.9 23.2 9.0
Nivolumab 1184 228 67 20 4 NA
unmatched
target therapy 3097 953 356 149 70 30
Nivolumab 1413 286 84 25 5 NA
Months after therapy initiation

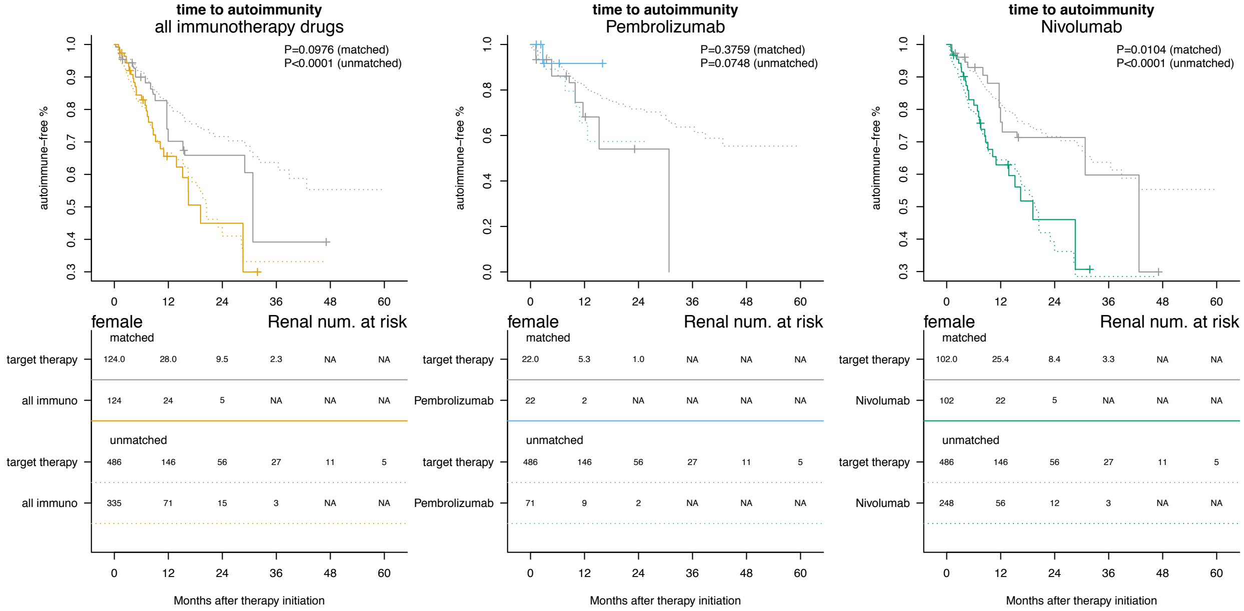

time to autoimmunity
all immunotherapy drugs
P=0.0976 (matched)
P<0.0001 (unmatched)
time to autoimmunity
Pembrolizumab
P=0.3759 (matched)
P=0.0748 (unmatched)
time to autoimmunity
Nivolumab
P=0.0104 (matched)
P<0.0001 (unmatched)
autoimmune−free %
female
Renal num. at risk
matched
target therapy 124.0 28.0 9.5 2.3 NA NA
all immuno 124 24 5 NA NA NA
unmatched
target therapy 486 146 56 27 11 5
all immuno 335 71 15 3 NA NA
matched
target therapy 22.0 5.3 1.0 NA NA NA
Pembrolizumab 22 2 NA NA NA NA
unmatched
target therapy 486 146 56 27 11 5
Pembrolizumab 71 9 2 NA NA NA
matched
target therapy 102.0 25.4 8.4 3.3 NA NA
Nivolumab 102 22 5 NA NA NA
unmatched
target therapy 486 146 56 27 11 5
Nivolumab 248 56 12 3 NA NA
0 12 24 36 48 60
Months after therapy initiation

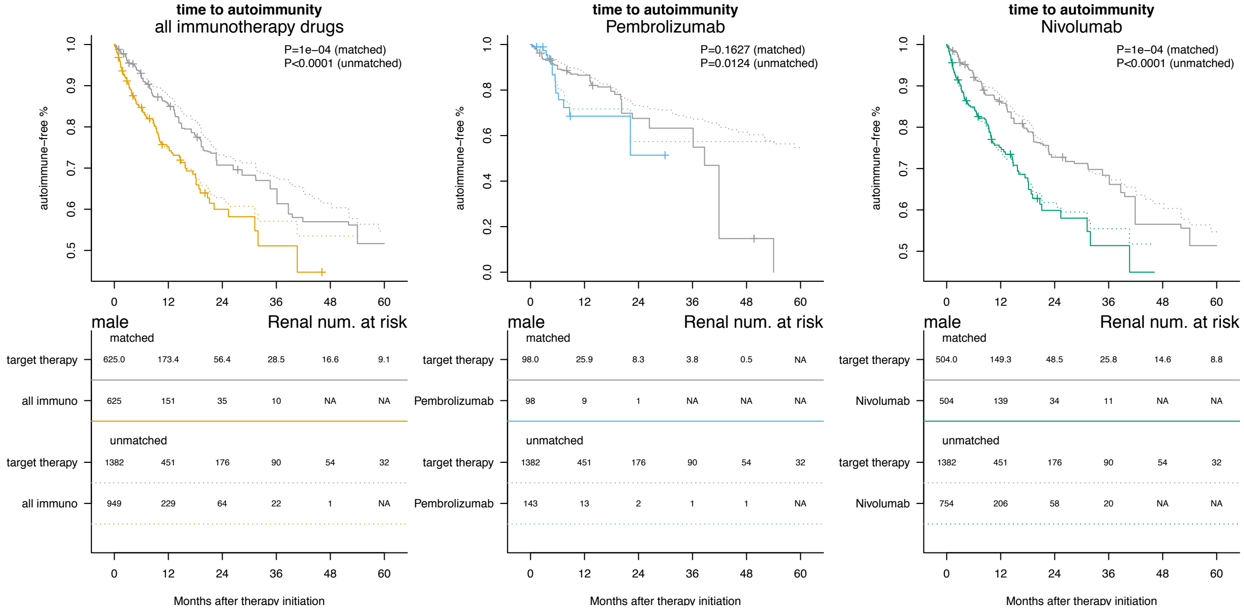

time to autoimmunity
all immunotherapy drugs
P=1e−04 (matched)
P<0.0001 (unmatched)
time to autoimmunity
Pembrolizumab
P=0.1627 (matched)
P=0.0124 (unmatched)
time to autoimmunity
Nivolumab
P=1e−04 (matched)
P<0.0001 (unmatched)
autoimmune−free %
male
Renal num. at risk
matched
target therapy 625.0 173.4 56.4 28.5 16.6 9.1
all immuno 625 151 35 10 NA NA
unmatched
target therapy 1382 451 176 90 54 32
all immuno 949 229 64 22 1 NA
matched
target therapy 98.0 25.9 8.3 3.8 0.5 NA
Pembrolizumab 98 9 1 NA NA NA
unmatched
target therapy 1382 451 176 90 54 32
Pembrolizumab 143 13 2 1 1 NA
matched
target therapy 504.0 149.3 48.5 25.8 14.6 8.8
Nivolumab 504 139 34 11 NA NA
unmatched
target therapy 1382 451 176 90 54 32
Nivolumab 754 206 58 20 NA NA
0 12 24 36 48 60
Months after therapy initiation

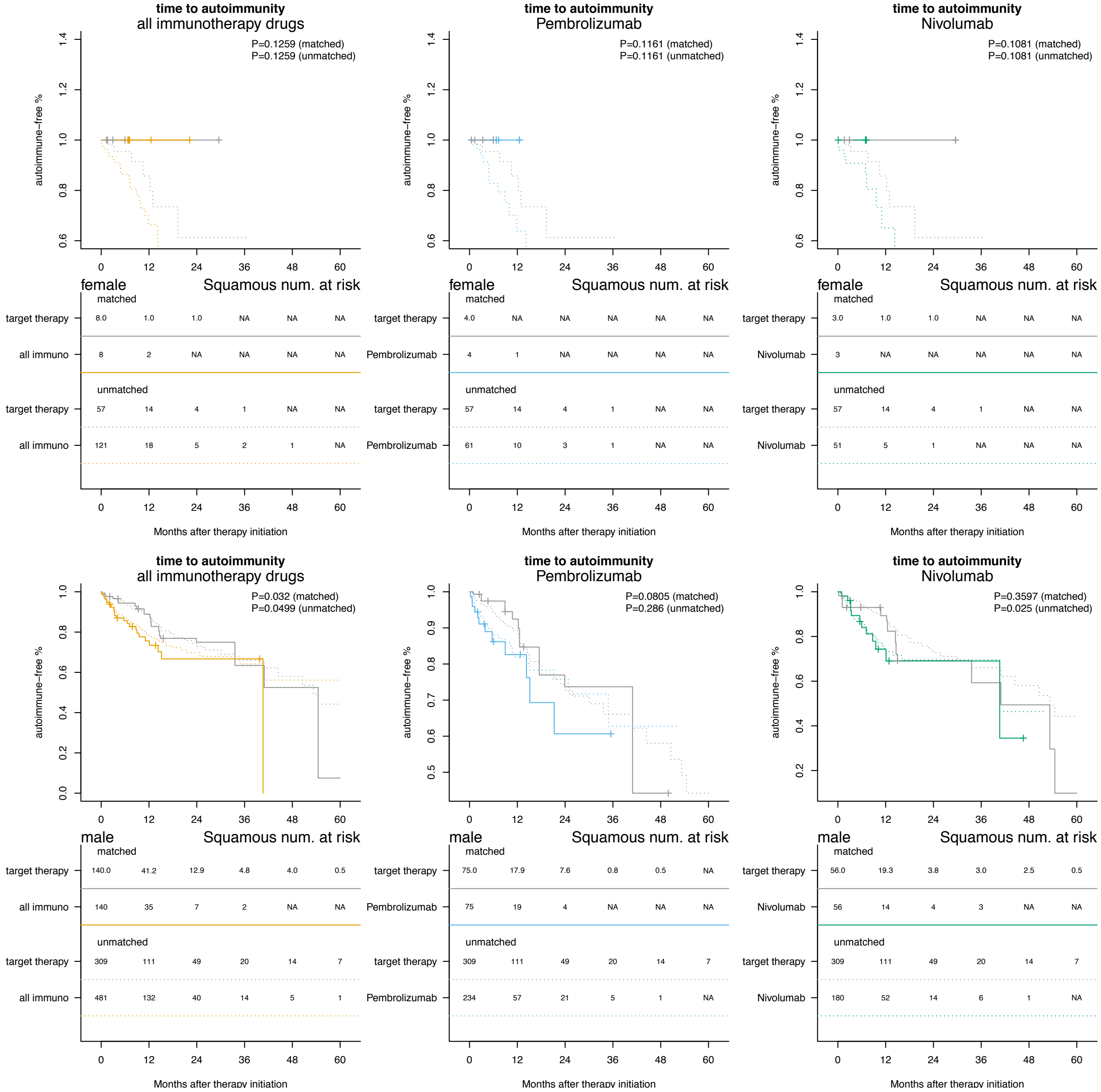
time to autoimmunity
all immunotherapy drugs
P=0.1259 (matched)
P=0.1259 (unmatched)
autoimmune−free %
female
Squamous num. at risk
matched
target therapy 8.0 1.0 1.0 NA NA NA
all immuno 8 2 NA NA NA NA
unmatched
target therapy 57 14 4 1 NA NA
all immuno 121 18 5 2 1 NA
Months after therapy initiation
time to autoimmunity
Pembrolizumab
P=0.1161 (matched)
P=0.1161 (unmatched)
female
Squamous num. at risk
matched
target therapy 4.0 NA NA NA NA NA
Pembrolizumab 4 1 NA NA NA NA
unmatched
target therapy 57 14 4 1 NA NA
Pembrolizumab 61 10 3 1 NA NA
Months after therapy initiation
time to autoimmunity
Nivolumab
P=0.1081 (matched)
P=0.1081 (unmatched)
female
Squamous num. at risk
matched
target therapy 3.0 1.0 1.0 NA NA NA
Nivolumab 3 NA NA NA NA NA
unmatched
target therapy 57 14 4 1 NA NA
Nivolumab 51 5 1 NA NA NA
Months after therapy initiation
time to autoimmunity
all immunotherapy drugs
P=0.032 (matched)
P=0.0499 (unmatched)
male
Squamous num. at risk
matched
target therapy 140.0 41.2 12.9 4.8 4.0 0.5
all immuno 140 35 7 2 NA NA
unmatched
target therapy 309 111 49 20 14 7
all immuno 481 132 40 14 5 1
Months after therapy initiation
time to autoimmunity
Pembrolizumab
P=0.0805 (matched)
P=0.286 (unmatched)
male
Squamous num. at risk
matched
target therapy 75.0 17.9 7.6 0.8 0.5 NA
Pembrolizumab 75 19 4 NA NA NA
unmatched
target therapy 309 111 49 20 14 7
Pembrolizumab 234 57 21 5 1 NA
Months after therapy initiation
time to autoimmunity
Nivolumab
P=0.3597 (matched)
P=0.025 (unmatched)
male
Squamous num. at risk
matched
target therapy 56.0 19.3 3.8 3.0 2.5 0.5
Nivolumab 56 14 4 3 NA NA
unmatched
target therapy 309 111 49 20 14 7
Nivolumab 180 52 14 6 1 NA
Months after therapy initiation

Age Stratification

We further stratified the results by looking at patients in different age groups separately. The trends of our main results are consistent across the age groups (Figure S12).

Figure S12. Results stratified by age groups.
Chemotherapy

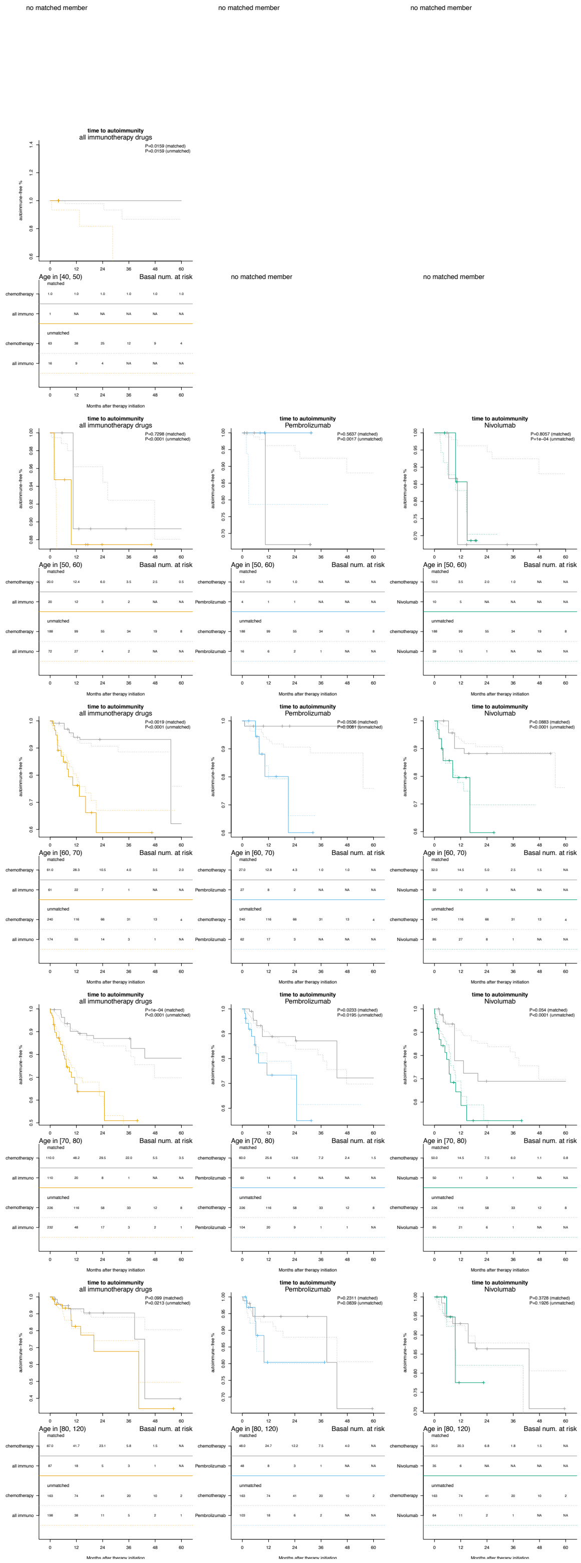

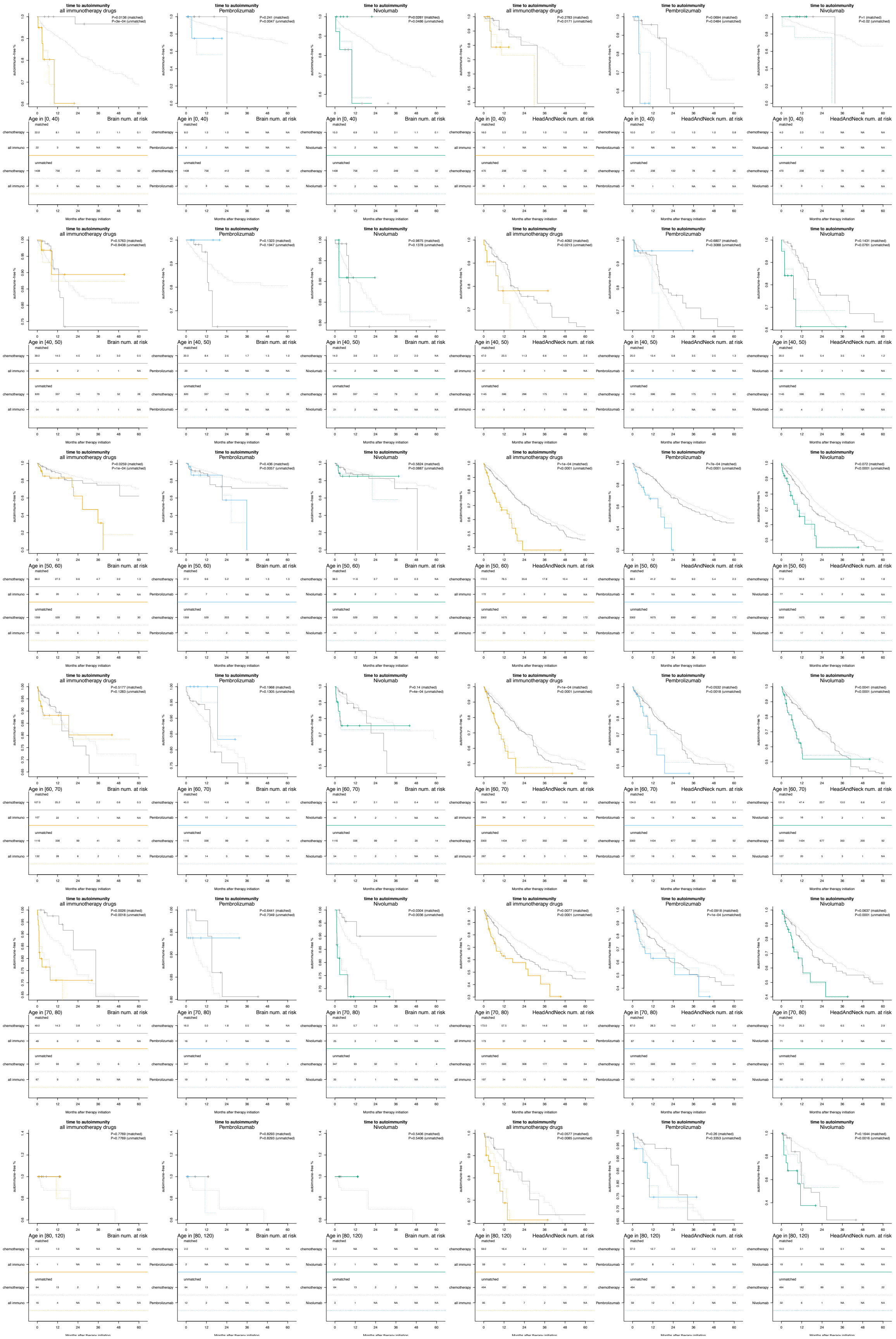

time to autoimmunity
all immunotherapy drugs
Pembrolizumab
Nivolumab
autoimmune-free %
Age in [0, 40)
Age in [40, 50)
Age in [50, 60)
Age in [60, 70)
Age in [70, 80)
Age in [80, 120)
Brain num. at risk
HeadAndNeck num. at risk
matched
unmatched
chemotherapy
all immuno
Months after therapy initiation

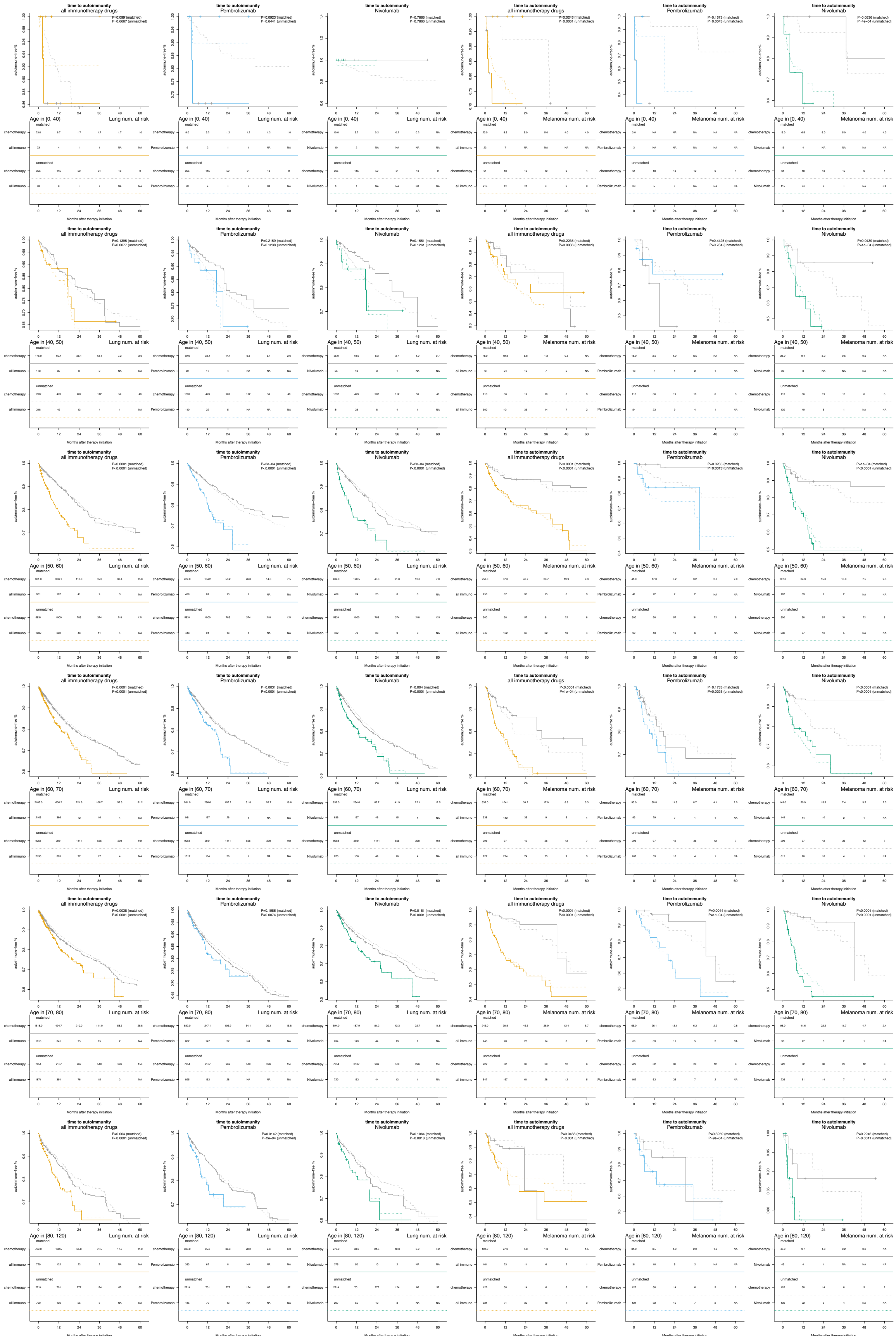

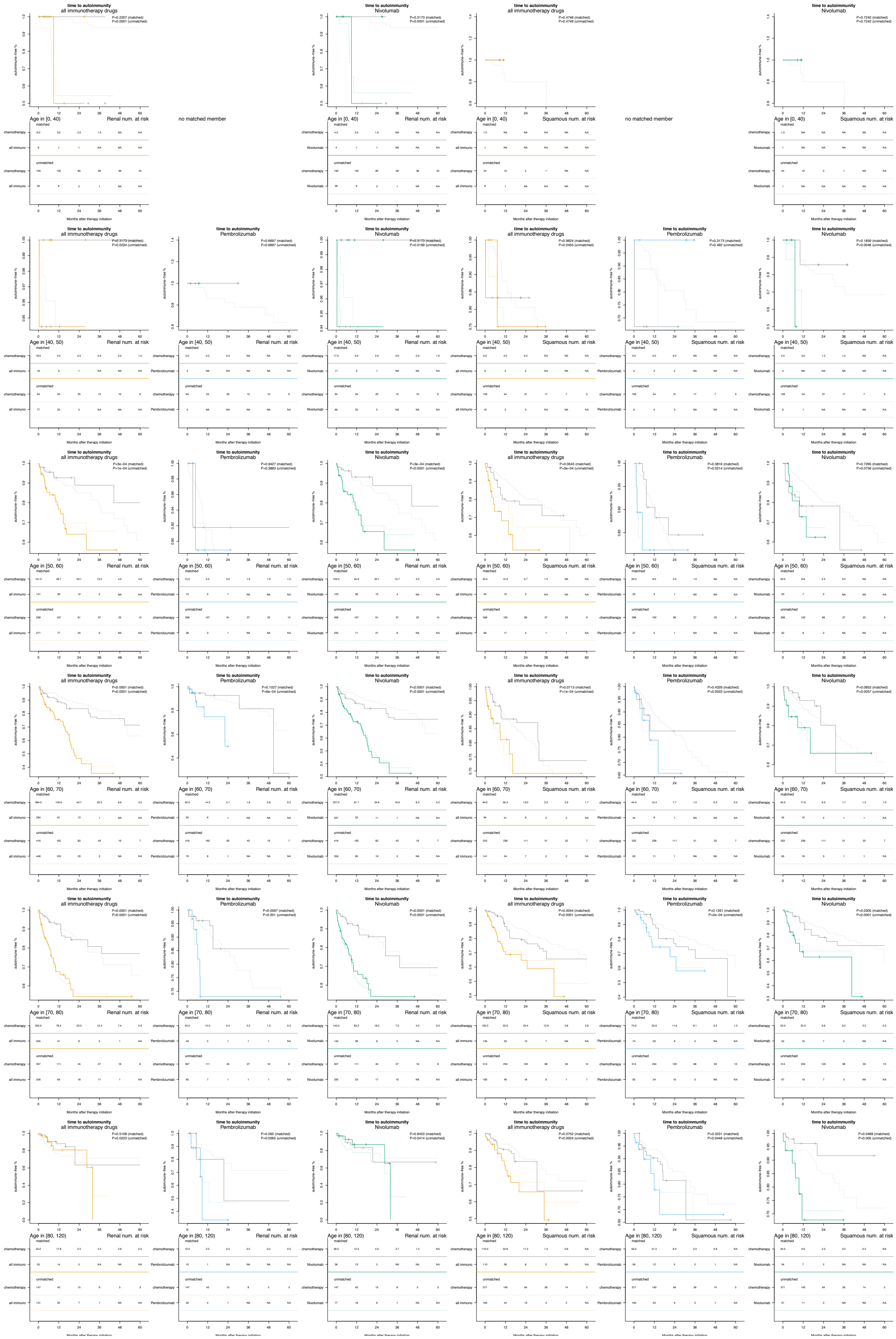

Targeted therapy

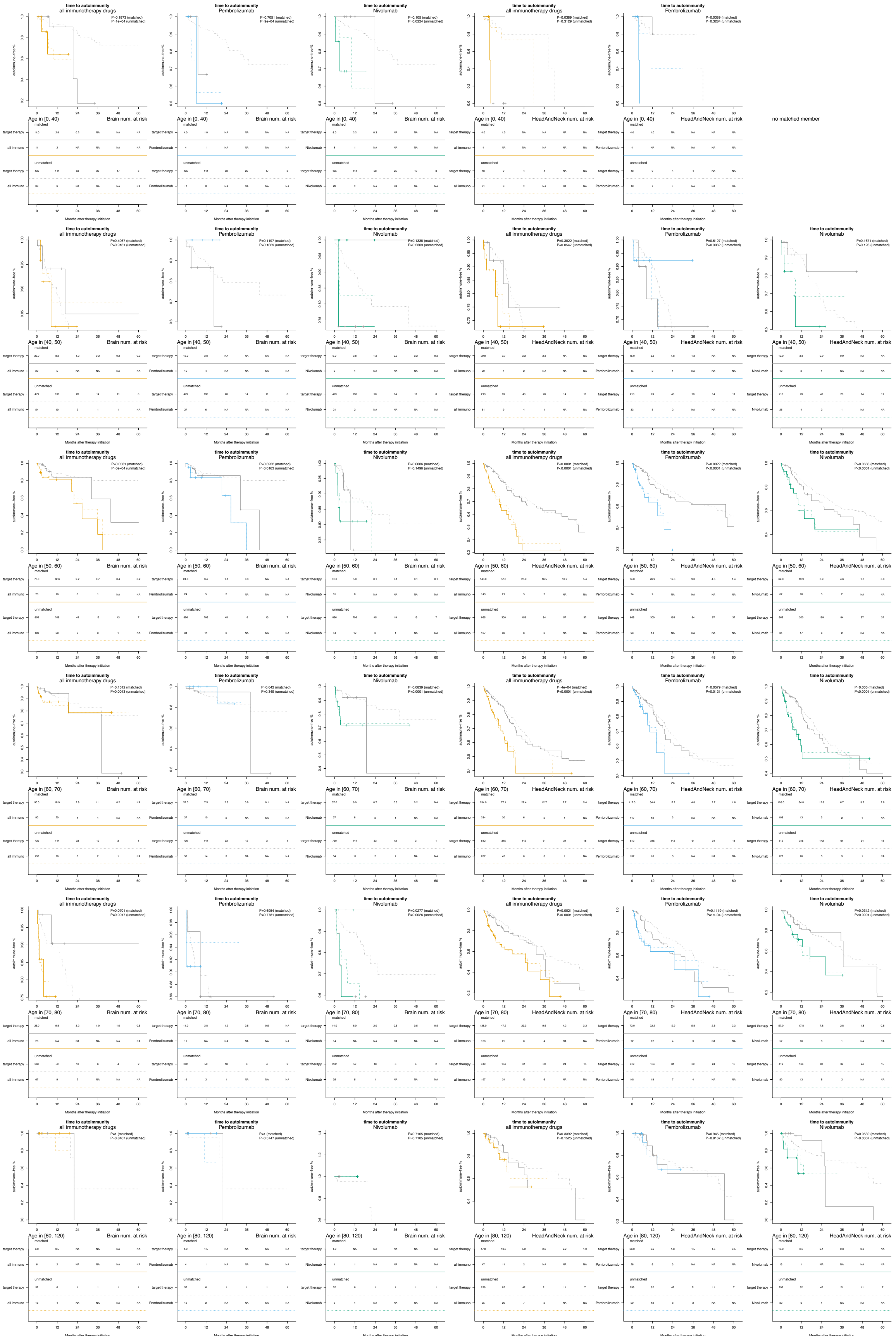

time to autoimmunity
all immunotherapy drugs
Pembrolizumab
Nivolumab
autoimmune-free %
Months after therapy initiation
target therapy
all immuno
matched
unmatched
Age in [0, 40)
Age in [40, 50)
Age in [50, 60)
Age in [60, 70)
Age in [70, 80)
Age in [80, 120)
Brain num. at risk
HeadAndNeck num. at risk
no matched member

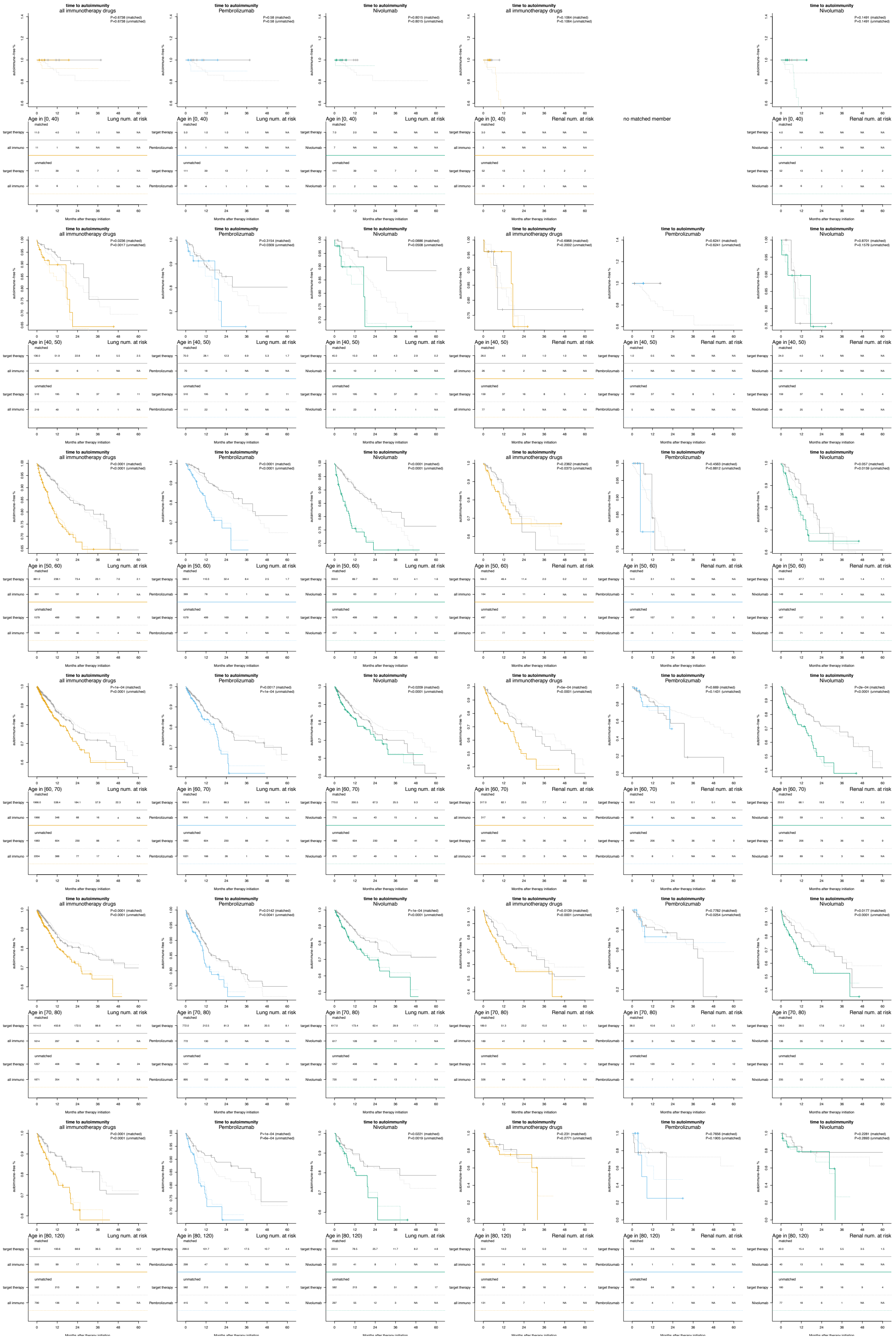

no matched member

no matched member

no matched member

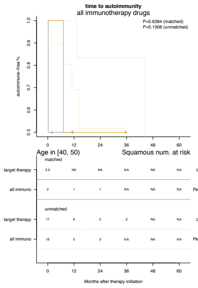

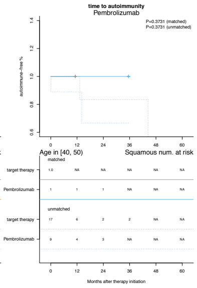

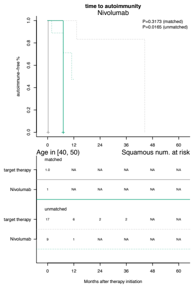

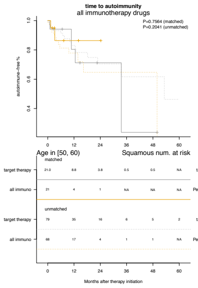

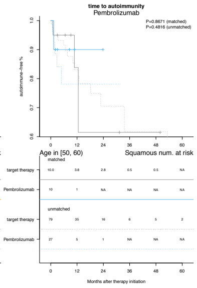

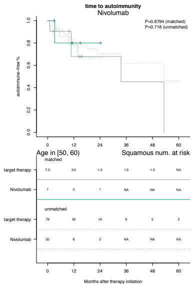

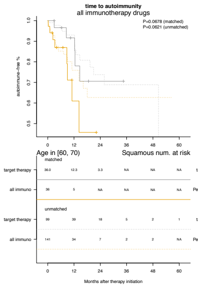

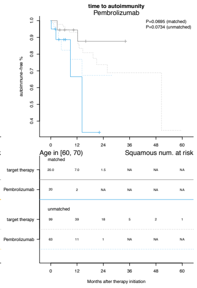

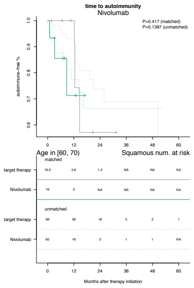

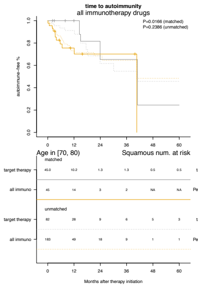

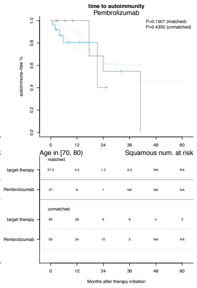

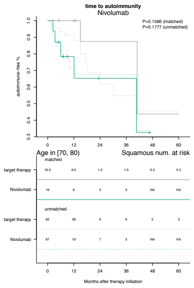

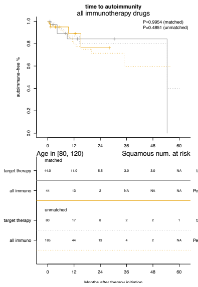

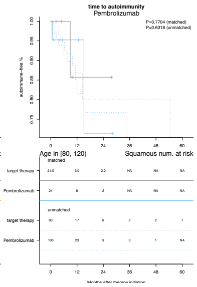

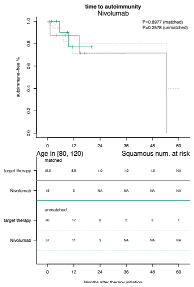

Income Stratification

Lastly, we conducted stratified analyses based on imputed income groups. The whole study population is separated into 5 quintiles using the zip-code-imputed income. The trends of our main results are consistent across the income groups (Figure S13).

Figure S13. Results stratified by income groups.
Chemotherapy

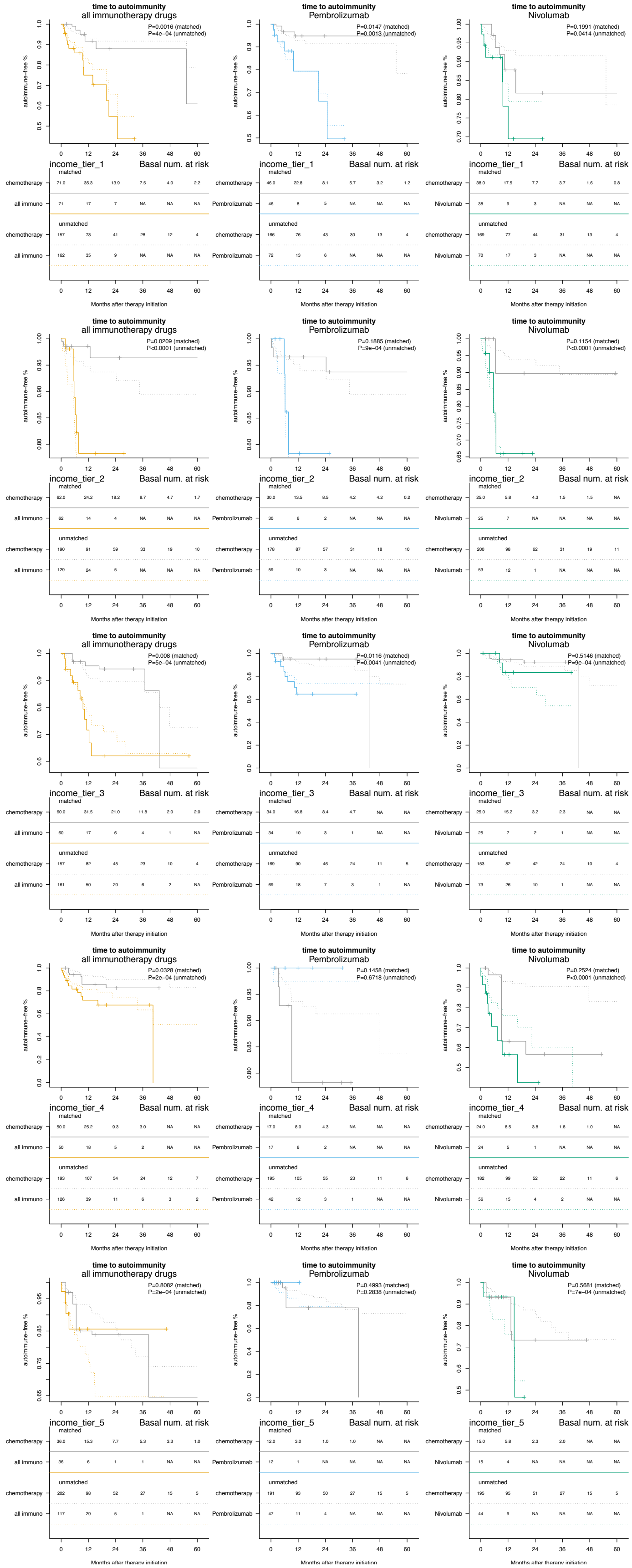

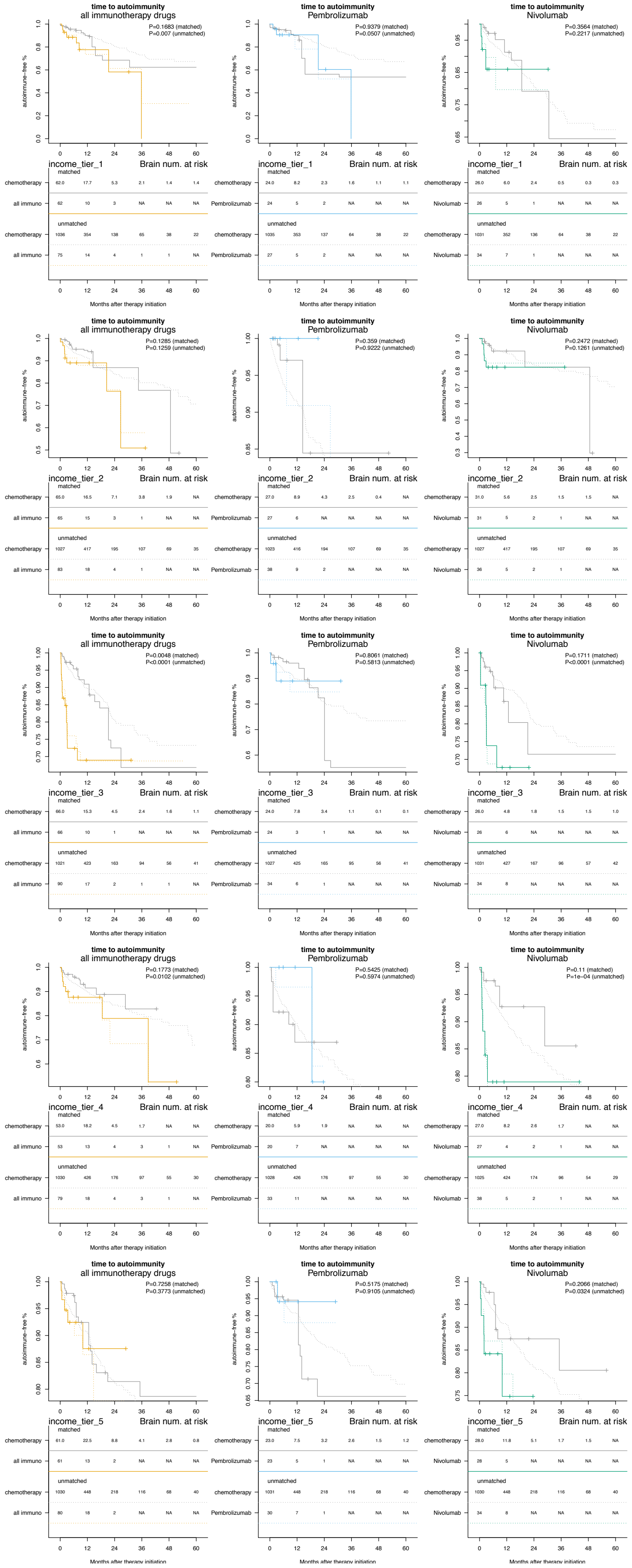

time to autoimmunity
all immunotherapy drugs
P=0.1683 (matched)
P=0.007 (unmatched)
time to autoimmunity
Pembrolizumab
P=0.9379 (matched)
P=0.0507 (unmatched)
time to autoimmunity
Nivolumab
P=0.3564 (matched)
P=0.2217 (unmatched)
income_tier_1
Brain num. at risk
time to autoimmunity
all immunotherapy drugs
P=0.1285 (matched)
P=0.1259 (unmatched)
time to autoimmunity
Pembrolizumab
P=0.359 (matched)
P=0.9222 (unmatched)
time to autoimmunity
Nivolumab
P=0.2472 (matched)
P=0.1261 (unmatched)
income_tier_2
time to autoimmunity
all immunotherapy drugs
P=0.0048 (matched)
P<0.0001 (unmatched)
time to autoimmunity
Pembrolizumab
P=0.8061 (matched)
P=0.5813 (unmatched)
time to autoimmunity
Nivolumab
P=0.1711 (matched)
P<0.0001 (unmatched)
income_tier_3
time to autoimmunity
all immunotherapy drugs
P=0.1773 (matched)
P=0.0102 (unmatched)
time to autoimmunity
Pembrolizumab
P=0.5425 (matched)
P=0.5974 (unmatched)
time to autoimmunity
Nivolumab
P=0.11 (matched)
P=1e-04 (unmatched)
income_tier_4
time to autoimmunity
all immunotherapy drugs
P=0.7258 (matched)
P=0.3773 (unmatched)
time to autoimmunity
Pembrolizumab
P=0.5175 (matched)
P=0.9105 (unmatched)
time to autoimmunity
Nivolumab
P=0.2066 (matched)
P=0.0324 (unmatched)
income_tier_5
autoimmune−free %
matched
unmatched
chemotherapy
all immuno
Pembrolizumab
Nivolumab
Months after therapy initiation

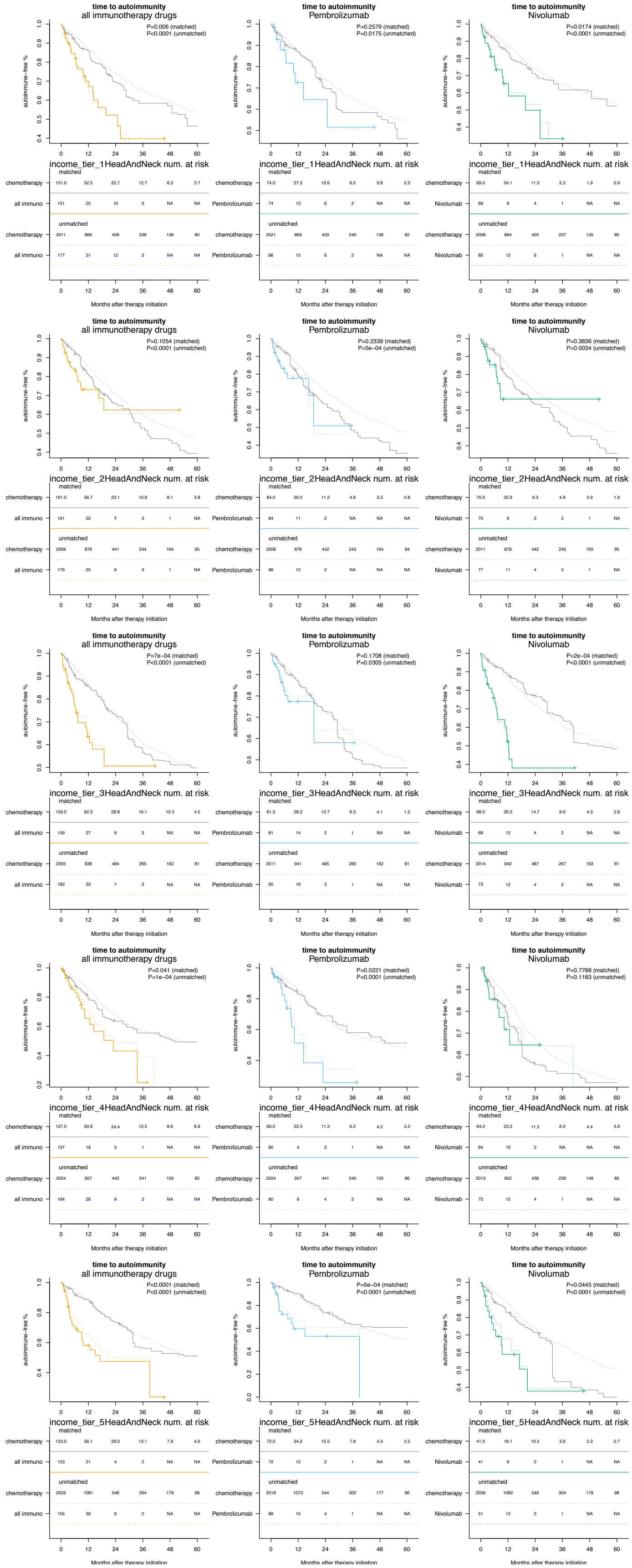

time to autoimmunity
all immunotherapy drugs
P=0.006 (matched)
P<0.0001 (unmatched)
time to autoimmunity
Pembrolizumab
P=0.2579 (matched)
P=0.0175 (unmatched)
time to autoimmunity
Nivolumab
P=0.0174 (matched)
P<0.0001 (unmatched)
income_tier_1HeadAndNeck num. at risk
time to autoimmunity
all immunotherapy drugs
P=0.1054 (matched)
P<0.0001 (unmatched)
time to autoimmunity
Pembrolizumab
P=0.2339 (matched)
P=5e−04 (unmatched)
time to autoimmunity
Nivolumab
P=0.3836 (matched)
P=0.0034 (unmatched)
income_tier_2HeadAndNeck num. at risk
time to autoimmunity
all immunotherapy drugs
P=7e−04 (matched)
P<0.0001 (unmatched)
time to autoimmunity
Pembrolizumab
P=0.1708 (matched)
P=0.0305 (unmatched)
time to autoimmunity
Nivolumab
P=2e−04 (matched)
P<0.0001 (unmatched)
income_tier_3HeadAndNeck num. at risk
time to autoimmunity
all immunotherapy drugs
P=0.041 (matched)
P=1e−04 (unmatched)
time to autoimmunity
Pembrolizumab
P=0.0221 (matched)
P<0.0001 (unmatched)
time to autoimmunity
Nivolumab
P=0.7788 (matched)
P=0.1183 (unmatched)
income_tier_4HeadAndNeck num. at risk
time to autoimmunity
all immunotherapy drugs
P<0.0001 (matched)
P<0.0001 (unmatched)
time to autoimmunity
Pembrolizumab
P=5e−04 (matched)
P<0.0001 (unmatched)
time to autoimmunity
Nivolumab
P=0.0445 (matched)
P<0.0001 (unmatched)
income_tier_5HeadAndNeck num. at risk
autoimmune−free %
matched
unmatched
chemotherapy
all immuno
Pembrolizumab
Nivolumab
Months after therapy initiation

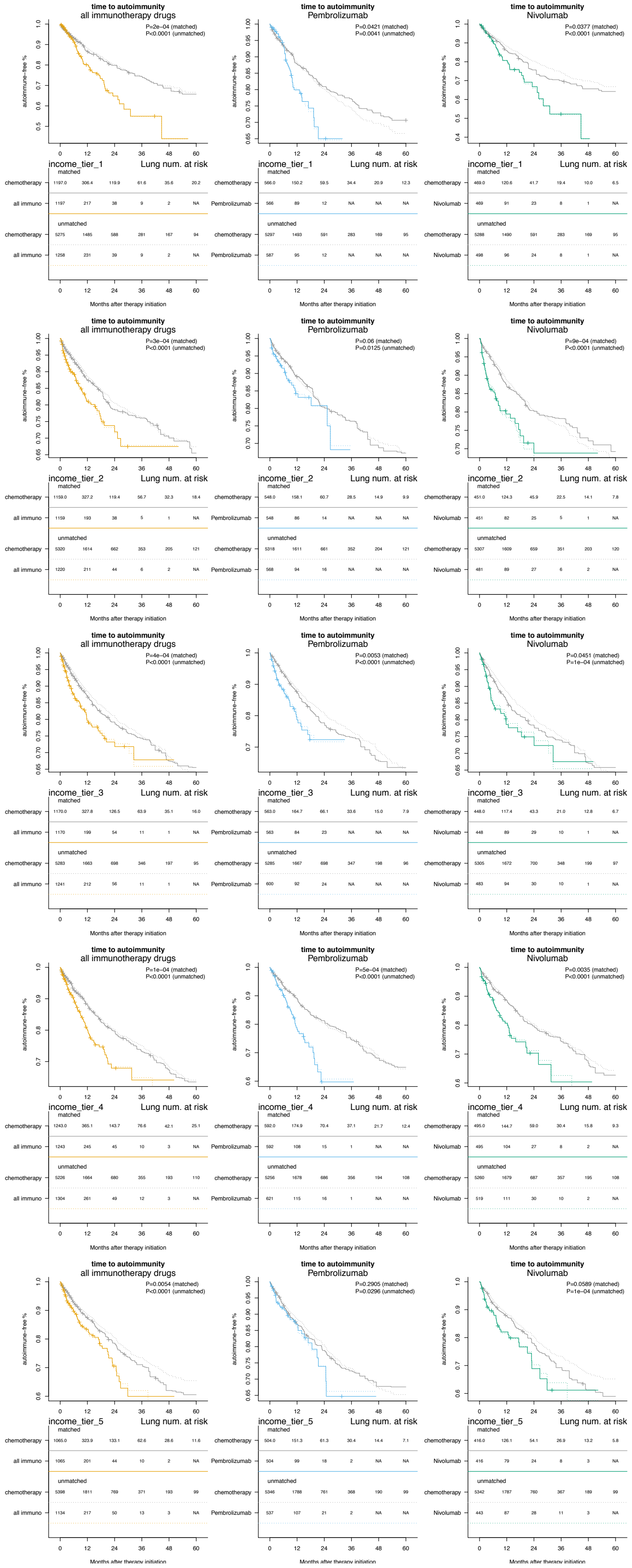
time to autoimmunity
all immunotherapy drugs
P=2e−04 (matched)
P<0.0001 (unmatched)
time to autoimmunity
Pembrolizumab
P=0.0421 (matched)
P=0.0041 (unmatched)
time to autoimmunity
Nivolumab
P=0.0377 (matched)
P<0.0001 (unmatched)
income_tier_1
Lung num. at risk
time to autoimmunity
all immunotherapy drugs
P=3e−04 (matched)
P<0.0001 (unmatched)
time to autoimmunity
Pembrolizumab
P=0.06 (matched)
P=0.0125 (unmatched)
time to autoimmunity
Nivolumab
P=9e−04 (matched)
P<0.0001 (unmatched)
income_tier_2
Lung num. at risk
time to autoimmunity
all immunotherapy drugs
P=4e−04 (matched)
P<0.0001 (unmatched)
time to autoimmunity
Pembrolizumab
P=0.0053 (matched)
P<0.0001 (unmatched)
time to autoimmunity
Nivolumab
P=0.0451 (matched)
P=1e−04 (unmatched)
income_tier_3
Lung num. at risk
time to autoimmunity
all immunotherapy drugs
P=1e−04 (matched)
P<0.0001 (unmatched)
time to autoimmunity
Pembrolizumab
P=5e−04 (matched)
P<0.0001 (unmatched)
time to autoimmunity
Nivolumab
P=0.0035 (matched)
P<0.0001 (unmatched)
income_tier_4
Lung num. at risk
time to autoimmunity
all immunotherapy drugs
P=0.0054 (matched)
P<0.0001 (unmatched)
time to autoimmunity
Pembrolizumab
P=0.2905 (matched)
P=0.0296 (unmatched)
time to autoimmunity
Nivolumab
P=0.0589 (matched)
P=1e−04 (unmatched)
income_tier_5
Lung num. at risk
Months after therapy initiation

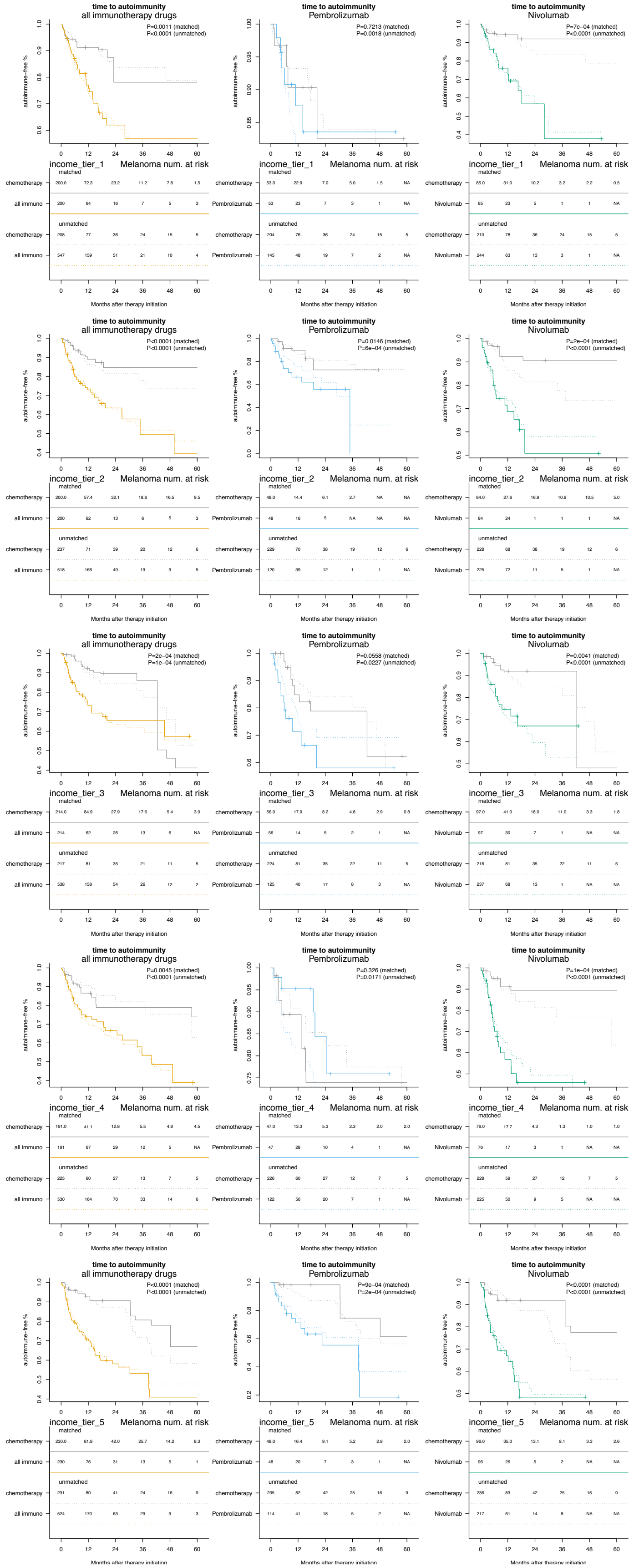
time to autoimmunity
all immunotherapy drugs
P=0.0011 (matched)
P<0.0001 (unmatched)
time to autoimmunity
Pembrolizumab
P=0.7213 (matched)
P=0.0018 (unmatched)
time to autoimmunity
Nivolumab
P=7e−04 (matched)
P<0.0001 (unmatched)
income_tier_1 Melanoma num. at risk
time to autoimmunity
all immunotherapy drugs
P<0.0001 (matched)
P<0.0001 (unmatched)
time to autoimmunity
Pembrolizumab
P=0.0146 (matched)
P=6e−04 (unmatched)
time to autoimmunity
Nivolumab
P=2e−04 (matched)
P<0.0001 (unmatched)
income_tier_2 Melanoma num. at risk
time to autoimmunity
all immunotherapy drugs
P=2e−04 (matched)
P=1e−04 (unmatched)
time to autoimmunity
Pembrolizumab
P=0.0558 (matched)
P=0.0227 (unmatched)
time to autoimmunity
Nivolumab
P=0.0041 (matched)
P<0.0001 (unmatched)
income_tier_3 Melanoma num. at risk
time to autoimmunity
all immunotherapy drugs
P=0.0045 (matched)
P<0.0001 (unmatched)
time to autoimmunity
Pembrolizumab
P=0.326 (matched)
P=0.0171 (unmatched)
time to autoimmunity
Nivolumab
P=1e−04 (matched)
P<0.0001 (unmatched)
income_tier_4 Melanoma num. at risk
time to autoimmunity
all immunotherapy drugs
P<0.0001 (matched)
P<0.0001 (unmatched)
time to autoimmunity
Pembrolizumab
P=9e−04 (matched)
P=2e−04 (unmatched)
time to autoimmunity
Nivolumab
P<0.0001 (matched)
P<0.0001 (unmatched)
income_tier_5 Melanoma num. at risk
autoimmune−free %
matched
unmatched
chemotherapy
all immuno
Pembrolizumab
Nivolumab
Months after therapy initiation

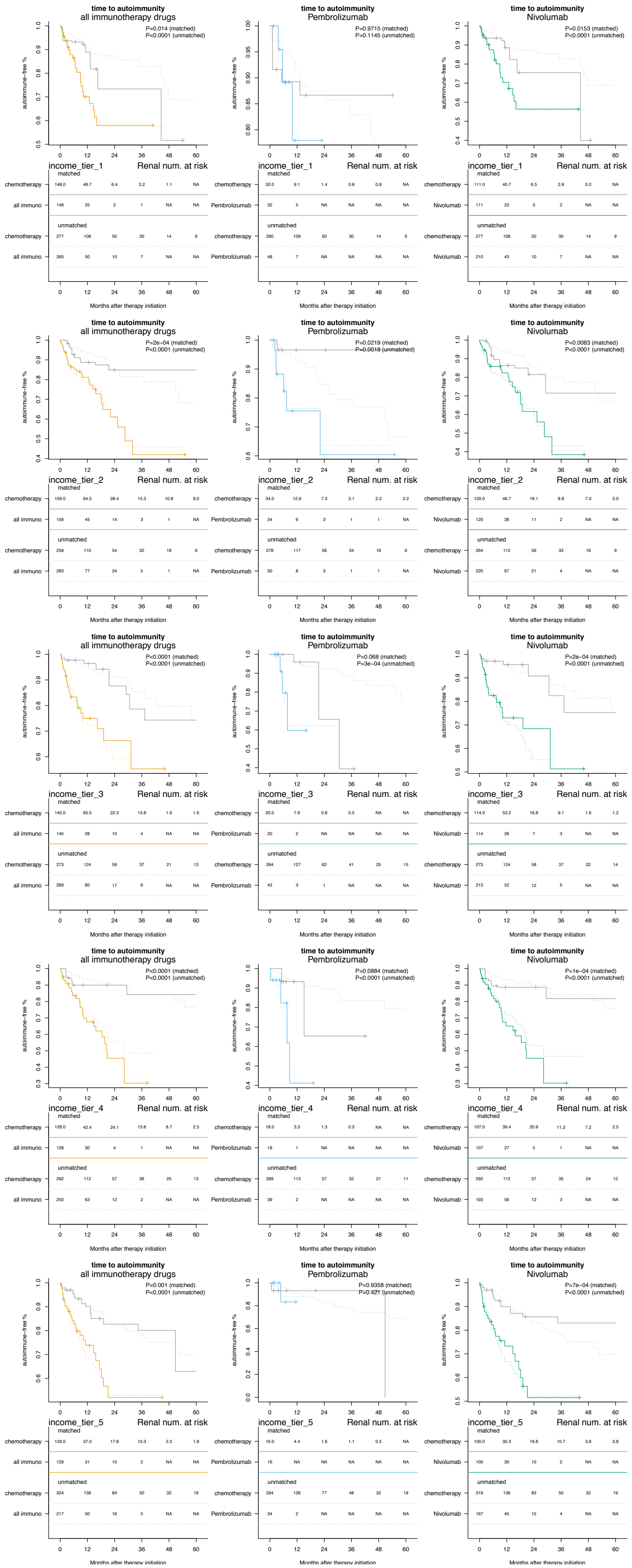

time to autoimmunity
all immunotherapy drugs
P=0.014 (matched)
P<0.0001 (unmatched)
income_tier_1
Renal num. at risk
time to autoimmunity
Pembrolizumab
P=0.9715 (matched)
P=0.1145 (unmatched)
income_tier_1
Renal num. at risk
time to autoimmunity
Nivolumab
P=0.0153 (matched)
P<0.0001 (unmatched)
income_tier_1
Renal num. at risk
time to autoimmunity
all immunotherapy drugs
P=2e−04 (matched)
P<0.0001 (unmatched)
income_tier_2
Renal num. at risk
time to autoimmunity
Pembrolizumab
P=0.0219 (matched)
P=0.0018 (unmatched)
income_tier_2
Renal num. at risk
time to autoimmunity
Nivolumab
P=0.0083 (matched)
P<0.0001 (unmatched)
income_tier_2
Renal num. at risk
time to autoimmunity
all immunotherapy drugs
P<0.0001 (matched)
P<0.0001 (unmatched)
income_tier_3
Renal num. at risk
time to autoimmunity
Pembrolizumab
P=0.068 (matched)
P=3e−04 (unmatched)
income_tier_3
Renal num. at risk
time to autoimmunity
Nivolumab
P=2e−04 (matched)
P<0.0001 (unmatched)
income_tier_3
Renal num. at risk
time to autoimmunity
all immunotherapy drugs
P<0.0001 (matched)
P<0.0001 (unmatched)
income_tier_4
Renal num. at risk
time to autoimmunity
Pembrolizumab
P=0.0884 (matched)
P<0.0001 (unmatched)
income_tier_4
Renal num. at risk
time to autoimmunity
Nivolumab
P=1e−04 (matched)
P<0.0001 (unmatched)
income_tier_4
Renal num. at risk
time to autoimmunity
all immunotherapy drugs
P=0.001 (matched)
P<0.0001 (unmatched)
income_tier_5
Renal num. at risk
time to autoimmunity
Pembrolizumab
P=0.9358 (matched)
P=0.421 (unmatched)
income_tier_5
Renal num. at risk
time to autoimmunity
Nivolumab
P=7e−04 (matched)
P<0.0001 (unmatched)
income_tier_5
Renal num. at risk
autoimmune−free %
Months after therapy initiation
matched
unmatched
chemotherapy
all immuno
Pembrolizumab
Nivolumab

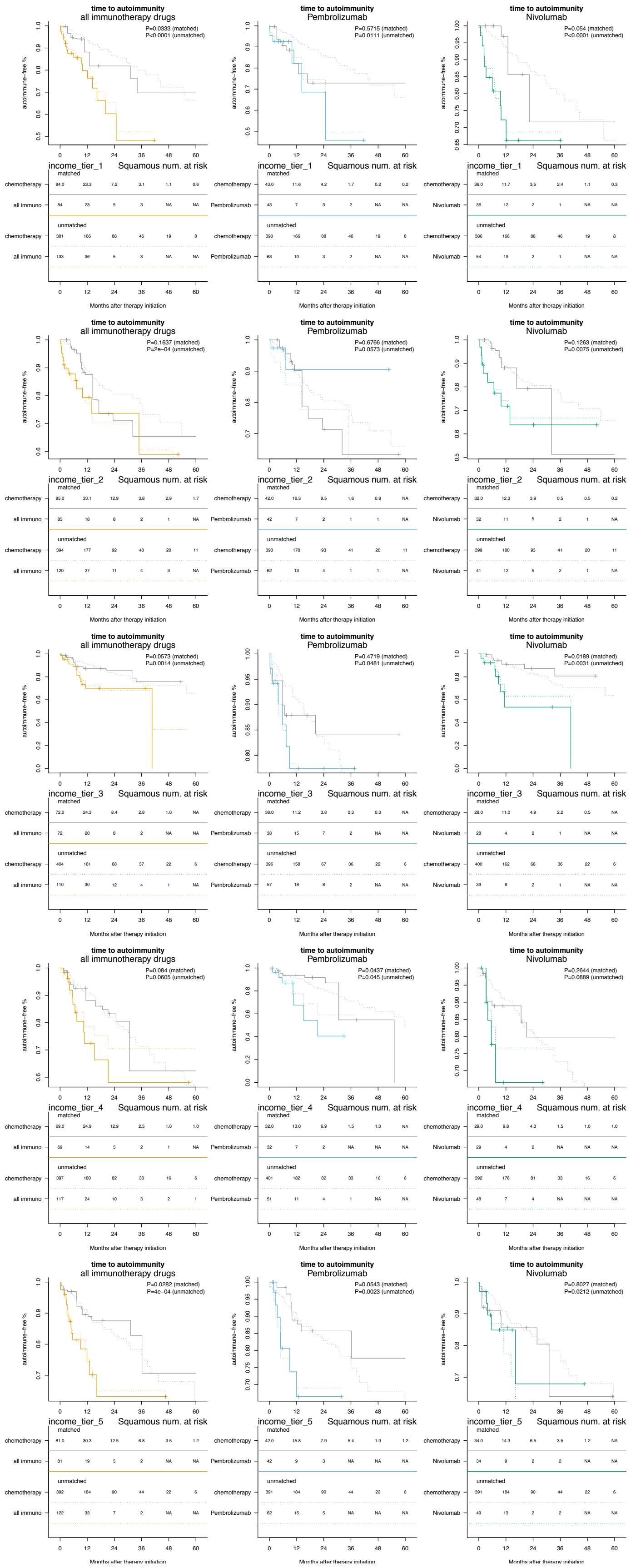

time to autoimmunity
all immunotherapy drugs
Pembrolizumab
Nivolumab
autoimmune-free %
Months after therapy initiation
income_tier_1 Squamous num. at risk
P=0.0333 (matched)
P<0.0001 (unmatched)
P=0.5715 (matched)
P=0.0111 (unmatched)
P=0.054 (matched)
P<0.0001 (unmatched)
income_tier_2 Squamous num. at risk
P=0.1637 (matched)
P=2e−04 (unmatched)
P=0.6766 (matched)
P=0.0573 (unmatched)
P=0.1263 (matched)
P=0.0075 (unmatched)
income_tier_3 Squamous num. at risk
P=0.0573 (matched)
P=0.0014 (unmatched)
P=0.4719 (matched)
P=0.0481 (unmatched)
P=0.0189 (matched)
P=0.0031 (unmatched)
income_tier_4 Squamous num. at risk
P=0.084 (matched)
P=0.0605 (unmatched)
P=0.0437 (matched)
P=0.045 (unmatched)
P=0.2644 (matched)
P=0.0889 (unmatched)
income_tier_5 Squamous num. at risk
P=0.0282 (matched)
P=4e−04 (unmatched)
P=0.0543 (matched)
P=0.0023 (unmatched)
P=0.8027 (matched)
P=0.0212 (unmatched)
matched
unmatched
chemotherapy
all immuno
Pembrolizumab
Nivolumab

Targeted therapy

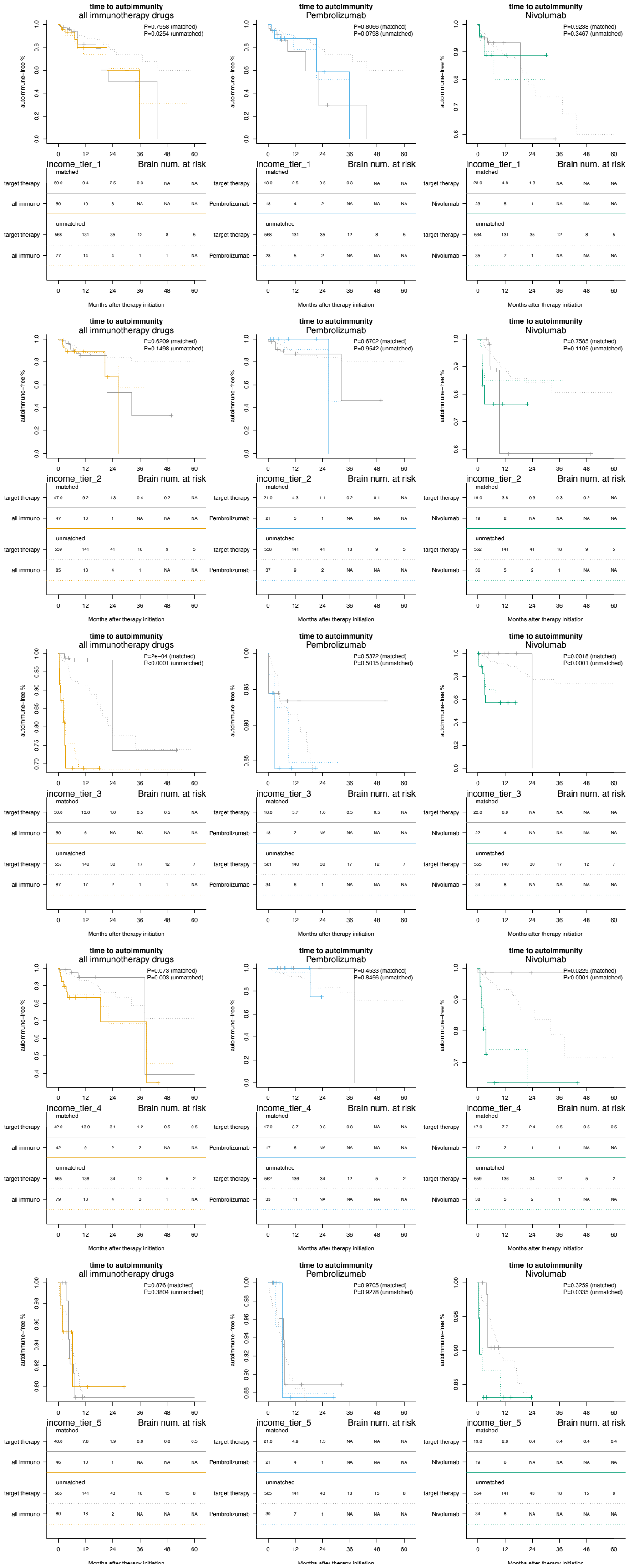

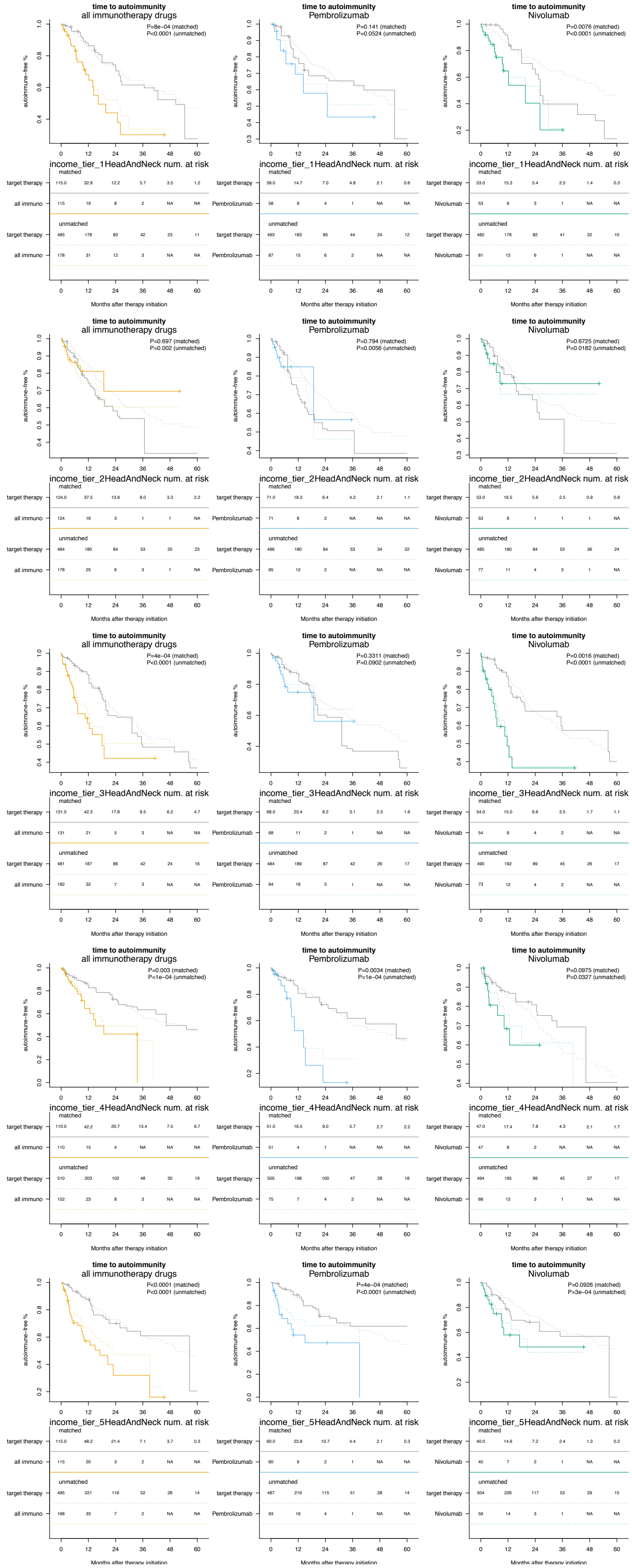

time to autoimmunity
all immunotherapy drugs
P=8e−04 (matched)
P<0.0001 (unmatched)
income_tier_1HeadAndNeck num. at risk
time to autoimmunity
Pembrolizumab
P=0.141 (matched)
P=0.0524 (unmatched)
income_tier_1HeadAndNeck num. at risk
time to autoimmunity
Nivolumab
P=0.0076 (matched)
P<0.0001 (unmatched)
income_tier_1HeadAndNeck num. at risk
time to autoimmunity
all immunotherapy drugs
P=0.697 (matched)
P=0.002 (unmatched)
income_tier_2HeadAndNeck num. at risk
time to autoimmunity
Pembrolizumab
P=0.794 (matched)
P=0.0056 (unmatched)
income_tier_2HeadAndNeck num. at risk
time to autoimmunity
Nivolumab
P=0.6725 (matched)
P=0.0182 (unmatched)
income_tier_2HeadAndNeck num. at risk
time to autoimmunity
all immunotherapy drugs
P=4e−04 (matched)
P<0.0001 (unmatched)
income_tier_3HeadAndNeck num. at risk
time to autoimmunity
Pembrolizumab
P=0.3311 (matched)
P=0.0902 (unmatched)
income_tier_3HeadAndNeck num. at risk
time to autoimmunity
Nivolumab
P=0.0016 (matched)
P<0.0001 (unmatched)
income_tier_3HeadAndNeck num. at risk
time to autoimmunity
all immunotherapy drugs
P=0.003 (matched)
P=1e−04 (unmatched)
income_tier_4HeadAndNeck num. at risk
time to autoimmunity
Pembrolizumab
P=0.0034 (matched)
P=1e−04 (unmatched)
income_tier_4HeadAndNeck num. at risk
time to autoimmunity
Nivolumab
P=0.0975 (matched)
P=0.0327 (unmatched)
income_tier_4HeadAndNeck num. at risk
time to autoimmunity
all immunotherapy drugs
P<0.0001 (matched)
P<0.0001 (unmatched)
income_tier_5HeadAndNeck num. at risk
time to autoimmunity
Pembrolizumab
P=4e−04 (matched)
P<0.0001 (unmatched)
income_tier_5HeadAndNeck num. at risk
time to autoimmunity
Nivolumab
P=0.0926 (matched)
P=3e−04 (unmatched)
income_tier_5HeadAndNeck num. at risk
autoimmune−free %
matched
unmatched
target therapy
all immuno
Pembrolizumab
Nivolumab
Months after therapy initiation

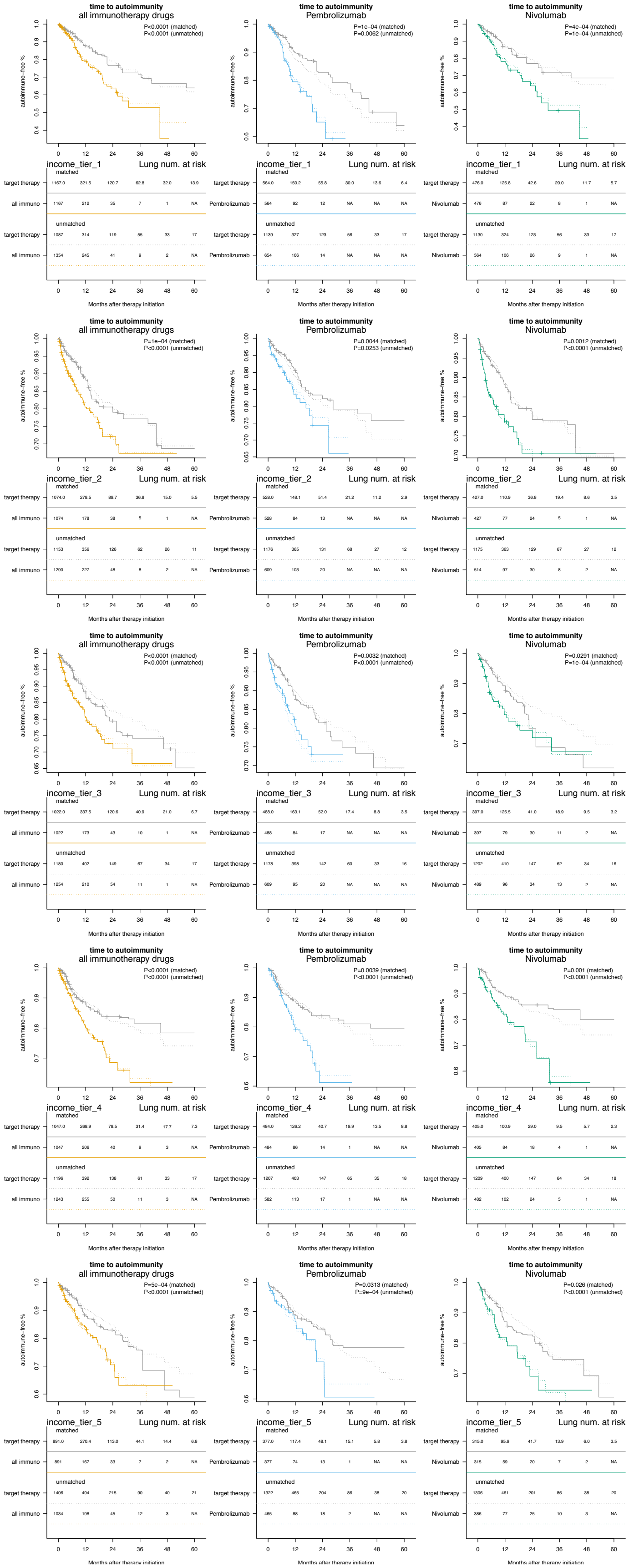
time to autoimmunity
all immunotherapy drugs
P<0.0001 (matched)
P<0.0001 (unmatched)
autoimmune−free %
income_tier_1
Lung num. at risk
matched
target therapy 1167.0 321.5 120.7 62.8 32.0 13.9
all immuno 1167 212 35 7 1 NA
unmatched
target therapy 1087 314 119 55 33 17
all immuno 1354 245 41 9 2 NA
Months after therapy initiation
time to autoimmunity
Pembrolizumab
P=1e−04 (matched)
P=0.0062 (unmatched)
income_tier_1
Lung num. at risk
matched
target therapy 564.0 150.2 55.8 30.0 13.6 6.4
Pembrolizumab 564 92 12 NA NA NA
unmatched
target therapy 1139 327 123 56 33 17
Pembrolizumab 654 106 14 NA NA NA
Months after therapy initiation
time to autoimmunity
Nivolumab
P=4e−04 (matched)
P=1e−04 (unmatched)
income_tier_1
Lung num. at risk
matched
target therapy 476.0 125.8 42.6 20.0 11.7 5.7
Nivolumab 476 87 22 8 1 NA
unmatched
target therapy 1130 324 123 56 33 17
Nivolumab 564 106 26 9 1 NA
Months after therapy initiation
time to autoimmunity
all immunotherapy drugs
P=1e−04 (matched)
P<0.0001 (unmatched)
income_tier_2
Lung num. at risk
matched
target therapy 1074.0 278.5 89.7 36.8 15.0 5.5
all immuno 1074 178 38 5 1 NA
unmatched
target therapy 1153 356 126 62 26 11
all immuno 1290 227 48 8 2 NA
Months after therapy initiation
time to autoimmunity
Pembrolizumab
P=0.0044 (matched)
P=0.0253 (unmatched)
income_tier_2
Lung num. at risk
matched
target therapy 528.0 148.1 51.4 21.2 11.2 2.9
Pembrolizumab 528 84 13 NA NA NA
unmatched
target therapy 1176 365 131 68 27 12
Pembrolizumab 609 103 20 NA NA NA
Months after therapy initiation
time to autoimmunity
Nivolumab
P=0.0012 (matched)
P<0.0001 (unmatched)
income_tier_2
Lung num. at risk
matched
target therapy 427.0 110.9 36.8 19.4 8.6 3.5
Nivolumab 427 77 24 5 1 NA
unmatched
target therapy 1175 363 129 67 27 12
Nivolumab 514 97 30 8 2 NA
Months after therapy initiation
time to autoimmunity
all immunotherapy drugs
P<0.0001 (matched)
P<0.0001 (unmatched)
income_tier_3
Lung num. at risk
matched
target therapy 1022.0 337.5 120.6 40.9 21.0 6.7
all immuno 1022 173 43 10 1 NA
unmatched
target therapy 1180 402 149 67 34 17
all immuno 1254 210 54 11 1 NA
Months after therapy initiation
time to autoimmunity
Pembrolizumab
P=0.0032 (matched)
P<0.0001 (unmatched)
income_tier_3
Lung num. at risk
matched
target therapy 488.0 163.1 52.0 17.4 8.8 3.5
Pembrolizumab 488 84 17 NA NA NA
unmatched
target therapy 1178 398 142 60 33 16
Pembrolizumab 609 95 20 NA NA NA
Months after therapy initiation
time to autoimmunity
Nivolumab
P=0.0291 (matched)
P=1e−04 (unmatched)
income_tier_3
Lung num. at risk
matched
target therapy 397.0 125.5 41.0 18.9 9.5 3.2
Nivolumab 397 79 30 11 2 NA
unmatched
target therapy 1202 410 147 62 34 16
Nivolumab 489 96 34 13 2 NA
Months after therapy initiation
time to autoimmunity
all immunotherapy drugs
P<0.0001 (matched)
P<0.0001 (unmatched)
income_tier_4
Lung num. at risk
matched
target therapy 1047.0 268.9 78.5 31.4 17.7 7.3
all immuno 1047 206 40 9 3 NA
unmatched
target therapy 1196 392 138 61 33 17
all immuno 1243 255 50 11 3 NA
Months after therapy initiation
time to autoimmunity
Pembrolizumab
P=0.0039 (matched)
P<0.0001 (unmatched)
income_tier_4
Lung num. at risk
matched
target therapy 484.0 126.2 40.7 19.9 13.5 8.8
Pembrolizumab 484 86 14 1 NA NA
unmatched
target therapy 1207 403 147 65 35 18
Pembrolizumab 582 113 17 1 NA NA
Months after therapy initiation
time to autoimmunity
Nivolumab
P=0.001 (matched)
P<0.0001 (unmatched)
income_tier_4
Lung num. at risk
matched
target therapy 405.0 100.9 29.0 9.5 5.7 2.3
Nivolumab 405 84 18 4 1 NA
unmatched
target therapy 1209 400 147 64 34 18
Nivolumab 482 102 24 5 1 NA
Months after therapy initiation
time to autoimmunity
all immunotherapy drugs
P=5e−04 (matched)
P<0.0001 (unmatched)
income_tier_5
Lung num. at risk
matched
target therapy 891.0 270.4 113.0 44.1 14.4 6.8
all immuno 891 167 33 7 2 NA
unmatched
target therapy 1406 494 215 90 40 21
all immuno 1034 198 45 12 3 NA
Months after therapy initiation
time to autoimmunity
Pembrolizumab
P=0.0313 (matched)
P=9e−04 (unmatched)
income_tier_5
Lung num. at risk
matched
target therapy 377.0 117.4 48.1 15.1 5.8 3.8
Pembrolizumab 377 74 13 1 NA NA
unmatched
target therapy 1322 465 204 86 38 20
Pembrolizumab 465 88 18 2 NA NA
Months after therapy initiation
time to autoimmunity
Nivolumab
P=0.026 (matched)
P<0.0001 (unmatched)
income_tier_5
Lung num. at risk
matched
target therapy 315.0 95.9 41.7 13.9 6.0 3.5
Nivolumab 315 59 20 7 2 NA
unmatched
target therapy 1306 461 201 86 38 20
Nivolumab 386 77 25 10 3 NA
Months after therapy initiation

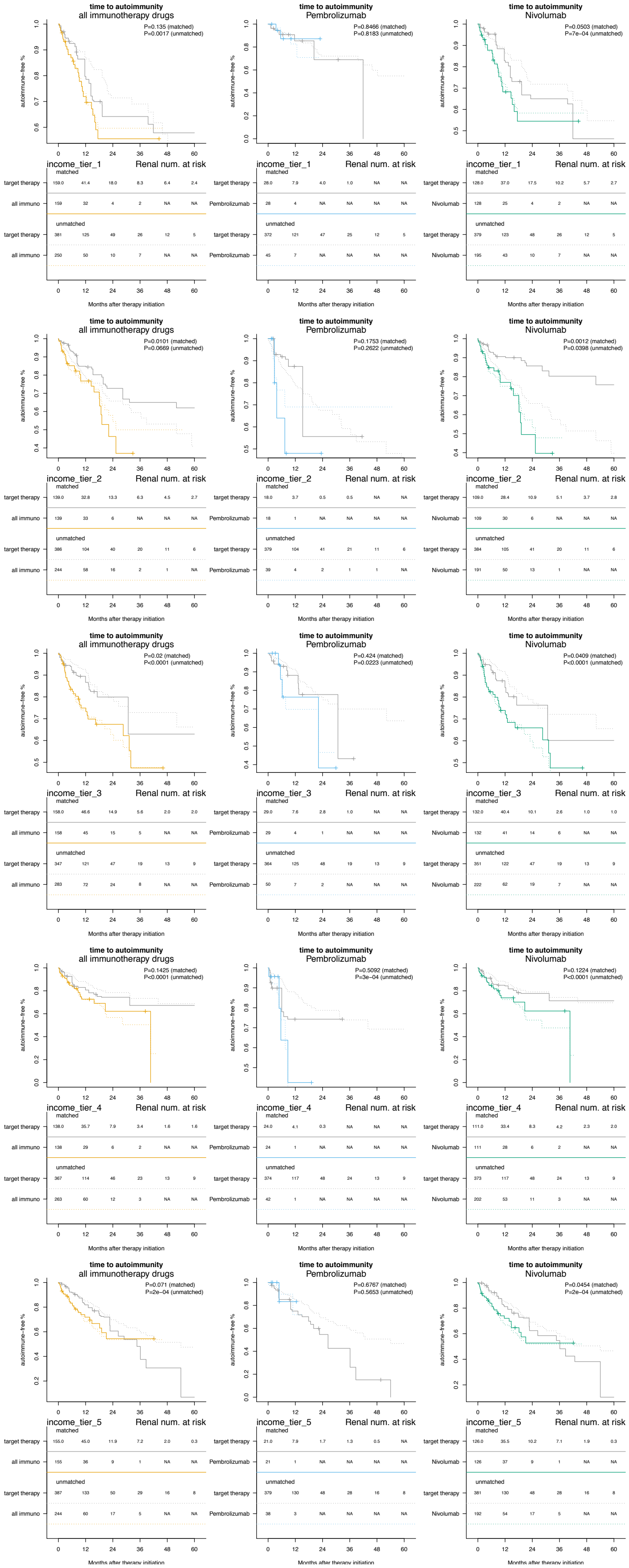
time to autoimmunity
all immunotherapy drugs
P=0.135 (matched)
P=0.0017 (unmatched)
income_tier_1
Renal num. at risk
time to autoimmunity
Pembrolizumab
P=0.8466 (matched)
P=0.8183 (unmatched)
income_tier_1
Renal num. at risk
time to autoimmunity
Nivolumab
P=0.0503 (matched)
P=7e−04 (unmatched)
income_tier_1
Renal num. at risk
time to autoimmunity
all immunotherapy drugs
P=0.0101 (matched)
P=0.0669 (unmatched)
income_tier_2
Renal num. at risk
time to autoimmunity
Pembrolizumab
P=0.1753 (matched)
P=0.2622 (unmatched)
income_tier_2
Renal num. at risk
time to autoimmunity
Nivolumab
P=0.0012 (matched)
P=0.0398 (unmatched)
income_tier_2
Renal num. at risk
time to autoimmunity
all immunotherapy drugs
P=0.02 (matched)
P<0.0001 (unmatched)
income_tier_3
Renal num. at risk
time to autoimmunity
Pembrolizumab
P=0.424 (matched)
P=0.0223 (unmatched)
income_tier_3
Renal num. at risk
time to autoimmunity
Nivolumab
P=0.0409 (matched)
P<0.0001 (unmatched)
income_tier_3
Renal num. at risk
time to autoimmunity
all immunotherapy drugs
P=0.1425 (matched)
P<0.0001 (unmatched)
income_tier_4
Renal num. at risk
time to autoimmunity
Pembrolizumab
P=0.5092 (matched)
P=3e−04 (unmatched)
income_tier_4
Renal num. at risk
time to autoimmunity
Nivolumab
P=0.1224 (matched)
P<0.0001 (unmatched)
income_tier_4
Renal num. at risk
time to autoimmunity
all immunotherapy drugs
P=0.071 (matched)
P=2e−04 (unmatched)
income_tier_5
Renal num. at risk
time to autoimmunity
Pembrolizumab
P=0.6767 (matched)
P=0.5653 (unmatched)
income_tier_5
Renal num. at risk
time to autoimmunity
Nivolumab
P=0.0454 (matched)
P=2e−04 (unmatched)
income_tier_5
Renal num. at risk
autoimmune−free %
Months after therapy initiation
matched
unmatched
target therapy
all immuno
Pembrolizumab
Nivolumab

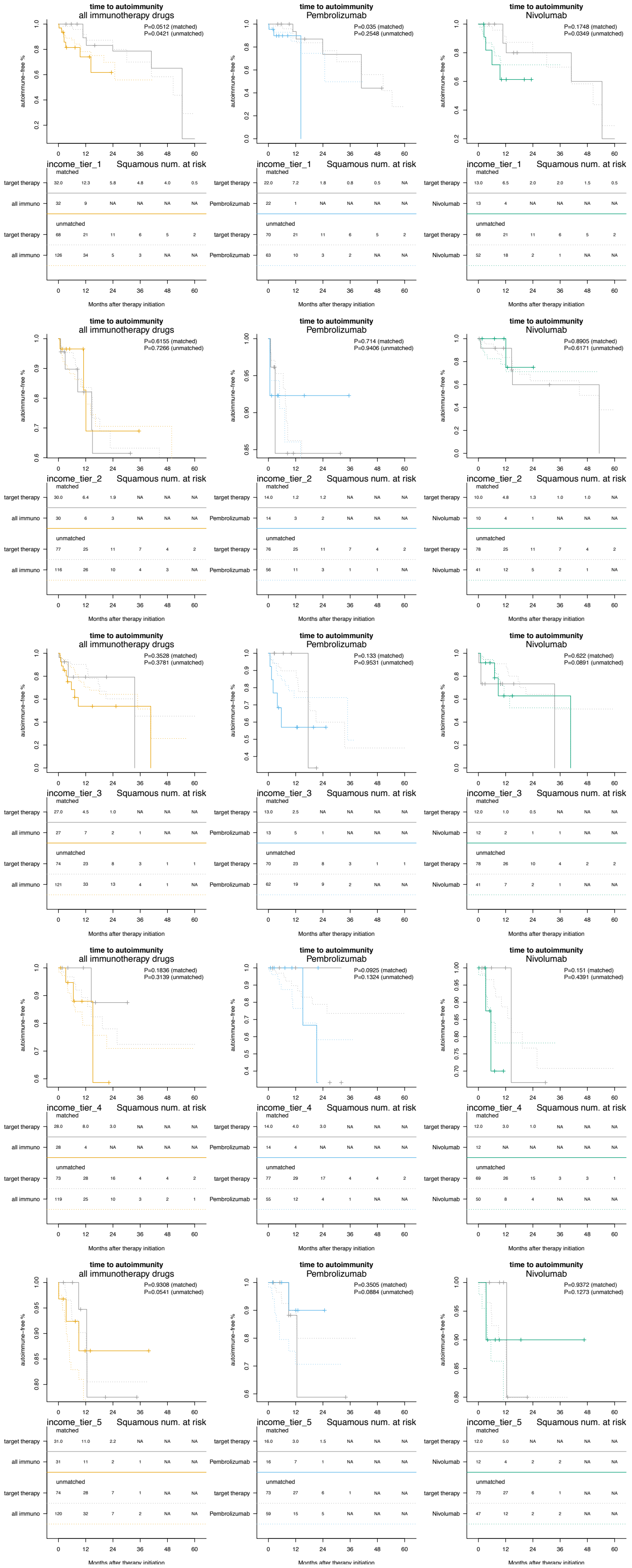

time to autoimmunity
all immunotherapy drugs
P=0.0512 (matched)
P=0.0421 (unmatched)
time to autoimmunity
Pembrolizumab
P=0.035 (matched)
P=0.2548 (unmatched)
time to autoimmunity
Nivolumab
P=0.1748 (matched)
P=0.0349 (unmatched)
income_tier_1
Squamous num. at risk
time to autoimmunity
all immunotherapy drugs
P=0.6155 (matched)
P=0.7266 (unmatched)
time to autoimmunity
Pembrolizumab
P=0.714 (matched)
P=0.9406 (unmatched)
time to autoimmunity
Nivolumab
P=0.8905 (matched)
P=0.6171 (unmatched)
income_tier_2
Squamous num. at risk
time to autoimmunity
all immunotherapy drugs
P=0.3528 (matched)
P=0.3781 (unmatched)
time to autoimmunity
Pembrolizumab
P=0.133 (matched)
P=0.9531 (unmatched)
time to autoimmunity
Nivolumab
P=0.622 (matched)
P=0.0891 (unmatched)
income_tier_3
Squamous num. at risk
time to autoimmunity
all immunotherapy drugs
P=0.1836 (matched)
P=0.3139 (unmatched)
time to autoimmunity
Pembrolizumab
P=0.0925 (matched)
P=0.1324 (unmatched)
time to autoimmunity
Nivolumab
P=0.151 (matched)
P=0.4391 (unmatched)
income_tier_4
Squamous num. at risk
time to autoimmunity
all immunotherapy drugs
P=0.9308 (matched)
P=0.0541 (unmatched)
time to autoimmunity
Pembrolizumab
P=0.3505 (matched)
P=0.0884 (unmatched)
time to autoimmunity
Nivolumab
P=0.9372 (matched)
P=0.1273 (unmatched)
income_tier_5
Squamous num. at risk
autoimmune−free %
matched
unmatched
target therapy
all immuno
Pembrolizumab
Nivolumab
Months after therapy initiation

Figure S14. The number of patients undergoing chemotherapy, targeted therapy, and immunotherapy across different types of cancers. The seven colors of bars represent the number of patients who are treated with chemotherapy, targeted therapy, chemotherapy/ targeted therapy, immunotherapy, and the number of matched immunotherapy patients when matching with three corresponding control groups. The horizontal line indicates a patient count of 200.

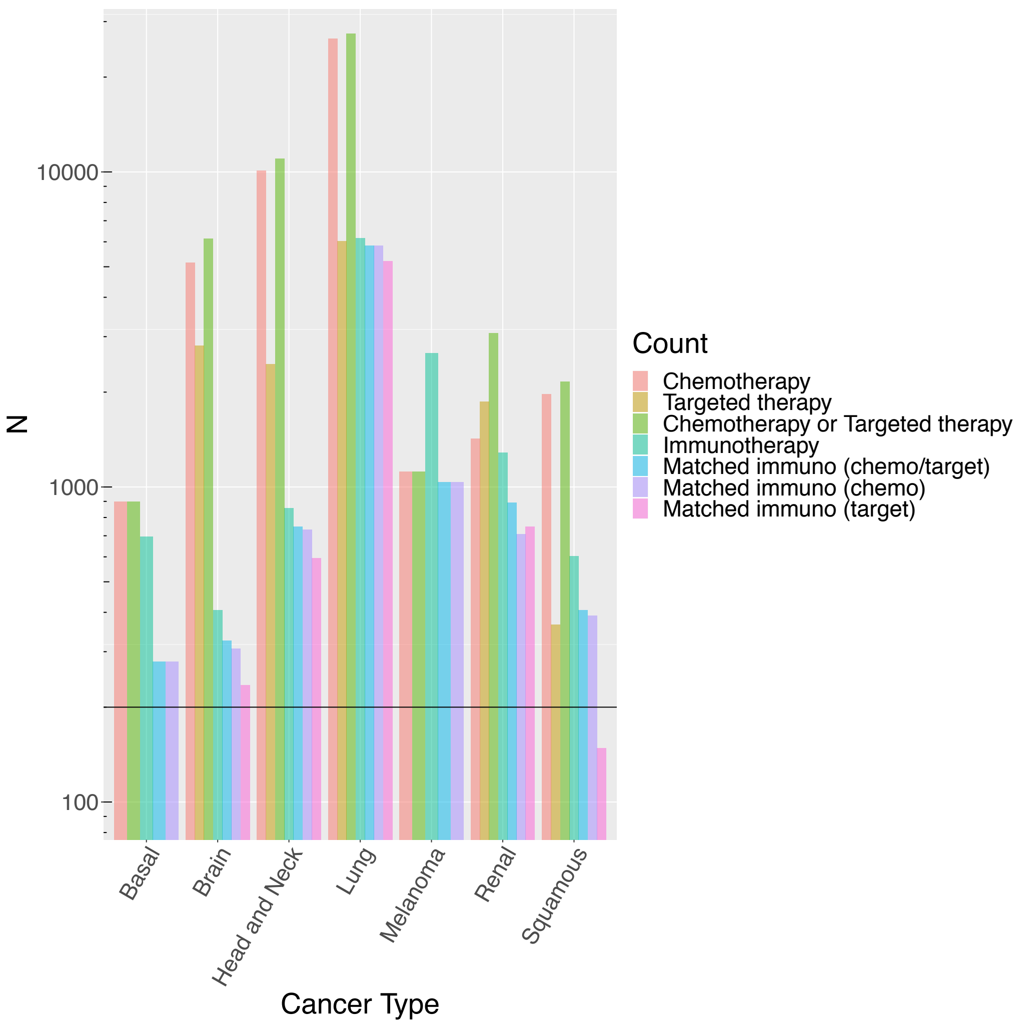

Figure S15. Time-to-event plot showing the time between treatment initiation and the development of immune adverse events with disorders of the thyroid gland as one group.

Chemotherapy

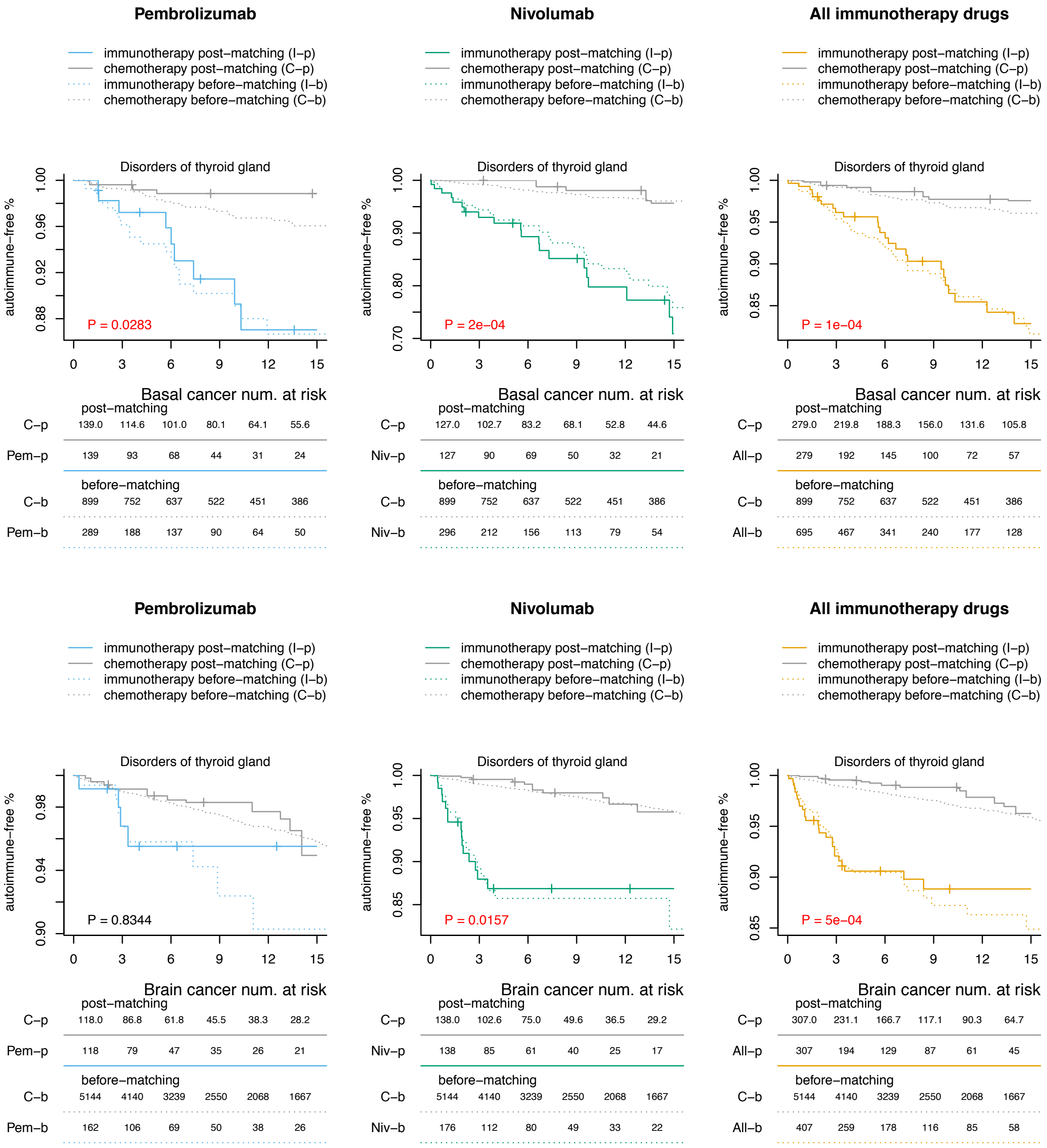

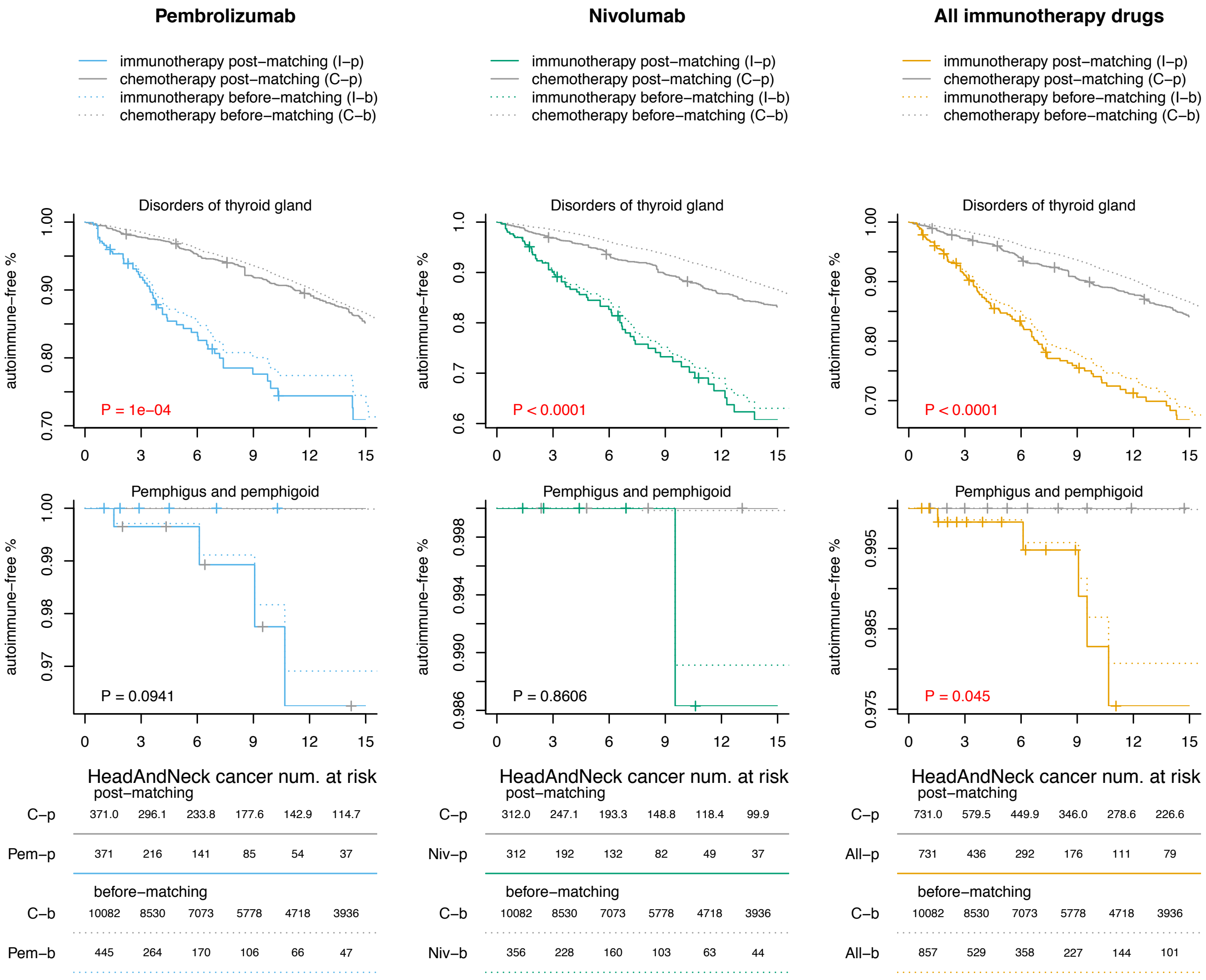
Pembrolizumab
Nivolumab
All immunotherapy drugs
immunotherapy post−matching (I−p)
chemotherapy post−matching (C−p)
immunotherapy before−matching (I−b)
chemotherapy before−matching (C−b)
Disorders of thyroid gland
Pemphigus and pemphigoid
autoimmune−free %
P = 1e−04
P < 0.0001
P < 0.0001
P = 0.0941
P = 0.8606
P = 0.045
HeadAndNeck cancer num. at risk
post−matching
before−matching
C−p 371.0 296.1 233.8 177.6 142.9 114.7
Pem−p 371 216 141 85 54 37
C−b 10082 8530 7073 5778 4718 3936
Pem−b 445 264 170 106 66 47
C−p 312.0 247.1 193.3 148.8 118.4 99.9
Niv−p 312 192 132 82 49 37
C−b 10082 8530 7073 5778 4718 3936
Niv−b 356 228 160 103 63 44
C−p 731.0 579.5 449.9 346.0 278.6 226.6
All−p 731 436 292 176 111 79
C−b 10082 8530 7073 5778 4718 3936
All−b 857 529 358 227 144 101

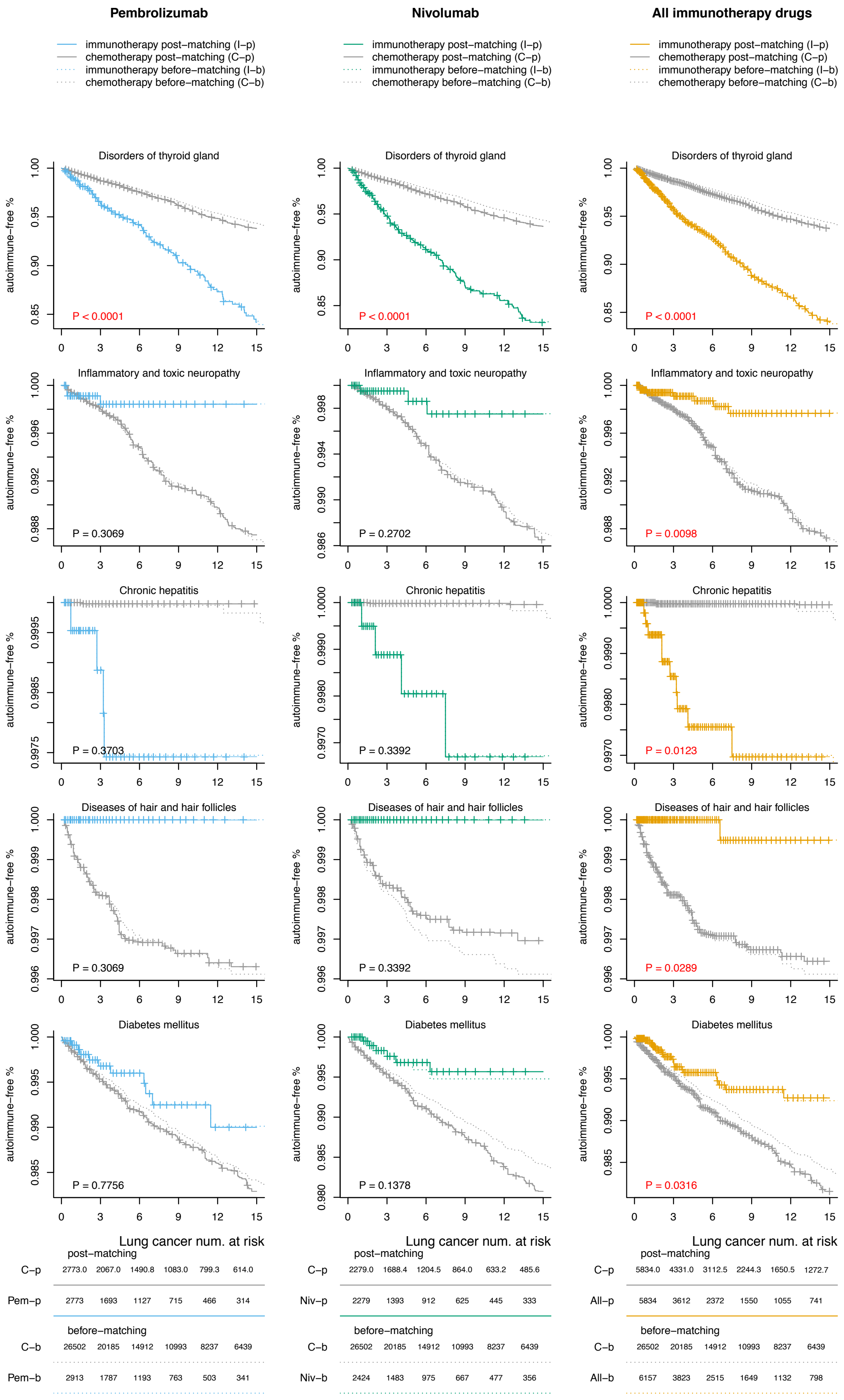
Pembrolizumab
Nivolumab
All immunotherapy drugs
immunotherapy post−matching (I−p)
chemotherapy post−matching (C−p)
immunotherapy before−matching (I−b)
chemotherapy before−matching (C−b)
Disorders of thyroid gland
Inflammatory and toxic neuropathy
Chronic hepatitis
Diseases of hair and hair follicles
Diabetes mellitus
autoimmune−free %
Pembrolizumab: P < 0.0001; P = 0.3069; P = 0.3703; P = 0.3069; P = 0.7756
Nivolumab: P < 0.0001; P = 0.2702; P = 0.3392; P = 0.3392; P = 0.1378
All immunotherapy drugs: P < 0.0001; P = 0.0098; P = 0.0123; P = 0.0289; P = 0.0316
Lung cancer num. at risk
post−matching
C−p 2773.0 2067.0 1490.8 1083.0 799.3 614.0
Pem−p 2773 1693 1127 715 466 314
C−p 2279.0 1688.4 1204.5 864.0 633.2 485.6
Niv−p 2279 1393 912 625 445 333
C−p 5834.0 4331.0 3112.5 2244.3 1650.5 1272.7
All−p 5834 3612 2372 1550 1055 741
before−matching
C−b 26502 20185 14912 10993 8237 6439
Pem−b 2913 1787 1193 763 503 341
Niv−b 2424 1483 975 667 477 356
All−b 6157 3823 2515 1649 1132 798

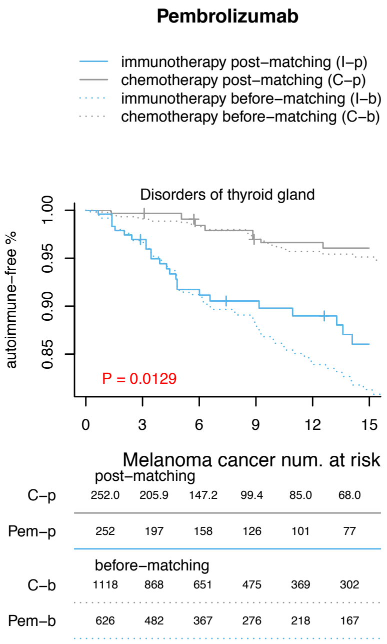
Pembrolizumab
immunotherapy post−matching (I−p)
chemotherapy post−matching (C−p)
immunotherapy before−matching (I−b)
chemotherapy before−matching (C−b)
Disorders of thyroid gland
autoimmune−free %
1.00
0.95
0.90
0.85
P = 0.0129
0 3 6 9 12 15
Melanoma cancer num. at risk
post−matching
C−p 252.0 205.9 147.2 99.4 85.0 68.0
Pem−p 252 197 158 126 101 77
before−matching
C−b 1118 868 651 475 369 302
Pem−b 626 482 367 276 218 167

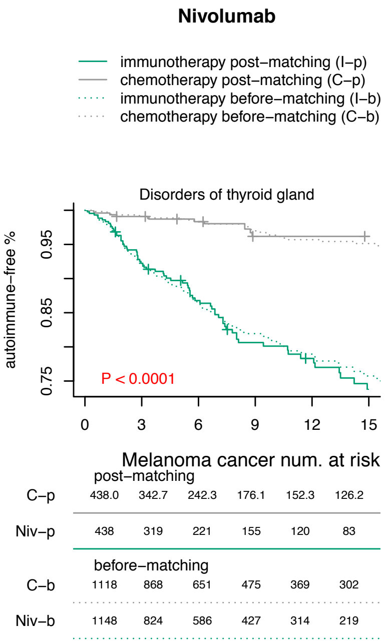
Nivolumab
immunotherapy post−matching (I−p)
chemotherapy post−matching (C−p)
immunotherapy before−matching (I−b)
chemotherapy before−matching (C−b)
Disorders of thyroid gland
autoimmune−free %
0.95
0.85
0.75
P < 0.0001
0 3 6 9 12 15
Melanoma cancer num. at risk
post−matching
C−p 438.0 342.7 242.3 176.1 152.3 126.2
Niv−p 438 319 221 155 120 83
before−matching
C−b 1118 868 651 475 369 302
Niv−b 1148 824 586 427 314 219

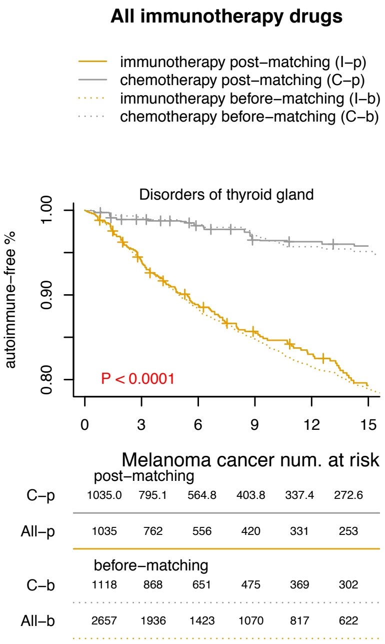
All immunotherapy drugs
immunotherapy post−matching (I−p)
chemotherapy post−matching (C−p)
immunotherapy before−matching (I−b)
chemotherapy before−matching (C−b)
Disorders of thyroid gland
autoimmune−free %
1.00
0.90
0.80
P < 0.0001
0 3 6 9 12 15
Melanoma cancer num. at risk
post−matching
C−p 1035.0 795.1 564.8 403.8 337.4 272.6
All−p 1035 762 556 420 331 253
before−matching
C−b 1118 868 651 475 369 302
All−b 2657 1936 1423 1070 817 622

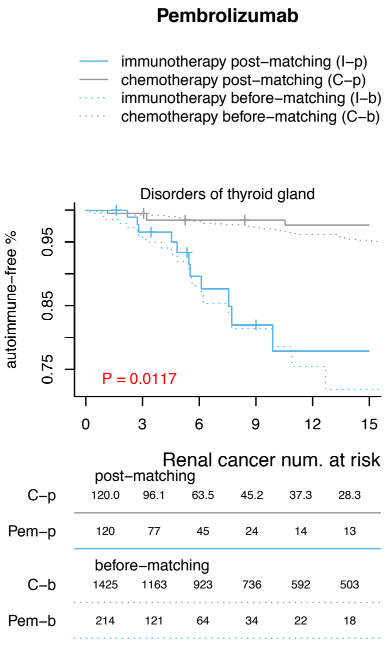
Pembrolizumab
immunotherapy post−matching (I−p)
chemotherapy post−matching (C−p)
immunotherapy before−matching (I−b)
chemotherapy before−matching (C−b)
Disorders of thyroid gland
autoimmune−free %
0.95
0.85
0.75
P = 0.0117
0 3 6 9 12 15
Renal cancer num. at risk
post−matching
C−p 120.0 96.1 63.5 45.2 37.3 28.3
Pem−p 120 77 45 24 14 13
before−matching
C−b 1425 1163 923 736 592 503
Pem−b 214 121 64 34 22 18

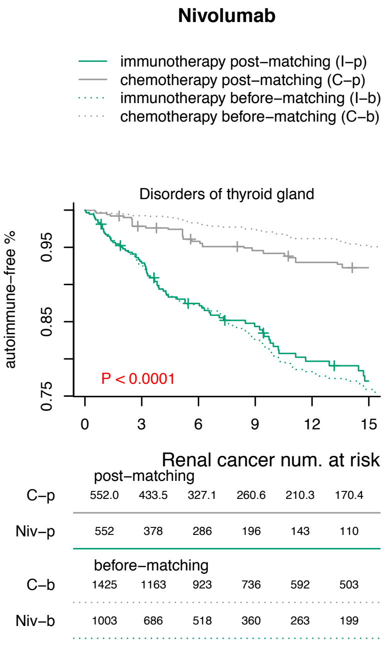
Nivolumab
immunotherapy post−matching (I−p)
chemotherapy post−matching (C−p)
immunotherapy before−matching (I−b)
chemotherapy before−matching (C−b)
Disorders of thyroid gland
autoimmune−free %
0.95
0.85
0.75
P < 0.0001
0 3 6 9 12 15
Renal cancer num. at risk
post−matching
C−p 552.0 433.5 327.1 260.6 210.3 170.4
Niv−p 552 378 286 196 143 110
before−matching
C−b 1425 1163 923 736 592 503
Niv−b 1003 686 518 360 263 199

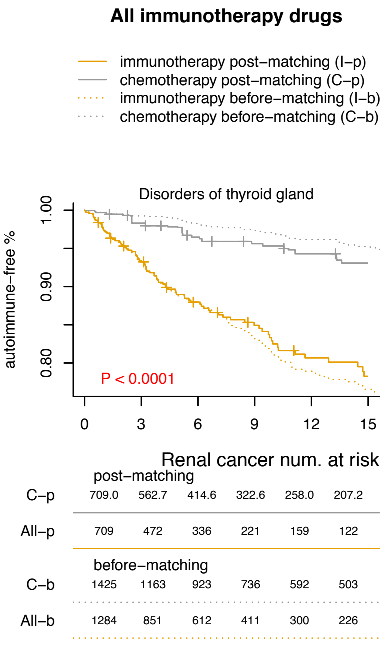
All immunotherapy drugs
immunotherapy post−matching (I−p)
chemotherapy post−matching (C−p)
immunotherapy before−matching (I−b)
chemotherapy before−matching (C−b)
Disorders of thyroid gland
autoimmune−free %
1.00
0.90
0.80
P < 0.0001
0 3 6 9 12 15
Renal cancer num. at risk
post−matching
C−p 709.0 562.7 414.6 322.6 258.0 207.2
All−p 709 472 336 221 159 122
before−matching
C−b 1425 1163 923 736 592 503
All−b 1284 851 612 411 300 226

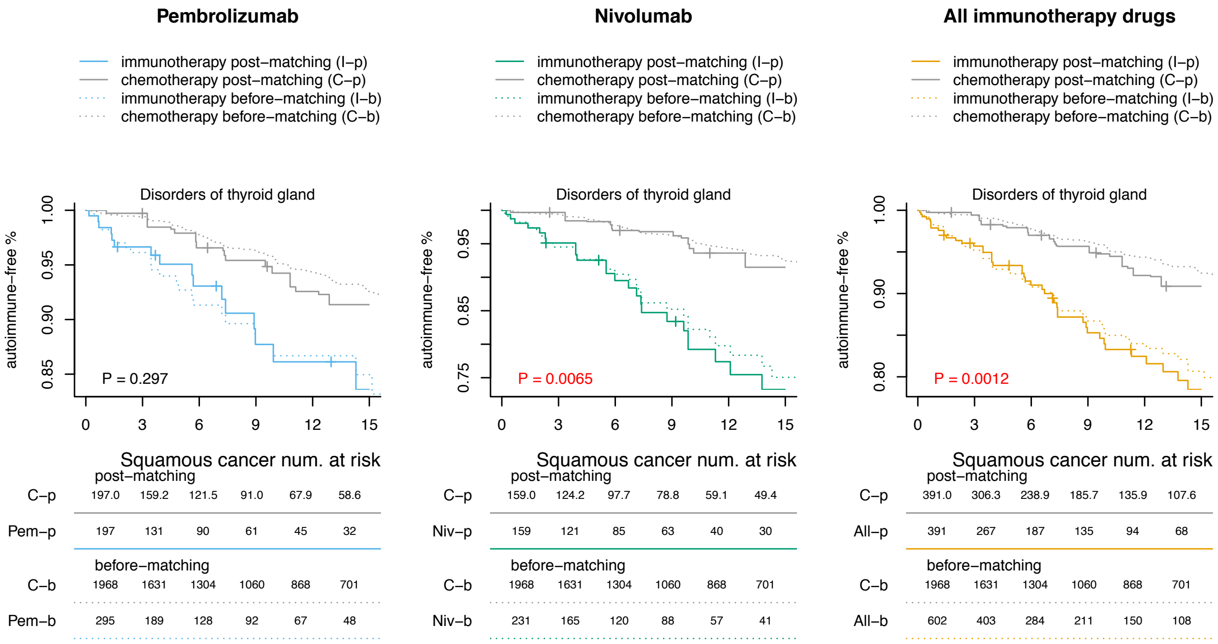
Pembrolizumab
Nivolumab
All immunotherapy drugs
immunotherapy post−matching (I−p)
chemotherapy post−matching (C−p)
immunotherapy before−matching (I−b)
chemotherapy before−matching (C−b)
Disorders of thyroid gland
autoimmune−free %
P = 0.297
P = 0.0065
P = 0.0012
0 3 6 9 12 15
Squamous cancer num. at risk
post−matching
C−p 197.0 159.2 121.5 91.0 67.9 58.6
Pem−p 197 131 90 61 45 32
before−matching
C−b 1968 1631 1304 1060 868 701
Pem−b 295 189 128 92 67 48
C−p 159.0 124.2 97.7 78.8 59.1 49.4
Niv−p 159 121 85 63 40 30
C−b 1968 1631 1304 1060 868 701
Niv−b 231 165 120 88 57 41
C−p 391.0 306.3 238.9 185.7 135.9 107.6
All−p 391 267 187 135 94 68
C−b 1968 1631 1304 1060 868 701
All−b 602 403 284 211 150 108

Targeted therapy

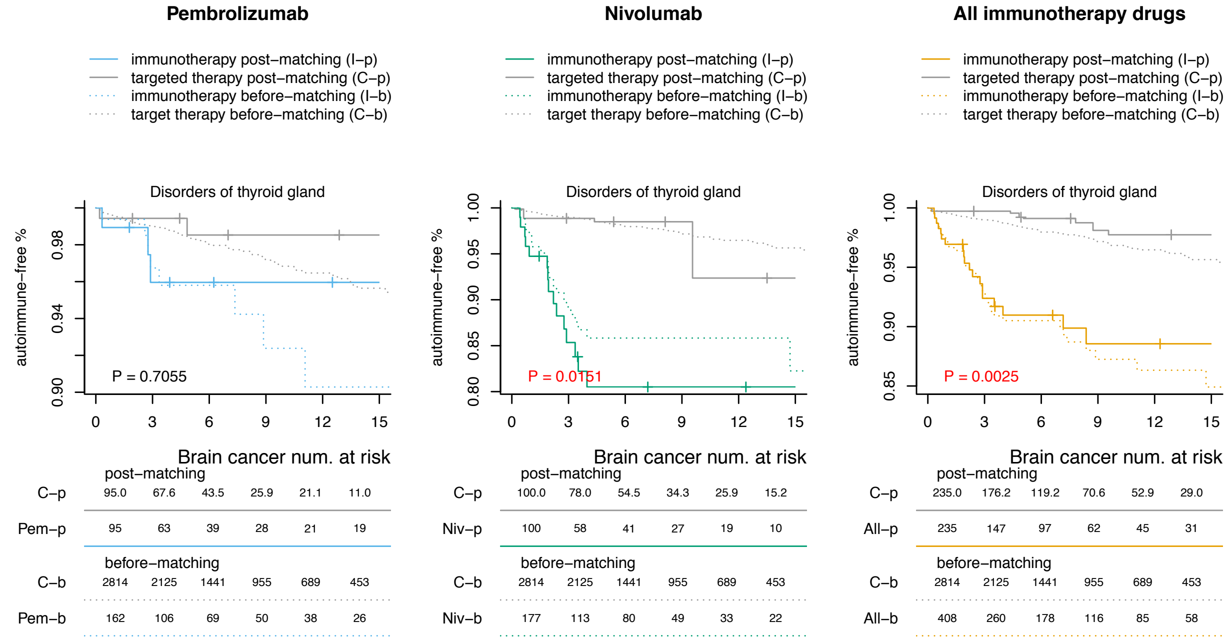

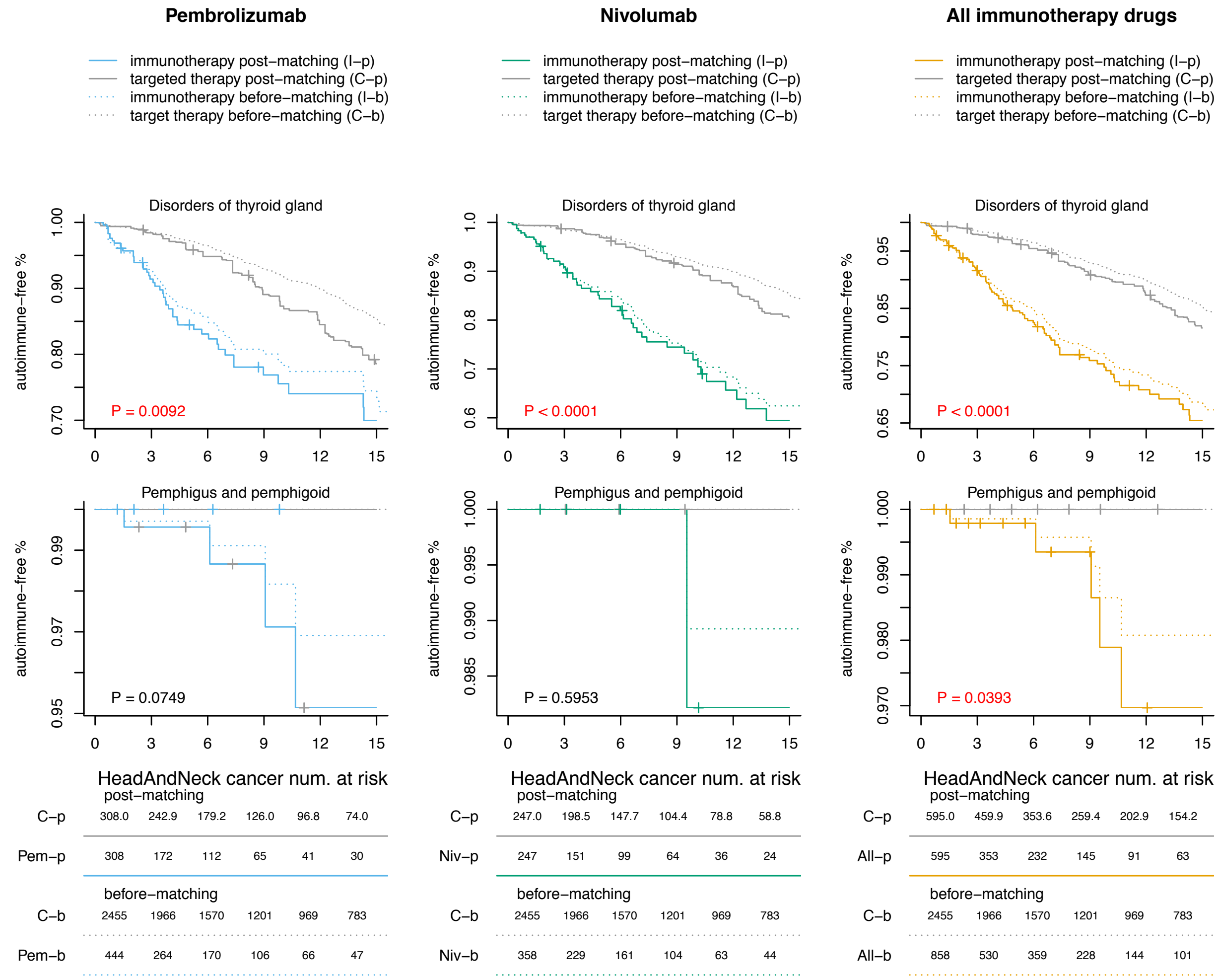

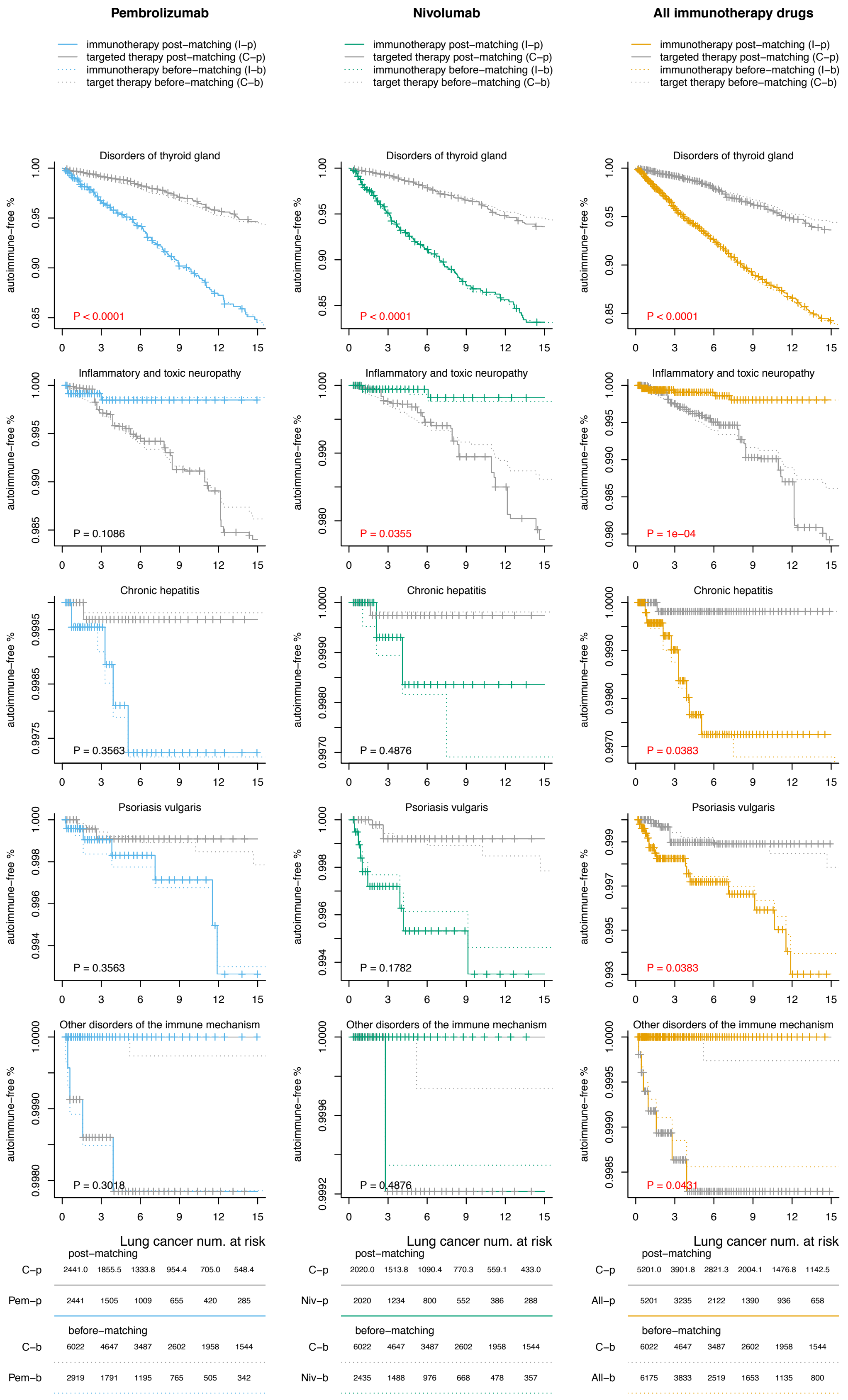

Pembrolizumab
Nivolumab
All immunotherapy drugs
immunotherapy post−matching (I−p)
targeted therapy post−matching (C−p)
immunotherapy before−matching (I−b)
target therapy before−matching (C−b)
Disorders of thyroid gland
Inflammatory and toxic neuropathy
Chronic hepatitis
Psoriasis vulgaris
Other disorders of the immune mechanism
autoimmune−free %
P < 0.0001
P = 0.1086
P = 0.0355
P = 1e−04
P = 0.3563
P = 0.4876
P = 0.0383
P = 0.1782
P = 0.3018
P = 0.0431
Lung cancer num. at risk
post−matching
before−matching
C−p 2441.0 1855.5 1333.8 954.4 705.0 548.4
Pem−p 2441 1505 1009 655 420 285
C−b 6022 4647 3487 2602 1958 1544
Pem−b 2919 1791 1195 765 505 342
C−p 2020.0 1513.8 1090.4 770.3 559.1 433.0
Niv−p 2020 1234 800 552 386 288
C−b 6022 4647 3487 2602 1958 1544
Niv−b 2435 1488 976 668 478 357
C−p 5201.0 3901.8 2821.3 2004.1 1476.8 1142.5
All−p 5201 3235 2122 1390 936 658
C−b 6022 4647 3487 2602 1958 1544
All−b 6175 3833 2519 1653 1135 800

Pembrolizumab

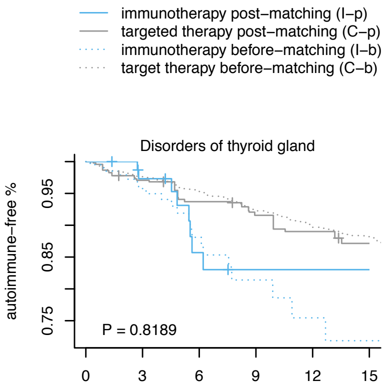

Renal cancer num. at risk

post−matching						
C−p	120.0	85.7	62.1	45.5	31.3	25.6
Pem−p	120	67	32	19	11	9
before−matching						
C−b	1868	1412	1041	794	597	486
Pem−b	214	121	64	34	22	18

Nivolumab

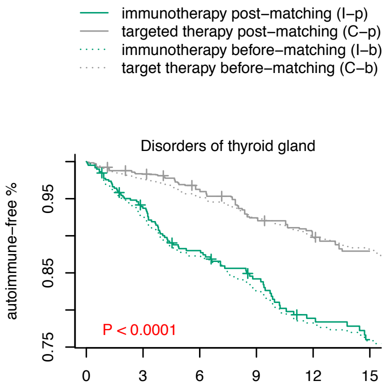

Renal cancer num. at risk

post−matching						
C−p	606.0	459.4	322.4	236.2	174.7	141.2
Niv−p	606	417	315	222	161	121
before−matching						
C−b	1868	1412	1041	794	597	486
Niv−b	1002	685	517	359	262	198

All immunotherapy drugs

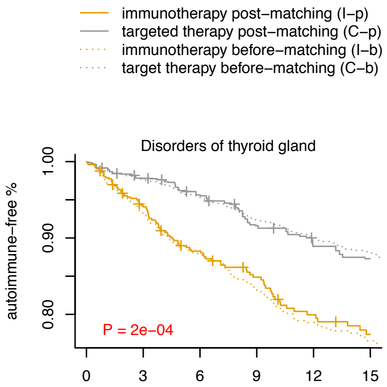

Renal cancer num. at risk

post−matching						
C−p	749.0	555.1	388.2	280.0	201.4	161.1
All−p	749	500	358	246	175	131
before−matching						
C−b	1868	1412	1041	794	597	486
All−b	1284	851	612	411	300	225

Pembrolizumab

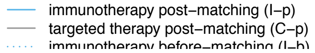

Squamous cancer num. at risk

post−matching						
C−p	79.0	52.2	40.9	30.6	17.9	15.4
Pem−p	79	53	34	24	20	11
before−matching						
C−b	366	274	222	168	125	94
Pem−b	295	189	128	92	67	48

Nivolumab

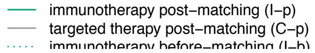

Squamous cancer num. at risk

post−matching						
C−p	59.0	41.7	35.2	30.8	20.3	12.3
Niv−p	59	49	33	24	14	8
before−matching						
C−b	366	274	222	168	125	94
Niv−b	231	165	120	88	57	41

All immunotherapy drugs

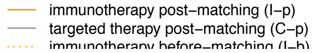

Squamous cancer num. at risk

post−matching						
C−p	148.0	95.9	79.2	63.8	42.2	31.9
All−p	148	103	69	50	37	21
before−matching						
C−b	366	274	222	168	125	94
All−b	602	403	284	211	150	108

Figure S16. Time-to-event plot showing the time between treatment initiation and the development of immune adverse event groups.

Chemotherapy

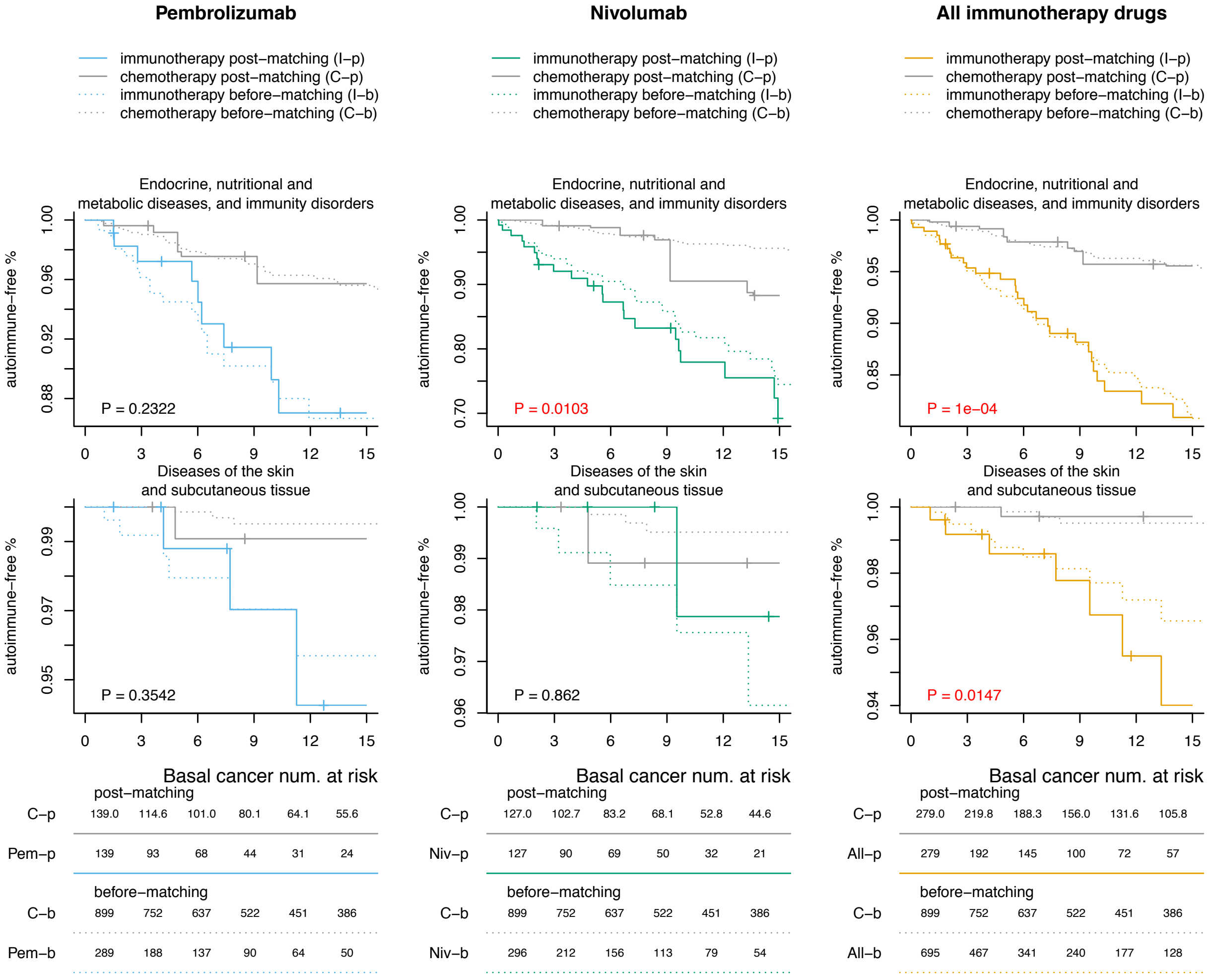

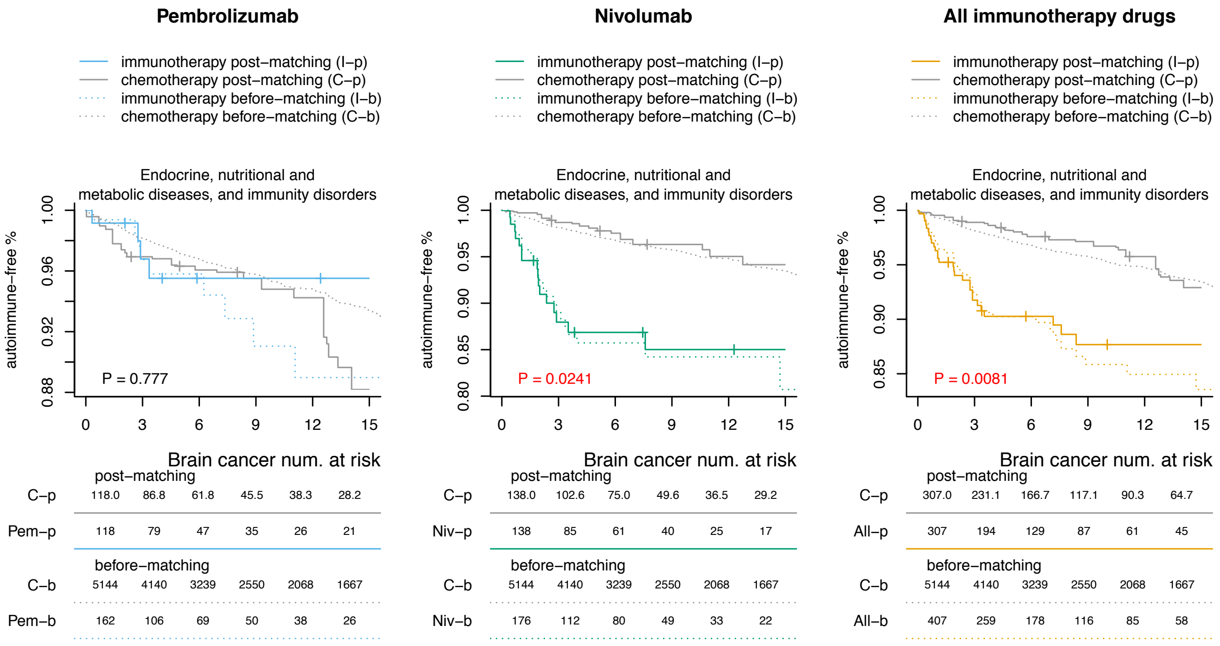
Pembrolizumab
immunotherapy post−matching (I−p)
chemotherapy post−matching (C−p)
immunotherapy before−matching (I−b)
chemotherapy before−matching (C−b)
Endocrine, nutritional and metabolic diseases, and immunity disorders
autoimmune−free %
P = 0.777
Brain cancer num. at risk
post−matching
C−p 118.0 86.8 61.8 45.5 38.3 28.2
Pem−p 118 79 47 35 26 21
before−matching
C−b 5144 4140 3239 2550 2068 1667
Pem−b 162 106 69 50 38 26
Nivolumab
immunotherapy post−matching (I−p)
chemotherapy post−matching (C−p)
immunotherapy before−matching (I−b)
chemotherapy before−matching (C−b)
Endocrine, nutritional and metabolic diseases, and immunity disorders
autoimmune−free %
P = 0.0241
Brain cancer num. at risk
post−matching
C−p 138.0 102.6 75.0 49.6 36.5 29.2
Niv−p 138 85 61 40 25 17
before−matching
C−b 5144 4140 3239 2550 2068 1667
Niv−b 176 112 80 49 33 22
All immunotherapy drugs
immunotherapy post−matching (I−p)
chemotherapy post−matching (C−p)
immunotherapy before−matching (I−b)
chemotherapy before−matching (C−b)
Endocrine, nutritional and metabolic diseases, and immunity disorders
autoimmune−free %
P = 0.0081
Brain cancer num. at risk
post−matching
C−p 307.0 231.1 166.7 117.1 90.3 64.7
All−p 307 194 129 87 61 45
before−matching
C−b 5144 4140 3239 2550 2068 1667
All−b 407 259 178 116 85 58

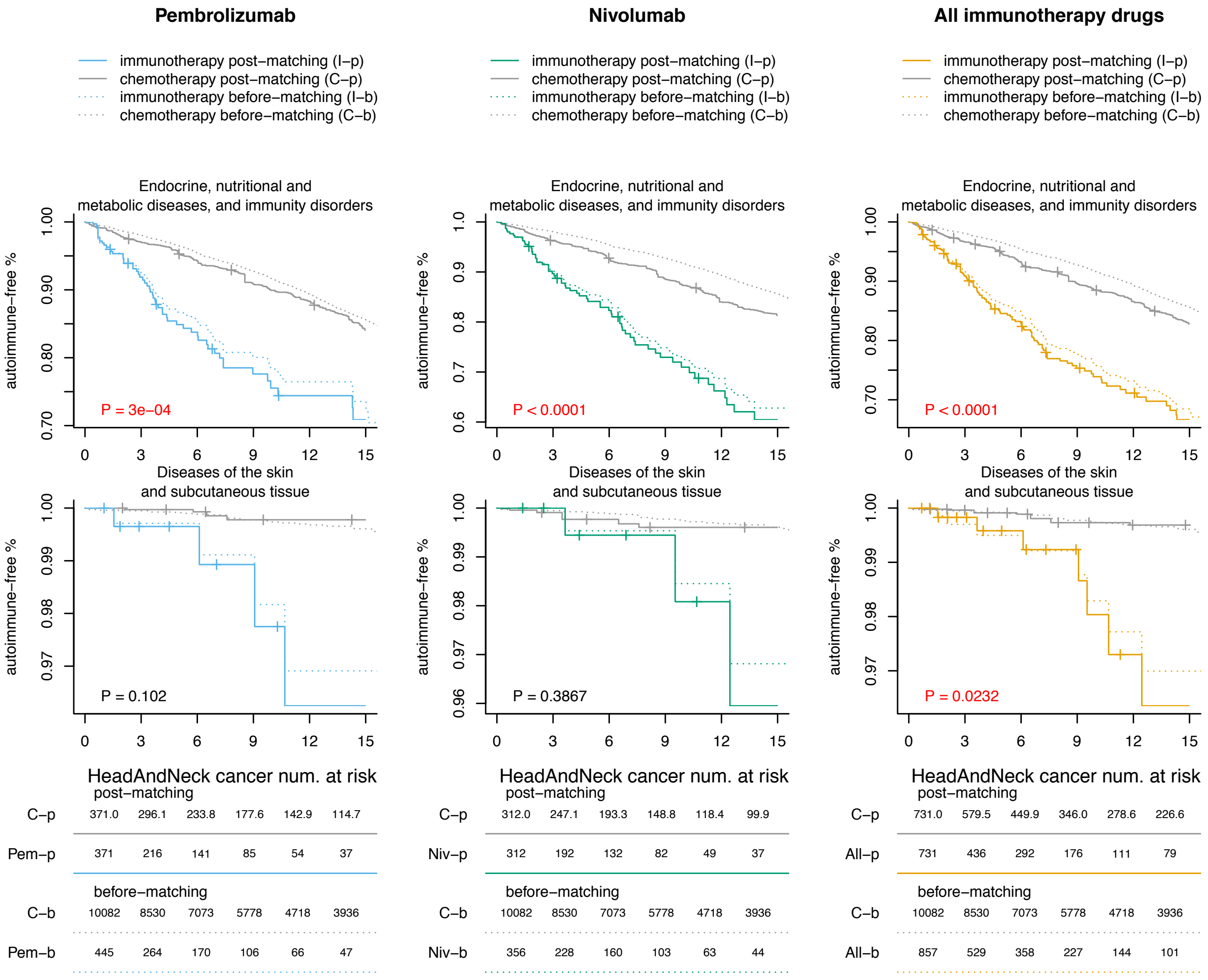
Pembrolizumab
immunotherapy post−matching (I−p)
chemotherapy post−matching (C−p)
immunotherapy before−matching (I−b)
chemotherapy before−matching (C−b)
Endocrine, nutritional and metabolic diseases, and immunity disorders
autoimmune−free %
P = 3e−04
Diseases of the skin and subcutaneous tissue
autoimmune−free %
P = 0.102
HeadAndNeck cancer num. at risk
post−matching
C−p 371.0 296.1 233.8 177.6 142.9 114.7
Pem−p 371 216 141 85 54 37
before−matching
C−b 10082 8530 7073 5778 4718 3936
Pem−b 445 264 170 106 66 47
Nivolumab
immunotherapy post−matching (I−p)
chemotherapy post−matching (C−p)
immunotherapy before−matching (I−b)
chemotherapy before−matching (C−b)
Endocrine, nutritional and metabolic diseases, and immunity disorders
autoimmune−free %
P < 0.0001
Diseases of the skin and subcutaneous tissue
autoimmune−free %
P = 0.3867
HeadAndNeck cancer num. at risk
post−matching
C−p 312.0 247.1 193.3 148.8 118.4 99.9
Niv−p 312 192 132 82 49 37
before−matching
C−b 10082 8530 7073 5778 4718 3936
Niv−b 356 228 160 103 63 44
All immunotherapy drugs
immunotherapy post−matching (I−p)
chemotherapy post−matching (C−p)
immunotherapy before−matching (I−b)
chemotherapy before−matching (C−b)
Endocrine, nutritional and metabolic diseases, and immunity disorders
autoimmune−free %
P < 0.0001
Diseases of the skin and subcutaneous tissue
autoimmune−free %
P = 0.0232
HeadAndNeck cancer num. at risk
post−matching
C−p 731.0 579.5 449.9 346.0 278.6 226.6
All−p 731 436 292 176 111 79
before−matching
C−b 10082 8530 7073 5778 4718 3936
All−b 857 529 358 227 144 101

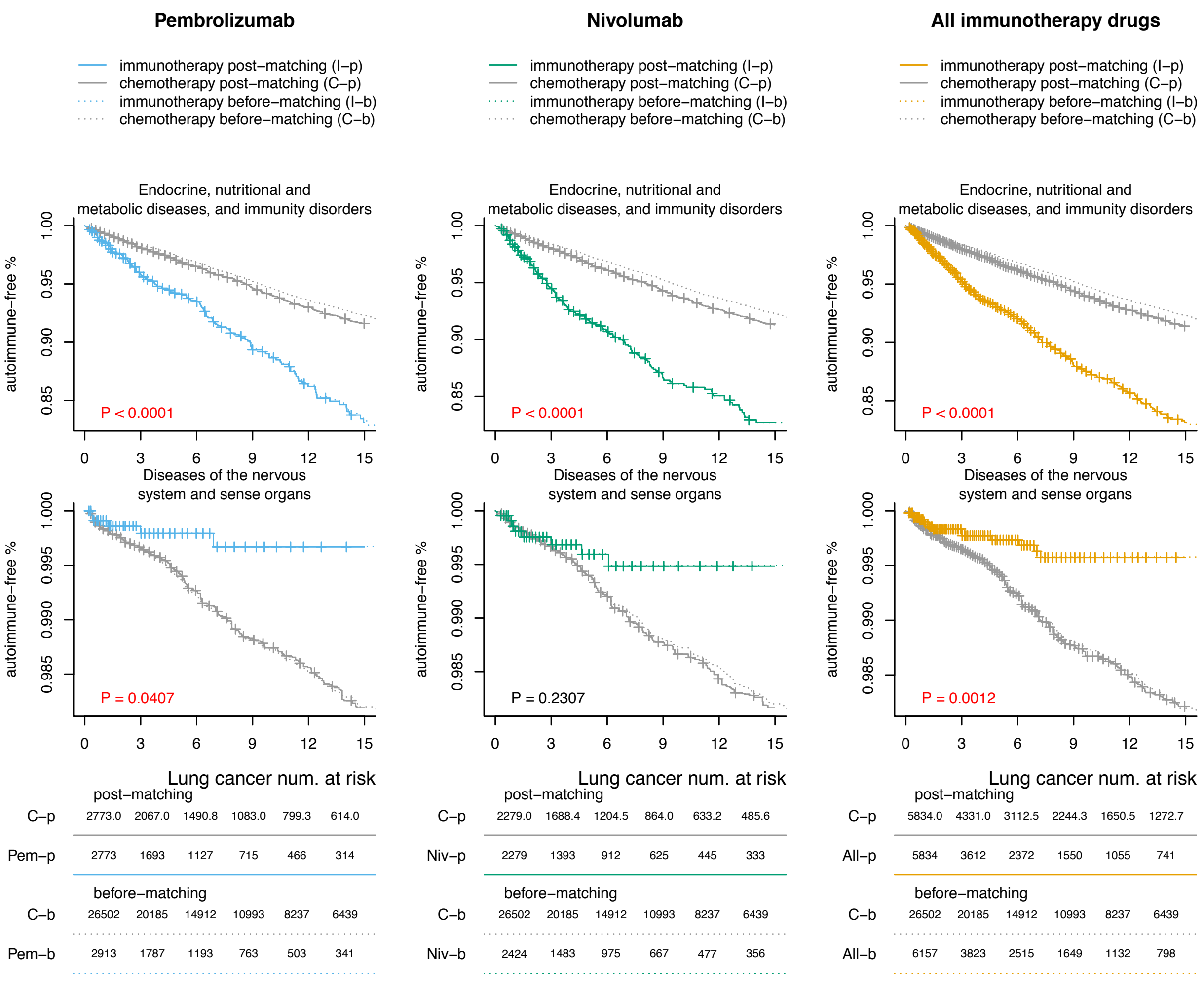
Pembrolizumab
Nivolumab
All immunotherapy drugs
immunotherapy post−matching (I−p)
chemotherapy post−matching (C−p)
immunotherapy before−matching (I−b)
chemotherapy before−matching (C−b)
Endocrine, nutritional and metabolic diseases, and immunity disorders
Diseases of the nervous system and sense organs
autoimmune−free %
P < 0.0001
P = 0.0407
P = 0.2307
P = 0.0012
Lung cancer num. at risk
post−matching
C−p 2773.0 2067.0 1490.8 1083.0 799.3 614.0
Pem−p 2773 1693 1127 715 466 314
before−matching
C−b 26502 20185 14912 10993 8237 6439
Pem−b 2913 1787 1193 763 503 341
post−matching
C−p 2279.0 1688.4 1204.5 864.0 633.2 485.6
Niv−p 2279 1393 912 625 445 333
before−matching
C−b 26502 20185 14912 10993 8237 6439
Niv−b 2424 1483 975 667 477 356
post−matching
C−p 5834.0 4331.0 3112.5 2244.3 1650.5 1272.7
All−p 5834 3612 2372 1550 1055 741
before−matching
C−b 26502 20185 14912 10993 8237 6439
All−b 6157 3823 2515 1649 1132 798

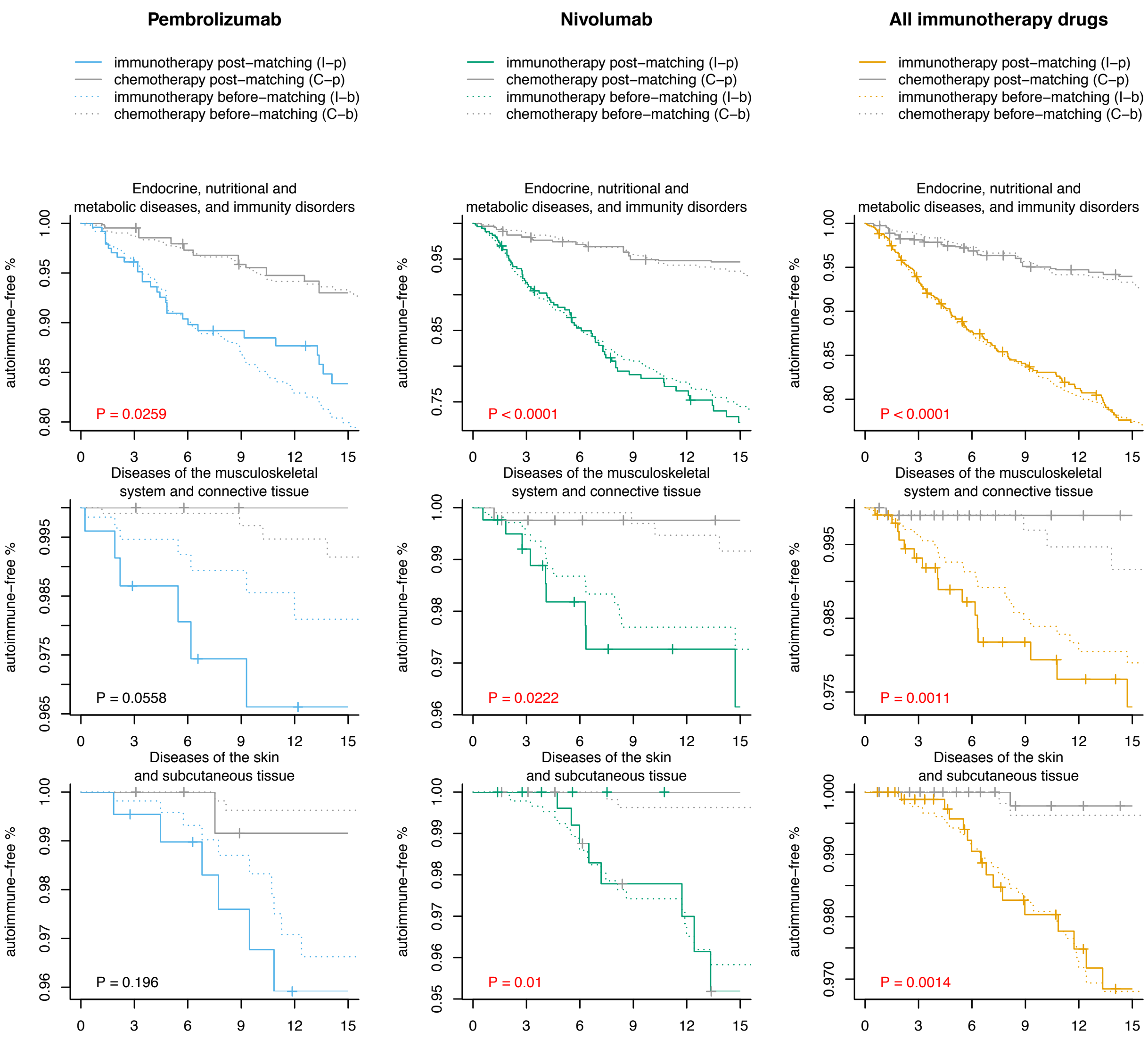

Melanoma cancer num. at risk

	0	3	6	9	12	15
post−matching						
C−p	252.0	205.9	147.2	99.4	85.0	68.0
Pem−p	252	197	158	126	101	77
before−matching						
C−b	1118	868	651	475	369	302
Pem−b	626	482	367	276	218	167

Melanoma cancer num. at risk

	0	3	6	9	12	15
post−matching						
C−p	438.0	342.7	242.3	176.1	152.3	126.2
Niv−p	438	319	221	155	120	83
before−matching						
C−b	1118	868	651	475	369	302
Niv−b	1148	824	586	427	314	219

Melanoma cancer num. at risk

	0	3	6	9	12	15
post−matching						
C−p	1035.0	795.1	564.8	403.8	337.4	272.6
All−p	1035	762	556	420	331	253
before−matching						
C−b	1118	868	651	475	369	302
All−b	2657	1936	1423	1070	817	622

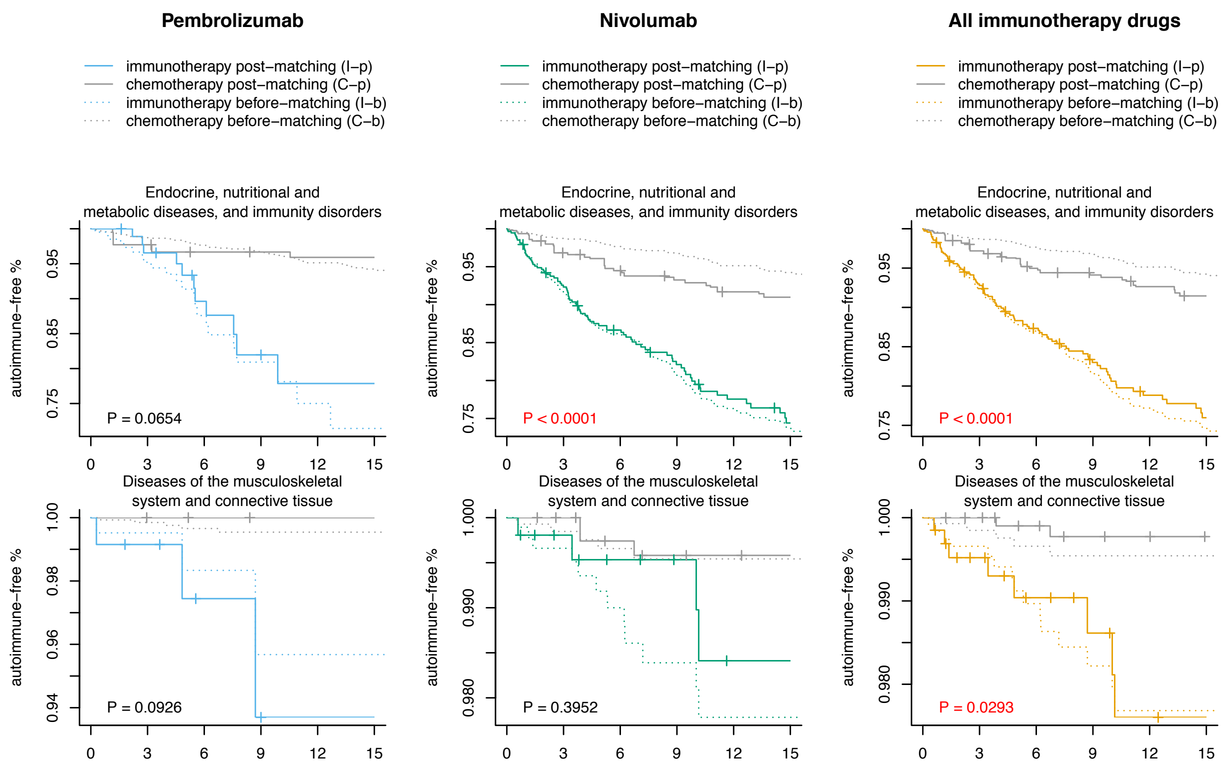

Renal cancer num. at risk

	Pembrolizumab						Nivolumab						All immunotherapy drugs					
post−matching																		
C−p	120.0	96.1	63.5	45.2	37.3	28.3	552.0	433.5	327.1	260.6	210.3	170.4	709.0	562.7	414.6	322.6	258.0	207.2
Pem−p / Niv−p / All−p	120	77	45	24	14	13	552	378	286	196	143	110	709	472	336	221	159	122
before−matching																		
C−b	1425	1163	923	736	592	503	1425	1163	923	736	592	503	1425	1163	923	736	592	503
Pem−b / Niv−b / All−b	214	121	64	34	22	18	1003	686	518	360	263	199	1284	851	612	411	300	226

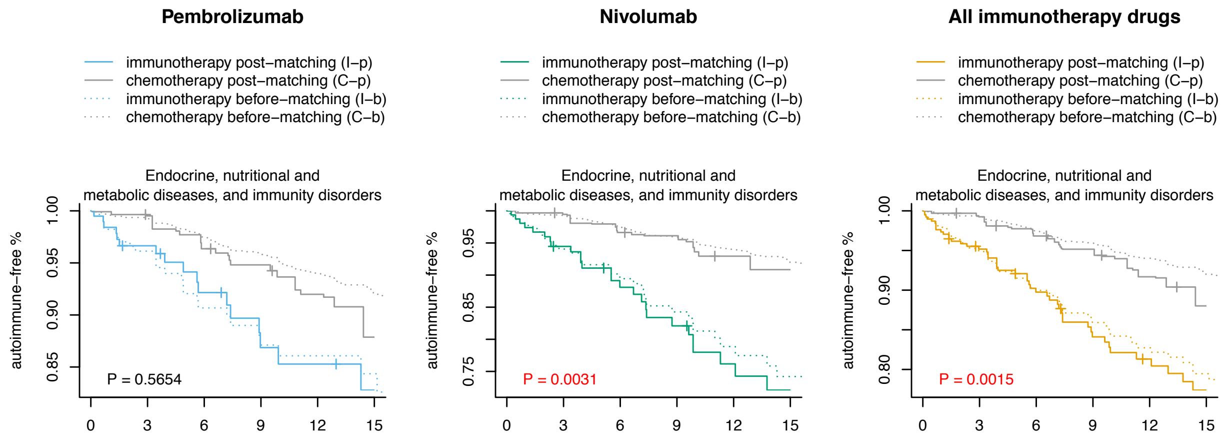

Squamous cancer num. at risk

	Pembrolizumab						Nivolumab						All immunotherapy drugs					
post−matching																		
C−p	197.0	159.2	121.5	91.0	67.9	58.6	159.0	124.2	97.7	78.8	59.1	49.4	391.0	306.3	238.9	185.7	135.9	107.6
Pem−p / Niv−p / All−p	197	131	90	61	45	32	159	121	85	63	40	30	391	267	187	135	94	68
before−matching																		
C−b	1968	1631	1304	1060	868	701	1968	1631	1304	1060	868	701	1968	1631	1304	1060	868	701
Pem−b / Niv−b / All−b	295	189	128	92	67	48	231	165	120	88	57	41	602	403	284	211	150	108

Targeted therapy

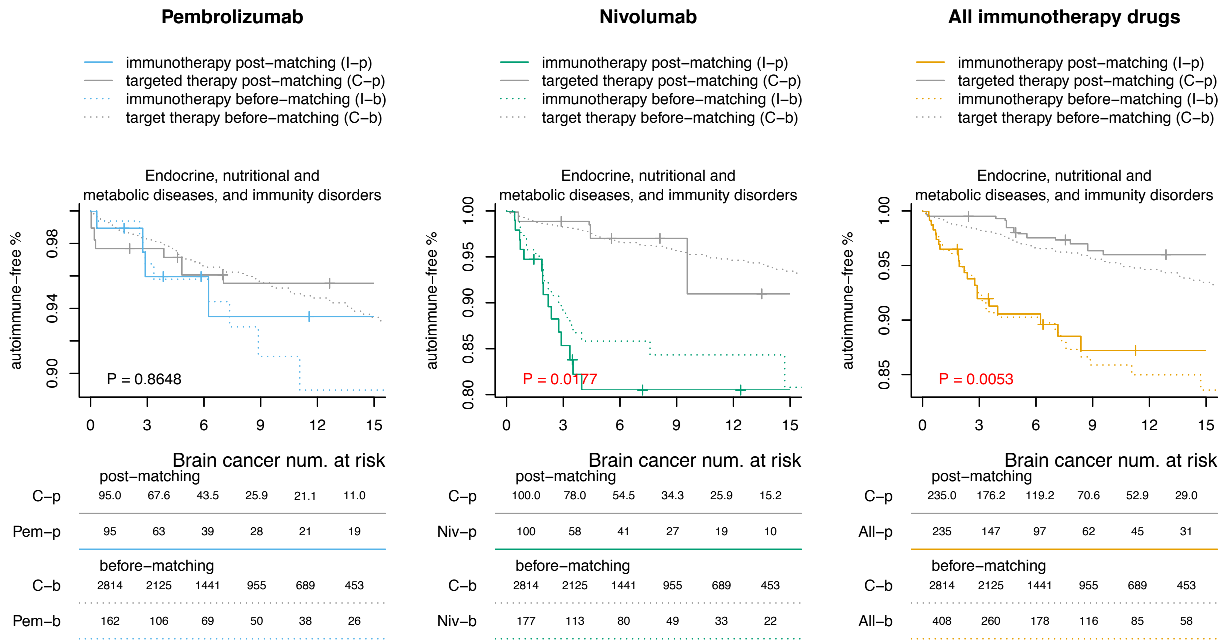

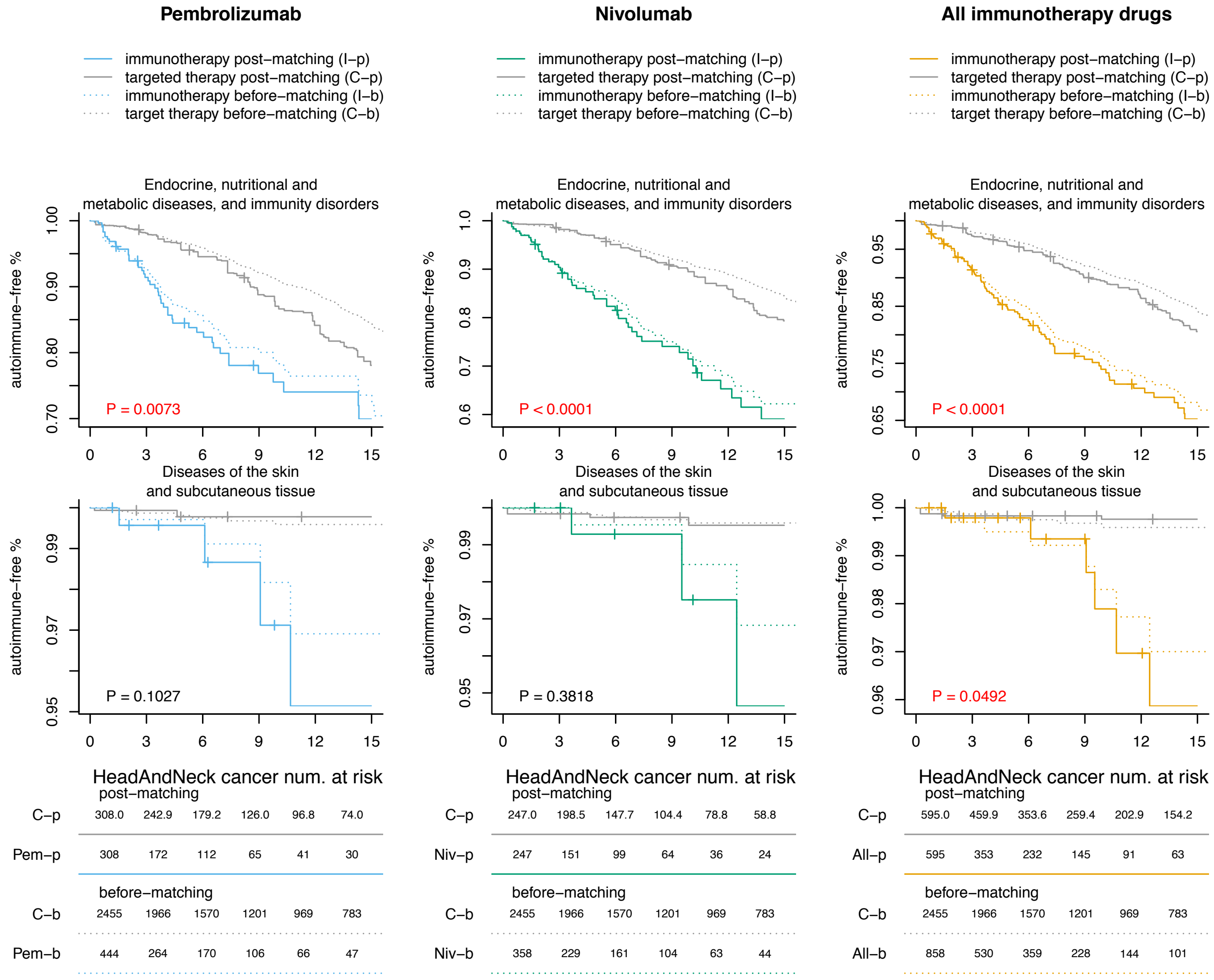

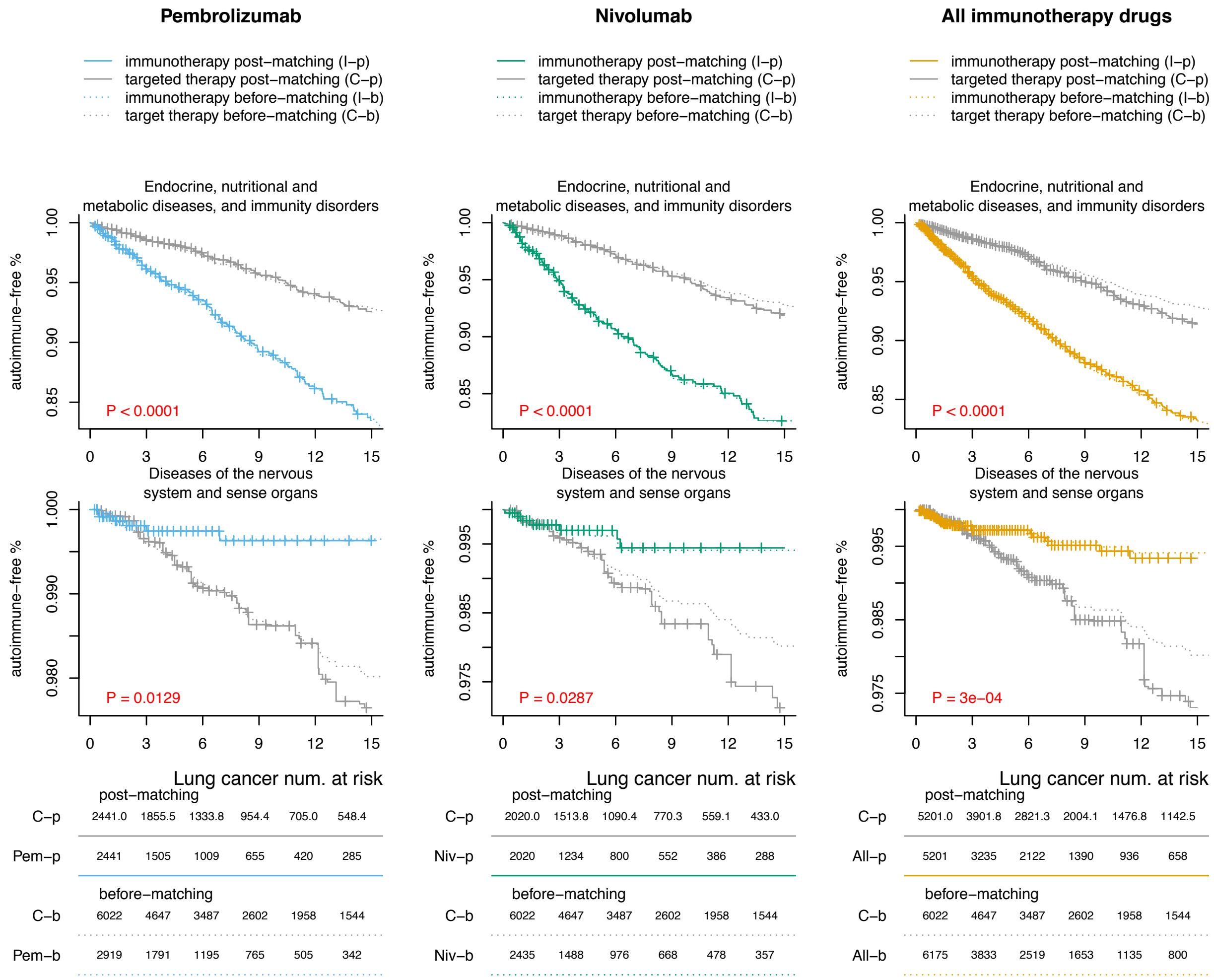
Pembrolizumab
Nivolumab
All immunotherapy drugs
immunotherapy post−matching (I−p)
targeted therapy post−matching (C−p)
immunotherapy before−matching (I−b)
target therapy before−matching (C−b)
Endocrine, nutritional and metabolic diseases, and immunity disorders
Diseases of the nervous system and sense organs
autoimmune−free %
P < 0.0001
P = 0.0129
P = 0.0287
P = 3e−04
Lung cancer num. at risk
post−matching
C−p 2441.0 1855.5 1333.8 954.4 705.0 548.4
Pem−p 2441 1505 1009 655 420 285
before−matching
C−b 6022 4647 3487 2602 1958 1544
Pem−b 2919 1791 1195 765 505 342
C−p 2020.0 1513.8 1090.4 770.3 559.1 433.0
Niv−p 2020 1234 800 552 386 288
C−b 6022 4647 3487 2602 1958 1544
Niv−b 2435 1488 976 668 478 357
C−p 5201.0 3901.8 2821.3 2004.1 1476.8 1142.5
All−p 5201 3235 2122 1390 936 658
C−b 6022 4647 3487 2602 1958 1544
All−b 6175 3833 2519 1653 1135 800

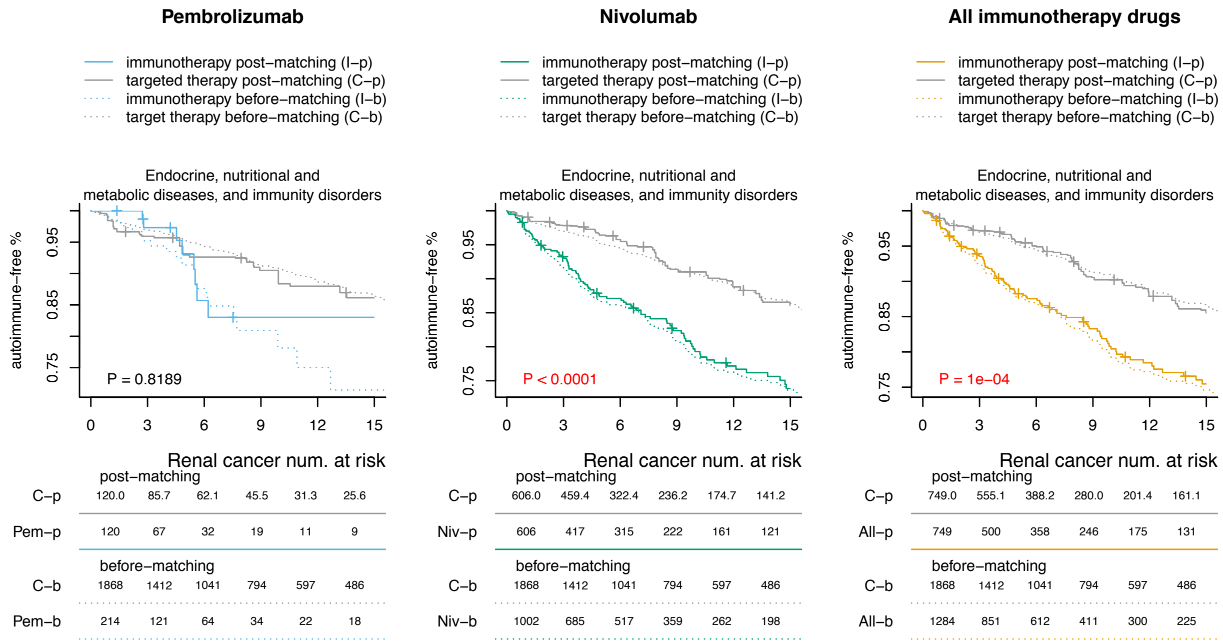
Pembrolizumab
Nivolumab
All immunotherapy drugs
immunotherapy post−matching (I−p)
targeted therapy post−matching (C−p)
immunotherapy before−matching (I−b)
target therapy before−matching (C−b)
Endocrine, nutritional and metabolic diseases, and immunity disorders
autoimmune−free %
P = 0.8189
P < 0.0001
P = 1e−04
Renal cancer num. at risk
post−matching
C−p 120.0 85.7 62.1 45.5 31.3 25.6
Pem−p 120 67 32 19 11 9
before−matching
C−b 1868 1412 1041 794 597 486
Pem−b 214 121 64 34 22 18
C−p 606.0 459.4 322.4 236.2 174.7 141.2
Niv−p 606 417 315 222 161 121
C−b 1868 1412 1041 794 597 486
Niv−b 1002 685 517 359 262 198
C−p 749.0 555.1 388.2 280.0 201.4 161.1
All−p 749 500 358 246 175 131
C−b 1868 1412 1041 794 597 486
All−b 1284 851 612 411 300 225

Figure S17. Results comparing the patients with only immunotherapy with patients who only have chemotherapy or only have targeted therapy.

Chemotherapy

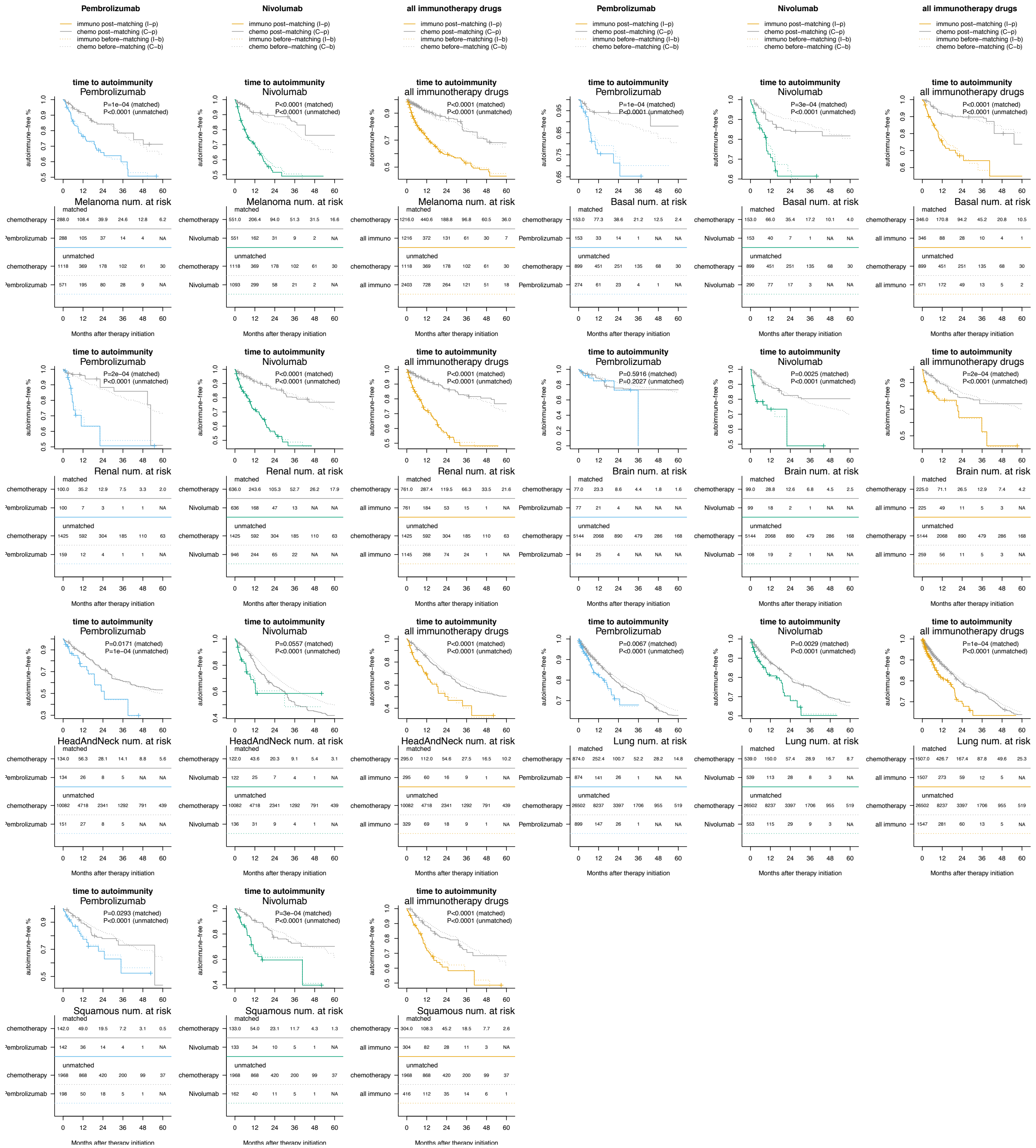

Targeted therapy

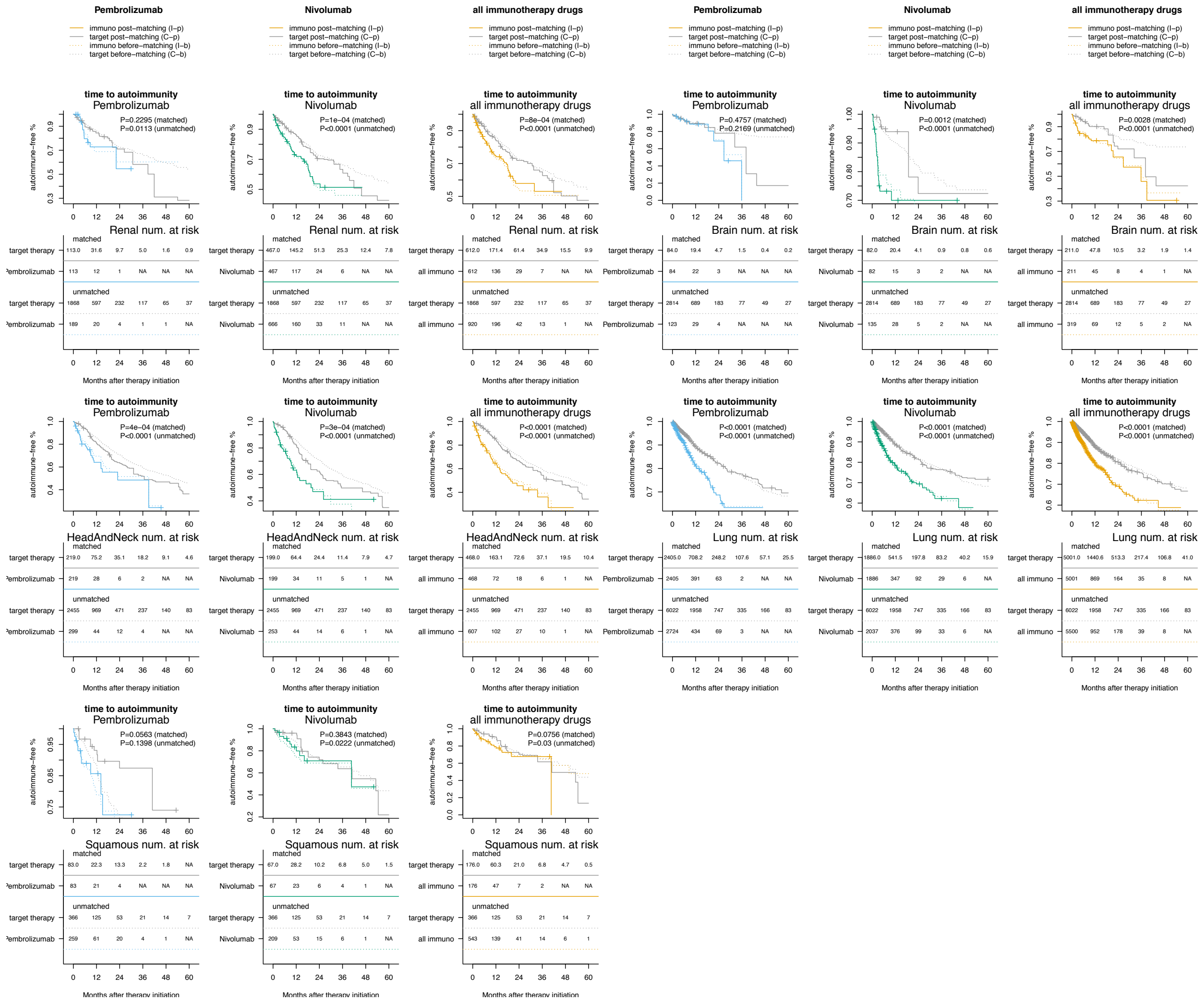

Figure S18. Kaplan-Meier curves where treatment group patients can have only one immunotherapy.

Chemotherapy

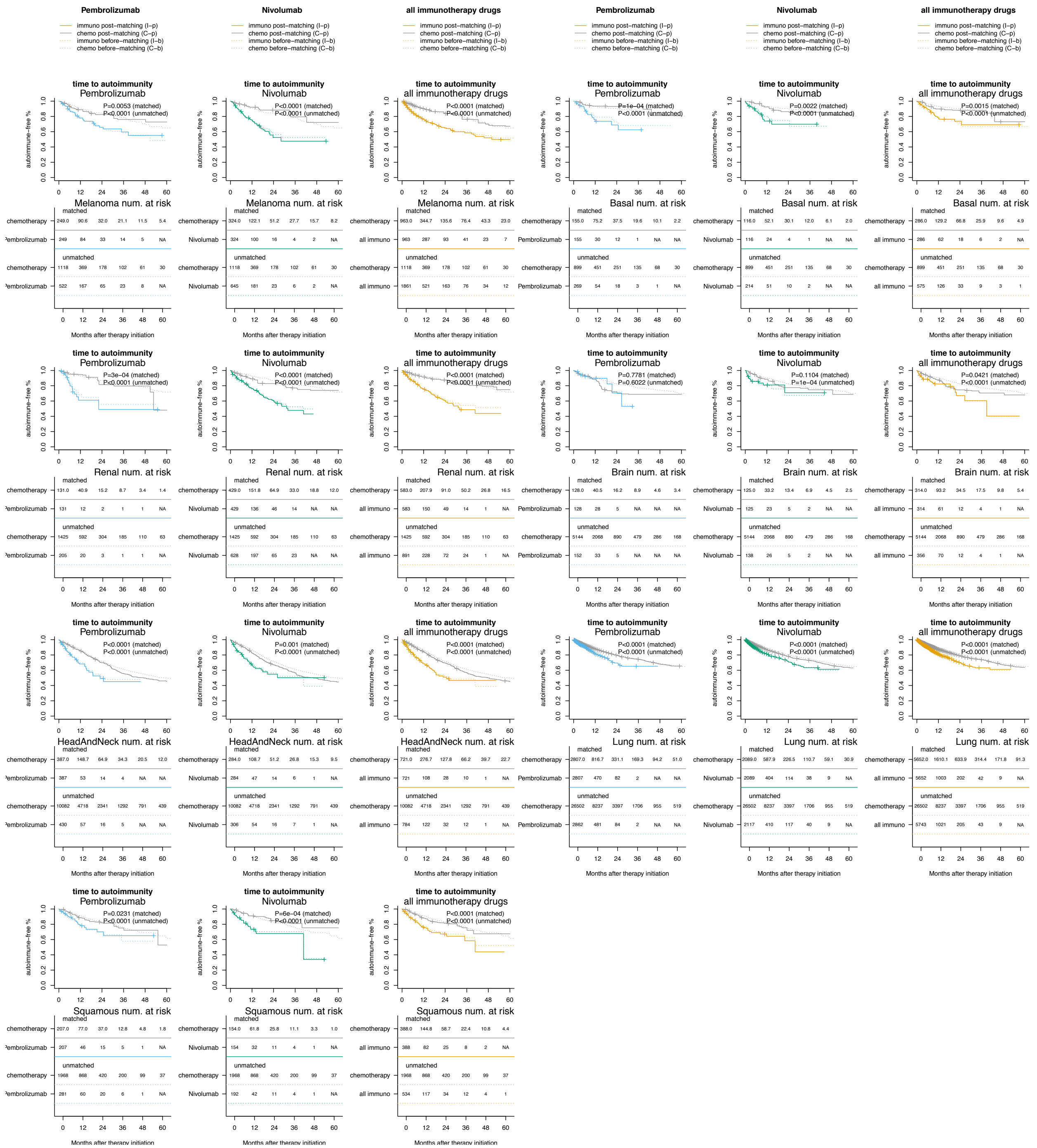

Targeted therapy

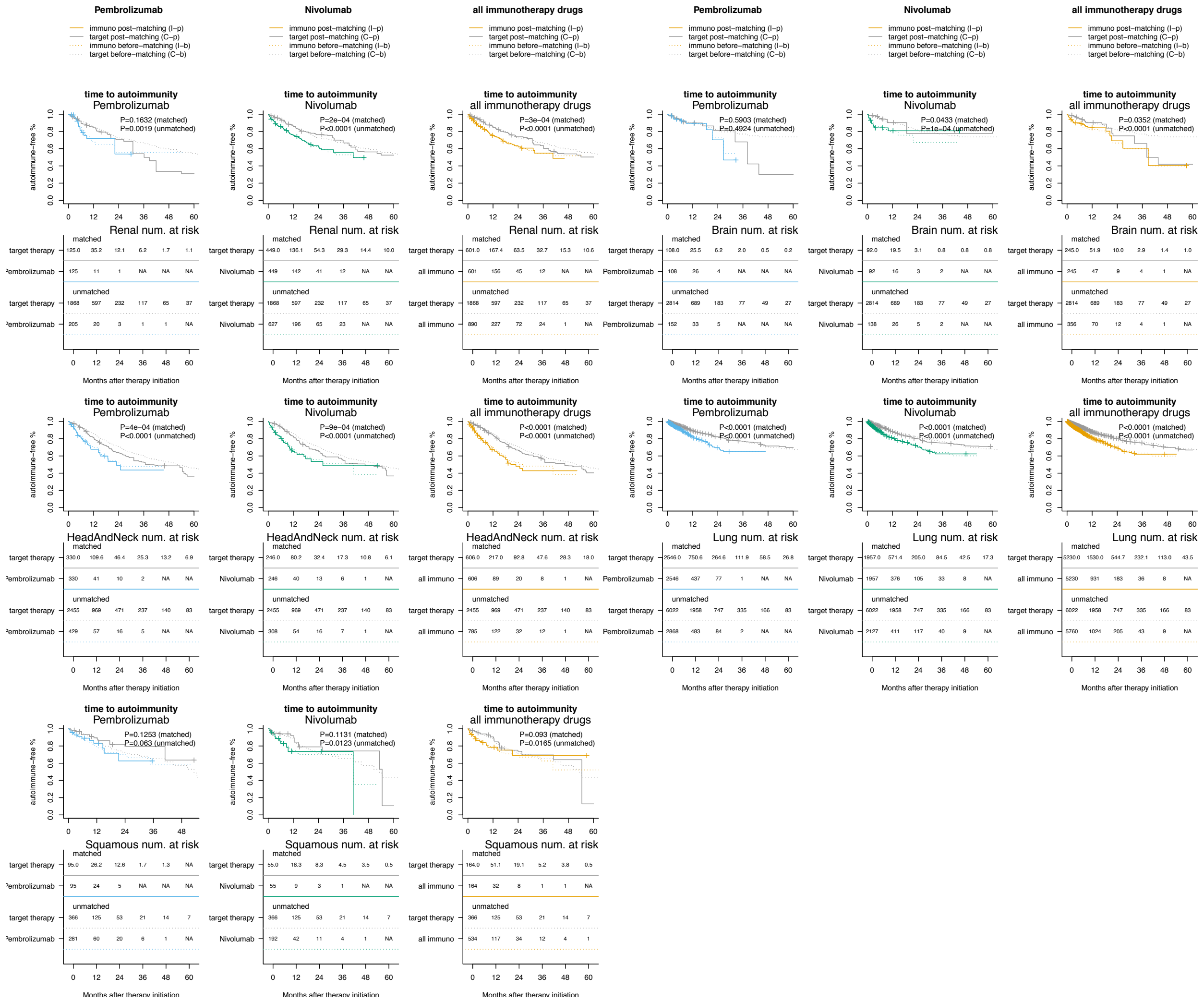

Table S1. Diagnosis codes included in this study.

Renal Cancer

IcdCode	PheWASCode	IcdString
189	189.11	Malignant neoplasm of kidney, except pelvis
C64	189.11	Malignant neoplasm of kidney, except renal pelvis
C64.1	189.11	Malignant neoplasm of right kidney, except renal pelvis
C64.2	189.11	Malignant neoplasm of left kidney, except renal pelvis
C64.9	189.11	Malignant neoplasm of unspecified kidney, except renal pelvis

Brain Cancer

IcdCode	PheWASCode	IcdString
191	191.11	Malignant neoplasm of brain
191	191.11	Malignant neoplasm of cerebrum, except lobes and ventricles
191.1	191.11	Malignant neoplasm of frontal lobe
191.2	191.11	Malignant neoplasm of temporal lobe
191.3	191.11	Malignant neoplasm of parietal lobe
191.4	191.11	Malignant neoplasm of occipital lobe
191.5	191.11	Malignant neoplasm of ventricles

191.6	191.11	Malignant neoplasm of cerebellum NOS
191.7	191.11	Malignant neoplasm of brain stem
191.8	191.11	Malignant neoplasm of other parts of brain
191.9	191.11	Malignant neoplasm of brain, unspecified
C71	191.11	Malignant neoplasm of brain
C71.0	191.11	Malignant neoplasm of supratentorial NOS
C71.1	191.11	Malignant neoplasm of frontal lobe
C71.2	191.11	Malignant neoplasm of temporal lobe
C71.3	191.11	Malignant neoplasm of parietal lobe
C71.4	191.11	Malignant neoplasm of occipital lobe
C71.5	191.11	Malignant neoplasm of cerebral ventricle
C71.6	191.11	Malignant neoplasm of cerebellum
C71.7	191.11	Malignant neoplasm of fourth cerebral ventricle
C71.8	191.11	Malignant neoplasm of overlapping sites of brain
C71.9	191.11	Malignant neoplasm of brain, unspecified

Squamous Cancer

IcdCode	PheWASCode	IcdString
173.02	172.22	Squamous cell carcinoma of skin of lip
173.12	172.22	Squamous cell carcinoma of eyelid, including canthus
173.22	172.22	Squamous cell carcinoma of skin of ear and external auditory canal
173.32	172.22	Squamous cell carcinoma of skin of other and unspecified parts of face
173.42	172.22	Squamous cell carcinoma of scalp and skin of neck
173.52	172.22	Squamous cell carcinoma of skin of trunk, except scrotum
173.62	172.22	Squamous cell carcinoma of skin of upper limb, including shoulder
173.72	172.22	Squamous cell carcinoma of skin of lower limb, including hip
173.82	172.22	Squamous cell carcinoma of other specified sites of skin
173.92	172.22	Squamous cell carcinoma of skin, site unspecified
C44.02	172.22	Squamous cell carcinoma of skin of lip
C44.121	172.22	Squamous cell carcinoma skin/ unsp eyelid, including canthus
C44.122	172.22	Squamous cell carcinoma skin/ right eyelid, inc canthus
C44.129	172.22	Squamous cell carcinoma skin/ left eyelid, including canthus
C44.221	172.22	Squamous cell carcinoma skin/ unsp ear and extrn auric canal
C44.222	172.22	Squamous cell carcinoma skin/ r ear and external auric canal

C44.229	172.22	Squamous cell carcinoma skin/ left ear and extrn auric canal
C44.320	172.22	Squamous cell carcinoma of skin of unspecified parts of face
C44.321	172.22	Squamous cell carcinoma of skin of nose
C44.329	172.22	Squamous cell carcinoma of skin of other parts of face
C44.42	172.22	Squamous cell carcinoma of skin of scalp and neck
C44.520	172.22	Squamous cell carcinoma of perianal skin
C44.521	172.22	Squamous cell carcinoma of skin of breast
C44.529	172.22	Squamous cell carcinoma of skin of other part of trunk
C44.621	172.22	Squamous cell carcinoma skin/ unsp upper limb, inc shoulder
C44.622	172.22	Squamous cell carcinoma skin/ right upper limb, inc shoulder
C44.629	172.22	Squamous cell carcinoma skin/ left upper limb, inc shoulder
C44.721	172.22	Squamous cell carcinoma skin/ unsp lower limb, including hip
C44.722	172.22	Squamous cell carcinoma skin/ right lower limb, inc hip
C44.729	172.22	Squamous cell carcinoma skin/ left lower limb, including hip
C44.82	172.22	Squamous cell carcinoma of overlapping sites of skin
C44.92	172.22	Squamous cell carcinoma of skin, unspecified

Basal Cancer

IcdCode	PheWASCode	IcdString
173.01	172.21	Basal cell carcinoma of skin of lip
173.11	172.21	Basal cell carcinoma of eyelid, including canthus
173.21	172.21	Basal cell carcinoma of skin of ear and external auditory canal
173.31	172.21	Basal cell carcinoma of skin of other and unspecified parts of face
173.41	172.21	Basal cell carcinoma of scalp and skin of neck
173.51	172.21	Basal cell carcinoma of skin of trunk, except scrotum
173.61	172.21	Basal cell carcinoma of skin of upper limb, including shoulder
173.71	172.21	Basal cell carcinoma of skin of lower limb, including hip
173.81	172.21	Basal cell carcinoma of other specified sites of skin
173.91	172.21	Basal cell carcinoma of skin, site unspecified
C44.01	172.21	Basal cell carcinoma of skin of lip
C44.111	172.21	Basal cell carcinoma skin/ unsp eyelid, including canthus
C44.112	172.21	Basal cell carcinoma skin/ right eyelid, including canthus
C44.119	172.21	Basal cell carcinoma skin/ left eyelid, including canthus
C44.211	172.21	Basal cell carcinoma skin/ unsp ear and external auric canal
C44.212	172.21	Basal cell carcinoma skin/ r ear and external auric canal

C44.219	172.21	Basal cell carcinoma skin/ left ear and external auric canal
C44.310	172.21	Basal cell carcinoma of skin of unspecified parts of face
C44.311	172.21	Basal cell carcinoma of skin of nose
C44.319	172.21	Basal cell carcinoma of skin of other parts of face
C44.41	172.21	Basal cell carcinoma of skin of scalp and neck
C44.510	172.21	Basal cell carcinoma of perianal skin
C44.511	172.21	Basal cell carcinoma of skin of breast
C44.519	172.21	Basal cell carcinoma of skin of other part of trunk
C44.611	172.21	Basal cell carcinoma skin/ unsp upper limb, inc shoulder
C44.612	172.21	Basal cell carcinoma skin/ right upper limb, inc shoulder
C44.619	172.21	Basal cell carcinoma skin/ left upper limb, inc shoulder
C44.711	172.21	Basal cell carcinoma skin/ unsp lower limb, including hip
C44.712	172.21	Basal cell carcinoma skin/ right lower limb, including hip
C44.719	172.21	Basal cell carcinoma skin/ left lower limb, including hip
C44.81	172.21	Basal cell carcinoma of overlapping sites of skin
C44.91	172.21	Basal cell carcinoma of skin, unspecified

HeadAndNeck Cancer

IcdCode	PheWASCode	IcdString
140	145.1	Malignant neoplasm of lip
140.0	145.1	Malignant neoplasm of upper lip, vermilion border
140.1	145.1	Malignant neoplasm of lower lip, vermilion border
140.3	145.1	Malignant neoplasm of upper lip, inner aspect
140.4	145.1	Malignant neoplasm of lower lip, inner aspect
140.5	145.1	Malignant neoplasm of lip, unspecified, inner aspect
140.6	145.1	Malignant neoplasm of commissure of lip
140.8	145.1	Malignant neoplasm of other sites of lip
140.9	145.1	Malignant neoplasm of lip, unspecified, vermilion border
141	145.2	Malignant neoplasm of tongue
141	145.2	Malignant neoplasm of base of tongue
141.1	145.2	Malignant neoplasm of dorsal surface of tongue
141.2	145.2	Malignant neoplasm of tip and lateral border of tongue
141.3	145.2	Malignant neoplasm of ventral surface of tongue
141.4	145.2	Malignant neoplasm of anterior two-thirds of tongue, part unspecified
141.5	145.2	Malignant neoplasm of junctional zone of tongue

141.6	145.2	Malignant neoplasm of lingual tonsil
141.8	145.2	Malignant neoplasm of other sites of tongue
141.9	145.2	Malignant neoplasm of tongue, unspecified
142	145.3	Malignant neoplasm of major salivary glands
142	145.3	Malignant neoplasm of parotid gland
142.1	145.3	Malignant neoplasm of submandibular gland
142.2	145.3	Malignant neoplasm of sublingual gland
142.8	145.3	Malignant neoplasm of other major salivary glands
142.9	145.3	Malignant neoplasm of salivary gland, unspecified
143	145.4	Malignant neoplasm of gum
143	145.4	Malignant neoplasm of upper gum
143.1	145.4	Malignant neoplasm of lower gum
143.8	145.4	Malignant neoplasm of other sites of gum
143.9	145.4	Malignant neoplasm of gum, unspecified
144	145.5	Malignant neoplasm of floor of mouth
144	145.5	Malignant neoplasm of anterior portion of floor of mouth
144.1	145.5	Malignant neoplasm of lateral portion of floor of mouth
144.8	145.5	Malignant neoplasm of other sites of floor of mouth

144.9	145.5	Malignant neoplasm of floor of mouth, part unspecified
145	145	Malignant neoplasm of other and unspecified parts of mouth
145	145	Malignant neoplasm of cheek mucosa
145.1	145	Malignant neoplasm of vestibule of mouth
145.2	145	Malignant neoplasm of hard palate
145.3	145	Malignant neoplasm of soft palate
145.4	145	Malignant neoplasm of uvula
145.5	145	Malignant neoplasm of palate, unspecified
145.6	145	Malignant neoplasm of retromolar area
145.8	145	Malignant neoplasm of other specified parts of mouth
145.9	145	Malignant neoplasm of mouth, unspecified
146	149.1	Malignant neoplasm of oropharynx
146	149.1	Malignant neoplasm of tonsil
146.1	149.1	Malignant neoplasm of tonsillar fossa
146.2	149.1	Malignant neoplasm of tonsillar pillars (anterior) (posterior)
146.3	149.1	Malignant neoplasm of vallecula
146.4	149.1	Malignant neoplasm of anterior aspect of epiglottis
146.5	149.1	Malignant neoplasm of junctional region of oropharynx

146.6	149.1	Malignant neoplasm of lateral wall of oropharynx
146.7	149.1	Malignant neoplasm of posterior wall of oropharynx
146.8	149.1	Malignant neoplasm of other specified sites of oropharynx
146.9	149.1	Malignant neoplasm of oropharynx, unspecified
147	149.2	Malignant neoplasm of nasopharynx
147	149.2	Malignant neoplasm of superior wall of nasopharynx
147.1	149.2	Malignant neoplasm of posterior wall of nasopharynx
147.2	149.2	Malignant neoplasm of lateral wall of nasopharynx
147.3	149.2	Malignant neoplasm of anterior wall of nasopharynx
147.8	149.2	Malignant neoplasm of other specified sites of nasopharynx
147.9	149.2	Malignant neoplasm of nasopharynx, unspecified
148	149.3	Malignant neoplasm of hypopharynx
148	149.3	Malignant neoplasm of postcricoid region of hypopharynx
148.1	149.3	Malignant neoplasm of pyriform sinus
148.2	149.3	Malignant neoplasm of aryepiglottic fold, hypopharyngeal aspect
148.3	149.3	Malignant neoplasm of posterior hypopharyngeal wall
148.8	149.3	Malignant neoplasm of other specified sites of hypopharynx
148.9	149.3	Malignant neoplasm of hypopharynx, unspecified

149	149	Malignant neoplasm of other and ill-defined sites within the lip, oral cavity, and pharynx
149	149	Malignant neoplasm of pharynx, unspecified
149.1	149	Malignant neoplasm of waldeyer's ring
149.8	149	Malignant neoplasm of other sites within the lip and oral cavity
149.9	149	Malignant neoplasm of ill-defined sites within the lip and oral cavity
160	149.9	Malignant neoplasm of nasal cavities, middle ear, and accessory sinuses
160	149.9	Malignant neoplasm of nasal cavities
160.1	149.9	Malignant neoplasm of auditory tube, middle ear, and mastoid air cells
160.2	149.9	Malignant neoplasm of maxillary sinus
160.3	149.9	Malignant neoplasm of ethmoidal sinus
160.4	149.9	Malignant neoplasm of frontal sinus
160.5	149.9	Malignant neoplasm of sphenoidal sinus
160.8	149.9	Malignant neoplasm of other accessory sinuses
160.9	149.9	Malignant neoplasm of accessory sinus, unspecified
161	149.4	Malignant neoplasm of larynx
C06.2	145	Malignant neoplasm of retromolar area
C05.9	145	Malignant neoplasm of roof of mouth
C08	145.3	malignant neoplasm of salivary ducts

C08.9	145.3	Malignant neoplasm of salivary gland (major) NOS
C05.1	145	Malignant neoplasm of soft palate
C31.3	149.9	Malignant neoplasm of sphenoid sinus
C32.2	149.4	Malignant neoplasm of subglottis
C08.1	145.3	Malignant neoplasm of sublingual gland
C08.0	145.3	Malignant neoplasm of submaxillary gland
C11.0	149.2	Malignant neoplasm of superior wall of nasopharynx
C02.1	145.2	Malignant neoplasm of tip of tongue
C02.9	145.2	Malignant neoplasm of tongue, unspecified
C09	149.1	Malignant neoplasm of tonsil
C09.9	149.1	Malignant neoplasm of tonsil, unspecified
C09.0	149.1	Malignant neoplasm of tonsillar fossa
C09.1	149.1	Malignant neoplasm of tonsillar pillar (anterior) (posterior)
C02.8	145.2	Malignant neoplasm of two or more contiguous sites of tongue
C30.1	149.9	Malignant neoplasm of tympanic cavity
C03.0	145.4	Malignant neoplasm of upper gum
C00.3	145.1	Malignant neoplasm of upper lip, inner aspect
C05.2	145	Malignant neoplasm of uvula

C10.0	149.1	Malignant neoplasm of vallecula
C02.2	145.2	Malignant neoplasm of ventral surface of tongue
C32.1	149.4	Malignant neoplasm of ventricular bands
C00.2	145.1	Malignant neoplasm of vermilion border of lip NOS
C00.1	145.1	Malignant neoplasm of vermilion border of lower lip
C00.0	145.1	Malignant neoplasm of vermilion border of upper lip
C06.1	145	Malignant neoplasm of vestibule of mouth
C30.0	149.9	Malignant neoplasm of vestibule of nose
C32.0	149.4	Malignant neoplasm of vocal cord (true) NOS
C14.2	149	Malignant neoplasm of Waldeyer's ring
Z85.03	145.2	Personal history of malignant neoplasm of large intestine
Z85.21	149.4	Personal history of malignant neoplasm of larynx
Z85.02	145.2	Personal history of malignant neoplasm of stomach
Z85.810	145.2	Personal history of malignant neoplasm of tongue
C14.8	149	Primary malignant neoplasm of two or more contiguous sites of lip, oral cavity and pharynx
Z85.22	149.9	Prsnl hx of malig neoplm of nasl cav, mid ear, & acces sinus
Z85.818	149.5	Prsnl hx of malig neoplm of site of lip, oral cav, & pharynx

Z85.819	149.5	Prsnl hx of malig neoplm of unsp site lip,oral cav,& pharynx
161	149.4	Malignant neoplasm of glottis
161.1	149.4	Malignant neoplasm of supraglottis
161.2	149.4	Malignant neoplasm of subglottis
161.3	149.4	Malignant neoplasm of laryngeal cartilages
161.8	149.4	Malignant neoplasm of other specified sites of larynx
161.9	149.4	Malignant neoplasm of larynx, unspecified
195	149.4	Malignant neoplasm of head, face, and neck
230	145	Carcinoma in situ of lip, oral cavity, and pharynx
231	149.4	Carcinoma in situ of larynx
V10.01	145.2	Personal history of malignant neoplasm of tongue
V10.02	149.5	Personal history of malignant neoplasm of other and unspecified parts of oral cavity and pharynx
V10.21	149.4	Personal history of malignant neoplasm of larynx
V10.22	149.9	Personal history of malignant neoplasm of nasal cavities, middle ear, and accessory sinuses
D00.02	145	Carcinoma in situ of buccal mucosa
D00.06	145	Carcinoma in situ of floor of mouth
D00.03	145	Carcinoma in situ of gingiva and edentulous alveolar ridge

D00.05	145	Carcinoma in situ of hard palate
D00.01	145	Carcinoma in situ of labial mucosa and vermilion border
D02.0	149.4	Carcinoma in situ of larynx
D00.0	145	Carcinoma in situ of lip, oral cavity and pharynx
D00.00	145	Carcinoma in situ of oral cavity, unspecified site
D00.08	145	Carcinoma in situ of pharynx
D00.04	145	Carcinoma in situ of soft palate
D00.07	145	Carcinoma in situ of tongue
C31.9	149.9	Malignant neoplasm of accessory sinus, unspecified
C31	149.9	Malignant neoplasm of accessory sinuses
C04.0	145.5	Malignant neoplasm of anterior to the premolar-canine junction
C10.4	149.1	Malignant neoplasm of branchial cyst [site of neoplasm]
C00.6	145.1	Malignant neoplasm of commissure of lip, unspecified
C02.0	145.2	Malignant neoplasm of dorsal surface of tongue
C31.1	149.9	Malignant neoplasm of ethmoidal sinus
C04	145.5	Malignant neoplasm of floor of mouth
C04.9	145.5	Malignant neoplasm of floor of mouth, unspecified

C31.2	149.9	Malignant neoplasm of frontal sinus
C03	145.4	malignant neoplasm of gingiva
C10.1	149.1	Malignant neoplasm of glossoepiglottic fold(s)
C03.9	145.4	Malignant neoplasm of gum, unspecified
C05.0	145	Malignant neoplasm of hard palate
C13	149.3	Malignant neoplasm of hypopharynx
C13.9	149.3	Malignant neoplasm of hypopharynx, unspecified
C13.1	149.3	Malignant neoplasm of interarytenoid fold, marginal zone
C06.0	145	Malignant neoplasm of internal cheek
C32.3	149.4	Malignant neoplasm of laryngeal cartilage
C32	149.4	Malignant neoplasm of larynx
C32.9	149.4	Malignant neoplasm of larynx, unspecified
C04.1	145.5	Malignant neoplasm of lateral floor of mouth
C10.2	149.1	Malignant neoplasm of lateral wall of oropharynx
C02.4	145.2	Malignant neoplasm of lingual tonsil
C00	145.1	Malignant neoplasm of lip
C00.9	145.1	Malignant neoplasm of lip, unspecified
C03.1	145.4	Malignant neoplasm of lower gum

C31.0	149.9	Malignant neoplasm of maxillary sinus
C02.3	145.2	Malignant neoplasm of mobile part of tongue NOS
C11	149.2	Malignant neoplasm of nasopharynx
C11.9	149.2	Malignant neoplasm of nasopharynx, unspecified
C76.0	195.3	Malignant neoplasm of nose NOS
C00.5	145.1	Malignant neoplasm of oral aspect of lip, unspecified
C00.4	145.1	Malignant neoplasm of oral aspect of lower lip
C06.9	145	Malignant neoplasm of oral cavity NOS
C10	149.1	Malignant neoplasm of oropharynx
C10.9	149.1	Malignant neoplasm of oropharynx, unspecified
C14	149	Malignant neoplasm of other and ill-defined sites in the lip, oral cavity and pharynx
C06	145	Malignant neoplasm of other and unspecified parts of mouth
C02	145.2	Malignant neoplasm of other and unspecified parts of tongue
C31.8	149.9	Malignant neoplasm of overlapping sites of accessory sinuses
C04.8	145.5	Malignant neoplasm of overlapping sites of floor of mouth
C13.8	149.3	Malignant neoplasm of overlapping sites of hypopharynx
C32.8	149.4	Malignant neoplasm of overlapping sites of larynx
C00.8	145.1	Malignant neoplasm of overlapping sites of lip

C11.8	149.2	Malignant neoplasm of overlapping sites of nasopharynx
C10.8	149.1	Malignant neoplasm of overlapping sites of oropharynx
C06.8	145	Malignant neoplasm of overlapping sites of other and unspecified parts of mouth
C06.89	145	Malignant neoplasm of overlapping sites of other parts of mouth
C05.8	145	Malignant neoplasm of overlapping sites of palate
C09.8	149.1	Malignant neoplasm of overlapping sites of tonsil
C06.80	145	Malignant neoplasm of ovrlp sites of unsp parts of mouth
C05	145	Malignant neoplasm of palate
C07	145.3	Malignant neoplasm of parotid gland
C11.2	149.2	Malignant neoplasm of pharyngeal recess
C14.0	149	Malignant neoplasm of pharynx, unspecified
C13.0	149.3	Malignant neoplasm of postcricoid region
C11.3	149.2	Malignant neoplasm of posterior margin of nasal septum
C01	145.2	Malignant neoplasm of posterior third of tongue
C13.2	149.3	Malignant neoplasm of posterior wall of hypopharynx
C11.1	149.2	Malignant neoplasm of posterior wall of nasopharynx
C10.3	149.1	Malignant neoplasm of posterior wall of oropharynx

C12	149.3	Malignant neoplasm of pyriform sinus

Melanoma Cancer

IcdCode	PheWASCode	IcdString
172	172.11	Malignant melanoma of skin
172.0	172.11	Malignant melanoma of skin of lip
172.1	172.11	Malignant melanoma of skin of eyelid, including canthus
172.2	172.11	Malignant melanoma of skin of ear and external auditory canal
172.3	172.11	Malignant melanoma of skin of other and unspecified parts of face
172.4	172.11	Malignant melanoma of skin of scalp and neck
172.5	172.11	Malignant melanoma of skin of trunk, except scrotum
172.6	172.11	Malignant melanoma of skin of upper limb, including shoulder
172.7	172.11	Malignant melanoma of skin of lower limb, including hip
172.8	172.11	Malignant melanoma of other specified sites of skin
172.9	172.11	Melanoma of skin, site unspecified
C43	172.11	Malignant melanoma of skin
C43.0	172.11	Malignant melanoma of lip

C43.1	172.11	Malignant melanoma of eyelid, including canthus
C43.10	172.11	Malignant melanoma of unspecified eyelid, including canthus
C43.11	172.11	Malignant melanoma of right eyelid, including canthus
C43.12	172.11	Malignant melanoma of left eyelid, including canthus
C43.2	172.11	Malignant melanoma of ear and external auricular canal
C43.20	172.11	Malignant melanoma of unspecified ear and external auricular canal
C43.21	172.11	Malignant melanoma of right ear and external auricular canal
C43.22	172.11	Malignant melanoma of left ear and external auricular canal
C43.3	172.11	Malignant melanoma of other and unspecified parts of face
C43.30	172.11	Malignant melanoma of unspecified part of face
C43.31	172.11	Malignant melanoma of nose
C43.39	172.11	Malignant melanoma of other parts of face
C43.4	172.11	Malignant melanoma of scalp and neck
C43.5	172.11	Malignant melanoma of trunk
C43.51	172.11	Malignant melanoma of perianal skin
C43.52	172.11	Malignant melanoma of skin of breast
C43.59	172.11	Malignant melanoma of other part of trunk
C43.6	172.11	Malignant melanoma of upper limb, including shoulder

C43.60	172.11	Malignant melanoma of unspecified upper limb, including shoulder
C43.61	172.11	Malignant melanoma of right upper limb, including shoulder
C43.62	172.11	Malignant melanoma of left upper limb, including shoulder
C43.7	172.11	Malignant melanoma of lower limb, including hip
C43.70	172.11	Malignant melanoma of unspecified lower limb, including hip
C43.71	172.11	Malignant melanoma of right lower limb, including hip
C43.72	172.11	Malignant melanoma of left lower limb, including hip
C43.8	172.11	Malignant melanoma of overlapping sites of skin
C43.9	172.11	Melanoma (malignant) NOS
D03	172.11	Melanoma in situ
D03.0	172.11	Melanoma in situ of lip
D03.1	172.11	Melanoma in situ of eyelid, including canthus
D03.10	172.11	Melanoma in situ of unspecified eyelid, including canthus
D03.11	172.11	Melanoma in situ of right eyelid, including canthus
D03.12	172.11	Melanoma in situ of left eyelid, including canthus
D03.2	172.11	Melanoma in situ of ear and external auricular canal
D03.20	172.11	Melanoma in situ of unspecified ear and external auricular canal
D03.21	172.11	Melanoma in situ of right ear and external auricular canal

D03.22	172.11	Melanoma in situ of left ear and external auricular canal
D03.3	172.11	Melanoma in situ of other and unspecified parts of face
D03.30	172.11	Melanoma in situ of unspecified part of face
D03.39	172.11	Melanoma in situ of other parts of face
D03.4	172.11	Melanoma in situ of scalp and neck
D03.5	172.11	Melanoma in situ of trunk
D03.51	172.11	Melanoma in situ of perianal skin
D03.52	172.11	Melanoma in situ of breast (skin) (soft tissue)
D03.59	172.11	Melanoma in situ of other part of trunk
D03.6	172.11	Melanoma in situ of upper limb, including shoulder
D03.60	172.11	Melanoma in situ of unspecified upper limb, including shoulder
D03.61	172.11	Melanoma in situ of right upper limb, including shoulder
D03.62	172.11	Melanoma in situ of left upper limb, including shoulder
D03.7	172.11	Melanoma in situ of lower limb, including hip
D03.70	172.11	Melanoma in situ of unspecified lower limb, including hip
D03.71	172.11	Melanoma in situ of right lower limb, including hip
D03.72	172.11	Melanoma in situ of left lower limb, including hip
D03.8	172.11	Melanoma in situ of scrotum

D03.9	172.11	Melanoma in situ, unspecified

Table S2. Immune checkpoint inhibitors included in this study.

name	checkpoint	HCPCS.code	NDC.code
Nivolumab.Opdivo	PD-1	C9453,J9299	00003373413<br>00003377211<br>00003377412
Pembrolizumab.Keytruda	PD-1	C9027,J9271	00006302602<br>00006302902
Ipilimumab.Yervoy	CTLA-4	C9284,J9228	00003232711<br>00003232822
Atezolizumab.Tecentriq	PD-L1	C9483,J9022	05024291701<br>05024291786
Avelumab.Bavencio	PD-L1	C9491,J9023	04408735351
Durvalumab.Imfinzi	PD-L1	C9492	00310450012<br>00310461150

Table S3. Chemotherapy CPT/HCPCS procedure codes. Codes for renal cancer are shown as examples.

PX2	CHEMOCAT	CODE_TYPE	DESCRIPTION
J9045	CARBOPLATIN	HCPC	Carboplatin, 50 mg
J9062	CISPLATIN	HCPC	Cisplatin, 50 mg
C9418	CISPLATIN	HCPC	CISPLATIN, POWDER OR S0LUTION, BRAND NAME, PER 10 MG
J9060	CISPLATIN	HCPC	Cisplatin, powder or solution, per 10 mg
209622	CISPLATIN	HCPC	REIMB CISPLATIN 10 MG VIAL (PLATINOL).
J9000	DOXORUBICIN	HCPC	Doxorubicin HCl, 10 mg A
J9001	DOXORUBICIN	HCPC	Doxorubicin HCl, all lipid formulations, 10 mg A
C9415	DOXORUBICIN	HCPC	DOXORUBICIN HCL, BRAND NAME, 10 MG
J9002	DOXORUBICIN	HCPC	DOXORUBICIN HCL, LIPOSOMAL, 10 MG IV
Q2049	DOXORUBICIN	HCPC	DOXORUBICIN HCL, LIPOSOMAL, 10 MG IV
Q2048	DOXORUBICIN	HCPC	INJ DOXORUBICIN HYDROCHLORIDE LIP DOXIL 10 MG
Q2050	DOXORUBICIN	HCPC	INJECTION DOXORUBICIN HCL LIPOSOMAL NOS 10 MG
J9190	FLUOROURACIL	HCPC	Fluorouracil, 500 mg
J9201	GEMCITABINE	HCPC	Gemcitabine HCl, 200 mg
J9264	PACLITAXEL	HCPC	Injection, paclitaxel protein-bound particles, 1 mg

J9267	PACLITAXEL	HCPC	INJECTION, PACLITAXEL, 1 MG
J9265	PACLITAXEL	HCPC	Paclitaxel, 30 mg
C9431	PACLITAXEL	HCPC	PACLITAXEL, BRAND NAME, 30 MG

Table S4. Chemotherapy National Drug Codes.

National Drug Code	CHEMOCAT	GENERIC_NAME	BRAND_NAME	DATE_ ADDED
16714046701	CAPECITABINE	CAPECITABINE	CAPECITABINE	3/31/2016
16714046801	CAPECITABINE	CAPECITABINE	CAPECITABINE	3/31/2016
00179019570	CAPECITABINE	CAPECITABINE	CAPECITABINE 500 MG TABLET (UD)	3/31/2016
00093747306	CAPECITABINE	CAPECITABINE	CAPECITABINE TAB 150 MG	6/16/2014
00093747489	CAPECITABINE	CAPECITABINE	CAPECITABINE TAB 500 MG	6/16/2014
54569571700	CAPECITABINE	CAPECITABINE	XELODA	4/25/2011
54868414300	CAPECITABINE	CAPECITABINE	XELODA	4/25/2011
54868414301	CAPECITABINE	CAPECITABINE	XELODA	4/25/2011
54868414302	CAPECITABINE	CAPECITABINE	XELODA	4/25/2011
54868526000	CAPECITABINE	CAPECITABINE	XELODA	4/25/2011
54868526001	CAPECITABINE	CAPECITABINE	XELODA	4/25/2011
54868526002	CAPECITABINE	CAPECITABINE	XELODA	4/25/2011
54868526003	CAPECITABINE	CAPECITABINE	XELODA	4/25/2011
54868526004	CAPECITABINE	CAPECITABINE	XELODA	4/25/2011

54868526005	CAPECITABINE	CAPECITABINE	XELODA	4/25/2011
54868526006	CAPECITABINE	CAPECITABINE	XELODA	4/25/2011
54868526007	CAPECITABINE	CAPECITABINE	XELODA	4/25/2011
54868526008	CAPECITABINE	CAPECITABINE	XELODA	4/25/2011
54868526009	CAPECITABINE	CAPECITABINE	XELODA	4/25/2011
68258903601	CAPECITABINE	CAPECITABINE	XELODA	4/25/2011
00004110020	CAPECITABINE	CAPECITABINE	XELODA TAB 150MG	4/25/2011
00004110051	CAPECITABINE	CAPECITABINE	XELODA TAB 150MG	4/25/2011
00004110116	CAPECITABINE	CAPECITABINE	XELODA TAB 500MG	4/25/2011
00004110150	CAPECITABINE	CAPECITABINE	XELODA TAB 500MG	4/25/2011
00004110175	CAPECITABINE	CAPECITABINE	XELODA TAB 500MG	5/31/2012
16729007212	CAPECITABINE	CAPECITABINE 150 MG TABLET	CAPECITABINE 150 MG ORAL TAB	7/17/2015
64980027606	CAPECITABINE	CAPECITABINE 150 MG TABLET	CAPECITABINE 150 MG ORAL TAB	4/10/2017
65162084306	CAPECITABINE	CAPECITABINE 150 MG TABLET	CAPECITABINE 150 MG ORAL TAB	4/10/2017
01671446701	CAPECITABINE	CAPECITABINE 150 MG TABLET	CAPECITABINE 150 MG TABLET	3/31/2016
01672907212	CAPECITABINE	CAPECITABINE 150 MG TABLET	CAPECITABINE 150 MG TABLET	3/31/2016

04229119060	CAPECITABINE	CAPECITABINE 150 MG TABLET	CAPECITABINE 150 MG TABLET	3/31/2016
00179014970	CAPECITABINE	CAPECITABINE 500 MG TABLET	CAPECITABINE 500 MG ORAL TAB	7/17/2015
00179022970	CAPECITABINE	CAPECITABINE 500 MG TABLET	CAPECITABINE 500 MG ORAL TAB	3/12/2018
16729007329	CAPECITABINE	CAPECITABINE 500 MG TABLET	CAPECITABINE 500 MG ORAL TAB	7/17/2015
64980027712	CAPECITABINE	CAPECITABINE 500 MG TABLET	CAPECITABINE 500 MG ORAL TAB	4/10/2017
65162084416	CAPECITABINE	CAPECITABINE 500 MG TABLET	CAPECITABINE 500 MG ORAL TAB	4/10/2017
01671446801	CAPECITABINE	CAPECITABINE 500 MG TABLET	CAPECITABINE 500 MG TABLET	3/31/2016
01672907329	CAPECITABINE	CAPECITABINE 500 MG TABLET	CAPECITABINE 500 MG TABLET	3/31/2016
04229119112	CAPECITABINE	CAPECITABINE 500 MG TABLET	CAPECITABINE 500 MG TABLET	3/31/2016
05107951001	CAPECITABINE	CAPECITABINE 500 MG TABLET	CAPECITABINE 500 MG TABLET	3/31/2016
05107951005	CAPECITABINE	CAPECITABINE 500 MG TABLET	CAPECITABINE 500 MG TABLET	3/31/2016
00179156770	CAPECITABINE	CAPECITABINE 500 MG TABLET	XELODA 500 MG ORAL TAB	7/17/2015
05486852600	CAPECITABINE	CAPECITABINE 500 MG TABLET	XELODA 500 MG TABLET	3/31/2016
05486852601	CAPECITABINE	CAPECITABINE 500 MG TABLET	XELODA 500 MG TABLET	3/31/2016
05486852602	CAPECITABINE	CAPECITABINE 500 MG TABLET	XELODA 500 MG TABLET	3/31/2016
05486852603	CAPECITABINE	CAPECITABINE 500 MG TABLET	XELODA 500 MG TABLET	3/31/2016

05486852605	CAPECITABINE	CAPECITABINE 500 MG TABLET	XELODA 500 MG TABLET	3/31/2016
05486852609	CAPECITABINE	CAPECITABINE 500 MG TABLET	XELODA 500 MG TABLET	3/31/2016
00054027121	CAPECITABINE	Capecitabine Tab 150 MG	#N/A	2/1/2017
00378251191	CAPECITABINE	Capecitabine Tab 150 MG	#N/A	7/17/2015
42291019060	CAPECITABINE	Capecitabine Tab 150 MG	#N/A	7/17/2015
42291016712	CAPECITABINE	CAPECITABINE TAB 500 MG	CAPECITABINE	3/12/2018
00054027223	CAPECITABINE	Capecitabine Tab 500 MG	#N/A	2/1/2017
00378251278	CAPECITABINE	Capecitabine Tab 500 MG	#N/A	7/17/2015
42291019112	CAPECITABINE	Capecitabine Tab 500 MG	#N/A	7/17/2015
51079051001	CAPECITABINE	Capecitabine Tab 500 MG	#N/A	7/17/2015
51079051005	CAPECITABINE	Capecitabine Tab 500 MG	#N/A	7/17/2015
60687014911	CAPECITABINE	Capecitabine Tab 500 MG	#N/A	2/1/2017
60687014994	CAPECITABINE	Capecitabine Tab 500 MG	#N/A	2/1/2017
54868414303	CAPECITABINE	XELODA 150 MG	XELODA	4/25/2011
49452317504	FLUOROURACIL	5-FLUOROURACIL (U.S.P.)	5-FLUOROURACIL	4/25/2011
44778007701	FLUOROURACIL	5-FLUOROURACIL 10 MG/0.2 ML INTRAVITREAL INJ	5-FLUOROURACIL 10 MG/0.2 ML INTRAVITREAL INJ	7/17/2015

00703301811	FLUOROURACIL	ADRUCIL	Fluorouracil Inj 2.5 GM/50ML (50 MG/ML)	6/16/2014
00703301911	FLUOROURACIL	ADRUCIL	Fluorouracil Inj 5 GM/100ML (50 MG/ML)	6/16/2014
00703301511	FLUOROURACIL	ADRUCIL	Fluorouracil Inj 500 MG/10ML (50 MG/ML)	6/16/2014
00004190406	FLUOROURACIL	FLUOROURACIL	[Generic]	3/31/2016
49452317502	FLUOROURACIL	FLUOROURACIL	5-FLUOROURACIL	4/25/2011
49452317503	FLUOROURACIL	FLUOROURACIL	5-FLUOROURACIL	4/25/2011
62991148602	FLUOROURACIL	FLUOROURACIL	5-FLUOROURACIL	4/25/2011
63370009515	FLUOROURACIL	FLUOROURACIL	5-FLUOROURACIL	4/25/2011
63370009525	FLUOROURACIL	FLUOROURACIL	5-FLUOROURACIL	4/25/2011
63370009535	FLUOROURACIL	FLUOROURACIL	5-FLUOROURACIL	4/25/2011
49452317501	FLUOROURACIL	FLUOROURACIL	5-FLUOROURACIL POW	4/25/2011
00013102691	FLUOROURACIL	FLUOROURACIL	ADRUCIL	3/31/2016
00013103601	FLUOROURACIL	FLUOROURACIL	ADRUCIL	3/31/2016
00013104601	FLUOROURACIL	FLUOROURACIL	ADRUCIL	3/31/2016
00013105602	FLUOROURACIL	FLUOROURACIL	ADRUCIL	3/31/2016
00013105691	FLUOROURACIL	FLUOROURACIL	ADRUCIL	3/31/2016

00703301513	FLUOROURACIL	FLUOROURACIL	ADRUCIL	4/25/2011
00703301812	FLUOROURACIL	FLUOROURACIL	ADRUCIL	4/25/2011
00703301912	FLUOROURACIL	FLUOROURACIL	ADRUCIL	4/25/2011
00013105694	FLUOROURACIL	FLUOROURACIL	ADRUCIL INJ 50MG/ML	4/25/2011
54868545000	FLUOROURACIL	FLUOROURACIL	CARAC	4/10/2017
00187320302	FLUOROURACIL	FLUOROURACIL	EFUDEX 5% SOLUTION	3/12/2018
00304217422	FLUOROURACIL	FLUOROURACIL	FLUOROURACIL	3/31/2016
10139006301	FLUOROURACIL	FLUOROURACIL	FLUOROURACIL	4/25/2011
10139006311	FLUOROURACIL	FLUOROURACIL	FLUOROURACIL	4/25/2011
10139006312	FLUOROURACIL	FLUOROURACIL	FLUOROURACIL	4/25/2011
10139006350	FLUOROURACIL	FLUOROURACIL	FLUOROURACIL	4/25/2011
38779002501	FLUOROURACIL	FLUOROURACIL	FLUOROURACIL	4/25/2011
38779002504	FLUOROURACIL	FLUOROURACIL	FLUOROURACIL	4/25/2011
38779002505	FLUOROURACIL	FLUOROURACIL	FLUOROURACIL	4/25/2011
38779002509	FLUOROURACIL	FLUOROURACIL	FLUOROURACIL	4/25/2011
51552073301	FLUOROURACIL	FLUOROURACIL	FLUOROURACIL	4/25/2011

51552073302	FLUOROURACIL	FLUOROURACIL	FLUOROURACIL	4/25/2011
51552073304	FLUOROURACIL	FLUOROURACIL	FLUOROURACIL	4/25/2011
51552073305	FLUOROURACIL	FLUOROURACIL	FLUOROURACIL	4/25/2011
51927108500	FLUOROURACIL	FLUOROURACIL	FLUOROURACIL	4/25/2011
53638059980	FLUOROURACIL	FLUOROURACIL	FLUOROURACIL	3/31/2016
54569140600	FLUOROURACIL	FLUOROURACIL	FLUOROURACIL	3/31/2016
68682008531	FLUOROURACIL	FLUOROURACIL	FLUOROURACIL	4/25/2011
39769001250	FLUOROURACIL	FLUOROURACIL	FLUOROURACIL 50MG/ML AMPUL	3/31/2016
00004197701	FLUOROURACIL	FLUOROURACIL	FLUOROURACIL 50MG/ML VIAL	4/25/2011
00469171060	FLUOROURACIL	FLUOROURACIL	FLUOROURACIL 50MG/ML VIAL	4/25/2011
00069017301	FLUOROURACIL	FLUOROURACIL	Fluorouracil Inj 1 GM/20ML (50 MG/ML)	6/16/2014
00069017302	FLUOROURACIL	FLUOROURACIL	Fluorouracil Inj 1 GM/20ML (50 MG/ML)	6/16/2014
10139006320	FLUOROURACIL	FLUOROURACIL	FLUOROURACIL INJ 1 GM/20ML (50 MG/ML)	4/22/2013
16729027667	FLUOROURACIL	FLUOROURACIL	Fluorouracil Inj 1 GM/20ML (50 MG/ML)	6/16/2014
00069017401	FLUOROURACIL	FLUOROURACIL	Fluorouracil Inj 2.5 GM/50ML (50 MG/ML)	6/16/2014
16729027611	FLUOROURACIL	FLUOROURACIL	Fluorouracil Inj 2.5 GM/50ML (50 MG/ML)	6/16/2014

00069017601	FLUOROURACIL	FLUOROURACIL	Fluorouracil Inj 5 GM/100ML (50 MG/ML)	6/16/2014
16729027638	FLUOROURACIL	FLUOROURACIL	Fluorouracil Inj 5 GM/100ML (50 MG/ML)	6/16/2014
00069016901	FLUOROURACIL	FLUOROURACIL	Fluorouracil Inj 500 MG/10ML (50 MG/ML)	6/16/2014
10139006310	FLUOROURACIL	FLUOROURACIL	FLUOROURACIL INJ 500 MG/10ML (50 MG/ML)	4/22/2013
16729027668	FLUOROURACIL	FLUOROURACIL	Fluorouracil Inj 500 MG/10ML (50 MG/ML)	6/16/2014
00187395364	FLUOROURACIL	FLUOROURACIL	FLUOROURACIL INJ 50MG/ML	4/25/2011
63323011751	FLUOROURACIL	FLUOROURACIL	FLUOROURACIL INJ 50MG/ML	4/25/2011
63323011761	FLUOROURACIL	FLUOROURACIL	FLUOROURACIL INJ 50MG/ML	4/25/2011
63323011710	FLUOROURACIL	FLUOROURACIL	FLUOROURACIL INJ 50MG/ML UD	4/25/2011
63323011720	FLUOROURACIL	FLUOROURACIL	FLUOROURACIL INJ 50MG/ML UD	4/25/2011
38779002503	FLUOROURACIL	FLUOROURACIL	FLUOROURCCIL (BULK) POWDER	4/22/2013
38779002506	FLUOROURACIL	FLUOROURACIL	FLUOROURCCIL (BULK) POWDER	4/22/2013
62991148604	FLUOROURACIL	FLUOROURACIL	FLUOROURCCIL (BULK) POWDER	4/22/2013
00013103691	FLUOROURACIL	FLUOROURACIL	#N/A	4/25/2011
00013103695	FLUOROURACIL	FLUOROURACIL	#N/A	4/25/2011
00013104694	FLUOROURACIL	FLUOROURACIL	#N/A	4/25/2011

00081039001	FLUOROURACIL	FLUOROURACIL	#N/A	4/25/2011
00081039003	FLUOROURACIL	FLUOROURACIL	#N/A	4/25/2011
00182306863	FLUOROURACIL	FLUOROURACIL	#N/A	4/25/2011
00418017110	FLUOROURACIL	FLUOROURACIL	#N/A	4/25/2011
00469171030	FLUOROURACIL	FLUOROURACIL	#N/A	4/25/2011
00469171040	FLUOROURACIL	FLUOROURACIL	#N/A	4/25/2011
00469171100	FLUOROURACIL	FLUOROURACIL	#N/A	4/25/2011
00702171030	FLUOROURACIL	FLUOROURACIL	#N/A	4/25/2011
10019095002	FLUOROURACIL	FLUOROURACIL	#N/A	4/25/2011
38779002510	FLUOROURACIL	FLUOROURACIL	#N/A	4/25/2011
38779002525	FLUOROURACIL	FLUOROURACIL	#N/A	4/25/2011
39769001210	FLUOROURACIL	FLUOROURACIL	#N/A	4/25/2011
39769001240	FLUOROURACIL	FLUOROURACIL	#N/A	4/25/2011
39769001290	FLUOROURACIL	FLUOROURACIL	#N/A	4/25/2011
51309021710	FLUOROURACIL	FLUOROURACIL	#N/A	4/25/2011
51309021720	FLUOROURACIL	FLUOROURACIL	#N/A	4/25/2011

51309021750	FLUOROURACIL	FLUOROURACIL	#N/A	4/25/2011
51309021798	FLUOROURACIL	FLUOROURACIL	#N/A	4/25/2011
51432040910	FLUOROURACIL	FLUOROURACIL	#N/A	4/25/2011
51432047010	FLUOROURACIL	FLUOROURACIL	#N/A	4/25/2011
53258171003	FLUOROURACIL	FLUOROURACIL	#N/A	4/25/2011
53258171100	FLUOROURACIL	FLUOROURACIL	#N/A	4/25/2011
53905011110	FLUOROURACIL	FLUOROURACIL	#N/A	4/25/2011
61703040932	FLUOROURACIL	FLUOROURACIL	#N/A	4/25/2011
61703040953	FLUOROURACIL	FLUOROURACIL	#N/A	4/25/2011
61703040967	FLUOROURACIL	FLUOROURACIL	#N/A	4/25/2011
49452317505	FLUOROURACIL	FLUOROURACIL (BULK) 100 % POWDER	FLUOROURACIL (BULK) 100 % MISC POWD	5/23/2017
60966013304	FLUOROURACIL	FLUOROURACIL (BULK) 100 % POWDER	FLUOROURACIL (BULK) 100 % MISC POWD	7/17/2015
60966023303	FLUOROURACIL	FLUOROURACIL (BULK) 100 % POWDER	FLUOROURACIL (BULK) 100 % MISC POWD	7/17/2015
60966033302	FLUOROURACIL	FLUOROURACIL (BULK) 100 % POWDER	FLUOROURACIL (BULK) 100 % MISC POWD	7/17/2015
60966043301	FLUOROURACIL	FLUOROURACIL (BULK) 100 %	FLUOROURACIL (BULK) 100 % MISC POWD	7/17/2015

		POWDER		
03877900251	FLUOROURACIL	FLUOROURACIL (BULK) 100 % POWDER	FLUOROURACIL (BULK) 100 % POWDER	3/31/2016
03877900253	FLUOROURACIL	FLUOROURACIL (BULK) 100 % POWDER	FLUOROURACIL (BULK) 100 % POWDER	3/31/2016
03877900254	FLUOROURACIL	FLUOROURACIL (BULK) 100 % POWDER	FLUOROURACIL (BULK) 100 % POWDER	3/31/2016
03877900255	FLUOROURACIL	FLUOROURACIL (BULK) 100 % POWDER	FLUOROURACIL (BULK) 100 % POWDER	3/31/2016
03877900256	FLUOROURACIL	FLUOROURACIL (BULK) 100 % POWDER	FLUOROURACIL (BULK) 100 % POWDER	3/31/2016
03877900259	FLUOROURACIL	FLUOROURACIL (BULK) 100 % POWDER	FLUOROURACIL (BULK) 100 % POWDER	3/31/2016
04945231751	FLUOROURACIL	FLUOROURACIL (BULK) 100 % POWDER	FLUOROURACIL (BULK) 100 % POWDER	3/31/2016
04945231752	FLUOROURACIL	FLUOROURACIL (BULK) 100 % POWDER	FLUOROURACIL (BULK) 100 % POWDER	3/31/2016
04945231753	FLUOROURACIL	FLUOROURACIL (BULK) 100 % POWDER	FLUOROURACIL (BULK) 100 % POWDER	3/31/2016
04945231754	FLUOROURACIL	FLUOROURACIL (BULK) 100 % POWDER	FLUOROURACIL (BULK) 100 % POWDER	3/31/2016
05155207331	FLUOROURACIL	FLUOROURACIL (BULK) 100 % POWDER	FLUOROURACIL (BULK) 100 % POWDER	3/31/2016

05155207332	FLUOROURACIL	FLUOROURACIL (BULK) 100 % POWDER	FLUOROURACIL (BULK) 100 % POWDER	3/31/2016
05155207334	FLUOROURACIL	FLUOROURACIL (BULK) 100 % POWDER	FLUOROURACIL (BULK) 100 % POWDER	3/31/2016
05155207335	FLUOROURACIL	FLUOROURACIL (BULK) 100 % POWDER	FLUOROURACIL (BULK) 100 % POWDER	3/31/2016
05192710850	FLUOROURACIL	FLUOROURACIL (BULK) 100 % POWDER	FLUOROURACIL (BULK) 100 % POWDER	3/31/2016
06096613304	FLUOROURACIL	FLUOROURACIL (BULK) 100 % POWDER	FLUOROURACIL (BULK) 100 % POWDER	3/31/2016
06096623303	FLUOROURACIL	FLUOROURACIL (BULK) 100 % POWDER	FLUOROURACIL (BULK) 100 % POWDER	3/31/2016
06096633302	FLUOROURACIL	FLUOROURACIL (BULK) 100 % POWDER	FLUOROURACIL (BULK) 100 % POWDER	3/31/2016
06096643301	FLUOROURACIL	FLUOROURACIL (BULK) 100 % POWDER	FLUOROURACIL (BULK) 100 % POWDER	3/31/2016
06299114861	FLUOROURACIL	FLUOROURACIL (BULK) 100 % POWDER	FLUOROURACIL (BULK) 100 % POWDER	3/31/2016
06299114862	FLUOROURACIL	FLUOROURACIL (BULK) 100 % POWDER	FLUOROURACIL (BULK) 100 % POWDER	3/31/2016
06299114863	FLUOROURACIL	FLUOROURACIL (BULK) 100 % POWDER	FLUOROURACIL (BULK) 100 % POWDER	3/31/2016
06299114864	FLUOROURACIL	FLUOROURACIL (BULK) 100 % POWDER	FLUOROURACIL (BULK) 100 % POWDER	3/31/2016

629911486 01	FLUOROUR ACIL	FLUOROURACIL (U.S.P.)	5-FLUOROURACIL	4/25/201 1
629911486 03	FLUOROUR ACIL	FLUOROURACIL (U.S.P.)	5-FLUOROURACIL	4/25/201 1
010139063 12	FLUOROUR ACIL	FLUOROURACIL 1 GRAM/20 ML INTRAVENOUS SO	FLUOROURACIL 1 GRAM/20 ML INTRAVENOU	3/31/201 6
010139063 20	FLUOROUR ACIL	FLUOROURACIL 1 GRAM/20 ML INTRAVENOUS SO	FLUOROURACIL 1 GRAM/20 ML INTRAVENOU	3/31/201 6
016729276 05	FLUOROUR ACIL	FLUOROURACIL 1 GRAM/20 ML INTRAVENOUS SO	FLUOROURACIL 1 GRAM/20 ML INTRAVENOU	3/31/201 6
016729276 67	FLUOROUR ACIL	FLUOROURACIL 1 GRAM/20 ML INTRAVENOUS SO	FLUOROURACIL 1 GRAM/20 ML INTRAVENOU	3/31/201 6
063323117 20	FLUOROUR ACIL	FLUOROURACIL 1 GRAM/20 ML INTRAVENOUS SO	FLUOROURACIL 1 GRAM/20 ML INTRAVENOU	3/31/201 6
063323117 28	FLUOROUR ACIL	FLUOROURACIL 1 GRAM/20 ML INTRAVENOUS SO	FLUOROURACIL 1 GRAM/20 ML INTRAVENOU	3/31/201 6
068001266 29	FLUOROUR ACIL	FLUOROURACIL 1 GRAM/20 ML INTRAVENOUS SO	FLUOROURACIL 1 GRAM/20 ML INTRAVENOU	3/31/201 6
068001266 31	FLUOROUR ACIL	FLUOROURACIL 1 GRAM/20 ML INTRAVENOUS SO	FLUOROURACIL 1 GRAM/20 ML INTRAVENOU	3/31/201 6
010139063 50	FLUOROUR ACIL	FLUOROURACIL 2.5 GRAM/50 ML INTRAVENOUS	FLUOROURACIL 2.5 GRAM/50 ML INTRAVEN	3/31/201 6
016729276 11	FLUOROUR ACIL	FLUOROURACIL 2.5 GRAM/50 ML INTRAVENOUS	FLUOROURACIL 2.5 GRAM/50 ML INTRAVEN	3/31/201 6

06332311751	FLUOROURACIL	FLUOROURACIL 2.5 GRAM/50 ML INTRAVENOUS	FLUOROURACIL 2.5 GRAM/50 ML INTRAVEN	3/31/2016
06332311758	FLUOROURACIL	FLUOROURACIL 2.5 GRAM/50 ML INTRAVENOUS	FLUOROURACIL 2.5 GRAM/50 ML INTRAVEN	3/31/2016
06332311759	FLUOROURACIL	FLUOROURACIL 2.5 GRAM/50 ML INTRAVENOUS	FLUOROURACIL 2.5 GRAM/50 ML INTRAVEN	3/31/2016
06800126624	FLUOROURACIL	FLUOROURACIL 2.5 GRAM/50 ML INTRAVENOUS	FLUOROURACIL 2.5 GRAM/50 ML INTRAVEN	3/31/2016
06800126627	FLUOROURACIL	FLUOROURACIL 2.5 GRAM/50 ML INTRAVENOUS	FLUOROURACIL 2.5 GRAM/50 ML INTRAVEN	3/31/2016
25021021598	FLUOROURACIL	FLUOROURACIL 2.5 GRAM/50 ML INTRAVENOUS SOLUTION	FLUOROURACIL 2.5 GRAM/50 ML IV SOLN	2/1/2017
63323011759	FLUOROURACIL	FLUOROURACIL 2.5 GRAM/50 ML INTRAVENOUS SOLUTION	FLUOROURACIL 2.5 GRAM/50 ML IV SOLN	7/17/2015
01013906301	FLUOROURACIL	FLUOROURACIL 5 GRAM/100 ML INTRAVENOUS S	FLUOROURACIL 5 GRAM/100 ML INTRAVENO	3/31/2016
01672927638	FLUOROURACIL	FLUOROURACIL 5 GRAM/100 ML INTRAVENOUS S	FLUOROURACIL 5 GRAM/100 ML INTRAVENO	3/31/2016
06332311761	FLUOROURACIL	FLUOROURACIL 5 GRAM/100 ML INTRAVENOUS S	FLUOROURACIL 5 GRAM/100 ML INTRAVENO	3/31/2016
06332311768	FLUOROURACIL	FLUOROURACIL 5 GRAM/100 ML INTRAVENOUS S	FLUOROURACIL 5 GRAM/100 ML INTRAVENO	3/31/2016

06332311769	FLUOROURACIL	FLUOROURACIL 5 GRAM/100 ML INTRAVENOUS S	FLUOROURACIL 5 GRAM/100 ML INTRAVENO	3/31/2016
06800126632	FLUOROURACIL	FLUOROURACIL 5 GRAM/100 ML INTRAVENOUS S	FLUOROURACIL 5 GRAM/100 ML INTRAVENO	3/31/2016
06800126633	FLUOROURACIL	FLUOROURACIL 5 GRAM/100 ML INTRAVENOUS S	FLUOROURACIL 5 GRAM/100 ML INTRAVENO	3/31/2016
25021021599	FLUOROURACIL	FLUOROURACIL 5 GRAM/100 ML INTRAVENOUS SOLUTION	FLUOROURACIL 5 GRAM/100 ML IV SOLN	2/1/2017
63323011769	FLUOROURACIL	FLUOROURACIL 5 GRAM/100 ML INTRAVENOUS SOLUTION	FLUOROURACIL 5 GRAM/100 ML IV SOLN	7/17/2015
01013906310	FLUOROURACIL	FLUOROURACIL 500 MG/10 ML INTRAVENOUS SO	FLUOROURACIL 500 MG/10 ML INTRAVENOU	3/31/2016
01013906311	FLUOROURACIL	FLUOROURACIL 500 MG/10 ML INTRAVENOUS SO	FLUOROURACIL 500 MG/10 ML INTRAVENOU	3/31/2016
01672927603	FLUOROURACIL	FLUOROURACIL 500 MG/10 ML INTRAVENOUS SO	FLUOROURACIL 500 MG/10 ML INTRAVENOU	3/31/2016
01672927668	FLUOROURACIL	FLUOROURACIL 500 MG/10 ML INTRAVENOUS SO	FLUOROURACIL 500 MG/10 ML INTRAVENOU	3/31/2016
06332311710	FLUOROURACIL	FLUOROURACIL 500 MG/10 ML INTRAVENOUS SO	FLUOROURACIL 500 MG/10 ML INTRAVENOU	3/31/2016
06332311718	FLUOROURACIL	FLUOROURACIL 500 MG/10 ML INTRAVENOUS SO	FLUOROURACIL 500 MG/10 ML INTRAVENOU	3/31/2016

06332311719	FLUOROURACIL	FLUOROURACIL 500 MG/10 ML INTRAVENOUS SO	FLUOROURACIL 500 MG/10 ML INTRAVENOU	3/31/2016
06800126628	FLUOROURACIL	FLUOROURACIL 500 MG/10 ML INTRAVENOUS SO	FLUOROURACIL 500 MG/10 ML INTRAVENOU	3/31/2016
06800126630	FLUOROURACIL	FLUOROURACIL 500 MG/10 ML INTRAVENOUS SO	FLUOROURACIL 500 MG/10 ML INTRAVENOU	3/31/2016
63323011719	FLUOROURACIL	FLUOROURACIL 500 MG/10 ML INTRAVENOUS SOLUTION	FLUOROURACIL 500 MG/10 ML IV SOLN	7/17/2015
16729027605	FLUOROURACIL	Fluorouracil Inj 1 GM/20ML (50 MG/ML)	#N/A	7/17/2015
63323011728	FLUOROURACIL	Fluorouracil Inj 1 GM/20ML (50 MG/ML)	#N/A	7/17/2015
68001026629	FLUOROURACIL	Fluorouracil Inj 1 GM/20ML (50 MG/ML)	#N/A	7/17/2015
68001026631	FLUOROURACIL	Fluorouracil Inj 1 GM/20ML (50 MG/ML)	#N/A	7/17/2015
63323011758	FLUOROURACIL	Fluorouracil Inj 2.5 GM/50ML (50 MG/ML)	#N/A	7/17/2015
68001026624	FLUOROURACIL	Fluorouracil Inj 2.5 GM/50ML (50 MG/ML)	#N/A	7/17/2015
68001026627	FLUOROURACIL	Fluorouracil Inj 2.5 GM/50ML (50 MG/ML)	#N/A	7/17/2015
63323011768	FLUOROURACIL	Fluorouracil Inj 5 GM/100ML (50	#N/A	7/17/2015

		MG/ML)		
68001026632	FLUOROURACIL	Fluorouracil Inj 5 GM/100ML (50 MG/ML)	#N/A	7/17/2015
68001026633	FLUOROURACIL	Fluorouracil Inj 5 GM/100ML (50 MG/ML)	#N/A	7/17/2015
66758004401	FLUOROURACIL	FLUOROURACIL INJ 50 MG/ML	FLUOROURACIL	5/31/2012
66758004403	FLUOROURACIL	FLUOROURACIL INJ 50 MG/ML	FLUOROURACIL	5/31/2012
00069016902	FLUOROURACIL	Fluorouracil Inj 500 MG/10ML (50 MG/ML)	#N/A	7/17/2015
16729027603	FLUOROURACIL	Fluorouracil Inj 500 MG/10ML (50 MG/ML)	#N/A	7/17/2015
63323011718	FLUOROURACIL	Fluorouracil Inj 500 MG/10ML (50 MG/ML)	#N/A	7/17/2015
68001026628	FLUOROURACIL	Fluorouracil Inj 500 MG/10ML (50 MG/ML)	#N/A	7/17/2015
68001026630	FLUOROURACIL	Fluorouracil Inj 500 MG/10ML (50 MG/ML)	#N/A	7/17/2015
55045210308	FLUOROURACIL	FLUOROURACIL SOLN 1%	FLUOROPLEX SOLN 1 % EX	3/12/2018
51672406201	FLUOROURACIL	FLUOROURACIL SOLN 2 %	FLUOROURACIL 2 % SOL TARO	3/12/2018
00187320202	FLUOROURACIL	FLUOROURACIL SOLN 2 % VALE	EFUDEX 2 % SOL VALE	3/12/2018
00187320210	FLUOROURACIL	FLUOROURACIL SOLN 2 % VALE	EFUDEX 2 % SOL VALE	3/12/2018

51672406301	FLUOROURACIL	FLUOROURACIL SOLN 5 %	FLUOROURACIL 5 % SOL TARO	3/12/2018
43547025801	FLUOROURACIL	FLUOROURACIL SOLN 5 % DRO SOLC	FLUOROURACIL 5 % DRO SOLC	3/12/2018
00187320310	FLUOROURACIL	FLUOROURACIL SOLN 5 % VALE	EFUDEX 5 % SOL VALE	3/12/2018
00004170506	FLUOROURACIL	FLUOROURACIL SOLN 5%	EFUDEX 5PC SOLUTION	3/12/2018
21695082940	FLUOROURACIL	#N/A	FLUOROURACIL	3/31/2016
54569627900	FLUOROURACIL	#N/A	FLUOROURACIL	3/31/2016
54868629300	FLUOROURACIL	#N/A	FLUOROURACIL	3/31/2016
44778017101	FLUOROURACIL	#N/A	FLUOROURACIL-TRIAMCINOLONE 20-1 MG/0.5 ML INTRALESIONAL INJ	2/1/2017

Table S5. Targeted therapy CPT/HCPCS procedure codes.

PX2	CHEMOCAT	CODE_TYPE	DESCRIPTION
S0116	BEVACIZUMAB	HCPC	BEVACIZUMAB 100 MG
C9257	BEVACIZUMAB	HCPC	Injection, bevacizumab, 0.25 mg
Q2024	BEVACIZUMAB	HCPC	Injection, bevacizumab, 0.25 mg
J9035	BEVACIZUMAB	HCPC	Injection, bevacizumab, 10 mg
C9214	BEVACIZUMAB	HCPC	INJECTION, BEVACIZUMAB, PER 10 MG
J9330	TEMSIROLIMUS	HCPC	INJECTION, TEMSIROLIMUS, 1 MG

Table S6. Targeted therapy National Drug Codes.

National Drug Code	CHEMOCAT	GENERIC_NAME	BRAND_NAME	DATE_ADDED
00069014501	AXITINIB	AXITINIB	INLYTA	5/21/2012
00069015111	AXITINIB	AXITINIB	INLYTA	5/21/2012
42388001214	CABOZANTINIB	CABOZANTINIB	CABOZANTINIB S-MAL CAP 1 X 80 MG & 1 X 20 MG (100 Dose) Kit	4/22/2013
42388001114	CABOZANTINIB	CABOZANTINIB	CABOZANTINIB S-MAL CAP 1 X 80 MG & 3 X 20 MG (140 Dose) Kit	4/22/2013
42388001314	CABOZANTINIB	CABOZANTINIB	CABOZANTINIB S-MALATE CAP 3 X 20 MG (60 MG Dose) Kit	4/22/2013
04238801214	CABOZANTINIB	CABOZANTINIB 100 MG/DAY (80 MG	COMETRIQ 100 MG/DAY(80 MG[1]-20 MG[1	3/31/2016
04238801114	CABOZANTINIB	CABOZANTINIB 140 MG/DAY (80 MG	COMETRIQ 140 MG/DAY(80 MG[1]-20 MG[3	3/31/2016
04238801314	CABOZANTINIB	CABOZANTINIB 60 MG/DAY (20 MG	COMETRIQ 60 MG/DAY (20 MG [3]/DAY) C	3/31/2016
42388002426	CABOZANTINIB	Cabozantinib S-Malate Tab 20 MG (Base Equivalent)	#N/A	2/1/2017
42388002526	CABOZANTINIB	Cabozantinib S-Malate Tab 40 MG (Base Equivalent)	#N/A	2/1/2017
42388002326	CABOZANTINIB	Cabozantinib S-Malate Tab 60 MG (Base Equivalent)	#N/A	2/1/2017

05024206 301	ERLOTINIB	ERLOTINIB 100 MG TABLET	TARCEVA 100 MG TABLET	3/31/2016
05486854 740	ERLOTINIB	ERLOTINIB 100 MG TABLET	TARCEVA 100 MG TABLET	3/31/2016
05024206 401	ERLOTINIB	ERLOTINIB 150 MG TABLET	TARCEVA 150 MG TABLET	3/31/2016
05486854 470	ERLOTINIB	ERLOTINIB 150 MG TABLET	TARCEVA 150 MG TABLET	3/31/2016
05024206 201	ERLOTINIB	ERLOTINIB 25 MG TABLET	TARCEVA 25 MG TABLET	3/31/2016
05486852 900	ERLOTINIB	ERLOTINIB 25 MG TABLET	TARCEVA 25 MG TABLET	3/31/2016
54868529 000	ERLOTINIB	ERLOTINIB HCL	TARCEVA	4/25/2011
54868544 700	ERLOTINIB	ERLOTINIB HCL	TARCEVA	4/25/2011
54868547 400	ERLOTINIB	ERLOTINIB HCL	TARCEVA	4/25/2011
50242006 301	ERLOTINIB	ERLOTINIB HCL	TARCEVA TAB 100MG	4/25/2011
54569584 700	ERLOTINIB	ERLOTINIB HCL	TARCEVA TAB 100MG	5/31/2012
50242006 401	ERLOTINIB	ERLOTINIB HCL	TARCEVA TAB 150MG	4/25/2011
54569584 800	ERLOTINIB	ERLOTINIB HCL	TARCEVA TAB 150MG	5/31/2012
50242006 201	ERLOTINIB	ERLOTINIB HCL	TARCEVA TAB 25MG	4/25/2011
00078062 651	EVEROLIMUS	AFINITOR DISPERZ	Everolimus Tab for Oral Susp 2 MG	6/16/2014
00078062 661	EVEROLIMUS	AFINITOR DISPERZ	Everolimus Tab for Oral Susp 2 MG	6/16/2014

00078062 751	EVEROLIMUS	AFINITOR DISPERZ	Everolimus Tab for Oral Susp 3 MG	6/16/2014
00078062 761	EVEROLIMUS	AFINITOR DISPERZ	Everolimus Tab for Oral Susp 3 MG	6/16/2014
00078062 851	EVEROLIMUS	AFINITOR DISPERZ	Everolimus Tab for Oral Susp 5 MG	6/16/2014
00078062 861	EVEROLIMUS	AFINITOR DISPERZ	Everolimus Tab for Oral Susp 5 MG	6/16/2014
00078056 751	EVEROLIMUS	EVEROLIMUS	AFINITOR TAB 10MG	4/25/2011
00078056 651	EVEROLIMUS	EVEROLIMUS	AFINITOR TAB 5MG	4/25/2011
00078041 761	EVEROLIMUS	EVEROLIMUS	EVEROLIMUS TAB 0.25 MG	5/31/2012
00078041 461	EVEROLIMUS	EVEROLIMUS	EVEROLIMUS TAB 0.5 MG	5/31/2012
00078041 561	EVEROLIMUS	EVEROLIMUS	EVEROLIMUS TAB 0.75 MG	5/31/2012
00078056 761	EVEROLIMUS	EVEROLIMUS	EVEROLIMUS TAB 10 MG	5/31/2012
00078059 451	EVEROLIMUS	EVEROLIMUS	EVEROLIMUS TAB 2.5 MG	5/31/2012
00078059 461	EVEROLIMUS	EVEROLIMUS	EVEROLIMUS TAB 2.5 MG	5/31/2012
00078056 661	EVEROLIMUS	EVEROLIMUS	EVEROLIMUS TAB 5 MG	5/31/2012
00078062 051	EVEROLIMUS	EVEROLIMUS	EVEROLIMUS TAB 7.5 MG	5/31/2012
00078062 061	EVEROLIMUS	EVEROLIMUS	EVEROLIMUS TAB 7.5 MG	5/31/2012
00078041 720	EVEROLIMUS	EVEROLIMUS	ZORTRESS TAB 0.25MG	5/21/2012

00078041 420	EVEROLIMUS	EVEROLIMUS	ZORTRESS TAB 0.5MG	5/21/2012
00078041 520	EVEROLIMUS	EVEROLIMUS	ZORTRESS TAB 0.75MG	5/21/2012
00173080 409	PAZOPANIB	PAZOPANIB HCL	VOTRIENT TAB 200MG	4/25/2011
00078067 066	PAZOPANIB	Pazopanib HCl Tab 200 MG (Base Equiv)	#N/A	2/1/2017
05041948 858	SORAFENIB	SORAFENIB 200 MG TABLET	NEXAVAR 200 MG TABLET	3/31/2016
00026848 858	SORAFENIB	SORAFENIB TOSYLATE	NEXAVAR TAB 200MG	4/25/2011
50419048 858	SORAFENIB	SORAFENIB TOSYLATE TAB 200 MG (BASE EQUIVALENT)	NEXAVAR	5/31/2012
99999090 198	SORAFENIB	#N/A	STUDY E-2805: SORAFENIB 200	6/16/2014
99999090 078	SORAFENIB	#N/A	STUDY RENAL CELL-BAY: SORAFENIB	6/16/2014
54569598 200	SUNITINIB	SUNITINIB MALATE	SUTENT	4/25/2011
54569598 300	SUNITINIB	SUNITINIB MALATE	SUTENT	4/25/2011
54868557 300	SUNITINIB	SUNITINIB MALATE	SUTENT	3/31/2016
00069055 038	SUNITINIB	SUNITINIB MALATE	SUTENT CAP 12.5MG	4/25/2011
00069077 030	SUNITINIB	SUNITINIB MALATE	SUTENT CAP 25MG	4/25/2011
00069077 038	SUNITINIB	SUNITINIB MALATE	SUTENT CAP 25MG	4/25/2011

00069098 030	SUNITINIB	SUNITINIB MALATE	SUTENT CAP 50MG	4/25/2011
00069098 038	SUNITINIB	SUNITINIB MALATE	SUTENT CAP 50MG	4/25/2011
00069055 030	SUNITINIB	SUNITINIB MALATE	#N/A	4/25/2011
00069083 038	SUNITINIB	Sunitinib Malate Cap 37.5 MG (Base Equivalent)	#N/A	7/17/2015
99999090 321	SUNITINIB	#N/A	STUDY E-2805: SUNITINIB 50M	6/16/2014

Table S7. Autoimmune disease ICD diagnosis codes. 56 categories of ICD codes associated with autoimmune diseases, including those for thyroiditis, type I diabetes mellitus, myalgia and myositis, chronic hepatitis, psoriasis vulgaris, inflammatory bowel disease (IBD, including ulcerative colitis and Crohn's disease), rheumatoid arthritis, Raynaud's syndrome, polymyalgia rheumatica, sarcoidosis, ankylosing spondylitis, autoimmune hemolytic anemias, systemic lupus erythematosus, biliary cirrhosis, celiac disease, diffuse diseases of connective tissue, peripheral autonomic neuropathy, psoriatic arthropathy, sicca syndrome, systemic sclerosis, autoimmune disease not elsewhere classified (NEC), "other immunological findings", "other disorders of the immune mechanism", acute rheumatic heart disease, alopecia, arthropathy, Behcet's syndrome, bullous dermatoses, dermatomyositis, diseases of hair and hair follicles, encephalitis, endocarditis, giant cell arteritis, glomerulonephritis, glucocorticoid deficiency, graves' disease, hypothyroidism NOS, inflammatory and toxic neuropathy, multiple sclerosis, myoneural disorders, nephritis, "other demyelinating diseases of central nervous system", "other diseases of lung", "other endocrine disorders", "other specified diffuse diseases of connective tissue", pemphigus and pemphigoid, polyarteritis nodosa and allied conditions, polymyositis, primary thrombocytopenia, Reiter's disease, rheumatic fever / chorea, thrombotic microangiopathy, thyrotoxicosis with or without goiter, toxic erythema, vitiligo, and Wegener's granulomatosis were examined.

ICD-version	ICD-code	ICD-description	PheWAS-code	PheWAS-string	disease-category
ICD9 CM	135	Sarcoidosis	697	Sarcoidosis	Sarcoidosis
ICD9 CM	245	Thyroiditis	245	Thyroiditis	Thyroiditis
ICD9 CM	245	Acute thyroiditis	245.1	Thyroiditis, acute and subacute	Thyroiditis
ICD9 CM	245.1	Subacute thyroiditis	245.1	Thyroiditis, acute and subacute	Thyroiditis
ICD9 CM	245.2	Chronic lymphocytic thyroiditis	245.2	Chronic lymphocytic thyroiditis	Thyroiditis
ICD9 CM	245.3	Chronic fibrous thyroiditis	245	Thyroiditis	Thyroiditis
ICD9 CM	245.8	Other and unspecified chronic thyroiditis	245	Thyroiditis	Thyroiditis
ICD9 CM	245.9	Thyroiditis, unspecified	245	Thyroiditis	Thyroiditis

ICD9 CM	250.01	Diabetes mellitus without mention of complication, type I [juvenile type], not stated as uncontrolled	250.1	Type 1 diabetes	DM
ICD9 CM	250.03	Diabetes mellitus without mention of complication, type I [juvenile type], uncontrolled	250.11	Type 1 diabetic ketoacidosis	DM
ICD9 CM	250.1	Diabetes with ketoacidosis	250	Diabetes mellitus	DM
ICD9 CM	250.11	Diabetes with ketoacidosis, type I [juvenile type], not stated as uncontrolled	250.11	Type 1 diabetic ketoacidosis	DM
ICD9 CM	250.13	Diabetes with ketoacidosis, type I [juvenile type], uncontrolled	250.11	Type 1 diabetic ketoacidosis	DM
ICD9 CM	250.21	Diabetes with hyperosmolarity, type I [juvenile type], not stated as uncontrolled	250.1	Type 1 diabetes	DM
ICD9 CM	250.23	Diabetes with hyperosmolarity, type I [juvenile type], uncontrolled	250.1	Type 1 diabetes	DM
ICD9 CM	250.31	Diabetes with other coma, type I [juvenile type], not stated as uncontrolled	250.1	Type 1 diabetes	DM
ICD9 CM	250.33	Diabetes with other coma, type I [juvenile type], uncontrolled	250.1	Type 1 diabetes	DM
ICD9 CM	250.41	Diabetes with renal manifestations, type I [juvenile type], not stated as uncontrolled	250.12	Type 1 diabetes nephropathy	DM
ICD9 CM	250.43	Diabetes with renal manifestations, type I	250.12	Type 1 diabetes nephropathy	DM

		[juvenile type], uncontrolled			
ICD9 CM	250.51	Diabetes with ophthalmic manifestations, type I [juvenile type], not stated as uncontrolled	250.1 3	Type 1 diabetic retinopathy	DM
ICD9 CM	250.53	Diabetes with ophthalmic manifestations, type I [juvenile type], uncontrolled	250.1 3	Type 1 diabetic retinopathy	DM
ICD9 CM	250.61	Diabetes with neurological manifestations, type I [juvenile type], not stated as uncontrolled	250.1 4	Type 1 diabetic neuropathy	DM
ICD9 CM	250.63	Diabetes with neurological manifestations, type I [juvenile type], uncontrolled	250.1 4	Type 1 diabetic neuropathy	DM
ICD9 CM	250.71	Diabetes with peripheral circulatory disorders, type I [juvenile type], not stated as uncontrolled	250.1 5	Type 1 diabetic peripheral circulatory disorders	DM
ICD9 CM	250.73	Diabetes with peripheral circulatory disorders, type I [juvenile type], uncontrolled	250.1 5	Type 1 diabetic peripheral circulatory disorders	DM
ICD9 CM	250.81	Diabetes with other specified manifestations, type I [juvenile type], not stated as uncontrolled	250.1	Type 1 diabetes	DM
ICD9 CM	250.83	Diabetes with other specified manifestations, type I [juvenile type], uncontrolled	250.1	Type 1 diabetes	DM
ICD9 CM	250.91	Diabetes with unspecified complication, type I [juvenile type], not stated	250.1	Type 1 diabetes	DM

		as uncontrolled			
ICD9 CM	250.93	Diabetes with unspecified complication, type I [juvenile type], uncontrolled	250.1	Type 1 diabetes	DM
ICD9 CM	279.49	Autoimmune disease, not elsewhere classified	279.2	Autoimmune disease NEC	Autoimmune disease NEC
ICD9 CM	283	Autoimmune hemolytic anemias	283.1	Autoimmune hemolytic anemias	Autoimmune hemolytic anemias
ICD9 CM	337.1	Peripheral autonomic neuropathy in disorders classified elsewhere	337.1	Peripheral autonomic neuropathy	Peripheral autonomic neuropathy
ICD9 CM	443	Raynaud's syndrome	443.1	Raynaud's syndrome	Raynaud's syndrome
ICD9 CM	555	Regional enteritis of small intestine	555.1	Crohn's disease	IBD
ICD9 CM	555.1	Regional enteritis of large intestine	555.1	Crohn's disease	IBD
ICD9 CM	555.2	Regional enteritis of small intestine with large intestine	555.1	Crohn's disease	IBD
ICD9 CM	555.9	Regional enteritis of unspecified site	555.1	Crohn's disease	IBD
ICD9 CM	556	Ulcerative colitis	555.2	Ulcerative colitis	IBD
ICD9 CM	556	Ulcerative (chronic) enterocolitis	555.2	Ulcerative colitis	IBD
ICD9 CM	556.1	Ulcerative (chronic) ileocolitis	555.2	Ulcerative colitis	IBD
ICD9 CM	556.2	Ulcerative (chronic) proctitis	555.2	Ulcerative colitis	IBD

ICD9 CM	556.3	Ulcerative (chronic) proctosigmoiditis	555.2	Ulcerative colitis	IBD
ICD9 CM	556.4	Pseudopolyposis of colon	555.2	Ulcerative colitis	IBD
ICD9 CM	556.5	Left-sided ulcerative (chronic) colitis	555.2	Ulcerative colitis	IBD
ICD9 CM	556.6	Universal ulcerative (chronic) colitis	555.2	Ulcerative colitis	IBD
ICD9 CM	556.8	Other ulcerative colitis	555.2	Ulcerative colitis	IBD
ICD9 CM	556.9	Ulcerative colitis, unspecified	555.2	Ulcerative colitis	IBD
ICD9 CM	571.42	Autoimmune hepatitis	70.4	Chronic hepatitis	Chronic hepatitis
ICD9 CM	571.6	Biliary cirrhosis	571.6	Biliary cirrhosis	Biliary cirrhosis
ICD9 CM	579	Celiac disease	557.11	Celiac disease	Celiac disease
ICD9 CM	696	Psoriatic arthropathy	696.42	Psoriatic arthropathy	Psoriatic arthropathy
ICD9 CM	696.1	Other psoriasis	696.41	Psoriasis vulgaris	Psoriasis vulgaris
ICD9 CM	710	Systemic lupus erythematosus	695.42	Systemic lupus erythematosus	Systemic lupus erythematosus
ICD9 CM	710.1	Systemic sclerosis	709.3	Systemic sclerosis	Systemic sclerosis
ICD9 CM	710.2	Sicca syndrome	709.2	Sicca syndrome	Sicca syndrome
ICD9 CM	710.9	Unspecified diffuse connective tissue disease	709	Diffuse diseases of connective tissue	Diffuse diseases of connective tissue

ICD9 CM	714	Rheumatoid arthritis	714.1	Rheumatoid arthritis	RA
ICD9 CM	714.3	Polyarticular juvenile rheumatoid arthritis, chronic or unspecified	714.2	Juvenile rheumatoid arthritis	RA
ICD9 CM	720	Ankylosing spondylitis	715.2	Ankylosing spondylitis	Ankylosing spondylitis
ICD9 CM	725	Polymyalgia rheumatica	717	Polymyalgia Rheumatica	Polymyalgia Rheumatica
ICD9 CM	795.79	Other and unspecified nonspecific immunological findings	279.7	Other immunological findings	Other immunological findings
ICD10 CM	D59.0	Drug-induced autoimmune hemolytic anemia	283.1	Autoimmune hemolytic anemias	Autoimmune hemolytic anemias
ICD10 CM	D59.1	Cold type (secondary) (symptomatic) hemolytic anemia	283.1	Autoimmune hemolytic anemias	Autoimmune hemolytic anemias
ICD10 CM	D86.9	Sarcoidosis, unspecified	697	Sarcoidosis	Sarcoidosis
ICD10 CM	D89.89	Other specified disorders involving the immune mechanism, not elsewhere classified	279.8	Other disorders of the immune mechanism	Other disorders of the immune mechanism
ICD10 CM	E06.1	Nonsuppurative thyroiditis	245.1	Thyroiditis, acute and subacute	Thyroiditis
ICD10 CM	E06.3	Hashitoxicosis (transient)	245.2	Chronic lymphocytic thyroiditis	Thyroiditis
ICD10 CM	E06.5	Chronic fibrous thyroiditis	245	Thyroiditis	Thyroiditis
ICD10 CM	E06.9	Thyroiditis, unspecified	245	Thyroiditis	Thyroiditis
ICD10 CM	E10.10	Type 1 diabetes mellitus with ketoacidosis without	250.11	Type 1 diabetic ketoacidosis	DM

		coma			
ICD10 CM	E10.21	Type 1 diabetes mellitus with intercapillary glomerulosclerosis	250.1 2	Type 1 diabetes nephropathy	DM
ICD10 CM	E10.29	Type 1 diabetes mellitus with other diabetic kidney complication	250.1 2	Type 1 diabetes nephropathy	DM
ICD10 CM	E10.31 1	Type 1 diabetes mellitus with unspecified diabetic retinopathy with macular edema	250.1 3	Type 1 diabetic retinopathy	DM
ICD10 CM	E10.31 9	Type 1 diabetes mellitus with unspecified diabetic retinopathy without macular edema	250.1 3	Type 1 diabetic retinopathy	DM
ICD10 CM	E10.36	Type 1 diabetes mellitus with diabetic cataract	250.1 3	Type 1 diabetic retinopathy	DM
ICD10 CM	E10.37 X1	Type 1 diabetes mellitus with diabetic macular edema, resolved following treatment, right eye	250.1 3	Type 1 diabetic retinopathy	DM
ICD10 CM	E10.37 X2	Type 1 diabetes mellitus with diabetic macular edema, resolved following treatment, left eye	250.1 3	Type 1 diabetic retinopathy	DM
ICD10 CM	E10.37 X3	Type 1 diabetes mellitus with diabetic macular edema, resolved following treatment, bilateral	250.1 3	Type 1 diabetic retinopathy	DM
ICD10 CM	E10.37 X9	Type 1 diabetes mellitus with diabetic macular edema, resolved following treatment, unspecified eye	250.1 3	Type 1 diabetic retinopathy	DM
ICD10 CM	E10.39	Type 1 diabetes mellitus with other diabetic ophthalmic complication	250.1 3	Type 1 diabetic retinopathy	DM

ICD10 CM	E10.40	Type 1 diabetes mellitus with diabetic neuropathy, unspecified	250.14	Type 1 diabetic neuropathy	DM
ICD10 CM	E10.42	Type 1 diabetes mellitus with diabetic polyneuropathy	250.6	Polyneuropathy in diabetes	DM
ICD10 CM	E10.51	Type 1 diabetes mellitus with diabetic peripheral angiopathy without gangrene	250.15	Type 1 diabetic peripheral circulatory disorders	DM
ICD10 CM	E10.65	Type 1 diabetes mellitus with hyperglycemia	250.1	Type 1 diabetes	DM
ICD10 CM	E10.8	Type 1 diabetes mellitus with unspecified complications	250.1	Type 1 diabetes	DM
ICD10 CM	E10.9	Type 1 diabetes mellitus without complications	250.1	Type 1 diabetes	DM
ICD10 CM	I73.00	Raynaud's syndrome without gangrene	443.1	Raynaud's syndrome	Raynaud's syndrome
ICD10 CM	I73.01	Raynaud's syndrome with gangrene	443.1	Raynaud's syndrome	Raynaud's syndrome
ICD10 CM	K50.00	Crohn's disease of small intestine without complications	555.1	Crohn's disease	IBD
ICD10 CM	K50.10	Crohn's disease of large intestine without complications	555.1	Crohn's disease	IBD
ICD10 CM	K50.80	Crohn's disease of both small and large intestine without complications	555.1	Crohn's disease	IBD
ICD10 CM	K50.90	Crohn's disease, unspecified, without complications	555.1	Crohn's disease	IBD
ICD10 CM	K51.00	Ulcerative (chronic) pancolitis without	555.2	Ulcerative colitis	IBD

		complications			
ICD10 CM	K51.20	Ulcerative (chronic) proctitis without complications	555.2	Ulcerative colitis	IBD
ICD10 CM	K51.30	Ulcerative (chronic) rectosigmoiditis without complications	555.2	Ulcerative colitis	IBD
ICD10 CM	K51.40	Inflammatory polyps of colon without complications	555.2	Ulcerative colitis	IBD
ICD10 CM	K51.50	Left sided colitis without complications	555.2	Ulcerative colitis	IBD
ICD10 CM	K51.80	Other ulcerative colitis without complications	555.2	Ulcerative colitis	IBD
ICD10 CM	K51.90	Ulcerative colitis, unspecified, without complications	555.2	Ulcerative colitis	IBD
ICD10 CM	K74.3	Chronic nonsuppurative destructive cholangitis	571.6	Biliary cirrhosis	Biliary cirrhosis
ICD10 CM	K74.4	Secondary biliary cirrhosis	571.6	Biliary cirrhosis	Biliary cirrhosis
ICD10 CM	K74.5	Biliary cirrhosis, unspecified	571.6	Biliary cirrhosis	Biliary cirrhosis
ICD10 CM	K75.4	Autoimmune hepatitis	70.4	Chronic hepatitis	Chronic hepatitis
ICD10 CM	K90.0	Celiac disease with steatorrhea	557.1 1	Celiac disease	Celiac disease
ICD10 CM	L40.0	Psoriasis vulgaris	696.4 1	Psoriasis vulgaris	Psoriasis vulgaris
ICD10 CM	L40.1	Generalized pustular psoriasis	696.4 1	Psoriasis vulgaris	Psoriasis vulgaris
ICD10 CM	L40.2	Acrodermatitis continua	696.4 1	Psoriasis vulgaris	Psoriasis vulgaris

ICD10 CM	L40.3	Pustulosis palmaris et plantaris	696.4 1	Psoriasis vulgaris	Psoriasis vulgaris
ICD10 CM	L40.4	Guttate psoriasis	696.4 1	Psoriasis vulgaris	Psoriasis vulgaris
ICD10 CM	L40.50	Arthropathic psoriasis, unspecified	696.4 2	Psoriatic arthropathy	Psoriatic arthropathy
ICD10 CM	L40.54	Psoriatic juvenile arthropathy	696.4 2	Psoriatic arthropathy	Psoriatic arthropathy
ICD10 CM	L40.59	Other psoriatic arthropathy	696.4 2	Psoriatic arthropathy	Psoriatic arthropathy
ICD10 CM	L40.8	Flexural psoriasis	696.4 1	Psoriasis vulgaris	Psoriasis vulgaris
ICD10 CM	M06.9	Rheumatoid arthritis, unspecified	714.1	Rheumatoid arthritis	RA
ICD10 CM	M08.00	Unspecified juvenile rheumatoid arthritis of unspecified site	714.2	Juvenile rheumatoid arthritis	RA
ICD10 CM	M32.10	Systemic lupus erythematosus, organ or system involvement unspecified	695.4 2	Systemic lupus erythematosus	Systemic lupus erythematosus
ICD10 CM	M34.0	Progressive systemic sclerosis	709.3	Systemic sclerosis	Systemic sclerosis
ICD10 CM	M34.1	Combination of calcinosis, Raynaud's phenomenon, esophageal dysfunction, sclerodactyly, telangiectasia	709.3	Systemic sclerosis	Systemic sclerosis
ICD10 CM	M34.2	Systemic sclerosis induced by drug and chemical	709.3	Systemic sclerosis	Systemic sclerosis
ICD10 CM	M34.81	Systemic sclerosis with lung involvement	510.1	Lung involvement in conditions classified elsewhere	Lung involvement in conditions classified elsewhere

ICD10 CM	M34.82	Systemic sclerosis with myopathy	709.3	Systemic sclerosis	Systemic sclerosis
ICD10 CM	M34.83	Systemic sclerosis with polyneuropathy	709.3	Systemic sclerosis	Systemic sclerosis
ICD10 CM	M34.89	Other systemic sclerosis	709.3	Systemic sclerosis	Systemic sclerosis
ICD10 CM	M34.9	Systemic sclerosis, unspecified	709.3	Systemic sclerosis	Systemic sclerosis
ICD10 CM	M35.00	Sicca syndrome, unspecified	709.2	Sicca syndrome	Sicca syndrome
ICD10 CM	M35.01	Sicca syndrome with keratoconjunctivitis	709.2	Sicca syndrome	Sicca syndrome
ICD10 CM	M35.02	Sicca syndrome with lung involvement	709.2	Sicca syndrome	Sicca syndrome
ICD10 CM	M35.03	Sicca syndrome with myopathy	709.2	Sicca syndrome	Sicca syndrome
ICD10 CM	M35.04	Sicca syndrome with tubulo-interstitial nephropathy	709.2	Sicca syndrome	Sicca syndrome
ICD10 CM	M35.09	Sicca syndrome with other organ involvement	709.2	Sicca syndrome	Sicca syndrome
ICD10 CM	M35.3	Polymyalgia rheumatica	717	Polymyalgia Rheumatica	Polymyalgia Rheumatica
ICD10 CM	M35.9	Systemic involvement of connective tissue, unspecified	709	Diffuse diseases of connective tissue	Diffuse diseases of connective tissue
ICD10 CM	M45.9	Ankylosing spondylitis of unspecified sites in spine	715.2	Ankylosing spondylitis	Ankylosing spondylitis
ICD10 CM	M60.9	Myositis, unspecified	770	Myalgia and myositis NOS	Myalgia and myositis NOS
ICD10 CM	M79.7	Fibromyositis	770	Myalgia and myositis NOS	Myalgia and myositis NOS

ICD10 CM	R76.8	Other specified abnormal immunological findings in serum	279.7	Other immunological findings	Other immunological findings
ICD10 CM	R76.9	Abnormal immunological finding in serum, unspecified	279.7	Other immunological findings	Other immunological findings
	99.3	Reiter's disease	711.2	Reiter's disease	Reiter's disease
	136.1	Behcet's syndrome	711.3	Behcet's syndrome	Behcet's syndrome
	242	Toxic diffuse goiter without mention of thyrotoxic crisis or storm	242	Thyrotoxicosis with or without goiter	Thyrotoxicosis with or without goiter
	242.01	Toxic diffuse goiter with mention of thyrotoxic crisis or storm	242.1	Graves' disease	Graves' disease
	242.9	Thyrotoxicosis without mention of goiter or other cause, and without mention of thyrotoxic crisis or storm	242	Thyrotoxicosis with or without goiter	Thyrotoxicosis with or without goiter
	242.91	Thyrotoxicosis without mention of goiter or other cause, with mention of thyrotoxic crisis or storm	242	Thyrotoxicosis with or without goiter	Thyrotoxicosis with or without goiter
	244.9	Unspecified acquired hypothyroidism	244.4	Hypothyroidism NOS	Hypothyroidism NOS
	255.41	Glucocorticoid deficiency	255.21	Glucocorticoid deficiency	Glucocorticoid deficiency
	258.1	Other combinations of endocrine dysfunction	259	Other endocrine disorders	Other endocrine disorders
	287.31	Immune thrombocytopenic purpura	287.31	Primary thrombocytopenia	Primary thrombocytopenia
	323.51	Encephalitis and	323	Encephalitis	Encephalitis

		encephalomyelitis following immunization procedures			
	323.61	Infectious acute disseminated encephalomyelitis (ADEM)	323	Encephalitis	Encephalitis
	323.62	Other postinfectious encephalitis and encephalomyelitis	323	Encephalitis	Encephalitis
	323.81	Other causes of encephalitis and encephalomyelitis	323.8	Encephalitis, non-infectious	Encephalitis
	323.82	Other causes of myelitis	323.8	Encephalitis, non-infectious	Encephalitis
	323.9	Unspecified causes of encephalitis, myelitis, and encephalomyelitis	323.8	Encephalitis, non-infectious	Encephalitis
	340	Multiple sclerosis	335	Multiple sclerosis	Multiple sclerosis
	341	Neuromyelitis optica	341	Other demyelinating diseases of central nervous system	Other demyelinating diseases of central nervous system
	341.8	Other demyelinating diseases of central nervous system	341	Other demyelinating diseases of central nervous system	Other demyelinating diseases of central nervous system
	341.9	Demyelinating disease of central nervous system, unspecified	341	Other demyelinating diseases of central nervous system	Other demyelinating diseases of central nervous system
	357	Acute infective	357	Inflammatory and toxic	Inflammatory

		polyneuritis		neuropathy	and toxic neuropathy
	357.1	Polyneuropathy in collagen vascular disease	357	Inflammatory and toxic neuropathy	Inflammatory and toxic neuropathy
	357.3	Polyneuropathy in malignant disease	357	Inflammatory and toxic neuropathy	Inflammatory and toxic neuropathy
	357.4	Polyneuropathy in other diseases classified elsewhere	357	Inflammatory and toxic neuropathy	Inflammatory and toxic neuropathy
	357.7	Polyneuropathy due to other toxic agents	357	Inflammatory and toxic neuropathy	Inflammatory and toxic neuropathy
	357.81	Chronic inflammatory demyelinating polyneuritis	357	Inflammatory and toxic neuropathy	Inflammatory and toxic neuropathy
	357.89	Other inflammatory and toxic neuropathy	357	Inflammatory and toxic neuropathy	Inflammatory and toxic neuropathy
	357.9	Unspecified inflammatory and toxic neuropathy	357	Inflammatory and toxic neuropathy	Inflammatory and toxic neuropathy
	358	Myasthenia gravis without (acute) exacerbation	358	Myoneural disorders	Myoneural disorders
	358.01	Myasthenia gravis with (acute) exacerbation	358.1	Myasthenia gravis	Myasthenia gravis
	359.6	Symptomatic inflammatory myopathy in diseases classified elsewhere	359.2	Myopathy	Myopathy
	390	Rheumatic fever without mention of heart involvement	41.21	Rheumatic fever / chorea	Rheumatic fever / chorea
	391	Acute rheumatic pericarditis	41.21	Rheumatic fever / chorea	Rheumatic fever / chorea

	391.1	Acute rheumatic endocarditis	420.3	Endocarditis	Endocarditis
	391.2	Acute rheumatic myocarditis	420.1	Myocarditis	Myocarditis
	391.8	Other acute rheumatic heart disease	394.4	Acute rheumatic heart disease	Acute rheumatic heart disease
	391.9	Acute rheumatic heart disease, unspecified	394.4	Acute rheumatic heart disease	Acute rheumatic heart disease
	392	Rheumatic chorea with heart involvement	41.21	Rheumatic fever / chorea	Rheumatic fever / chorea
	392.9	Rheumatic chorea without mention of heart involvement	41.21	Rheumatic fever / chorea	Rheumatic fever / chorea
	446	Polyarteritis nodosa	446	Polyarteritis nodosa and allied conditions	Polyarteritis nodosa and allied conditions
	446.1	Acute febrile mucocutaneous lymph node syndrome [MCLS]	446.2	Acute febrile mucocutaneous lymph node syndrome (Kawasaki disease)	Acute febrile mucocutaneous lymph node syndrome (Kawasaki disease)
	446.4	Wegener's granulomatosis	446.4	Wegener's granulomatosis	Wegener's granulomatosis
	446.5	Giant cell arteritis	446.5	Giant cell arteritis	Giant cell arteritis
	446.6	Thrombotic microangiopathy	446.8	Thrombotic microangiopathy	Thrombotic microangiopath y
	517.2	Lung involvement in systemic sclerosis	709.3	Systemic sclerosis	Systemic sclerosis

	517.8	Lung involvement in other diseases classified elsewhere	510	Other diseases of lung	Other diseases of lung
	580	Acute glomerulonephritis with lesion of proliferative glomerulonephritis	580	Nephritis; nephrosis; renal sclerosis	Nephritis
	580.4	Acute glomerulonephritis with lesion of rapidly progressive glomerulonephritis	580.11	Proliferative glomerulonephritis	Glomerulonephritis
	580.89	Acute glomerulonephritis with other specified pathological lesion in kidney	580.13	Acute glomerulonephritis, NOS	Glomerulonephritis
	580.9	Acute glomerulonephritis with unspecified pathological lesion in kidney	580.13	Acute glomerulonephritis, NOS	Glomerulonephritis
	582	Chronic glomerulonephritis with lesion of proliferative glomerulonephritis	580.1	Glomerulonephritis	Glomerulonephritis
	582.1	Chronic glomerulonephritis with lesion of membranous glomerulonephritis	580.12	Non-proliferative glomerulonephritis	Glomerulonephritis
	582.2	Chronic glomerulonephritis with lesion of membranoproliferative glomerulonephritis	580.11	Proliferative glomerulonephritis	Glomerulonephritis
	582.4	Chronic glomerulonephritis with lesion of rapidly progressive glomerulonephritis	580.11	Proliferative glomerulonephritis	Glomerulonephritis
	582.89	Chronic glomerulonephritis with other specified pathological lesion in kidney	580.14	Chronic glomerulonephritis, NOS	Glomerulonephritis

	582.9	Chronic glomerulonephritis with unspecified pathological lesion in kidney	580.14	Chronic glomerulonephritis, NOS	Glomerulonephritis
	583	Nephritis and nephropathy, not specified as acute or chronic, with lesion of proliferative glomerulonephritis	580	Nephritis; nephrosis; renal sclerosis	Nephritis
	583.1	Nephritis and nephropathy, not specified as acute or chronic, with lesion of membranous glomerulonephritis	580.12	Non-proliferative glomerulonephritis	Glomerulonephritis
	583.2	Nephritis and nephropathy, not specified as acute or chronic, with lesion of membranoproliferative glomerulonephritis	580.11	Proliferative glomerulonephritis	Glomerulonephritis
	583.81	Nephritis and nephropathy, not specified as acute or chronic, in diseases classified elsewhere	580.31	Nephritis and nephropathy in diseases classified elsewhere	Nephritis
	583.89	Nephritis and nephropathy, not specified as acute or chronic, with other specified pathological lesion in kidney	580.32	Nephritis and nephropathy with pathological lesion	Nephritis
	694	Dermatitis herpetiformis	695.2	Bullous dermatoses	Bullous dermatoses
	694.2	Juvenile dermatitis herpetiformis	695.21	Dermatitis herpetiformis	Dermatitis herpetiformis
	694.3	Impetigo herpetiformis	695.21	Dermatitis herpetiformis	Dermatitis herpetiformis
	694.4	Pemphigus	695.22	Pemphigus and pemphigoid	Pemphigus and pemphigoid

	694.5	Pemphigoid	695.22	Pemphigus and pemphigoid	Pemphigus and pemphigoid
	694.6	Benign mucous membrane pemphigoid without mention of ocular involvement	695.22	Pemphigus and pemphigoid	Pemphigus and pemphigoid
	695.15	Toxic epidermal necrolysis	695.1	Toxic erythema	Toxic erythema
	704	Alopecia, unspecified	704	Diseases of hair and hair follicles	Diseases of hair and hair follicles
	704.09	Other alopecia	704.1	Alopecia	Alopecia
	709.01	Vitiligo	694.1	Vitiligo	Vitiligo
	710.3	Dermatomyositis	709.5	Dermatomyositis	Dermatomyositis
	710.4	Polymyositis	709.4	Polymyositis	Polymyositis
	710.8	Other specified diffuse diseases of connective tissue	709.6	Other specified diffuse diseases of connective tissue	Other specified diffuse diseases of connective tissue
	711.4	Arthropathy associated with other bacterial diseases, site unspecified	711	Arthropathy associated with infections	Arthropathy
	711.41	Arthropathy associated with other bacterial diseases, shoulder region	711	Arthropathy associated with infections	Arthropathy
	711.42	Arthropathy associated with other bacterial diseases, upper arm	711	Arthropathy associated with infections	Arthropathy
	711.43	Arthropathy associated with other bacterial diseases, forearm	711	Arthropathy associated with infections	Arthropathy

	711.44	Arthropathy associated with other bacterial diseases, hand	711	Arthropathy associated with infections	Arthropathy
	711.45	Arthropathy associated with other bacterial diseases, pelvic region and thigh	711	Arthropathy associated with infections	Arthropathy
	711.46	Arthropathy associated with other bacterial diseases, lower leg	711	Arthropathy associated with infections	Arthropathy
	711.47	Arthropathy associated with other bacterial diseases, ankle and foot	711	Arthropathy associated with infections	Arthropathy
	711.48	Arthropathy associated with other bacterial diseases, other specified sites	711	Arthropathy associated with infections	Arthropathy
	711.49	Arthropathy associated with other bacterial diseases, multiple sites	711	Arthropathy associated with infections	Arthropathy
	713.7	Other general diseases with articular involvement	713	Arthropathy associated with other disorders classified elsewhere	Arthropathy
	714.1	Felty's syndrome	714.1	Rheumatoid arthritis	RA
	714.2	Other rheumatoid arthritis with visceral or systemic involvement	714.1	Rheumatoid arthritis	RA
	714.32	Pauciarticular juvenile rheumatoid arthritis	714.2	Juvenile rheumatoid arthritis	RA
	714.81	Rheumatoid lung	714.1	Rheumatoid arthritis	RA
	714.89	Other specified inflammatory polyarthropathies	714	Rheumatoid arthritis and other inflammatory polyarthropathies	RA

	M02.30	Reiter's disease	711.2	Reiter's disease	Reiter's disease
	D86.0	Sarcoidosis	697	Sarcoidosis	Sarcoidosis
	M35.2	Behcet's syndrome	711.3	Behcet's syndrome	Behcet's syndrome
	E05.00	Toxic diffuse goiter without mention of thyrotoxic crisis or storm	242	Thyrotoxicosis with or without goiter	Thyrotoxicosis with or without goiter
	E05.01	Toxic diffuse goiter with mention of thyrotoxic crisis or storm	242.1	Graves' disease	Graves' disease
	E05.90	Thyrotoxicosis without mention of goiter or other cause, and without mention of thyrotoxic crisis or storm	242	Thyrotoxicosis with or without goiter	Thyrotoxicosis with or without goiter
	E05.91	Thyrotoxicosis without mention of goiter or other cause, with mention of thyrotoxic crisis or storm	242	Thyrotoxicosis with or without goiter	Thyrotoxicosis with or without goiter
	E03.9	Unspecified acquired hypothyroidism	244.4	Hypothyroidism NOS	Hypothyroidis m NOS
	E10.11	Diabetes with other coma, type I [juvenile type], not stated as uncontrolled	250.1	Type 1 diabetes	DM
	E10.61 8	Diabetes with other specified manifestations, type I [juvenile type], not stated as uncontrolled	250.1	Type 1 diabetes	DM
	E27.1	Glucocorticoid deficiency	255.2 1	Glucocorticoid deficiency	Glucocorticoid deficiency
	E31.0	Other combinations of endocrine dysfunction	259	Other endocrine disorders	Other endocrine disorders
	D69.3	Immune thrombocytopenic purpura	287.3 1	Primary thrombocytopenia	Primary thrombocytope

					nia
	G04.02	Encephalitis and encephalomyelitis following immunization procedures	323	Encephalitis	Encephalitis
	G04.00	Infectious acute disseminated encephalomyelitis (ADEM)	323	Encephalitis	Encephalitis
	G04.30	Other postinfectious encephalitis and encephalomyelitis	323	Encephalitis	Encephalitis
	G04.81	Other causes of encephalitis and encephalomyelitis	323.8	Encephalitis, non-infectious	Encephalitis
	G04.89	Other causes of myelitis	323.8	Encephalitis, non-infectious	Encephalitis
	G04.90	Unspecified causes of encephalitis, myelitis, and encephalomyelitis	323.8	Encephalitis, non-infectious	Encephalitis
	G35	Multiple sclerosis	335	Multiple sclerosis	Multiple sclerosis
	G36.0	Neuromyelitis optica	341	Other demyelinating diseases of central nervous system	Other demyelinating diseases of central nervous system
	G36.1	Other demyelinating diseases of central nervous system	341	Other demyelinating diseases of central nervous system	Other demyelinating diseases of central nervous system
	G36.9	Demyelinating disease of central nervous system, unspecified	341	Other demyelinating diseases of central nervous system	Other demyelinating diseases of central nervous

					system
	G04.1	Tropical spastic paraplegia	344	Other paralytic syndromes	Other paralytic syndromes
	G61.0	Acute infective polyneuritis	357	Inflammatory and toxic neuropathy	Inflammatory and toxic neuropathy
	M05.50	Polyneuropathy in collagen vascular disease	357	Inflammatory and toxic neuropathy	Inflammatory and toxic neuropathy
	G13.1	Polyneuropathy in malignant disease	357	Inflammatory and toxic neuropathy	Inflammatory and toxic neuropathy
	G61.1	Polyneuropathy due to other toxic agents	357	Inflammatory and toxic neuropathy	Inflammatory and toxic neuropathy
	G61.81	Chronic inflammatory demyelinating polyneuritis	357	Inflammatory and toxic neuropathy	Inflammatory and toxic neuropathy
	G61.89	Other inflammatory and toxic neuropathy	357	Inflammatory and toxic neuropathy	Inflammatory and toxic neuropathy
	G61.9	Unspecified inflammatory and toxic neuropathy	357	Inflammatory and toxic neuropathy	Inflammatory and toxic neuropathy
	G70.00	Myasthenia gravis without (acute) exacerbation	358	Myoneural disorders	Myoneural disorders
	G70.01	Myasthenia gravis with (acute) exacerbation	358.1	Myasthenia gravis	Myasthenia gravis
	M05.40	Symptomatic inflammatory myopathy in diseases classified elsewhere	359.2	Myopathy	Myopathy
	I00	Rheumatic fever without mention of heart involvement	41.21	Rheumatic fever / chorea	Rheumatic fever / chorea

	I01.0	Acute rheumatic pericarditis	41.21	Rheumatic fever / chorea	Rheumatic fever / chorea
	I01.1	Acute rheumatic endocarditis	420.3	Endocarditis	Endocarditis
	I01.2	Acute rheumatic myocarditis	420.1	Myocarditis	Myocarditis
	I01.8	Other acute rheumatic heart disease	394.4	Acute rheumatic heart disease	Acute rheumatic heart disease
	I01.9	Acute rheumatic heart disease, unspecified	394.4	Acute rheumatic heart disease	Acute rheumatic heart disease
	I02.0	Rheumatic chorea with heart involvement	41.21	Rheumatic fever / chorea	Rheumatic fever / chorea
	I02.9	Rheumatic chorea without mention of heart involvement	41.21	Rheumatic fever / chorea	Rheumatic fever / chorea
	M32.12	Pericarditis in systemic lupus erythematosus	420.21	Acute pericarditis	Acute pericarditis
	M32.11	Endocarditis in systemic lupus erythematosus	395	Heart valve disorders	Heart valve disorders
	M30.0	Polyarteritis nodosa	446	Polyarteritis nodosa and allied conditions	Polyarteritis nodosa and allied conditions
	M30.3	Acute febrile mucocutaneous lymph node syndrome [MCLS]	446.2	Acute febrile mucocutaneous lymph node syndrome (Kawasaki disease)	Acute febrile mucocutaneous lymph node syndrome (Kawasaki disease)
	M30.1	Wegener's granulomatosis	446.4	Wegener's granulomatosis	Wegener's granulomatosis
	M31.5	Giant cell arteritis	446.5	Giant cell arteritis	Giant cell arteritis

	M31.1	Thrombotic microangiopathy	446.8	Thrombotic microangiopathy	Thrombotic microangiopathy
	M32.13	Lung involvement in other diseases classified elsewhere	510	Other diseases of lung	Other diseases of lung
	N00.0	Acute glomerulonephritis with lesion of proliferative glomerulonephritis	580	Nephritis; nephrosis; renal sclerosis	Nephritis
	N01.0	Acute glomerulonephritis with lesion of rapidly progressive glomerulonephritis	580.11	Proliferative glomerulonephritis	Glomerulonephritis
	N00.8	Acute glomerulonephritis with other specified pathological lesion in kidney	580.13	Acute glomerulonephritis, NOS	Glomerulonephritis
	N00.9	Acute glomerulonephritis with unspecified pathological lesion in kidney	580.13	Acute glomerulonephritis, NOS	Glomerulonephritis
	N03.2	Chronic glomerulonephritis with lesion of proliferative glomerulonephritis	580.1	Glomerulonephritis	Glomerulonephritis
	N03.1	Chronic glomerulonephritis with lesion of membranous glomerulonephritis	580.12	Non-proliferative glomerulonephritis	Glomerulonephritis
	N03.4	Chronic glomerulonephritis with lesion of membranoproliferative glomerulonephritis	580.11	Proliferative glomerulonephritis	Glomerulonephritis
	N03.8	Chronic glomerulonephritis with lesion of rapidly progressive glomerulonephritis	580.11	Proliferative glomerulonephritis	Glomerulonephritis
	N03.0	Chronic glomerulonephritis	580.1	Chronic	Glomeruloneph

		with other specified pathological lesion in kidney	4	glomerulonephritis, NOS	ritis
	N03.9	Chronic glomerulonephritis with unspecified pathological lesion in kidney	580.14	Chronic glomerulonephritis, NOS	Glomerulonephritis
	N05.9	Nephritis and nephropathy, not specified as acute or chronic, with lesion of proliferative glomerulonephritis	580	Nephritis; nephrosis; renal sclerosis	Nephritis
	N05.2	Nephritis and nephropathy, not specified as acute or chronic, with lesion of membranous glomerulonephritis	580.12	Non-proliferative glomerulonephritis	Glomerulonephritis
	N05.3	Nephritis and nephropathy, not specified as acute or chronic, with lesion of membranoproliferative glomerulonephritis	580.11	Proliferative glomerulonephritis	Glomerulonephritis
	M32.14	Nephritis and nephropathy, not specified as acute or chronic, in diseases classified elsewhere	580.31	Nephritis and nephropathy in diseases classified elsewhere	Nephritis
	N05.0	Nephritis and nephropathy, not specified as acute or chronic, with other specified pathological lesion in kidney	580.32	Nephritis and nephropathy with pathological lesion	Nephritis
	L13.0	Dermatitis herpetiformis	695.2	Bullous dermatoses	Bullous dermatoses
	L12.2	Juvenile dermatitis herpetiformis	695.21	Dermatitis herpetiformis	Dermatitis herpetiformis
	L10.0	Pemphigus	695.22	Pemphigus and pemphigoid	Pemphigus and pemphigoid

	L12.0	Pemphigoid	695.22	Pemphigus and pemphigoid	Pemphigus and pemphigoid
	L12.1	Benign mucous membrane pemphigoid without mention of ocular involvement	695.22	Pemphigus and pemphigoid	Pemphigus and pemphigoid
	L12.30	Toxic epidermal necrolysis	695.1	Toxic erythema	Toxic erythema
	L64.9	Alopecia, unspecified	704	Diseases of hair and hair follicles	Diseases of hair and hair follicles
	L64.0	Other alopecia	704.1	Alopecia	Alopecia
	L80	Vitiligo	694.1	Vitiligo	Vitiligo
	M32.0	Systemic lupus erythematosus	709	Diffuse diseases of connective tissue	Diffuse diseases of connective tissue
	M33.00	Dermatomyositis	709.5	Dermatomyositis	Dermatomyositis
	M33.20	Polymyositis	709.4	Polymyositis	Polymyositis
	M35.1	Other specified diffuse diseases of connective tissue	709.6	Other specified diffuse diseases of connective tissue	Other specified diffuse diseases of connective tissue
	M02.80	Arthropathy associated with other bacterial diseases, site unspecified	711	Arthropathy associated with infections	Arthropathy
	M02.811	Arthropathy associated with other bacterial diseases, shoulder region	711	Arthropathy associated with infections	Arthropathy
	M02.821	Arthropathy associated with other bacterial diseases, upper arm	711	Arthropathy associated with infections	Arthropathy

	M02.83 1	Arthropathy associated with other bacterial diseases, forearm	711	Arthropathy associated with infections	Arthropathy
	M02.84 1	Arthropathy associated with other bacterial diseases, hand	711	Arthropathy associated with infections	Arthropathy
	M02.85 1	Arthropathy associated with other bacterial diseases, pelvic region and thigh	711	Arthropathy associated with infections	Arthropathy
	M02.86 1	Arthropathy associated with other bacterial diseases, lower leg	711	Arthropathy associated with infections	Arthropathy
	M02.87 1	Arthropathy associated with other bacterial diseases, ankle and foot	711	Arthropathy associated with infections	Arthropathy
	M02.88	Arthropathy associated with other bacterial diseases, other specified sites	711	Arthropathy associated with infections	Arthropathy
	M02.89	Arthropathy associated with other bacterial diseases, multiple sites	711	Arthropathy associated with infections	Arthropathy
	M02.9	Other general diseases with articular involvement	713	Arthropathy associated with other disorders classified elsewhere	Arthropathy
	M05.70	Rheumatoid arthritis	714	Rheumatoid arthritis and other inflammatory polyarthropathies	RA
	M05.00	Felty's syndrome	714.1	Rheumatoid arthritis	RA
	M05.20	Other rheumatoid arthritis with visceral or systemic involvement	714.1	Rheumatoid arthritis	RA
	M08.40	Pauciarticular juvenile rheumatoid arthritis	714.2	Juvenile rheumatoid arthritis	RA

	M05.10	Rheumatoid lung	714.1	Rheumatoid arthritis	RA
	M06.4	Other specified inflammatory polyarthropathies	714	Rheumatoid arthritis and other inflammatory polyarthropathies	RA
	M08.1	Ankylosing spondylitis	715	Other inflammatory spondylopathies	Other inflammatory spondylopathies
	E03.5	Myxedema coma	348.7	Coma	Coma

Table S8. Patient characteristics of the immunotherapy and chemotherapy groups.

Melanoma

Feature	Nivolumab.N %		Ipilimumab.N %		Pembrolizumab.N %		all.immunotherapy.drugs.N %		chemo.N %	
All	1148	100.00	871	100.00	626	100.00	2657	100.00	1118	100.00
F	371	32.32	285	32.72	212	33.87	872	32.82	420	37.57
M	777	67.68	586	67.28	414	66.13	1785	67.18	698	62.43
Sex p value	p=0.0114		p=0.0313		p=0.1371		p=0.0061		Control Group	
1	124	10.80	83	9.53	26	4.15	233	8.77	69	6.17
2	133	11.59	123	14.12	56	8.95	313	11.78	128	11.45
3	257	22.39	232	26.64	110	17.57	601	22.62	308	27.55
4	308	26.83	231	26.52	164	26.20	705	26.53	291	26.03
5	215	18.73	139	15.96	156	24.92	514	19.35	217	19.41
6	111	9.67	63	7.23	114	18.21	291	10.95	105	9.39
Age p value	p=9e-04		p=0.0068		p<1e-04		p=0.0048		Control Group	
Matched	438	38.15	340	39.04	252	40.26	1035	38.95	Control Group	

Renal

Feature	Pembrolizumab.N %		Nivolumab.N %		all.immunotherapy.drugs.N %		chemo.N %	
All	214	100.00	1003	100.00	1284	100.00	1425	100.00
F	71	33.18	249	24.83	336	26.17	523	36.70
M	143	66.82	754	75.17	948	73.83	902	63.30
Gender.pvalue	p=0.3205		p<1e-04		p<1e-04		Control Group	
[0,40)	<5	2.33	29	2.89	34	2.65	195	13.68
[40,50)	7	3.26	76	7.58	86	6.70	109	7.65
[50,60)	29	13.49	256	25.52	295	22.98	301	21.12
[60,70)	72	33.49	358	35.69	449	34.97	413	28.98
[70,80)	64	29.77	217	21.64	304	23.68	291	20.42
[80,120)	38	17.67	67	6.68	116	9.03	116	8.14
Age.pvalue	p<1e-04		p<1e-04		p<1e-04		Control Group	
Matched	120	56.07	552	55.03	709	55.22	Control Group	

Head and Neck

Feature	Nivolumab.N %		Pembrolizumab.N %		all.immunotherapy.drugs.N %		chemo.N %	
All	356	100.00	445	100.00	857	100.00	10082	100.00
F	72	20.22	98	22.02	182	21.24	2006	19.90
M	284	79.78	347	77.98	675	78.76	8076	80.10
Gender.pvalue	p=0.8942		p=0.2676		p=0.3482		Control Group	
[0,40)	11	3.09	22	4.94	36	4.20	521	5.17

[40,50)	26	7.30	32	7.19	62	7.23	1313	13.03
[50,60)	97	27.25	112	25.17	218	25.44	3457	34.30
[60,70)	117	32.87	136	30.56	276	32.21	3127	31.02
[70,80)	76	21.35	91	20.45	181	21.12	1247	12.37
[80,120)	29	8.15	52	11.69	84	9.80	415	4.12
Age.pvalue	p<1e-04		p<1e-04		p<1e-04		Control Group	
Matched	312	87.64	371	83.37	731	85.30	Control Group	

Squamous

Feature	Nivolumab.N %		Pembrolizumab.N %		all.immunotherapy.drugs.N %		chemo.N %	
All	231	100.00	295	100.00	602	100.00	1968	100.00
F	51	22.08	61	20.68	121	20.10	606	30.79
M	180	77.92	234	79.32	481	79.90	1362	69.21
Gender.pvalue	p=0.0065		p=5e-04		p<1e-04		Control Group	
[0,40)	<5	2.13	6	2.03	8	1.33	27	1.37
[40,50)	11	4.68	7	2.37	19	3.16	130	6.61
[50,60)	35	14.89	31	10.51	76	12.62	425	21.60
[60,70)	67	28.51	70	23.73	151	25.08	557	28.30
[70,80)	65	27.66	85	28.81	175	29.07	496	25.20
[80,120)	52	22.13	96	32.54	173	28.74	333	16.92
Age.pvalue	p=0.0662		p<1e-04		p<1e-04		Control Group	
Matched	159	68.83	197	66.78	391	64.95	Control Group	

Basal

Feature	Nivolumab.N %		Pembrolizumab.N %		Ipilimumab.N %		all.immunotherapy.drugs.N %		chemo.N %	
All	296	100.00	289	100.00	71	100.00	695	100.00	899	100.00
F	66	22.30	71	24.57	9	12.68	156	22.45	348	38.71
M	230	77.70	218	75.43	62	87.32	539	77.55	551	61.29
Gender.pvalue	p<1e-04		p<1e-04		p<1e-04		p<1e-04		Control Group	
[0,40)	<5	1.68	<5	1.69	<5	6.33	<5	0.72	23	2.56
[40,50)	12	4.04	<5	1.69	<5	6.33	18	2.59	71	7.90
[50,60)	51	17.17	19	6.44	16	20.25	91	13.07	202	22.47
[60,70)	80	26.94	68	23.05	20	25.32	175	25.14	229	25.47
[70,80)	92	30.98	104	35.25	19	24.05	231	33.19	233	25.92
[80,120)	57	19.19	94	31.86	14	17.72	176	25.29	141	15.68
Age.pvalue	p=0.0308		p<1e-04		p=0.5169		p<1e-04		Control Group	
Matched	127	42.91	139	48.10	31	43.66	279	40.14	Control Group	

Brain

Feature	Pembrolizumab.N %		Nivolumab.N %		Ipilimumab.N %		all.immunotherapy.drugs.N %		chemo.N %	
All	162	100.00	176	100.00	54	100.00	407	100.00	5144	100.00

F	65	40.12	65	36.93	14	25.93	152	37.35	2110	41.02
M	97	59.88	111	63.07	40	74.07	255	62.65	3034	58.98
Gender.pvalue	p=0.8717		p=0.3078		p=0.0277		p=0.1612		Control Group	
[0,40)	16	9.88	22	12.36	<5	8.33	42	10.32	1492	29.03
[40,50)	25	15.43	18	10.11	6	10.00	50	12.29	862	16.77
[50,60)	40	24.69	47	26.40	23	38.33	117	28.75	1401	27.26
[60,70)	50	30.86	59	33.15	10	16.67	124	30.47	1018	19.81
[70,80)	20	12.35	27	15.17	11	18.33	59	14.50	314	6.11
[80,120)	11	6.79	<5	2.81	<5	8.33	15	3.69	53	1.03
Age.pvalue	p<1e-04		p<1e-04		p<1e-04		p<1e-04		Control Group	
Matched	118	72.84	138	78.41	39	72.22	307	75.43	Control Group	

Lung

Feature	Pembrolizumab.N	%	Nivolumab.N	%	Atezolizumab.N	%	Durvalumab.N	%	all.immunotherapy.drugs.N	%	chemo.N	%
All	2913	100.00	2424	100.00	574	100.00	203	100.00	6157	100.00	26502	100.00
F	1251	42.95	1018	42.00	258	44.95	89	43.84	2630	42.72	11552	43.59
M	1662	57.05	1406	58.00	316	55.05	114	56.16	3527	57.28	14950	56.41
Gender.pvalue	p=0.5153		p=0.136		p=0.5277		p=0.9426		p=0.2109		Control Group	
[0,40)	34	1.17	22	0.91	<5	0.87	<5	2.40	60	0.97	357	1.35
[40,50)	127	4.36	94	3.88	25	4.34	6	2.88	256	4.16	1607	6.06
[50,60)	520	17.85	511	21.08	119	20.66	39	18.75	1205	19.57	6388	24.10
[60,70)	1012	34.74	859	35.44	212	36.81	86	41.35	2179	35.39	9198	34.71
[70,80)	873	29.97	698	28.80	164	28.47	63	30.29	1808	29.36	6737	25.42
[80,120)	347	11.91	240	9.90	51	8.85	9	4.33	649	10.54	2215	8.36
Age.pvalue	p<1e-04		p<1e-04		p=0.087		p=0.007		p<1e-04		Control Group	
Matched	2773	95.19	2279	94.02	545	94.95	199	98.03	5834	94.75	Control Group	

Table S9. Patient characteristics of the immunotherapy and targeted therapy groups.

Renal

Feature	Pembrolizumab.N	%	Nivolumab.N	%	Atezolizumab.N	%	all.immunotherapy.drugs.N	%	target.N	%
All	214	100.00	1002	100.00	52	100.00	1284	100.00	1868	100.00
F	71	33.18	248	24.75	12	23.08	335	26.09	486	26.02
M	143	66.82	754	75.25	40	76.92	949	73.91	1382	73.98
Gender.pvalue	p=0.0275		p=0.4718		p=0.7444		p=0.9692		Control Group	
[0,40)	<5	2.33	29	2.89	<5	8.47	34	2.65	56	3.00
[40,50)	7	3.26	76	7.58	<5	8.47	86	6.70	181	9.69
[50,60)	29	13.49	256	25.55	6	10.17	295	22.98	540	28.91
[60,70)	72	33.49	357	35.63	15	25.42	449	34.97	637	34.10
[70,80)	64	29.77	217	21.66	19	32.20	304	23.68	301	16.11
[80,120)	38	17.67	67	6.69	9	15.25	116	9.03	153	8.19
Age.pvalue	p<1e-04		p=0.0013		p=3e-04		p<1e-04		Control Group	
Matched	120	56.07	606	60.48	24	46.15	749	58.33	Control Group	

Head and Neck

Feature	Nivolumab.N	%	Pembrolizumab.N	%	all.immunotherapy.drugs.N	%	target.N	%
All	358	100.00	444	100.00	858	100.00	2455	100.00
F	72	20.11	97	21.85	181	21.10	433	17.64
M	286	79.89	347	78.15	677	78.90	2022	82.36
Gender.pvalue	p=0.2669		p=0.0385		p=0.028		Control Group	
[0,40)	12	3.35	22	4.95	37	4.31	54	2.20
[40,50)	26	7.26	32	7.21	62	7.23	251	10.22
[50,60)	98	27.37	111	25.00	218	25.41	714	29.08
[60,70)	117	32.68	136	30.63	276	32.17	773	31.49
[70,80)	76	21.23	91	20.50	181	21.10	402	16.37
[80,120)	29	8.10	52	11.71	84	9.79	261	10.63
Age.pvalue	p=0.0446		p=0.0013		p<1e-04		Control Group	
Matched	247	68.99	308	69.37	595	69.35	Control Group	

Squamous

Feature	Nivolumab.N	%	Pembrolizumab.N	%	all.immunotherapy.drugs.N	%	target.N	%
All	231	100.00	295	100.00	602	100.00	366	100.00
F	51	22.08	61	20.68	121	20.10	57	15.57
M	180	77.92	234	79.32	481	79.90	309	84.43
Gender.pvalue	p=0.0482		p=0.096		p=0.0895		Control Group	
[0,40)	<5	2.13	6	2.03	8	1.33	10	2.73
[40,50)	11	4.68	7	2.37	19	3.16	21	5.74
[50,60)	35	14.89	31	10.51	76	12.62	81	22.13

[60,70)	67	28.51	70	23.73	151	25.08	99	27.05
[70,80)	65	27.66	85	28.81	175	29.07	80	21.86
[80,120)	52	22.13	96	32.54	173	28.74	75	20.49
Age.pvalue	p=0.2467		p<1e-04		p<1e-04		Control Group	
Matched	59	25.54	79	26.78	148	24.58	Control Group	

Brain

Feature	Nivolumab.N %		Pembrolizumab.N %		Ipilimumab.N %		all.immunotherapy.drugs.N %		target.N %	
All	162	100.00	177	100.00	54	100.00	408	100.00	2814	100.00
F	65	40.12	65	36.72	14	25.93	152	37.25	1086	38.59
M	97	59.88	112	63.28	40	74.07	256	62.75	1728	61.41
Gender.pvalue	p=0.7437		p=0.6334		p=0.0688		p=0.6237		Control Group	
African American/Black	<5	2.48	<5	2.33	<5	5.15	<5	1.13	19	0.67
American Indian/Alaskan Native	<5	2.48	<5	2.33	<5	5.15	<5	1.13	<5	0.18
Asian	<5	2.48	<5	2.33	<5	5.15	<5	1.13	22	0.78
Black (Non-Hispanic)	<5	2.48	<5	2.33	<5	5.15	<5	1.13	<5	0.18
Caucasian (Non-Hispanic)	<5	2.48	<5	2.33	<5	5.15	<5	1.13	<5	0.18
Hispanic/Latino	<5	2.48	<5	2.33	<5	5.15	<5	1.13	19	0.67
Other	<5	2.48	<5	2.33	<5	5.15	<5	1.13	8	0.28
Pacific Islander	<5	2.48	<5	2.33	<5	5.15	<5	1.13	<5	0.18
Two or more races	<5	2.48	<5	2.33	<5	5.15	10	2.25	31	1.10
White	18	8.91	20	9.30	12	12.37	50	11.26	374	13.21
Unknown	139	68.81	150	69.77	40	41.24	344	77.48	2338	82.59
Ethnicity.pvalue	p=0.3882		p=0.2436		p=0.3604		p=0.1247		Control Group	
[0,40)	16	9.88	23	12.85	<5	8.33	43	10.54	465	16.52
[40,50)	25	15.43	18	10.06	6	10.00	50	12.25	515	18.30
[50,60)	40	24.69	47	26.26	23	38.33	117	28.68	882	31.34
[60,70)	50	30.86	59	32.96	10	16.67	124	30.39	671	23.85
[70,80)	20	12.35	27	15.08	11	18.33	59	14.46	242	8.60
[80,120)	11	6.79	<5	2.79	<5	8.33	15	3.68	39	1.39
Age.pvalue	p<1e-04		p=2e-04		p=2e-04		p<1e-04		Control Group	
Matched	95	58.64	100	56.50	30	55.56	235		Control Group	

Lung

Feature	Nivolumab.N %		Pembrolizumab.N %		Atezolizumab.N %		Durvalumab.N %		all.immunotherapy.drugs.N %		target.N %	
All	2919	100.00	2435	100.00	574	100.00	204	100.00	6175	100.00	6022	100.00
F	1255	42.99	1022	41.97	258	44.95	89	43.63	2638	42.72	2925	48.57
M	1664	57.01	1413	58.03	316	55.05	115	56.37	3537	57.28	3097	51.43
Gender.pvalue	p<1e-04		p<1e-04		p=0.1133		p=0.1768		p<1e-04		Control Group	
[0,40)	34	1.16	22	0.90	<5	0.87	<5	2.39	60	0.97	133	2.21
[40,50)	128	4.39	94	3.86	25	4.34	6	2.87	257	4.16	589	9.78

[50,60)	521	17.85	516	21.19	119	20.66	39	18.66	1211	19.61	1700	28.23
[60,70)	1016	34.81	865	35.52	212	36.81	87	41.63	2190	35.47	1898	31.52
[70,80)	873	29.91	698	28.67	164	28.47	63	30.14	1808	29.28	1212	20.13
[80,120)	347	11.89	240	9.86	51	8.85	9	4.31	649	10.51	490	8.14
Age.pvalue	p<1e-04		p<1e-04		p<1e-04		p<1e-04		p<1e-04		Control Group	
Matched	2441	83.62	2020	82.96	478	83.28	186		5201		Control Group	

Table S10. The cumulative incidence rate (%) of autoimmune diseases of the immunotherapy and the chemotherapy groups.

Melanoma

	matched					unmatched				
time	immunotherapy	chemotherapy	Hazard-Ratio	Log-rank test p value	Immuno.N	immunotherapy	chemotherapy	Hazard-Ratio	Log-rank test p value	Immuno.N
Pembrolizumab										
month=3	6.03[2.91, 9.05]	1.04[0, 2.34]	5.79[1.27, 39.43]	5.86E-05	252	5.09[3.26, 6.88]	2.01[1.13, 2.88]	2.53[1.24, 5.31]	7.37E-11	626
month=6	14.16[9.29, 18.76]	5.23[2, 8.36]	2.71[1.18, 12.55]	5.86E-05	252	12.4[9.45, 15.24]	4.21[2.85, 5.54]	2.95[1.94, 4.94]	7.37E-11	626
month=9	17.52[12.09, 22.62]	8.15[3.8, 12.3]	2.15[1.05, 6.51]	5.86E-05	252	16.62[13.13, 19.98]	7.01[5.11, 8.88]	2.37[1.74, 3.55]	7.37E-11	626
month=12	21.01[14.93, 26.66]	9.68[4.75, 14.35]	2.17[1.06, 5.66]	5.86E-05	252	22.56[18.31, 26.59]	9.53[7.19, 11.81]	2.37[1.74, 3.24]	7.37E-11	626
month=15	25.42[18.42, 31.82]	11.35[5.78, 16.59]	2.24[1.13, 5.7]	5.86E-05	252	26.14[21.43, 30.57]	10.86[8.27, 13.38]	2.41[1.83, 3.31]	7.37E-11	626
Nivolumab										
month=3	10.21[7.15, 13.17]	2.48[0.94, 3.99]	4.12[1.86, 14.8]	1.27E-23	438	11.85[9.85, 13.81]	2.01[1.13, 2.88]	5.9[3.73, 11.39]	0.00E+00	1148
month=6	20.52[16.14, 24.67]	4.62[2.4, 6.8]	4.44[2.3, 9.35]	1.27E-23	438	20.6[17.95, 23.16]	4.21[2.85, 5.54]	4.9[3.42, 7.07]	0.00E+00	1148
month=9	28.4[23.16, 33.28]	7.52[4.34, 10.59]	3.78[2.13, 7.67]	1.27E-23	438	26.75[23.68, 29.71]	7.01[5.11, 8.88]	3.82[2.95, 5.02]	0.00E+00	1148
month=12	31.03[25.44, 36.19]	7.66[4.44, 10.77]	4.05[2.28, 8.05]	1.27E-23	438	30.64[27.26, 33.87]	9.53[7.19, 11.81]	3.22[2.55, 4.05]	0.00E+00	1148
month=15	37.7[31.11, 43.65]	7.82[4.54, 11]	4.82[2.72, 9.19]	1.27E-23	438	34.74[30.96, 38.32]	10.86[8.27, 13.38]	3.2[2.59, 3.98]	0.00E+00	1148
Ipilimumab										
month=3	11.77[8.01, 15.38]	2.09[0.46, 3.68]	5.65[2.19, 26.42]	5.88E-09	340	10.09[7.93, 12.19]	2.01[1.13, 2.88]	5.02[3.49, 8.18]	0.00E+00	871
month=6	18.75[13.96, 23.28]	6.4[3.27, 9.42]	2.93[1.64, 8.56]	5.88E-09	340	17.99[15.08, 20.8]	4.21[2.85, 5.54]	4.28[3.06, 6.25]	0.00E+00	871
month=9	21.39[16.18, 26.28]	7.42[3.98, 10.73]	2.88[1.59, 7.3]	5.88E-09	340	22.37[19.06, 25.54]	7.01[5.11, 8.88]	3.19[2.3, 4.27]	0.00E+00	871
month=12	25.83[19.86, 31.35]	10.19[5.73, 14.44]	2.53[1.42, 5.64]	5.88E-09	340	27.53[23.76, 31.12]	9.53[7.19, 11.81]	2.89[2.2, 3.93]	0.00E+00	871
month=15	29.69[23.03, 35.77]	13.57[8.01, 18.8]	2.19[1.29, 4.9]	5.88E-09	340	29.79[25.8, 33.57]	10.86[8.27, 13.38]	2.74[2.06, 3.74]	0.00E+00	871
all immunotherapy drugs										
month=3	8.99[7.12, 10.82]	2.69[1.64, 3.72]	3.34[1.83, 8.55]	3.14E-27	1035	9.62[8.43, 10.8]	2.01[1.13, 2.88]	4.79[3.16, 7.92]	0.00E+00	2657
month=6	17.64[15, 20.2]	5.27[3.71, 6.8]	3.35[2.16, 5.99]	3.14E-27	1035	17.72[16.08, 19.32]	4.21[2.85, 5.54]	4.21[3.16, 6.39]	0.00E+00	2657
month=9	22.65[19.6, 25.57]	8.19[6.06, 10.28]	2.76[1.9, 4.64]	3.14E-27	1035	22.81[20.91, 24.66]	7.01[5.11, 8.88]	3.25[2.67, 4.54]	0.00E+00	2657
month=12	26.48[23.1, 29.7]	9.34[6.99, 11.63]	2.83[1.9, 4.45]	3.14E-27	1035	27.62[25.47, 29.7]	9.53[7.19, 11.81]	2.9[2.44, 3.97]	0.00E+00	2657
month=15	31.49[27.65, 35.13]	10.62[8.01, 13.15]	2.96[1.98, 4.58]	3.14E-27	1035	30.92[28.58, 33.18]	10.86[8.27, 13.38]	2.85[2.37, 3.75]	0.00E+00	2657

Renal

	matched					unmatched				
time	immunotherapy	chemotherapy	Hazard-Ratio	Log-rank test p value	Immuno.N	immunotherapy	chemotherapy	Hazard-Ratio	Log-rank test p value	Immuno.N
Pembrolizumab										
month=3	4.27[0.05, 8.31]	3.13[0, 6.26]	1.37[0, 12.25]	2.30E-05	120	5.25[1.8, 8.59]	2.08[1.32, 2.84]	2.52[1.1, 5.26]	9.80E-12	214
month=6	13.91[5.25, 21.78]	6.15[1.19, 10.86]	2.26[0.57, 15.6]	2.30E-05	120	15.43[8.38, 21.94]	3.82[2.72, 4.9]	4.04[2.29, 7.91]	9.80E-12	214

month=9	26.46[12.84, 37.95]	6.15[1.19, 10.86]	4.3[1.49, 27.45]	2.30E-05	120	25.53[15.1, 34.68]	5.08[3.76, 6.38]	5.03[3.03, 7.97]	9.80E-12	214
month=12	30.14[14.89, 42.65]	6.89[1.32, 12.15]	4.37[1.49, 24.54]	2.30E-05	120	30.97[18.32, 41.66]	7.34[5.64, 9.01]	4.22[2.41, 6.46]	9.80E-12	214
month=15	30.14[14.89, 42.65]	7.58[1.37, 13.41]	3.97[1.4, 18.61]	2.30E-05	120	34.26[20.22, 45.83]	8.89[6.93, 10.8]	3.86[2.34, 5.65]	9.80E-12	214
Nivolumab										
month=3	9.66[6.98, 12.26]	3.71[2.05, 5.34]	2.6[1.47, 8.89]	7.54E-23	552	10.44[8.39, 12.45]	2.08[1.32, 2.84]	5.02[3.13, 8.69]	0.00E+00	1003
month=6	15.71[12.25, 19.05]	6.55[4.22, 8.83]	2.4[1.47, 5.57]	7.54E-23	552	16.63[14, 19.17]	3.82[2.72, 4.9]	4.36[3.24, 6.06]	0.00E+00	1003
month=9	21.18[16.96, 25.19]	7.91[5.27, 10.48]	2.68[1.7, 5.62]	7.54E-23	552	23.13[19.9, 26.23]	5.08[3.76, 6.38]	4.56[3.32, 6.4]	0.00E+00	1003
month=12	26.77[21.79, 31.44]	10.05[6.91, 13.09]	2.66[1.74, 4.99]	7.54E-23	552	28.2[24.51, 31.71]	7.34[5.64, 9.01]	3.84[2.89, 4.82]	0.00E+00	1003
month=15	30.89[25.24, 36.12]	11.73[8.17, 15.16]	2.63[1.66, 4.63]	7.54E-23	552	31.3[27.26, 35.11]	8.89[6.93, 10.8]	3.52[2.82, 4.4]	0.00E+00	1003
all immunotherapy drugs										
month=3	9.25[6.92, 11.53]	3.37[1.97, 4.74]	2.75[1.54, 9.43]	2.16E-28	709	9.42[7.67, 11.12]	2.08[1.32, 2.84]	4.52[3.13, 7.5]	0.00E+00	1284
month=6	15.2[12.11, 18.19]	6.18[4.18, 8.15]	2.46[1.54, 5.45]	2.16E-28	709	15.77[13.44, 18.03]	3.82[2.72, 4.9]	4.13[3.07, 5.67]	0.00E+00	1284
month=9	20.95[17.07, 24.65]	7.14[4.93, 9.29]	2.93[1.88, 5.81]	2.16E-28	709	22.4[19.46, 25.23]	5.08[3.76, 6.38]	4.41[3.44, 5.68]	0.00E+00	1284
month=12	26.04[21.46, 30.35]	9.08[6.42, 11.67]	2.87[1.86, 5.05]	2.16E-28	709	27.35[23.96, 30.59]	7.34[5.64, 9.01]	3.73[2.86, 4.79]	0.00E+00	1284
month=15	29.76[24.57, 34.59]	11.29[8.11, 14.37]	2.63[1.71, 4.42]	2.16E-28	709	30.34[26.63, 33.87]	8.89[6.93, 10.8]	3.41[2.53, 4.06]	0.00E+00	1284

Head and Neck

time	matched					unmatched				
	immunotherapy	chemotherapy	Hazard-Ratio	Log-rank test p value	Immuno.N	immunotherapy	chemotherapy	Hazard-Ratio	Log-rank test p value	Immuno.N
Pembrolizumab										
month=3	9.46[6.08, 12.72]	4.27[2.12, 6.37]	2.22[1.34, 4.05]	8.50E-82	371	8.57[5.65, 11.39]	2.83[2.5, 3.16]	3.03[1.83, 4.04]	5.83E-14	445
month=6	18.43[13.41, 23.16]	7.82[4.79, 10.76]	2.36[1.63, 3.62]	8.50E-82	371	16.09[11.79, 20.18]	6.11[5.6, 6.61]	2.63[1.96, 3.39]	5.83E-14	445
month=9	25.81[19.43, 31.69]	11.76[7.85, 15.51]	2.19[1.6, 3.22]	8.50E-82	371	22.85[17.32, 28.02]	9.49[8.85, 10.14]	2.41[1.82, 2.88]	5.83E-14	445
month=12	30.8[23.31, 37.55]	14.53[10.01, 18.83]	2.12[1.56, 2.96]	8.50E-82	371	28.95[22.06, 35.23]	13.42[12.63, 14.21]	2.16[1.74, 2.61]	5.83E-14	445
month=15	34.09[25.47, 41.72]	19.29[13.75, 24.47]	1.77[1.28, 2.44]	8.50E-82	371	31.63[23.92, 38.56]	17.38[16.46, 18.3]	1.82[1.42, 2.19]	5.83E-14	445
Nivolumab										
month=3	11.85[7.87, 15.66]	4.73[2.24, 7.16]	2.5[1.49, 4]	4.87E-73	312	11.5[7.87, 14.99]	2.83[2.5, 3.16]	4.06[2.81, 5.67]	0.00E+00	356
month=6	19.98[14.6, 25.02]	8.84[5.3, 12.25]	2.26[1.52, 3.39]	4.87E-73	312	18.37[13.61, 22.86]	6.11[5.6, 6.61]	3.01[2.33, 3.96]	0.00E+00	356
month=9	29.78[22.8, 36.13]	13.46[8.89, 17.8]	2.21[1.62, 3.25]	4.87E-73	312	27.75[21.52, 33.47]	9.49[8.85, 10.14]	2.92[2.33, 3.6]	0.00E+00	356
month=12	37.91[29.42, 45.37]	18.54[12.93, 23.79]	2.04[1.52, 2.88]	4.87E-73	312	35.03[27.6, 41.7]	13.42[12.63, 14.21]	2.61[2.12, 3.17]	0.00E+00	356
month=15	44.54[34.78, 52.84]	21.93[15.67, 27.72]	2.03[1.55, 2.76]	4.87E-73	312	41.64[32.97, 49.18]	17.38[16.46, 18.3]	2.39[2.02, 2.9]	0.00E+00	356
all immunotherapy drugs										
month=3	11.13[8.57, 13.61]	4.4[2.84, 5.94]	2.53[1.77, 3.65]	2.31E-163	731	10.41[8.15, 12.62]	2.83[2.5, 3.16]	3.68[2.79, 4.79]	0.00E+00	857

month=6	19.77[16.22, 23.17]	8.68[6.4, 10.91]	2.28[1.8, 3.14]	2.31E-163	731	17.84[14.74, 20.83]	6.11[5.6, 6.61]	2.92[2.35, 3.48]	0.00E+00	857
month=9	28.08[23.58, 32.32]	12.97[10.06, 15.78]	2.17[1.77, 2.91]	2.31E-163	731	25.47[21.53, 29.21]	9.49[8.85, 10.14]	2.68[2.22, 3.17]	0.00E+00	857
month=12	33.81[28.52, 38.71]	16.24[12.86, 19.48]	2.08[1.72, 2.73]	2.31E-163	731	31.59[26.88, 36]	13.42[12.63, 14.21]	2.35[1.97, 2.82]	0.00E+00	857
month=15	39.2[33.01, 44.83]	20.65[16.67, 24.43]	1.9[1.56, 2.43]	2.31E-163	731	36.96[31.45, 42.02]	17.38[16.46, 18.3]	2.13[1.83, 2.46]	0.00E+00	857

Squamous

	matched					unmatched				
time	immunotherapy	chemotherapy	Hazard-Ratio	Log-rank test p value	Immuno.N	immunotherapy	chemotherapy	Hazard-Ratio	Log-rank test p value	Immuno.N
Pembrolizumab										
month=3	5.17[1.8, 8.42]	1.24[0, 2.81]	4.15[0.79, 38.91]	6.42E-03	197	6.62[3.5, 9.64]	1.62[1.04, 2.19]	4.09[1.87, 6.41]	4.21E-07	295
month=6	11.24[5.72, 16.44]	6.07[2.19, 9.8]	1.85[0.64, 5.5]	6.42E-03	197	13.06[8.26, 17.61]	4.28[3.29, 5.26]	3.05[1.73, 4.2]	4.21E-07	295
month=9	16.33[9.02, 23.06]	7.58[3.14, 11.82]	2.15[0.86, 5.68]	6.42E-03	197	16.48[10.72, 21.86]	6.16[4.93, 7.38]	2.67[1.6, 3.77]	4.21E-07	295
month=12	22.73[13.35, 31.09]	11.64[5.58, 17.32]	1.95[0.82, 4.05]	6.42E-03	197	21.83[14.6, 28.45]	9.02[7.45, 10.56]	2.42[1.67, 3.26]	4.21E-07	295
month=15	25[14.71, 34.06]	16.04[8.42, 23.02]	1.56[0.71, 3.43]	6.42E-03	197	24.86[16.62, 32.29]	11.44[9.59, 13.25]	2.17[1.47, 2.88]	4.21E-07	295
Nivolumab										
month=3	7.63[3.18, 11.87]	2.2[0, 4.56]	3.47[1.06, 49.64]	9.08E-12	159	7.44[3.73, 11.01]	1.62[1.04, 2.19]	4.6[2.09, 10.83]	4.38E-10	231
month=6	16.08[9.45, 22.23]	5.33[1.29, 9.19]	3.02[1.44, 14.74]	9.08E-12	159	13.64[8.44, 18.53]	4.28[3.29, 5.26]	3.19[1.92, 5.06]	4.38E-10	231
month=9	21.8[13.73, 29.12]	5.83[1.57, 9.9]	3.74[1.91, 15.48]	9.08E-12	159	19.35[12.86, 25.36]	6.16[4.93, 7.38]	3.14[2.08, 4.45]	4.38E-10	231
month=12	28.63[18.94, 37.16]	9.86[3.76, 15.58]	2.9[1.54, 7.57]	9.08E-12	159	26.42[18.41, 33.64]	9.02[7.45, 10.56]	2.93[1.8, 3.88]	4.38E-10	231
month=15	32.46[21.64, 41.78]	11.9[4.87, 18.41]	2.73[1.33, 6.56]	9.08E-12	159	30.77[21.6, 38.87]	11.44[9.59, 13.25]	2.69[1.72, 3.66]	4.38E-10	231
all immunotherapy drugs										
month=3	7.25[4.45, 9.96]	1.44[0.19, 2.68]	5.02[2.09, 41.61]	8.21E-15	391	7.23[4.98, 9.44]	1.62[1.04, 2.19]	4.47[2.63, 7.26]	1.90E-14	602
month=6	13.97[9.86, 17.89]	5.29[2.7, 7.81]	2.64[1.53, 6.34]	8.21E-15	391	13.2[9.98, 16.3]	4.28[3.29, 5.26]	3.08[2.05, 4.26]	1.90E-14	602
month=9	19.38[14.24, 24.21]	7.36[4.21, 10.4]	2.63[1.63, 5.56]	8.21E-15	391	17.81[13.86, 21.59]	6.16[4.93, 7.38]	2.89[2.1, 3.61]	1.90E-14	602
month=12	24.78[18.64, 30.45]	11.91[7.56, 16.05]	2.08[1.33, 3.99]	8.21E-15	391	23.53[18.69, 28.08]	9.02[7.45, 10.56]	2.61[1.96, 3.36]	1.90E-14	602
month=15	28.41[21.49, 34.73]	15.67[10.34, 20.68]	1.81[1.19, 3.56]	8.21E-15	391	27.17[21.67, 32.28]	11.44[9.59, 13.25]	2.37[1.76, 2.96]	1.90E-14	602

Basal

	matched					unmatched				
time	immunotherapy	chemotherapy	Hazard-Ratio	Log-rank test p value	Immuno.N	immunotherapy	chemotherapy	Hazard-Ratio	Log-rank test p value	Immuno.N
Pembrolizumab										
month=3	4.46[0.54, 8.23]	1.02[0, 2.74]	4.36[0.33, 19.83]	1.84E-05	139	5.91[2.84, 8.89]	1.81[0.9, 2.71]	3.27[1.45, 6.87]	2.43E-07	289
month=6	8.24[2.48, 13.66]	4.39[0.57, 8.06]	1.88[0.38, 11.07]	1.84E-05	139	9.95[5.73, 13.98]	3.49[2.18, 4.78]	2.85[1.67, 4.23]	2.43E-07	289
month=9	15.33[7.05, 22.88]	5.36[1.11, 9.43]	2.86[0.82, 14.59]	1.84E-05	139	15.63[10.02, 20.9]	5.28[3.62, 6.92]	2.96[1.72, 4.74]	2.43E-07	289
month=12	21.72[10.94, 31.19]	7.79[2.39, 12.88]	2.79[0.78, 10.93]	1.84E-05	139	20.05[13.1, 26.45]	6.61[4.69, 8.49]	3.03[1.95, 4.3]	2.43E-07	289
month=15	21.72[10.94, 31.19]	7.79[2.39, 12.88]	2.79[0.78, 10.93]	1.84E-05	139	20.05[13.1, 26.45]	7.92[5.75, 10.04]	2.53[1.59, 3.63]	2.43E-07	289

Nivolumab										
month=3	7.96[2.8, 12.84]	0.91[0, 2.66]	8.79[0.92, 12.29]	9.49E-05	127	6.85[3.73, 9.88]	1.81[0.9, 2.71]	3.79[1.9, 8.4]	1.11E-16	296
month=6	13.7[6.64, 20.22]	3.72[0, 7.36]	3.68[1.03, 26.02]	9.49E-05	127	13.14[8.64, 17.41]	3.49[2.18, 4.78]	3.77[2.4, 6.29]	1.11E-16	296
month=9	19.29[10.56, 27.17]	6.71[1.44, 11.7]	2.87[1.13, 17.41]	9.49E-05	127	18.3[12.72, 23.52]	5.28[3.62, 6.92]	3.46[2.4, 5.39]	1.11E-16	296
month=12	26.02[15.45, 35.26]	14.37[6.27, 21.76]	1.81[0.75, 9.95]	9.49E-05	127	22.89[16.41, 28.87]	6.61[4.69, 8.49]	3.46[2.37, 5.12]	1.11E-16	296
month=15	34.3[20.52, 45.69]	16.47[7.66, 24.44]	2.08[0.88, 8.17]	9.49E-05	127	30.76[22.42, 38.21]	7.92[5.75, 10.04]	3.89[2.81, 5.72]	1.11E-16	296
all immunotherapy drugs										
month=3	7.96[2.8, 12.84]	0.91[0, 2.66]	8.79[0.92, 12.29]	9.49E-05	127	7.35[5.19, 9.45]	1.81[0.9, 2.71]	4.06[2.23, 8.11]	0.00E+00	695
month=6	13.7[6.64, 20.22]	3.72[0, 7.36]	3.68[1.03, 26.02]	9.49E-05	127	12.96[9.98, 15.84]	3.49[2.18, 4.78]	3.72[2.05, 5.85]	0.00E+00	695
month=9	19.29[10.56, 27.17]	6.71[1.44, 11.7]	2.87[1.13, 17.41]	9.49E-05	127	17.86[14.2, 21.36]	5.28[3.62, 6.92]	3.38[2.05, 5.1]	0.00E+00	695
month=12	26.02[15.45, 35.26]	14.37[6.27, 21.76]	1.81[0.75, 9.95]	9.49E-05	127	21.62[17.4, 25.62]	6.61[4.69, 8.49]	3.27[2.13, 4.86]	0.00E+00	695
month=15	34.3[20.52, 45.69]	16.47[7.66, 24.44]	2.08[0.88, 8.17]	9.49E-05	127	26.22[21.2, 30.91]	7.92[5.75, 10.04]	3.31[2.19, 4.47]	0.00E+00	695

Brain

	matched					unmatched				
time	immunotherapy	chemotherapy	Hazard-Ratio	Log-rank test p value	Immuno.N	immunotherapy	chemotherapy	Hazard-Ratio	Log-rank test p value	Immuno.N
Pembrolizumab										
month=3	4.15[0.04, 8.09]	3.48[0, 6.89]	1.19[0, 6.97]	1.46E-01	118	3.95[0.46, 7.32]	3.77[3.23, 4.31]	1.05[0.18, 2.25]	4.76E-01	162
month=6	5.41[0.63, 9.96]	4.75[0.4, 8.91]	1.14[0, 4.19]	1.46E-01	118	4.88[0.95, 8.66]	6.41[5.68, 7.13]	0.76[0.15, 1.55]	4.76E-01	162
month=9	5.41[0.63, 9.96]	5.41[0.52, 10.05]	1[0, 3.41]	1.46E-01	118	9.61[2.97, 15.79]	8.08[7.23, 8.92]	1.19[0.36, 2.12]	4.76E-01	162
month=12	5.41[0.63, 9.96]	6.9[0.87, 12.58]	0.78[0, 2.41]	1.46E-01	118	11.66[3.93, 18.77]	9.67[8.71, 10.63]	1.21[0.54, 2.22]	4.76E-01	162
month=15	5.41[0.63, 9.96]	18.93[6.43, 29.76]	0.29[0, 0.97]	1.46E-01	118	11.66[3.93, 18.77]	11.4[10.3, 12.49]	1.02[0.45, 1.88]	4.76E-01	162
Nivolumab										
month=3	16.07[9.11, 22.49]	2.76[0, 5.62]	5.83[2.16, 21.26]	5.29E-05	138	14.55[8.69, 20.03]	3.77[3.23, 4.31]	3.86[2.36, 5.83]	2.30E-09	176
month=6	19.07[11.48, 26]	4.28[0.37, 8.05]	4.45[1.86, 11.8]	5.29E-05	138	19.3[12.52, 25.54]	6.41[5.68, 7.13]	3.01[2.14, 4.15]	2.30E-09	176
month=9	20.79[12.54, 28.26]	9.15[2.53, 15.32]	2.27[1, 6.31]	5.29E-05	138	21.9[14.32, 28.8]	8.08[7.23, 8.92]	2.71[1.98, 3.77]	2.30E-09	176
month=12	22.87[13.73, 31.04]	10.37[3.03, 17.16]	2.21[0.93, 5.62]	5.29E-05	138	23.56[15.37, 30.95]	9.67[8.71, 10.63]	2.44[1.71, 3.29]	2.30E-09	176
month=15	22.87[13.73, 31.04]	11.6[3.47, 19.05]	1.97[0.81, 4.84]	5.29E-05	138	26.74[16.44, 35.77]	11.4[10.3, 12.49]	2.35[1.61, 3.38]	2.30E-09	176
all immunotherapy drugs										
month=3	11.5[7.57, 15.26]	2.36[0.56, 4.13]	4.87[2.25, 11.42]	2.35E-07	307	10.23[6.98, 13.37]	3.77[3.23, 4.31]	2.71[2.03, 3.88]	3.09E-09	407
month=6	13.83[9.47, 17.98]	4.04[1.51, 6.51]	3.42[1.73, 6.33]	2.35E-07	307	13.09[9.35, 16.67]	6.41[5.68, 7.13]	2.04[1.39, 2.66]	3.09E-09	407
month=9	16.31[11.18, 21.13]	6.66[3.01, 10.16]	2.45[1.29, 4.59]	2.35E-07	307	17.85[12.98, 22.45]	8.08[7.23, 8.92]	2.21[1.68, 2.72]	3.09E-09	407
month=12	17.31[11.85, 22.44]	8.19[3.91, 12.28]	2.11[1.12, 3.83]	2.35E-07	307	19.44[14.14, 24.41]	9.67[8.71, 10.63]	2.01[1.54, 2.6]	3.09E-09	407
month=15	17.31[11.85, 22.44]	13.92[7.36, 20.01]	1.24[0.71, 2.43]	2.35E-07	307	20.76[14.89, 26.23]	11.4[10.3, 12.49]	1.82[1.34, 2.36]	3.09E-09	407

Lung

	matched	unmatched

time	immunotherapy	chemotherapy	Hazard-Ratio	Log-rank test p value	Immuno.N	immunotherapy	chemotherapy	Hazard-Ratio	Log-rank test p value	Immuno.N
Pembrolizumab										
month=3	5.67[4.7, 6.63]	3.59[2.85, 4.32]	1.58[1.23, 1.91]	5.47E-104	2773	5.51[4.58, 6.43]	3.25[3.03, 3.48]	1.69[1.47, 2.01]	3.77E-15	2913
month=6	9.04[7.73, 10.34]	6.58[5.52, 7.63]	1.37[1.13, 1.6]	5.47E-104	2773	8.82[7.56, 10.07]	6.09[5.77, 6.42]	1.45[1.27, 1.69]	3.77E-15	2913
month=9	14.19[12.33, 16]	9.26[7.91, 10.58]	1.53[1.26, 1.75]	5.47E-104	2773	14.03[12.24, 15.79]	8.76[8.35, 9.18]	1.6[1.38, 1.84]	3.77E-15	2913
month=12	18.56[16.21, 20.85]	11.91[10.28, 13.52]	1.56[1.3, 1.77]	5.47E-104	2773	18.43[16.15, 20.65]	11.46[10.96, 11.97]	1.61[1.41, 1.86]	3.77E-15	2913
month=15	21.48[18.73, 24.13]	14.22[12.31, 16.09]	1.51[1.28, 1.74]	5.47E-104	2773	21.12[18.49, 23.67]	13.74[13.15, 14.33]	1.54[1.33, 1.79]	3.77E-15	2913
Nivolumab										
month=3	7.19[6, 8.37]	3.9[3.06, 4.74]	1.84[1.45, 2.23]	1.59E-106	2279	6.91[5.77, 8.03]	3.25[3.03, 3.48]	2.12[1.79, 2.5]	0.00E+00	2424
month=6	12.23[10.57, 13.85]	7.27[6.04, 8.48]	1.68[1.4, 1.99]	1.59E-106	2279	11.88[10.29, 13.45]	6.09[5.77, 6.42]	1.95[1.7, 2.22]	0.00E+00	2424
month=9	17.18[15.04, 19.26]	10.08[8.54, 11.59]	1.7[1.43, 1.97]	1.59E-106	2279	17[14.92, 19.02]	8.76[8.35, 9.18]	1.94[1.7, 2.18]	0.00E+00	2424
month=12	19.74[17.33, 22.08]	12.99[11.1, 14.84]	1.52[1.3, 1.75]	1.59E-106	2279	19.66[17.32, 21.94]	11.46[10.96, 11.97]	1.72[1.5, 1.95]	0.00E+00	2424
month=15	22.71[19.94, 25.39]	15.06[12.91, 17.16]	1.51[1.28, 1.74]	1.59E-106	2279	22.44[19.77, 25.02]	13.74[13.15, 14.33]	1.63[1.41, 1.84]	0.00E+00	2424
all immunotherapy drugs										
month=3	6.15[5.46, 6.83]	3.72[3.2, 4.23]	1.65[1.4, 1.91]	4.85E-242	5834	6.04[5.38, 6.7]	3.25[3.03, 3.48]	1.86[1.61, 2.09]	0.00E+00	6157
month=6	10.47[9.5, 11.42]	6.96[6.21, 7.7]	1.5[1.32, 1.69]	4.85E-242	5834	10.3[9.36, 11.22]	6.09[5.77, 6.42]	1.69[1.5, 1.86]	0.00E+00	6157
month=9	15.45[14.16, 16.73]	9.75[8.81, 10.69]	1.58[1.4, 1.76]	4.85E-242	5834	15.47[14.21, 16.71]	8.76[8.35, 9.18]	1.77[1.65, 1.95]	0.00E+00	6157
month=12	19.24[17.67, 20.78]	12.7[11.53, 13.85]	1.52[1.35, 1.69]	4.85E-242	5834	19.26[17.74, 20.76]	11.46[10.96, 11.97]	1.68[1.53, 1.84]	0.00E+00	6157
month=15	21.94[20.15, 23.69]	14.78[13.45, 16.09]	1.48[1.33, 1.65]	4.85E-242	5834	21.77[20.05, 23.46]	13.74[13.15, 14.33]	1.58[1.45, 1.7]	0.00E+00	6157

Table S11. The cumulative incidence rate (%) of autoimmune diseases of the immunotherapy and the targeted therapy groups.

Renal

time	matched					unmatched				
	immunotherapy	chemotherapy	Hazard-Ratio	Log-rank test p value	Immuno.N	immunotherapy	chemotherapy	Hazard-Ratio	Log-rank test p value	Immuno.N
Pembrolizumab										
month=3	3.51[0, 7.41]	6.68[1.71, 11.4]	0.53[0, 1.58]	1.09E-02	120	5.25[1.8, 8.59]	4.24[3.27, 5.21]	1.24[0.47, 2.85]	1.42E-03	214
month=6	19.7[8, 29.92]	10.03[3.65, 15.99]	1.96[0.63, 4.92]	1.09E-02	120	15.43[8.38, 21.94]	7.47[6.11, 8.81]	2.07[1.16, 3.26]	1.42E-03	214
month=9	28.88[13.59, 41.47]	13.83[5.84, 21.13]	2.09[0.83, 4.96]	1.09E-02	120	25.53[15.1, 34.68]	11.26[9.48, 12.99]	2.27[1.35, 3.46]	1.42E-03	214
month=12	28.88[13.59, 41.47]	17.49[7.83, 26.14]	1.65[0.62, 3.71]	1.09E-02	120	30.97[18.32, 41.66]	13.99[11.91, 16.02]	2.21[1.16, 3.09]	1.42E-03	214
month=15	28.88[13.59, 41.47]	21.54[9.83, 31.73]	1.34[0.47, 2.68]	1.09E-02	120	34.26[20.22, 45.83]	17.58[15.11, 19.99]	1.95[1.07, 3.01]	1.42E-03	214
Nivolumab										
month=3	9.16[6.67, 11.58]	4.26[2.52, 5.97]	2.15[1.27, 4.78]	4.24E-18	606	10.46[8.4, 12.47]	4.24[3.27, 5.21]	2.47[1.82, 3.38]	3.33E-16	1002
month=6	15.43[12.13, 18.6]	6.95[4.61, 9.23]	2.22[1.38, 4.03]	4.24E-18	606	16.65[14.02, 19.2]	7.47[6.11, 8.81]	2.23[1.78, 3]	3.33E-16	1002
month=9	21.2[17.17, 25.03]	11.86[8.53, 15.06]	1.79[1.23, 2.87]	4.24E-18	606	23.16[19.93, 26.26]	11.26[9.48, 12.99]	2.06[1.67, 2.64]	3.33E-16	1002
month=12	26.87[22.15, 31.31]	15.48[11.42, 19.35]	1.74[1.22, 2.74]	4.24E-18	606	28.25[24.55, 31.76]	13.99[11.91, 16.02]	2.02[1.69, 2.48]	3.33E-16	1002
month=15	31.03[25.68, 35.99]	20.33[15.35, 25.01]	1.53[1.1, 2.34]	4.24E-18	606	31.36[27.31, 35.18]	17.58[15.11, 19.99]	1.78[1.48, 2.21]	3.33E-16	1002
all immunotherapy drugs										
month=3	8.7[6.5, 10.84]	4.53[2.92, 6.12]	1.92[1.13, 3.68]	6.48E-15	749	9.42[7.67, 11.12]	4.24[3.27, 5.21]	2.22[1.6, 2.78]	1.07E-14	1284
month=6	15.35[12.32, 18.28]	7.5[5.3, 9.64]	2.05[1.33, 3.3]	6.48E-15	749	15.77[13.44, 18.03]	7.47[6.11, 8.81]	2.11[1.67, 2.73]	1.07E-14	1284
month=9	21.1[17.35, 24.68]	13.18[10.01, 16.24]	1.6[1.15, 2.49]	6.48E-15	749	22.4[19.46, 25.23]	11.26[9.48, 12.99]	1.99[1.59, 2.51]	1.07E-14	1284
month=12	26.32[21.92, 30.48]	17.09[13.22, 20.79]	1.54[1.14, 2.41]	6.48E-15	749	27.35[23.96, 30.59]	13.99[11.91, 16.02]	1.95[1.63, 2.48]	1.07E-14	1284
month=15	30.09[25.13, 34.72]	21.83[17.16, 26.24]	1.38[1.01, 2.02]	6.48E-15	749	30.35[26.63, 33.88]	17.58[15.11, 19.99]	1.73[1.49, 2.22]	1.07E-14	1284

Head and Neck

time	matched					unmatched				
	immunotherapy	chemotherapy	Hazard-Ratio	Log-rank test p value	Immuno.N	immunotherapy	chemotherapy	Hazard-Ratio	Log-rank test p value	Immuno.N
Pembrolizumab										
month=3	9.84[5.97, 13.55]	2.64[0.75, 4.49]	3.73[1.54, 8.5]	6.32E-10	308	8.57[5.66, 11.4]	3.02[2.31, 3.72]	2.84[1.86, 4.3]	2.28E-10	444
month=6	19.26[13.52, 24.62]	7.7[4.27, 11]	2.5[1.42, 5.12]	6.32E-10	308	16.1[11.8, 20.19]	5.96[4.92, 6.99]	2.7[1.97, 3.63]	2.28E-10	444
month=9	27.06[19.68, 33.77]	13.61[8.67, 18.29]	1.99[1.27, 3.77]	6.32E-10	308	22.86[17.32, 28.03]	10.52[9.06, 11.95]	2.17[1.57, 2.99]	2.28E-10	444
month=12	32.26[23.56, 39.97]	18.75[12.62, 24.44]	1.72[1.16, 3.03]	6.32E-10	308	28.95[22.06, 35.24]	14.19[12.43, 15.91]	2.04[1.42, 2.71]	2.28E-10	444
month=15	36.02[26.08, 44.63]	25.08[17.66, 31.83]	1.44[0.94, 2.3]	6.32E-10	308	31.63[23.93, 38.56]	18.96[16.84, 21.02]	1.67[1.18, 2.18]	2.28E-10	444
Nivolumab										
month=3	11.2[6.84, 15.35]	2.67[0.55, 4.74]	4.2[1.69, 12.65]	3.59E-17	247	11.43[7.82, 14.9]	3.02[2.31, 3.72]	3.79[2.6, 5.47]	0.00E+00	358

month=6	21.04[14.74, 26.87]	6.48[2.95, 9.87]	3.25[1.64, 6.56]	3.59E-17	247	18.27[13.53, 22.74]	5.96[4.92, 6.99]	3.07[2.16, 4.04]	0.00E+00	358
month=9	28.26[20.57, 35.2]	11.46[6.43, 16.21]	2.47[1.41, 4.44]	3.59E-17	247	27.59[21.4, 33.29]	10.52[9.06, 11.95]	2.62[1.87, 3.42]	0.00E+00	358
month=12	38.87[28.86, 47.46]	16.33[9.9, 22.31]	2.38[1.48, 3.96]	3.59E-17	247	35.61[28.11, 42.32]	14.19[12.43, 15.91]	2.51[1.77, 3.22]	0.00E+00	358
month=15	46.34[34.52, 56.03]	22.92[14.79, 30.27]	2.02[1.29, 3.17]	3.59E-17	247	42.15[33.47, 49.69]	18.96[16.84, 21.02]	2.22[1.71, 2.79]	0.00E+00	358
all immunotherapy drugs										
month=3	10.36[7.6, 13.04]	3.63[2.02, 5.2]	2.86[1.6, 5.39]	1.34E-28	595	10.39[8.13, 12.59]	3.02[2.31, 3.72]	3.44[2.37, 5.42]	0.00E+00	858
month=6	20.05[16.02, 23.89]	7.34[4.96, 9.66]	2.73[1.76, 4.44]	1.34E-28	595	17.81[14.71, 20.79]	5.96[4.92, 6.99]	2.99[2.25, 3.86]	0.00E+00	858
month=9	27.28[22.3, 31.93]	12.28[8.99, 15.46]	2.22[1.55, 3.36]	1.34E-28	595	25.41[21.48, 29.14]	10.52[9.06, 11.95]	2.42[1.85, 2.99]	0.00E+00	858
month=12	34.25[28.24, 39.77]	16.21[12.23, 20.01]	2.11[1.54, 3.11]	1.34E-28	595	31.88[27.14, 36.31]	14.19[12.43, 15.91]	2.25[1.81, 2.74]	0.00E+00	858
month=15	40.69[33.57, 47.04]	22.1[17.19, 26.72]	1.84[1.36, 2.64]	1.34E-28	595	37.22[31.7, 42.3]	18.96[16.84, 21.02]	1.96[1.6, 2.35]	0.00E+00	858

Squamous

	matched					unmatched				
time	immunotherapy	chemotherapy	Hazard-Ratio	Log-rank test p value	Immuno.N	immunotherapy	chemotherapy	Hazard-Ratio	Log-rank test p value	Immuno.N
Pembrolizumab										
month=3	8.41[1.7, 14.66]	2.46[0, 6.32]	3.41[0, 20.42]	1.11E-01	79	6.62[3.5, 9.64]	3.07[1.17, 4.93]	2.16[0.9, 5.15]	7.50E-02	295
month=6	12.85[3.76, 21.07]	2.46[0, 6.32]	5.21[0, 31.18]	1.11E-01	79	13.06[8.26, 17.61]	5.42[2.77, 7.99]	2.41[1.21, 5.19]	7.50E-02	295
month=9	16.33[4.96, 26.34]	5.41[0, 12.02]	3.02[0.23, 32.59]	1.11E-01	79	16.48[10.72, 21.86]	9.26[5.56, 12.81]	1.78[0.99, 3.13]	7.50E-02	295
month=12	16.33[4.96, 26.34]	7.48[0, 15.84]	2.18[0.18, 22.72]	1.11E-01	79	21.83[14.6, 28.45]	11.17[6.93, 15.22]	1.95[1.19, 3.28]	7.50E-02	295
month=15	22.77[5.47, 36.9]	15.22[0, 28.41]	1.5[0.12, 15.51]	1.11E-01	79	24.86[16.62, 32.29]	19.42[13.21, 25.19]	1.28[0.79, 1.93]	7.50E-02	295
Nivolumab										
month=3	3.78[0, 8.79]	6.63[0, 12.97]	0.57[0, 4.44]	1.91E-01	59	7.44[3.73, 11.01]	3.07[1.17, 4.93]	2.42[1.26, 6.58]	7.22E-03	231
month=6	15.26[4.05, 25.16]	6.63[0, 12.97]	2.3[0.28, 12.15]	1.91E-01	59	13.64[8.44, 18.53]	5.42[2.77, 7.99]	2.52[1.66, 5.13]	7.22E-03	231
month=9	21.36[7.53, 33.12]	6.63[0, 12.97]	3.22[0.61, 14.8]	1.91E-01	59	19.35[12.86, 25.36]	9.26[5.56, 12.81]	2.09[1.38, 3.83]	7.22E-03	231
month=12	24.93[9.6, 37.67]	10.18[0.38, 19]	2.45[0.72, 14.13]	1.91E-01	59	26.42[18.41, 33.64]	11.17[6.93, 15.22]	2.36[1.57, 4.43]	7.22E-03	231
month=15	30.3[11.75, 44.95]	29.34[8.47, 45.45]	1.03[0.33, 5.05]	1.91E-01	59	30.77[21.6, 38.87]	19.42[13.21, 25.19]	1.58[0.97, 2.86]	7.22E-03	231
all immunotherapy drugs										
month=3	7.45[2.88, 11.8]	3.26[0.09, 6.33]	2.29[0.49, 14.14]	3.02E-02	148	7.23[4.98, 9.44]	3.07[1.17, 4.93]	2.36[1.13, 5.73]	1.08E-02	602
month=6	13.48[6.96, 19.54]	5.42[1.07, 9.57]	2.49[0.68, 16.66]	3.02E-02	148	13.2[9.98, 16.3]	5.42[2.77, 7.99]	2.44[1.36, 4.09]	1.08E-02	602
month=9	19.37[10.84, 27.08]	8.23[2.42, 13.7]	2.35[0.79, 13.2]	3.02E-02	148	17.81[13.86, 21.59]	9.26[5.56, 12.81]	1.92[1.18, 3.34]	1.08E-02	602
month=12	23.06[13.27, 31.75]	10.85[3.72, 17.45]	2.13[0.86, 11.11]	3.02E-02	148	23.53[18.69, 28.08]	11.17[6.93, 15.22]	2.11[1.29, 3.09]	1.08E-02	602
month=15	28.31[16.2, 38.67]	22.57[10.71, 32.86]	1.25[0.56, 3.8]	3.02E-02	148	27.17[21.67, 32.28]	19.42[13.21, 25.19]	1.4[0.93, 2.05]	1.08E-02	602

Brain

time	matched					unmatched				
	immunotherapy	chemotherapy	Hazard-Ratio	Log-rank test p value	Immuno.N	immunotherapy	chemotherapy	Hazard-Ratio	Log-rank test p value	Immuno.N
Pembrolizumab										
month=3	5.18[0.05, 10.05]	2.69[0, 5.91]	1.92[0, 17.29]	3.23E-01	95	3.95[0.46, 7.32]	2.79[2.15, 3.43]	1.41[0.28, 2.81]	3.84E-01	162
month=6	5.18[0.05, 10.05]	7.05[0.77, 12.93]	0.73[0, 3.9]	3.23E-01	95	4.88[0.95, 8.66]	6.3[5.24, 7.34]	0.78[0.13, 1.47]	3.84E-01	162
month=9	7.61[0.59, 14.14]	10.96[1.97, 19.12]	0.69[0.12, 2.76]	3.23E-01	95	9.61[2.97, 15.79]	8.43[7.11, 9.73]	1.14[0.32, 2.19]	3.84E-01	162
month=12	7.61[0.59, 14.14]	10.96[1.97, 19.12]	0.69[0.12, 2.76]	3.23E-01	95	11.66[3.93, 18.77]	9.85[8.34, 11.33]	1.18[0.42, 2.18]	3.84E-01	162
month=15	7.61[0.59, 14.14]	10.96[1.97, 19.12]	0.69[0.12, 2.76]	3.23E-01	95	11.66[3.93, 18.77]	11.44[9.65, 13.19]	1.02[0.36, 1.93]	3.84E-01	162
Nivolumab										
month=3	19.14[10.14, 27.24]	1.39[0, 3.67]	13.78[2.58, 135.92]	2.19E-08	100	14.45[8.63, 19.9]	2.79[2.15, 3.43]	5.18[3.03, 7.9]	1.52E-09	177
month=6	26.36[15.82, 35.58]	5.78[0.22, 11.03]	4.56[1.8, 36.12]	2.19E-08	100	19.16[12.43, 25.37]	6.3[5.24, 7.34]	3.04[1.79, 4.22]	1.52E-09	177
month=9	26.36[15.82, 35.58]	5.78[0.22, 11.03]	4.56[1.8, 36.12]	2.19E-08	100	21.76[14.23, 28.64]	8.43[7.11, 9.73]	2.58[1.42, 3.45]	1.52E-09	177
month=12	26.36[15.82, 35.58]	11.64[1.77, 20.52]	2.26[0.86, 22.76]	2.19E-08	100	23.43[15.28, 30.8]	9.85[8.34, 11.33]	2.38[1.37, 3.25]	1.52E-09	177
month=15	26.36[15.82, 35.58]	11.64[1.77, 20.52]	2.26[0.86, 22.76]	2.19E-08	100	26.62[16.34, 35.64]	11.44[9.65, 13.19]	2.33[1.32, 3.29]	1.52E-09	177
all immunotherapy drugs										
month=3	11.38[6.85, 15.7]	1.16[0, 2.57]	9.8[2.67, 60.13]	2.65E-08	235	10.2[6.96, 13.33]	2.79[2.15, 3.43]	3.65[2.55, 5.18]	3.39E-09	408
month=6	13.93[8.83, 18.74]	5.48[1.87, 8.96]	2.54[1.15, 7.39]	2.65E-08	235	13.05[9.32, 16.62]	6.3[5.24, 7.34]	2.07[1.66, 2.96]	3.39E-09	408
month=9	17.11[10.93, 22.85]	9.54[4.1, 14.66]	1.79[0.85, 4.33]	2.65E-08	235	17.81[12.95, 22.41]	8.43[7.11, 9.73]	2.11[1.61, 2.85]	3.39E-09	408
month=12	17.11[10.93, 22.85]	9.88[4.29, 15.14]	1.73[0.82, 4.11]	2.65E-08	235	19.4[14.11, 24.37]	9.85[8.34, 11.33]	1.97[1.59, 2.56]	3.39E-09	408
month=15	17.11[10.93, 22.85]	9.88[4.29, 15.14]	1.73[0.82, 4.11]	2.65E-08	235	20.73[14.86, 26.19]	11.44[9.65, 13.19]	1.81[1.34, 2.36]	3.39E-09	408

Lung

time	matched					unmatched				
	immunotherapy	chemotherapy	Hazard-Ratio	Log-rank test p value	Immuno.N	immunotherapy	chemotherapy	Hazard-Ratio	Log-rank test p value	Immuno.N
Pembrolizumab										
month=3	5.33[4.33, 6.32]	2.66[1.98, 3.34]	2[1.42, 2.9]	7.72E-43	2441	5.5[4.57, 6.42]	2.75[2.32, 3.19]	2[1.49, 2.45]	3.33E-16	2919
month=6	8.7[7.32, 10.05]	5.03[4.04, 6.01]	1.73[1.33, 2.34]	7.72E-43	2441	8.86[7.59, 10.11]	5.34[4.69, 5.97]	1.66[1.4, 1.97]	3.33E-16	2919
month=9	13.77[11.83, 15.67]	7.96[6.59, 9.31]	1.73[1.36, 2.19]	7.72E-43	2441	14.06[12.26, 15.81]	8.04[7.2, 8.88]	1.75[1.47, 2.03]	3.33E-16	2919
month=12	18.44[15.94, 20.87]	10.62[8.92, 12.28]	1.74[1.37, 2.16]	7.72E-43	2441	18.44[16.16, 20.66]	10.74[9.7, 11.77]	1.72[1.44, 2.05]	3.33E-16	2919
month=15	21.24[18.34, 24.04]	13.04[11.03, 15]	1.63[1.29, 2.03]	7.72E-43	2441	21.12[18.5, 23.66]	12.87[11.67, 14.06]	1.64[1.38, 1.93]	3.33E-16	2919
Nivolumab										
month=3	6.69[5.46, 7.9]	2.65[1.9, 3.4]	2.52[1.68, 3.6]	5.26E-45	2020	6.88[5.75, 7.99]	2.75[2.32, 3.19]	2.5[2, 3.07]	0.00E+00	2435
month=6	11.94[10.19, 13.66]	5.77[4.59, 6.94]	2.07[1.58, 2.76]	5.26E-45	2020	11.91[10.32, 13.47]	5.34[4.69, 5.97]	2.23[1.91, 2.61]	0.00E+00	2435
month=9	17.31[15.01, 19.56]	8.91[7.32, 10.47]	1.94[1.52, 2.48]	5.26E-45	2020	17.01[14.94, 19.04]	8.04[7.2, 8.88]	2.11[1.82, 2.44]	0.00E+00	2435
month=12	20.08[17.47, 22.62]	11.94[9.95, 13.88]	1.68[1.32, 2.11]	5.26E-45	2020	19.68[17.33, 21.95]	10.74[9.7, 11.77]	1.83[1.58, 2.16]	0.00E+00	2435
month=15	23.02[20.03, 25.9]	14.78[12.4, 17.09]	1.56[1.24, 1.95]	5.26E-45	2020	22.45[19.78, 25.03]	12.87[11.67, 14.06]	1.74[1.5, 2.07]	0.00E+00	2435

all immunotherapy drugs										
month=3	6.03[5.31, 6.75]	2.76[2.29, 3.24]	2.18[1.64, 2.79]	1.60E-88	5201	6.02[5.36, 6.68]	2.75[2.32, 3.19]	2.19[1.82, 2.62]	0.00E+00	6175
month=6	10.39[9.37, 11.4]	5.65[4.93, 6.37]	1.84[1.49, 2.26]	1.60E-88	5201	10.32[9.39, 11.24]	5.34[4.69, 5.97]	1.93[1.64, 2.3]	0.00E+00	6175
month=9	15.4[14.04, 16.75]	8.97[7.98, 9.95]	1.72[1.44, 2.09]	1.60E-88	5201	15.48[14.22, 16.73]	8.04[7.2, 8.88]	1.92[1.64, 2.25]	0.00E+00	6175
month=12	19.31[17.64, 20.94]	11.79[10.57, 12.99]	1.64[1.38, 1.96]	1.60E-88	5201	19.27[17.74, 20.76]	10.74[9.7, 11.77]	1.79[1.51, 2.02]	0.00E+00	6175
month=15	22.04[20.14, 23.9]	14.67[13.21, 16.09]	1.5[1.27, 1.79]	1.60E-88	5201	21.77[20.05, 23.45]	12.87[11.67, 14.06]	1.69[1.48, 1.89]	0.00E+00	6175
