# Supplementary material for: Real-world data analyses unveiled the immune-related adverse effects of immune checkpoint inhibitors across cancer types
Source: NPJ Precis Oncol. 2021 Sep 10;5:82. doi: 10.1038/s41698-021-00223-x (PMC8433190; doi:10.1038/s41698-021-00223-x)
Supplement: Supplementary file 2 — Reporting Summary [file 41698_2021_223_MOESM2_ESM.pdf]

nature research

Corresponding author(s): S. C. Kou and Kun-Hsing Yu

Last updated by author(s): Jul 24, 2021

Reporting Summary

Nature Research wishes to improve the reproducibility of the work that we publish. This form provides structure for consistency and transparency in reporting. For further information on Nature Research policies, see our Editorial Policies and the Editorial Policy Checklist.

Statistics

For all statistical analyses, confirm that the following items are present in the figure legend, table legend, main text, or Methods section.

n/a	Confirmed	
☐	☒	The exact sample size (n) for each experimental group/condition, given as a discrete number and unit of measurement
☐	☒	A statement on whether measurements were taken from distinct samples or whether the same sample was measured repeatedly
☐	☒	The statistical test(s) used AND whether they are one- or two-sided<br>*Only common tests should be described solely by name; describe more complex techniques in the Methods section.*
☐	☒	A description of all covariates tested
☐	☒	A description of any assumptions or corrections, such as tests of normality and adjustment for multiple comparisons
☐	☒	A full description of the statistical parameters including central tendency (e.g. means) or other basic estimates (e.g. regression coefficient) AND variation (e.g. standard deviation) or associated estimates of uncertainty (e.g. confidence intervals)
☐	☒	For null hypothesis testing, the test statistic (e.g. F, t, r) with confidence intervals, effect sizes, degrees of freedom and P value noted<br>*Give P values as exact values whenever suitable.*
☐	☒	For Bayesian analysis, information on the choice of priors and Markov chain Monte Carlo settings
☐	☒	For hierarchical and complex designs, identification of the appropriate level for tests and full reporting of outcomes
☐	☒	Estimates of effect sizes (e.g. Cohen's d, Pearson's r), indicating how they were calculated

Our web collection on statistics for biologists contains articles on many of the points above.

Software and code

Policy information about availability of computer code

Data collection	We used un-identifiable member claims data from Aetna, a nationwide managed care plan.
Data analysis	The detailed data analysis procedure is described in the Methods section.<br>We identified our patient cohorts, the treatments they received (chemotherapy, targeted therapy, or immunotherapy), and immune adverse events by SQL Server Management Studio (v17.9).<br>We performed the matching methods, conducted survival analysis, sensitivity analyses, and generated figures and table data using R (version 3.6.1).

For manuscripts utilizing custom algorithms or software that are central to the research but not yet described in published literature, software must be made available to editors and reviewers. We strongly encourage code deposition in a community repository (e.g. GitHub). See the Nature Research guidelines for submitting code & software for further information.

Data

Policy information about availability of data

All manuscripts must include a data availability statement. This statement should provide the following information, where applicable:

- Accession codes, unique identifiers, or web links for publicly available datasets
- A list of figures that have associated raw data
- A description of any restrictions on data availability

The codes that support the findings of this study are available from the authors upon reasonable request. The data was provided under license by Aetna, a nationwide managed care plan, and is not publicly available. Data are however available from and with permission of Aetna.

Field-specific reporting

Please select the one below that is the best fit for your research. If you are not sure, read the appropriate sections before making your selection.

☒ Life sciences ☐ Behavioural & social sciences ☐ Ecological, evolutionary & environmental sciences

For a reference copy of the document with all sections, see nature.com/documents/nr-reporting-summary-flat.pdf

Life sciences study design

All studies must disclose on these points even when the disclosure is negative.

Sample size	We conducted real-world data analyses using nationwide insurance claims data with 85.97 million enrollees across eight years. After our inclusion and exclusion criteria, we identified 92,858 patients for our study, which is sufficient for our planned analyses.
Data exclusions	The detailed inclusion and exclusion criteria is described in the Inclusion and Exclusion Criteria subsection in the Methods section. No ad-hoc data exclusion was performed.<br>We include a patient in our study only if he or she had at least 3 diagnostic codes of the same cancer type on different days within 18 consecutive months.<br>We excluded patients who have developed autoimmune-related phenotypes before they were diagnosed with cancer.<br>We further excluded patients who had chemotherapy, targeted therapy, or immunotherapy within 60 days of insurance enrollment.
Replication	Our analysis on observational data can be repeated with our codes and data source.
Randomization	Since we use observational data, we apply the matching methods to account for the confounders, which emulate a randomized trial.<br>We match exactly on both sex and race (when the information is available).<br>We require that the difference in age between the matched patients be less than 2 years.<br>Three other patient characteristics are also matched: the zip-code-derived median household income (derived from the Census Reporter), the rate of hospital visits before treatment initialization, and the rate of ICD codes before treatment initialization.
Blinding	Researchers who participated in the study design (S.C.K. and K.-H. Y.) were blinded to the group the patients belong to.

Reporting for specific materials, systems and methods

We require information from authors about some types of materials, experimental systems and methods used in many studies. Here, indicate whether each material, system or method listed is relevant to your study. If you are not sure if a list item applies to your research, read the appropriate section before selecting a response.

Materials & experimental systems

n/a	Involved in the study	
☒	☐	Antibodies
☒	☐	Eukaryotic cell lines
☒	☐	Palaeontology and archaeology
☒	☐	Animals and other organisms
☐	☒	Human research participants
☒	☐	Clinical data
☒	☐	Dual use research of concern

Methods

n/a	Involved in the study	
☒	☐	ChIP-seq
☒	☐	Flow cytometry
☒	☐	MRI-based neuroimaging

Human research participants

Policy information about studies involving human research participants

Population characteristics	We included patients with any of the 7 types of cancer (lung cancer, renal cancer, head and neck cancers, melanoma, brain cancer, basal cell carcinoma, and squamous cell carcinoma) who are treated with immunotherapy, chemotherapy, or targeted therapy. The detailed patient characteristics (age and gender) can be found in Table 1 and Supplemental Tables S8-S9.
Recruitment	We obtained observational data from participants in the Aetna insurance plan. Written informed consent was obtained from the participants.
Ethics oversight	This study was approved by the Harvard Medical School Institutional Review Board (IRB20-0957).

Note that full information on the approval of the study protocol must also be provided in the manuscript.
